# Bond-length distributions for ions bonded to oxygen: results for the transition metals and quantification of the factors underlying bond-length variation in inorganic solids

**DOI:** 10.1107/S2052252520005928

**Published:** 2020-06-09

**Authors:** Olivier Charles Gagné, Frank Christopher Hawthorne

**Affiliations:** a Earth and Planets Laboratory, Carnegie Institution for Science, Washington, D.C. 20015, USA; b Geological Sciences, University of Manitoba, 125 Dysart Road, Winnipeg, Manitoba R3T2N2, Canada

**Keywords:** bond-length variation, bond-topological effects, vibronic mixing, pseudo Jahn–Teller effect, materials design

## Abstract

Bond-length distributions are examined for 63 transition metal ions bonded to O^2−^ in 147 configurations, for 7522 coordination polyhedra and 41 488 bond distances, providing baseline statistical knowledge of bond lengths for transition metals bonded to O^2−^. *A priori* bond valences are calculated for 140 crystal structures containing 266 coordination polyhedra for 85 transition metal ion configurations with anomalous bond-length distributions. Two new indices, Δ_topol_ and Δ_cryst_, are proposed to quantify bond-length variation arising from bond-topological and crystallographic effects in extended solids. Bond-topological mechanisms of bond-length variation are (1) non-local bond-topological asymmetry and (2) multiple-bond formation; crystallographic mechanisms are (3) electronic effects (with an inherent focus on coupled electronic vibrational degeneracy in this work) and (4) crystal-structure effects. The indices Δ_topol_ and Δ_cryst_ allow one to determine the primary cause(s) of bond-length variation for individual coordination polyhedra and ion configurations, quantify the distorting power of cations via electronic effects (by subtracting the bond-topological contribution to bond-length variation), set expectation limits regarding the extent to which functional properties linked to bond-length variation may be optimized in a given crystal structure (and inform how optimization may be achieved) and more. These indices further provide an equal footing for comparing bond-length variation and the distorting power of ions across ligand types, including resolution for heteroligand polyhedra. The observation of multiple bonds is found to be primarily driven by the bond-topological requirements of crystal structures in solids. However, sometimes multiple bonds are observed to form as a result of electronic effects (*e.g.* the pseudo Jahn–Teller effect, PJTE); resolution of the origins of multiple-bond formation follows calculation of the Δ_topol_ and Δ_cryst_ indices on a structure-by-structure basis. Non-local bond-topological asymmetry is the most common cause of bond-length variation in transition metal oxides and oxysalts, followed closely by the PJTE. Non-local bond-topological asymmetry is further suggested to be the most widespread cause of bond-length variation in the solid state, with no *a priori* limitations with regard to ion identity. Overall, bond-length variations resulting from the PJTE are slightly larger than those resulting from non-local bond-topological asymmetry, comparable with those resulting from the strong JTE, and less than those induced by π-bond formation. From a comparison of *a priori* and observed bond valences for ∼150 coordination polyhedra in which the strong JTE or the PJTE is the main reason underlying bond-length variation, the JTE is found *not* to have a cooperative relation with the bond-topological requirements of crystal structures. The magnitude of bond-length variation caused by the PJTE decreases in the following order for octahedrally coordinated *d* ^0^ transition metal oxyanions: Os^8+^ > Mo^6+^ > W^6+^ >> V^5+^ > Nb^5+^ > Ti^4+^ > Ta^5+^ > Hf^4+^ > Zr^4+^ > Re^7+^ >> Y^3+^ > Sc^3+^. Such ranking varies by coordination number; for [4] it is Re^7+^ > Ti^4+^ > V^5+^ > W^6+^ > Mo^6+^ > Cr^6+^ > Os^8+^ >> Mn^7+^; for [5] it is Os^8+^ > Re^7+^ > Mo^6+^ > Ti^4+^ > W^6+^ > V^5+^ > Nb^5+^. It is concluded that non-octahedral coordinations of *d* ^0^ ion configurations are likely to occur with bond-length variations that are similar in magnitude to their octahedral counterparts. However, smaller bond-length variations are expected from the PJTE for non-*d* ^0^ transition metal oxyanions.

## Abstract 2

Bond-length distributions are examined for transition metals bonded to O^2−^. Causal mechanisms underlying bond-length variation in inorganic solids are resolved, and the extent to which these mechanisms individually result in bond-length variation is quantified for transition metals with anomalous bond-length distributions.

## 1. Introduction   

Transition metals are a unique set of elements whose compounds have an extraordinarily varied range of chemical and physical properties. The behaviour of transition metal compounds is characterized by the metastability of partially filled *d* orbitals, affording them distinctive electronic, magnetic, vibronic, optical and other properties of fundamental and technological interest. For instance, the wide array of metastable oxidation states characteristic of transition metals facilitates electron-transfer reactions central to catalysis (Fukuzumi, 2001 ▸), while metastable spin states associated with *d*-orbital occupancy are used as bistable atomic switches in spin-crossover compounds, controllable via external perturbations (Halcrow, 2013 ▸; Guionneau, 2014 ▸; Senthil Kumar & Ruben, 2017 ▸), and whose lifetime may be increased by several orders of magnitude via coupled electronic vibrational degeneracy (Garcia-Fernandez & Bersuker, 2011 ▸).

The functional properties of materials are often linked to irregular bond distances; some of these properties necessarily arise from non-centrosymmetric behaviour, *e.g.* piezoelectricity, ferroelectricity, pyroelectricity, second-harmonic generation response and dielectric behaviour (Halasyamani & Poeppelmeier, 1998 ▸; Halasyamani, 2004 ▸; Wu *et al.*, 2019 ▸), while others are sometimes simply enhanced by it, *e.g.* ferromagnetism (Coey, 2005 ▸), flexoelectricity (Wang *et al.*, 2012 ▸), negative thermal expansion (Marinkovic *et al.*, 2009 ▸), the photovoltaic effect (Yin *et al.*, 2015 ▸), photoluminescence (Chen *et al.*, 2017 ▸), photocatalysis (Kudo & Hijii, 1999 ▸), thermoelectricity (Lai *et al.*, 2015 ▸) and magnetic dielectric bistability (Bersuker, 2017 ▸). As such, deciphering the causal mechanisms underlying bond-length variation, and the extent to which bond lengths vary in solids, has significant implications in the materials sciences. For one thing, systematization of chemical bonding behaviour via large-scale bond-length dispersion analysis facilitates tracing anomalous bonding behaviour to the causal mechanisms underlying material properties, and further facilitates recognition of anomalously bonded coordination units bearing functional properties for their transposition into new chemical spaces. Further resolving the extent to which these mechanisms affect bond-length variation is crucial in order to maximize the harnessing of these effects within the constraints of physically realistic crystal structures. In addition, knowledge derived from large-scale bond-length dispersion analysis facilitates ion identification in crystal-structure refinements (with additional help from the bond-valence model), as the metrics of bonding behaviour are often characteristic of an ion configuration, particularly for transition metals. This information facilitates quantitative resolution of disordered and/or mixed-valent site occupancy in crystals, with particular relevance to understanding the mineralogical makeup of Earth and other planetary bodies, and the many geological processes we may infer from them.

The growing use of crystal-structure databases in the 1980s resulted in many sizeable bond-length dispersion analyses whose publication impacted fields such as organic chemistry (Allen *et al.*, 1987 ▸), coordination chemistry (Mayer, 1988 ▸; Orpen *et al.*, 1989 ▸) and protein crystallography (Engh & Huber, 1991 ▸; Laskowski *et al.*, 1993 ▸). Many such studies emerged from the Cambridge Structure Database (CSD; Groom *et al.*, 2016 ▸), which has been key for demonstrating the considerable potential of database analysis in the structural sciences (Allen & Motherwell, 2002 ▸; Groom & Allen, 2014 ▸). Although similar large-scale studies were done for inorganic crystals in the 1970s and 1980s (Shannon, 1976 ▸; Brown & Altermatt, 1985 ▸), publication of raw data and their statistics has been lacking. For reasons unknown, no large-scale bond-length dispersion analysis of inorganic compounds has been published since the development of the Inorganic Crystal Structure Database (ICSD; http://icsd.fiz-karlsruhe.de/icsd/) in the late 1970s. Recently, Waroquiers *et al.* (2017 ▸) analysed the ICSD to derive coordination-environment statistics in oxides and oxysalts but stopped short of investigating constituent bond lengths. It has been the primary goal of our work to provide baseline statistical knowledge of bond lengths in inorganic solids, such that the underlying reasons for variation may be rigorously examined. While this series has focused on bonds to oxygen, it is desirable that similar studies be done in the future for other anions; Gagné recently published a similar study for cations bonded to N^3−^ in inorganic compounds (Gagné, 2020 ▸).

This article is the fifth and last of a series in which we describe bond-length data for ions bonded to oxygen in inorganic crystals. In this series, we have examined the distribution of bond lengths for 135 ions bonded to oxygen in 460 configurations (on the basis of coordination number), using 177 446 bond lengths extracted from 9210 crystal structures refined since 1975; these data cover most ions of the periodic table and the coordination environments in which they occur in inorganic compounds. Here, we report bond-length data for 63 transition metal ions bonded to O^2−^ in 147 configurations, using 41 488 bond lengths and 7522 coordination polyhedra taken from 3814 crystal structure refinements. As we have done for the previous articles of this series (Gagné & Hawthorne, 2016 ▸; Gagné, 2018 ▸; Gagné & Hawthorne, 2018*a* ▸,*b* ▸), we deposit all bond-length data and their associated collection codes in the ICSD so that they may easily be used by others. For a description of data collection and filtering, we refer the reader to the first article of this series (Gagné & Hawthorne, 2016 ▸).

## 2. Scope of this work   

There are three objectives in this work: (I) to provide a comprehensive description of bond-length variations for transition metals bonded to O^2−^; (II) to resolve the causal mechanisms underlying bond-length variation for transition metals bonded to O^2−^; (III) to quantify the extent to which causal mechanisms result in bond-length variation for those transition metal configurations with anomalous bond-length distributions. We split this article into three parts in accord with these objectives.

The information we derive in (I) will provide crystallographers in a broad sense with more comprehensive and accurate bond-length data than are currently achievable via addition of ionic radii. These data are useful for refining and interpreting new crystal structures (particularly Rietveld refinements), modelling crystal structures and assessing the validity of computational studies, without which much effort is wasted on unrealistic atomic arrangements (Richardson, 2013 ▸; Zunger, 2019 ▸). Some implications for (II) and (III) were discussed in the *Introduction*; essentially, the resolution and quantification of anomalous bonding behaviour will facilitate the targeted design of materials whose functional properties are linked to asymmetric coordination environments, and will further facilitate optimization of these properties within the constraints of physically realistic crystal structures.

## 3. Part I. Bond-length dispersion analysis   

Data collection and filtering criteria were described in the first part of this series (Gagné & Hawthorne, 2016 ▸, Section 4.2), along with the method used for the particularly difficult task of validating excessively long bonds (Sections 3.2 and 3.3 therein). We stress that the bond-length data presented throughout this series are those which follow these collection and filtering criteria. Our datasets will necessarily evolve with the refinement of new crystal structures (and their more precise refinement); shorter and longer bonds will eventually be observed, and so will new ion configurations. For example, we give bond-length data for 149 new ion configurations throughout this series, in comparison to the seminal work of Shannon (1976 ▸) (30 new ion configurations for transition metals therein).

**Figure 1:**
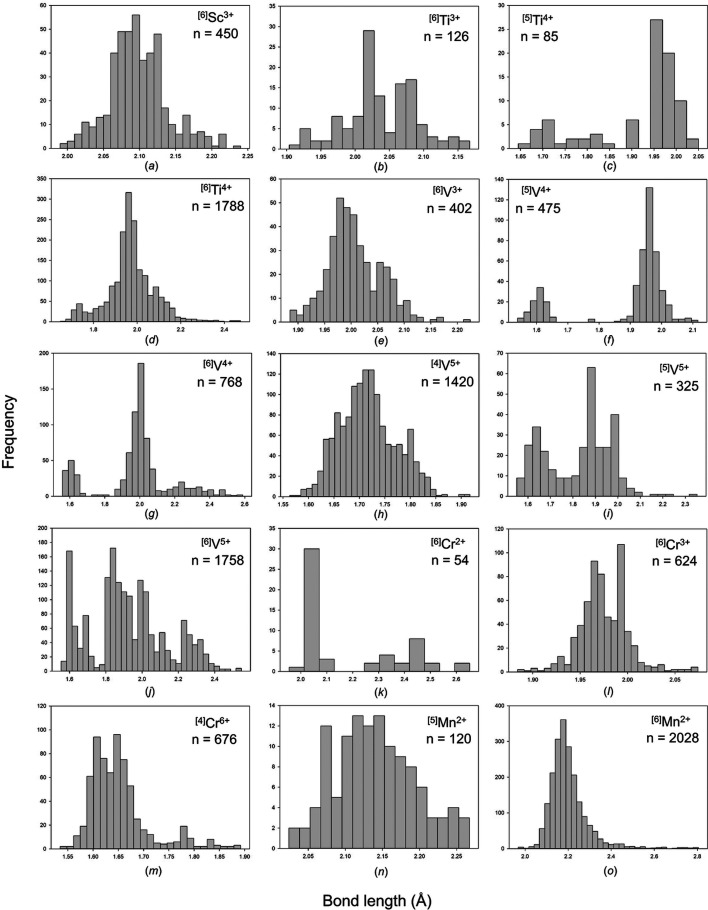

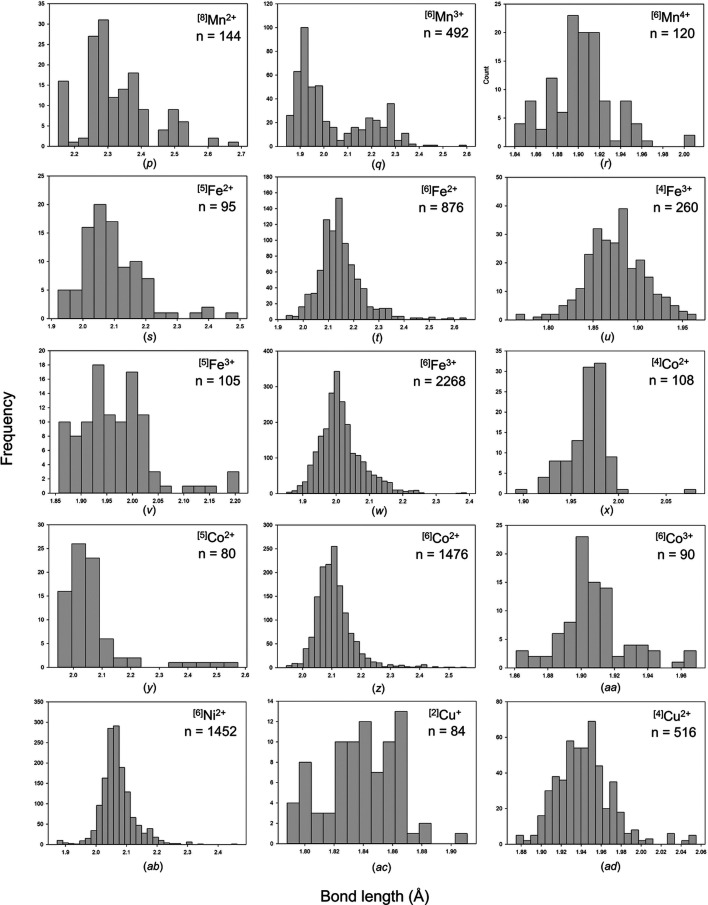

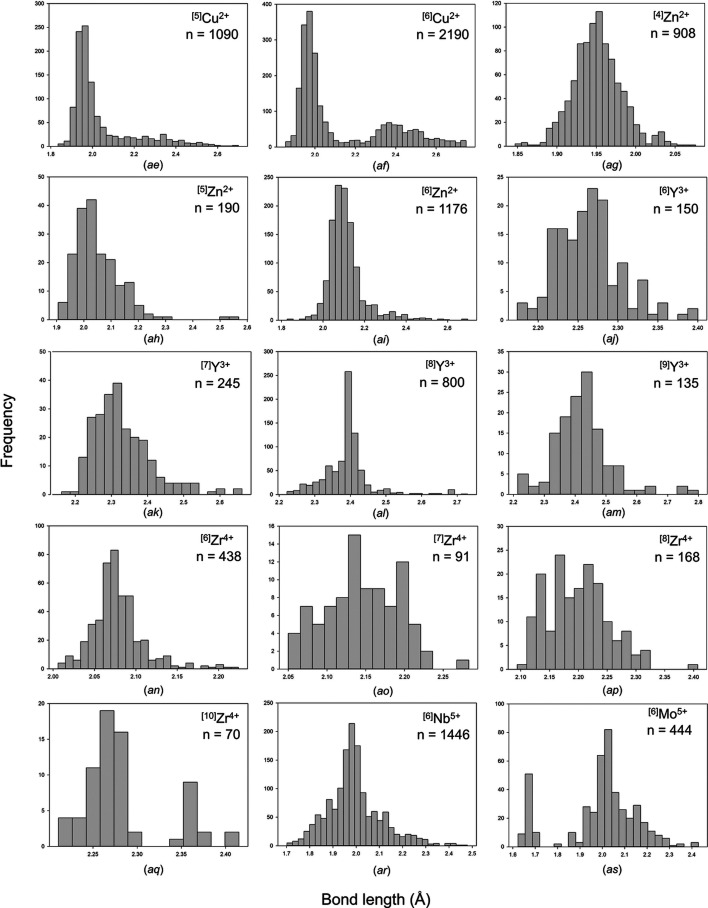

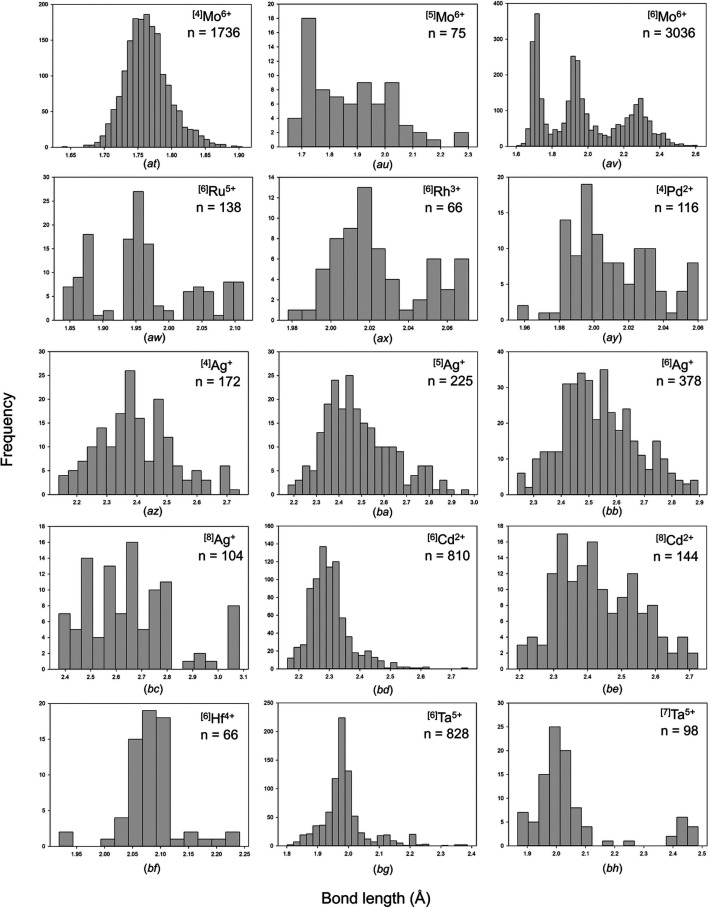

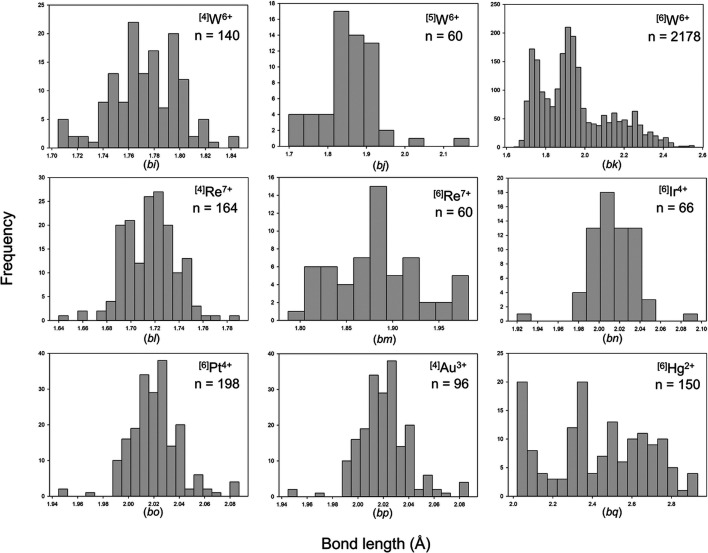
Bond-length distributions for selected configurations of the transition metal ions bonded to O^2−^: (*a*) ^[6]^Sc^3+^, (*b*) ^[6]^Ti^3+^, (*c*) ^[5]^Ti^4+^, (*d*) ^[6]^Ti^4+^, (*e*) ^[6]^V^3+^, (*f*) ^[5]^V^4+^, (*g*) ^[6]^V^4+^, (*h*) ^[4]^V^5+^, (*i*) ^[5]^V^5+^, (*j*) ^[6]^V^5+^, (*k*) ^[6]^Cr^2+^, (*l*) ^[6]^Cr^3+^, (*m*) ^[4]^Cr^6+^, (*n*) ^[5]^Mn^2+^, (O) ^[6]^Mn^2+^, (*p*) ^[8]^Mn^2+^, (*q*) ^[6]^Mn^3+^, (*r*) ^[6]^Mn^4+^, (*s*) ^[5]^Fe^2+^, (*t*) ^[6]^Fe^2+^, (*u*) ^[4]^Fe^3+^, (*v*) ^[5]^Fe^3+^, (*w*) ^[6]^Fe^3+^, (*x*) ^[4]^Co^2+^, (*y*) ^[5]^Co^2+^, (*z*) ^[6]^Co^2+^, (*aa*) ^[6]^Co^3+^, (*ab*) ^[6]^Ni^2+^, (*ac*) ^[2]^Cu^+^, (*ad*) ^[4]^Cu^2+^, (*ae*) ^[5]^Cu^2+^, (*af*) ^[6]^Cu^2+^, (*ag*) ^[4]^Zn^2+^, (*ah*) ^[5]^Zn^2+^, (*ai*) ^[6]^Zn^2+^, (*aj*) ^[6]^Y^3+^, (*ak*) ^[7]^Y^3+^, (*al*) ^[8]^Y^3+^, (*am*) ^[9]^Y^3+^, (*an*) ^[6]^Zr^4+^, (*ao*) ^[7]^Zr^4+^, (*ap*) ^[8]^Zr^4+^, (*aq*) ^[10]^Zr^4+^, (*ar*) ^[6]^Nb^5+^, (*as*) ^[6]^Mo^5+^, (*at*) ^[4]^Mo^6+^, (*au*) ^[5]^Mo^6+^, (*av*) ^[6]^Mo^6+^, (*aw*) ^[6]^Ru^5+^, (*ax*) ^[6]^Rh^3+^, (*ay*) ^[4]^Pd^2+^, (*az*) ^[4]^Ag^+^, (*ba*) ^[5]^Ag^+^, (*bb*) ^[6]^Ag^+^, (*bc*) ^[8]^Ag^+^, (*bd*) ^[6]^Cd^2+^, (*be*) ^[8]^Cd^2+^, (*bf*) ^[6]^Hf^4+^, (*bg*) ^[6]^Ta^5+^, (*bh*) ^[7]^Ta^5+^, (*bi*) ^[4]^W^6+^, (*bj*) ^[5]^W^6+^, (*bk*) ^[6]^W^6+^, (*bl*) ^[4]^Re^7+^, (*bm*) ^[6]^Re^7+^, (*bn*) ^[6]^Ir^4+^, (*bo*) ^[6]^Pt^4+^, (*bp*) ^[4]^Au^3+^ and (*bq*) ^[6]^Hg^2+^.

The collection and filtering criteria described in the first part of this series (Gagné & Hawthorne, 2016 ▸) resulted in a sample size of 41 488 bonds and 7522 coordination polyhedra for transition metal ions bonded to O^2−^. Table 1 ▸ gives the mean bond length and standard deviation, the minimum and maximum bond lengths (and ranges), the skewness and kurtosis (where justified by sample size), and the number of bonds and coordination polyhedra for the 63 transition metal ions observed in 147 configurations in terms of oxidation state and coordination number. All bond-length and bond-valence distributions are deposited in supplementary Figs. S1 and S2, respectively [we use the bond-valence parameters of Gagné & Hawthorne (2015 ▸) throughout this work]; bond-length distributions of adequate sample size (see below) are given in Fig. 1 ▸. As we have done in the previous parts of this series, we pay particular attention to confirming the reliability of the data at the limits of the bond-length distributions, *i.e.* the shortest and longest few bonds for each ion configuration. Anomalous bond lengths that result from positional and/or substitutional disorder, anomalous displacement parameters, uncorrected twinning effects *etc.* were removed from our dataset.

### 3.1. Effect of sample size   

To ensure the quality and reliability of the data reported throughout this series, we set stringent data collection and filtering criteria for the crystal structures used in our analysis, carefully scrutinized data populating the tails of reported bond-length distributions, and examined the effects of sampling on the reported data.

We previously described the typical shape of a bond-length distribution as a positively skewed Gaussian distribution which originates from the variation in Born repulsion and Coulomb attraction as a function of interatomic distance, *i.e.* that which results from a two-body Morse potential (Gagné & Hawthorne, 2016 ▸). In practice, this shape is seldom observed. Before we ascribe deviations in shape to causal mechanisms, it is crucial that we first understand the extent to which sample size influences the shape and statistics of bond-length distributions.

We examined the effects of sampling (*e.g.* the presence of outliers, non-random sampling) on the grand mean bond length (and its standard deviation), skewness and kurtosis for the alkali and alkaline earth metal ions bonded to O^2−^ in the first article of this series (Gagné & Hawthorne, 2016 ▸). We described the effect of sample size on these values for ^[6]^Na^+^ (Gagné & Hawthorne, 2016 ▸), ^[4]^S^6+^ and ^[6]^I^5+^ (Gagné & Hawthorne, 2018*b* ▸), ^[4]^Si^4+^ and ^[8]^Bi^3+^ (Gagné & Hawthorne, 2018*a* ▸), and ^[8]^La^3+^ bonded to O^2−^ (Gagné, 2018 ▸). We also showed a dependence of the grand mean bond length and the skewness and kurtosis values on (i) the number of data, (ii) the mean bond valence (Pauling bond strength) and (iii) the multi-modality of the bond-length distribution. Here, we report a similar analysis for ^[6]^Ti^4+^, with a mean bond valence of 0.67 v.u., which lies within a range of values not yet examined by other papers in this series.

**Figure 2:**
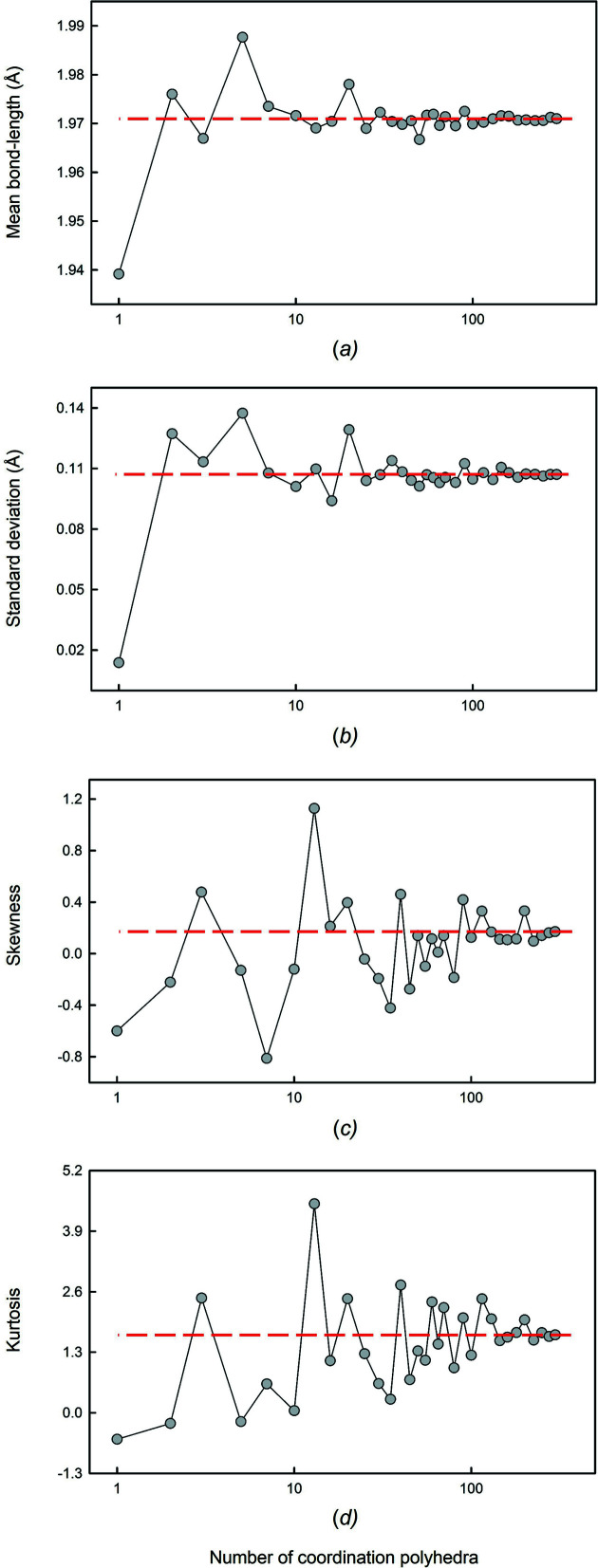
The effect of sample size on (*a*) mean bond length, (*b*) standard deviation of the mean bond length, (*c*) skewness and (*d*) kurtosis for ^[6]^Ti^4+^. The dashed line shows the value for the parent distribution.

Fig. 2 ▸ shows that for ^[6]^Ti^4+^, a sample size greater than 20 coordination polyhedra is required for the values of grand mean bond lengths to fluctuate by less than ±0.005 Å, while reliable values for skewness (±0.2) and kurtosis (±0.6) are obtained for sample sizes greater than ∼115 coordination polyhedra. Table 2 ▸ gives a summary for all ion configurations of this series analysed in such a manner; values for mean bond-length distributions are in parentheses, and ion configurations with multi-modal bond-length distributions (here caused by lone-pair stereoactivity) are shown in bold font. For the different ion configurations, we observe that fewer data are necessary to get an accurate estimate of the grand mean bond length with increasing mean bond strength. It is difficult to ascribe significance to the values of skewness and kurtosis; while these values are sometimes useful in describing well developed, smooth and generally ‘similar’ unimodal distributions, their extreme sensitivity to sample size requires caution in their interpretation. While multi-modal behaviour changes the minimum sample-size requirements significantly, we note that stronger bonds require smaller sample sizes to satisfy a given threshold. Skewness and kurtosis converge very rapidly for multi-modal bond-length distributions, as the bonding pattern of individual coordination polyhedra overwhelms the sensitivity to variability among polyhedra. We used the results of Table 2 ▸ to help decide whether or not to give values of skewness and kurtosis associated with bond-length and mean bond-length distributions in our work.

### 3.2. Variation in bond lengths and mean bond lengths   

When bonded to O^2−^, transition metals have an average range of bond lengths of 0.475 Å for ion configurations with sample sizes greater than ten coordination polyhedra (*n* = 74 ion configurations). As a function of electronic configuration, the average bond-length ranges are 0.492 (*d* ^0^), 0.736 (*d* ^1^), 0.399 (*d* ^2^), 0.221 (*d* ^3^), 0.505 (*d* ^4^), 0.391 (*d* ^5^), 0.338 (*d* ^6^), 0.414 (*d* ^7^), 0.245 (*d* ^8^), 0.646 (*d* ^9^) and 0.585 Å (*d* ^10^). For octahedral coordination (*n* = 33 ion configurations), these numbers are 0.585 (*d* ^0^), 0.788 (*d* ^1^), 0.399 (*d* ^2^), 0.221 (*d* ^3^), 0.755 (*d* ^4^), 0.517 (*d* ^5^), 0.264 (*d* ^6^), 0.614 (*d* ^7^), 0.589 (*d* ^8^), 0.893 (*d* ^9^), 0.756 Å (*d* ^10^). The wide variation in these bond-length ranges demonstrates inconsistent bonding behaviour as a function of electronic configuration and coordination number. This anomalous behaviour is, to some extent, expected from the Jahn–Teller effect (JTE); bond-length variations attributable to the JTE will be discussed in detail below, along with other significant causes of bond-length variation. The largest bond-length ranges are for ^[6]^V^4+^ (1.030 Å; *d* ^1^), ^[6]^Mo^6+^ (1.005 Å; *d* ^0^), ^[7]^Hg^2+^ (0.998 Å; *d* ^10^), ^[6]^V^5+^ (0.993 Å; *d* ^0^), ^[6]^W^6+^ (0.919 Å; *d* ^0^), ^[6]^Hg^2+^ (0.912 Å; *d* ^10^) and ^[6]^Cu^2+^ (0.893 Å; *d* ^9^), and it is notable that all these ion configurations show multimodal distributions.

**Figure 3:**
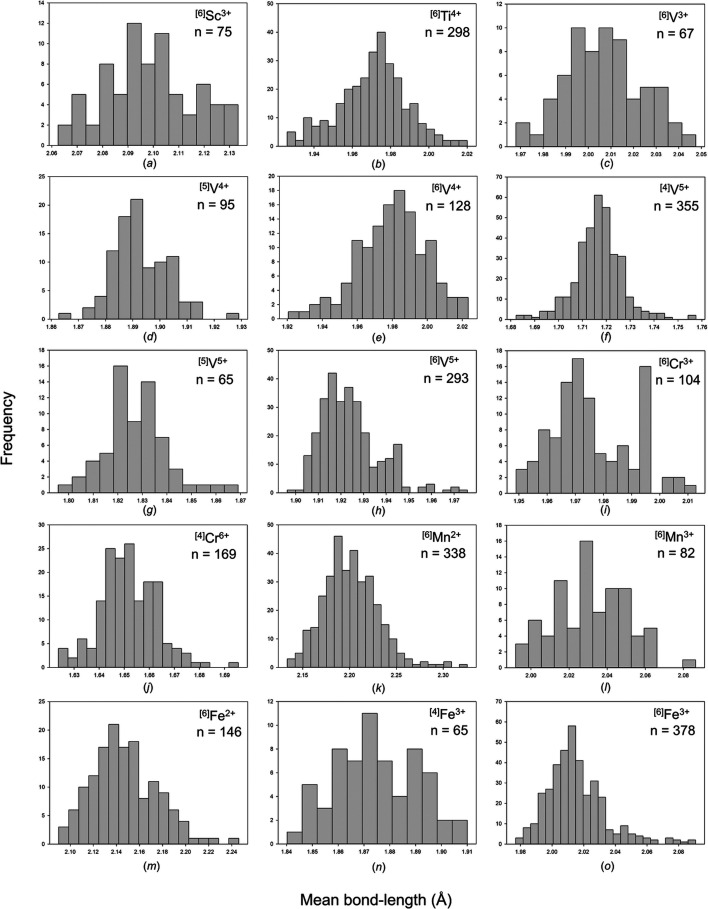

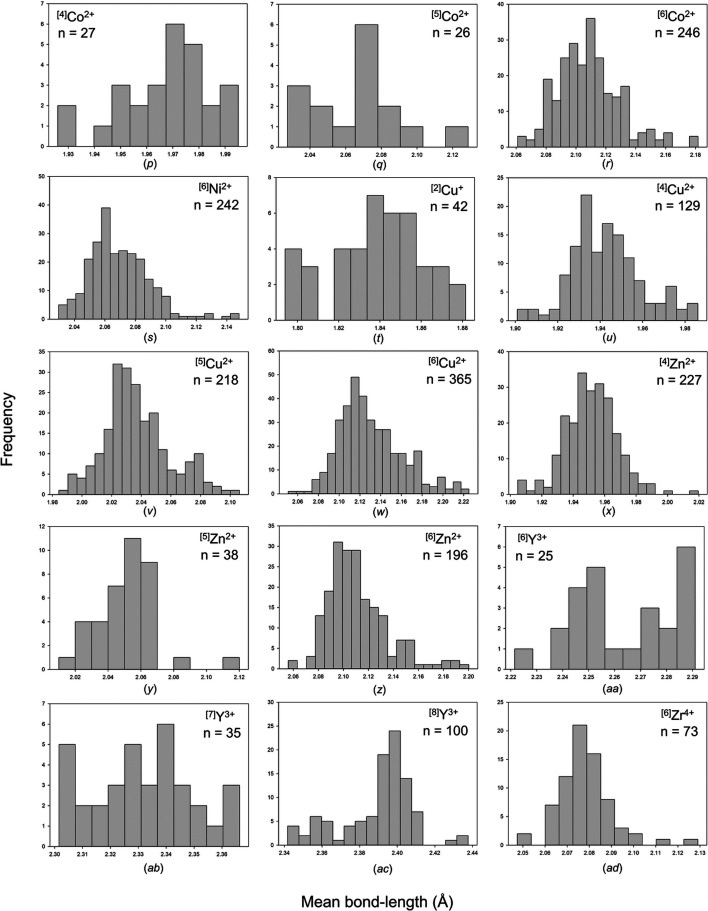

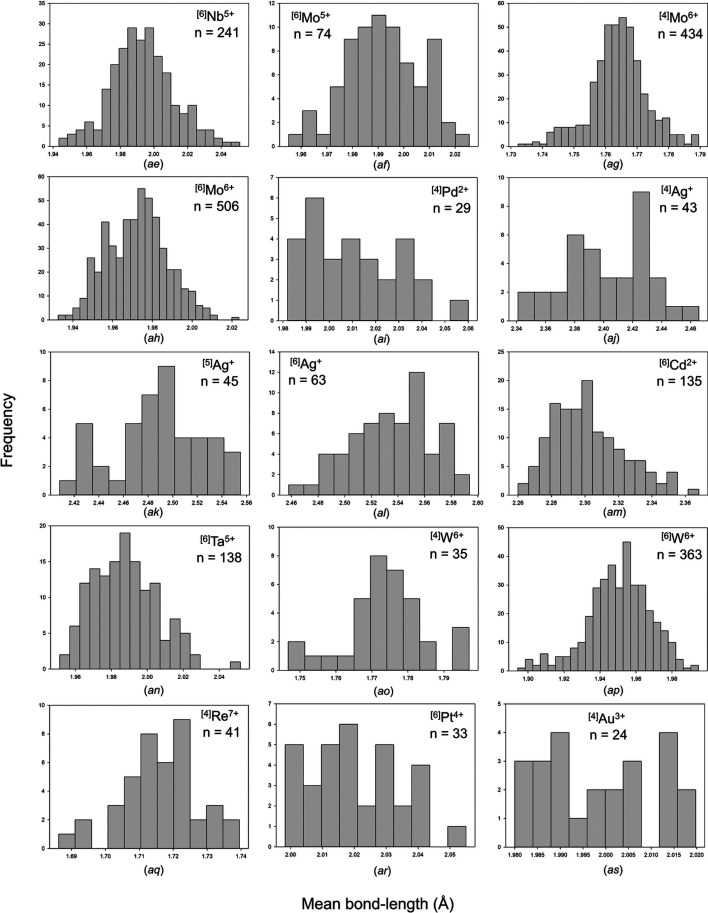

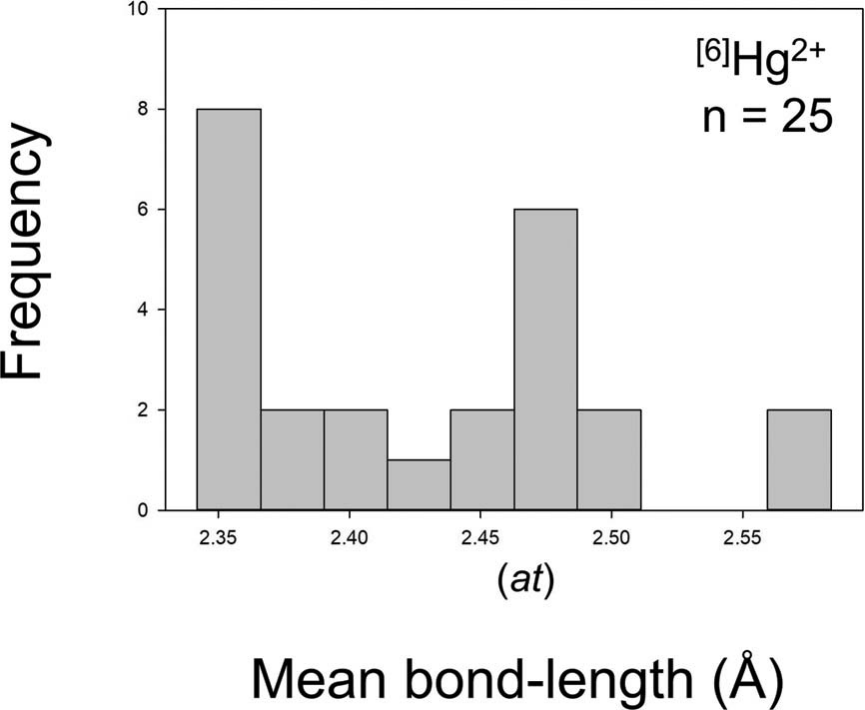
Mean bond-length distributions for selected configurations of the transition metal ions bonded to O^2−^: (*a*) ^[6]^Sc^3+^, (*b*) ^[4]^Ti^4+^, (*c*) ^[6]^V^3+^, (*d*) ^[5]^V^4+^, (*e*) ^[6]^V^4+^, (*f*) ^[4]^V^5+^, (*g*) ^[5]^V^5+^, (*h*) ^[6]^V^5+^, (*i*) ^[6]^Cr^3+^, (*j*) ^[4]^Cr^6+^, (*k*) ^[6]^Mn^2+^, (*l*) ^[6]^Mn^3+^, (*m*) ^[6]^Fe^2+^, (*n*) ^[4]^Fe^3+^, (O) ^[6]^Fe^3+^, (*p*) ^[4]^Co^2+^, (*q*) ^[5]^Co^2+^, (*r*) ^[6]^Co^2+^, (*s*) ^[6]^Ni^2+^, (*t*) ^[2]^Cu^+^, (*u*) ^[4]^Cu^2+^, (*v*) ^[5]^Cu^2+^, (*w*) ^[6]^Cu^2+^, (*x*) ^[4]^Zn^2+^, (*y*) ^[5]^Zn^2+^, (*z*) ^[6]^Zn^2+^, (*aa*) ^[6]^Y^3+^, (*ab*) ^[7]^Y^3+^, (*ac*) ^[8]^Y^3+^, (*ad*) ^[6]^Zr^4+^, (*ae*) ^[6]^Nb^5+^, (*af*) ^[6]^Mo^5+^, (*ag*) ^[4]^Mo^6+^, (*ah*) ^[6]^Mo^6+^, (*ai*) ^[4]^Pd^2+^, (*aj*) ^[4]^Ag^+^, (*ak*) ^[5]^Ag^+^, (*al*) ^[6]^Ag^+^, (*am*) ^[6]^Cd^2+^, (*an*) ^[6]^Ta^5+^, (*ao*) ^[4]^W^6+^, (*ap*) ^[6]^W^6+^, (*aq*) ^[4]^Re^7+^, (*ar*) ^[6]^Pt^4+^, (*as*) ^[4]^Au^3+^ and (*at*) ^[6]^Hg^2+^.

Mean bond-length distributions are given in Fig. S3, and those with adequate sample size (see Section 3.1) are given in Fig. 3 ▸. Table 3 ▸ gives the grand mean bond length and standard deviation, the minimum and maximum mean bond lengths (and ranges), the skewness and kurtosis of each distribution (where justified by sample size) and the number of coordination polyhedra for each configuration observed. When bonded to O^2−^, transition metals have an average range of mean bond lengths of 0.085 Å for sample sizes greater than ten coordination polyhedra. For octahedrally coordinated *d* ^0^ transition metals, this range is 0.078 Å (0.086 Å excluding Sc^3+^, Y^3+^, Zr^4+^, Hf^4+^), while that of ions exhibiting the classic JTE in octahedral coordination (weak or strong) is 0.097 Å. For the latter group, the largest mean bond-length ranges are observed for ^[6]^Cu^2+^ 0.174, ^[6]^V^4+^ 0.103, ^[6]^Mn^3+^ 0.094 and ^[6]^Mo^5+^ 0.070 Å. For the *d* ^0^ transition metals, the largest ranges (irrespective of coordination number, for sample sizes greater than ten coordination polyhedra) are ^[6]^Nb^5+^ 0.102, ^[6]^W^6+^ 0.100, ^[6]^Ta^5+^ 0.100, ^[8]^Y^3+^ 0.096, ^[6]^Ti^4+^ 0.094, ^[6]^Mo^6+^ 0.091 and ^[6]^Zr^4+^ 0.082 Å.

Despite the significant effect of the JTE on bond-length variation, its corresponding effect on mean bond length is not marked. The mean bond-length range observed for transition metals bonded to O^2−^ (0.085 Å) is typical of ions not showing electronic and/or crystal-structure effects, and is due to their high Lewis acidity (values given by Gagné & Hawthorne, 2017*a* ▸). For comparison, strongly bonded oxyanions have typical mean bond-length ranges of 0.06–0.10 Å (Gagné & Hawthorne, 2018*a* ▸,*b* ▸), actinides 0.07 Å and lanthanides 0.10 Å (Gagné, 2018 ▸); the ranges are larger for ions with stereoactive lone-pair electrons at ∼0.1–0.3 Å (Gagné & Hawthorne, 2018*a* ▸,*b* ▸), for alkaline earth metals ∼0.20–0.25 Å and for alkali metals ∼0.30–0.40 Å (Gagné & Hawthorne, 2016 ▸).

### 3.3. Bond-length distortion   

Bond-length distortion, defined as the mean-square relative deviation of bond lengths from their mean value (Brown & Shannon, 1973 ▸), is a common measure of bond-length dispersion used on the basis of individual coordination polyhedra. Although it is more a scalar index of bond-length dispersion than a measure of distortion, we retain the terminology ‘bond-length distortion’ for historical reasons (notably, the link between bond-length distortion and the distortion theorem of the bond-valence model, which regards the inherent increase in mean bond length with increasing bond-length dispersion; Brown, 1978 ▸).

**Figure 4:**
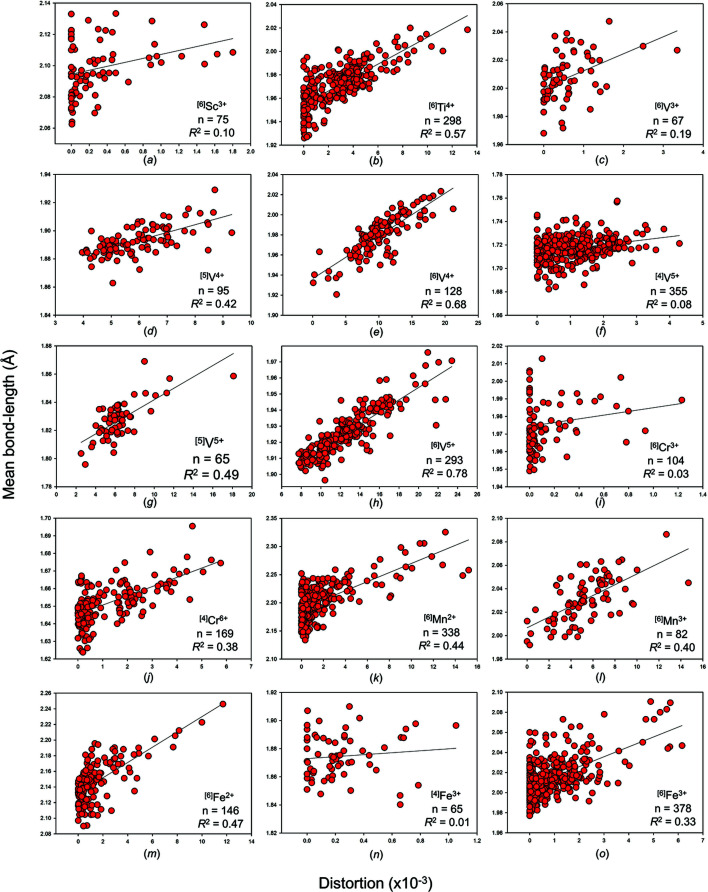

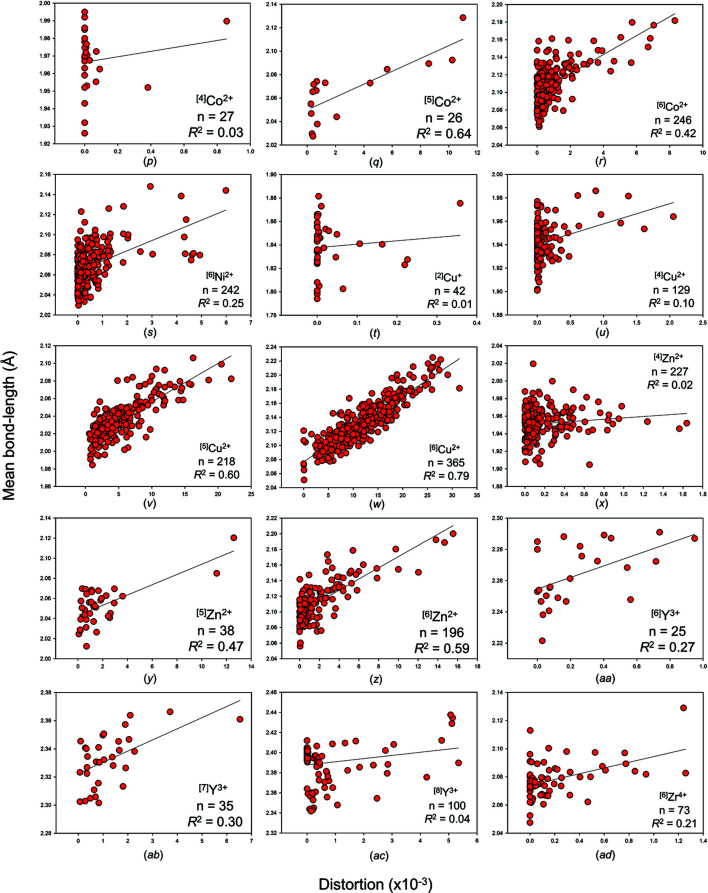

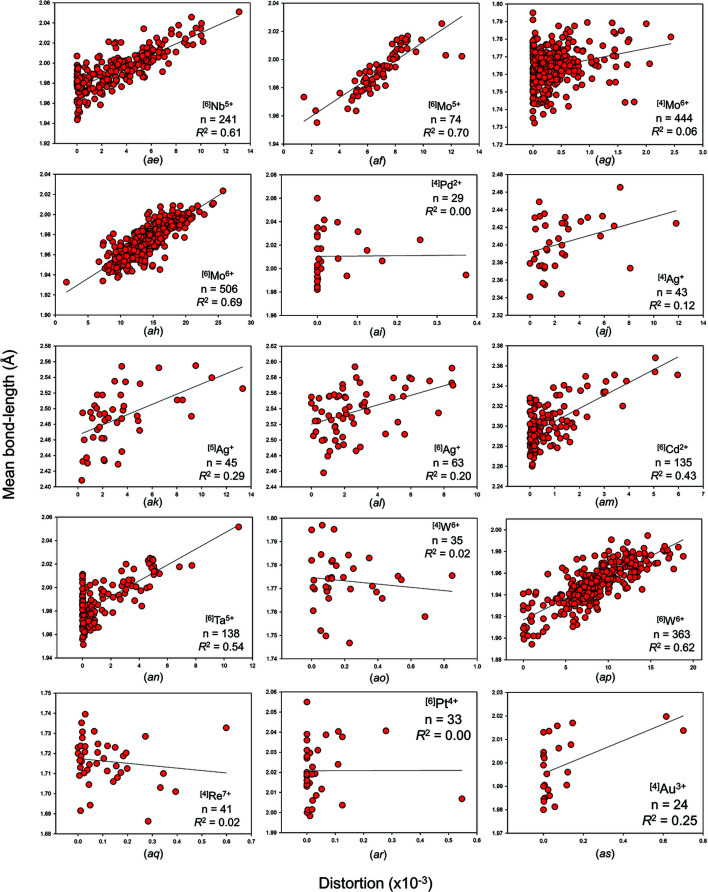

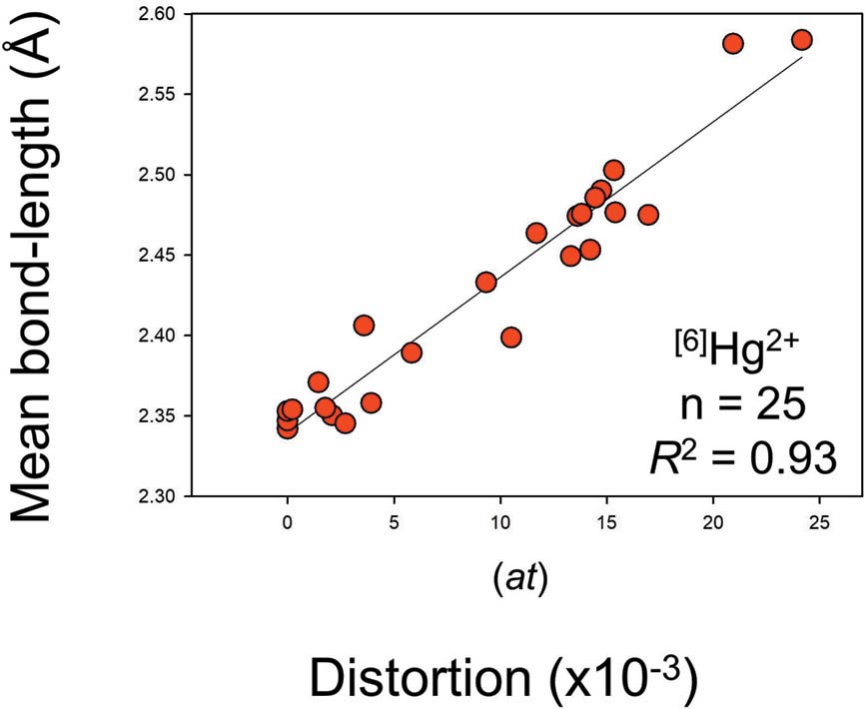
The effect of bond-length distortion on mean bond length for selected configurations of the transition metal ions bonded to O^2−^: (*a*) ^[6]^Sc^3+^, (*b*) ^[4]^Ti^4+^, (*c*) ^[6]^V^3+^, (*d*) ^[5]^V^4+^, (*e*) ^[6]^V^4+^, (*f*) ^[4]^V^5+^, (*g*) ^[5]^V^5+^, (*h*) ^[6]^V^5+^, (*i*) ^[6]^Cr^3+^, (*j*) ^[4]^Cr^6+^, (*k*) ^[6]^Mn^2+^, (*l*) ^[6]^Mn^3+^, (*m*) ^[6]^Fe^2+^, (*n*) ^[4]^Fe^3+^, (O) ^[6]^Fe^3+^, (*p*) ^[4]^Co^2+^, (*q*) ^[5]^Co^2+^, (*r*) ^[6]^Co^2+^, (*s*) ^[6]^Ni^2+^, (*t*) ^[2]^Cu^+^, (*u*) ^[4]^Cu^2+^, (*v*) ^[5]^Cu^2+^, (*w*) ^[6]^Cu^2+^, (*x*) ^[4]^Zn^2+^, (*y*) ^[5]^Zn^2+^, (*z*) ^[6]^Zn^2+^, (*aa*) ^[6]^Y^3+^, (*ab*) ^[7]^Y^3+^, (*ac*) ^[8]^Y^3+^, (*ad*) ^[6]^Zr^4+^, (*ae*) ^[6]^Nb^5+^, (*af*) ^[6]^Mo^5+^, (*ag*) ^[4]^Mo^6+^, (*ah*) ^[6]^Mo^6+^, (*ai*) ^[4]^Pd^2+^, (*aj*) ^[4]^Ag^+^, (*ak*) ^[5]^Ag^+^, (*al*) ^[6]^Ag^+^, (*am*) ^[6]^Cd^2+^, (*an*) ^[6]^Ta^5+^, (*ao*) ^[4]^W^6+^, (*ap*) ^[6]^W^6+^, (*aq*) ^[4]^Re^7+^, (*ar*) ^[6]^Pt^4+^, (*as*) ^[4]^Au^3+^ and (*at*) ^[6]^Hg^2+^.

Fig. S4 shows mean bond length as a function of bond-length distortion for all transition metal ions bonded to O^2−^, and Fig. 4 ▸ shows those of adequate sample size. Transition metal ions show a wide range of bond-length distortion when bonded to O^2−^, from weakly distorted (0–10 × 10^−3^) to moderately distorted (10–20 × 10^−3^) to highly distorted (>20 × 10^−3^). There is a strong correlation between bond-length distortion and mean bond length for moderately distorted ion configurations (10–20 × 10^−3^) or higher. In previous articles in this series, we found that the correlation between bond-length distortion and mean bond length is strong for ion configurations with values of distortion >20 × 10^−3^ for metalloids and post-transition metal ions bonded to O^2−^ (Gagné & Hawthorne, 2018*a* ▸), >10 × 10^−3^ for alkaline earth metal (Gagné & Hawthorne, 2016 ▸), non-metal (Gagné & Hawthorne, 2018*b* ▸) and actinide (Gagné, 2018 ▸) ions bonded to O^2−^, and <10 × 10^−3^ for lanthanide ions bonded to O^2−^ (Gagné, 2018 ▸). There is no particularly strong correlation between bond-length distortion and mean bond length for alkali metal ions bonded to O^2−^ (Gagné & Hawthorne, 2016 ▸).

#### 3.3.1. Causal mechanisms underlying mean bond-length variations   

Gagné & Hawthorne (2017*b* ▸) examined potential factors leading to mean bond-length variation for 55 ion configurations bonded to O^2−^, including 20 configurations for transition metals bonded to O^2−^: ^[6]^Ti^4+^, ^[6]^V^4+^, ^[4]^V^5+^, ^[6]^V^5+^, ^[6]^Cr^6+^, ^[6]^Mn^2+^, ^[6]^Fe^2+^, ^[6]^Fe^3+^, ^[6]^Co^2+^, ^[6]^Ni^2+^, ^[5]^Cu^2+^, ^[6]^Cu^2+^, ^[4]^Zn^2+^, ^[6]^Zn^2+^, ^[6]^Nb^5+^, ^[4]^Mo^6+^, ^[6]^Mo^6+^, ^[6]^Cd^2+^, ^[6]^Ta^5+^ and ^[6]^W^6+^. They found mean bond length to be correlated with bond-length distortion for 17 of those 20 ion configurations at a 99% confidence level (〈*R* ^2^〉 = 0.50), citing the distortion theorem (Brown, 1978 ▸) as the mechanism causing this correlation. Other factors investigated were found to be statistically insignificant, including the ionization energy and electronegativity of next-nearest neighbours, and the coordination number of the bonded anions, leading them to propose that the inability of crystal structures to attain their ideal (*a priori*) bond lengths within the constraints of space group and translational symmetry is the leading cause of mean bond-length variation in crystals. Below, we expand on their analysis to investigate the underlying causal mechanisms of bond-length variation in transition metal oxide and oxysalt crystals.

## 4. Part II. Resolving the principal mechanisms underlying bond-length variation   

Transition metal oxides and oxysalts are ideally suited for examining bond-length variations in solids as they are highly susceptible to two important mechanisms underlying bond-length variation: (i) coupled electronic vibrational degeneracy (leading to the JTE) and (ii) formation of multiple (π) bonds. In recent years, the non-local bond-topological asymmetry of coordination environments (sometimes referred to as asymmetry in the bond network) has been proposed as an additional mechanism underlying bond-length variation in crystal structures (Kunz & Brown, 1995 ▸; Bosi, 2014 ▸). In this section, we exploit the size and comprehensiveness of our bond-length dispersion analysis to resolve the various causal mechanisms underlying bond-length variation in transition metal oxides and oxysalts by way of rationalizing the shape of anomalous bond-length distributions (Fig. S1). We further summarize the theoretical underpinnings of these mechanisms.

The discussion for the present section is split into four subsections reflecting the principal causal mechanisms identified in this work: (1) non-local bond-topological effects, (2) multiple-bond formation, (3) electronic effects (with an inherent focus on coupled electronic vibrational degeneracy) and (4) crystal-structure effects. As shown below, mechanisms (1)–(3) may each lead to large bond-length variations and/or multi-modal bond-length distributions. However, these effects rarely occur in isolation, and their combination is often what leads to wide variations in bond lengths. It is worth noting that these mechanisms may be present in a limited number of crystal structures, or in all crystal structures in which the given ion configuration occurs. As a result, the shape of a bond-length distribution depends on the relative importance/magnitude of the effect(s) sampled. Thus, we focus our attention below on quantifiable features that may be recognized within the constituent data of these bond-length distributions.

In Table 4 ▸, we list 52 of the most interesting transition metal ion configurations observed in this work, for either (i) having a shape that departs prominently from that expected for a two-body Morse potential, (ii) displaying a very wide range of observed bond lengths and/or (iii) being of interest to some of the more general questions addressed in this work. These are the data we focus on for Parts II and III of this work. In Part III, we will identify the causal mechanism(s) underlying bond-length variation for each of these ion configurations and quantify their extent.

### 4.1. Mechanism (1): Non-local bond-topological effects   

The connection between bond topology and bond-length variation was first demonstrated by Kunz & Brown (1995 ▸). Unfortunately, a lack of follow-up work left unclear the extent to which this mechanism operates in terms of frequency of occurrence and magnitude of bond-length variation, leading few to acknowledge this mechanism as a noteworthy driver of bond-length variation in inorganic solids. We dedicate a significant part of this work to resolving this issue: we will clarify the mechanism of bond-length variation via bond-topological arguments using the bond-valence model, introduce new indices to quantify the effect of bond topology on bond-length variation, and provide worked examples to show the prevalence and scope of this effect.

#### 4.1.1. The bond-valence model   

The bond-valence model is an electrostatic model of chemical bonding used extensively in the study of minerals and inorganic materials (Brown, 2016 ▸). The main axioms of the bond-valence model, analogous to Kirchhoff’s rules for electrical circuits, are: (i) the *valence-sum rule*, which states that the sum of the directed bond valences around an ion is equal to its oxidation state (essentially, a modernization of Pauling’s second rule), and (ii) the *path rule*, which states that the sum of the directed bond valences along any path of bonds in a structure is zero when the path begins and ends on symmetrically equivalent ions (Gagné *et al.*, 2018 ▸). Although the model finds many applications both in solution and in the solid state (summarized by Brown, 2009 ▸, 2016 ▸), its most common use is to serve as a check on newly refined crystal structures via verification of the valence-sum rule.

Key to this model is the relation between the length of a bond and its strength (called its *bond valence*):
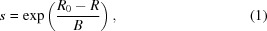
where *s* is the bond valence for a bond of length *R*, and *R*_0_ and *B* are the bond-valence parameters of the ion pair. The bond-valence parameters are constants that are typically derived empirically; large sets of bond-valence parameters include those of Gagné & Hawthorne (2015 ▸), Brese & O’Keeffe (1991 ▸) and Brown & Altermatt (1985 ▸). From this, the valence-sum rule can then be written as
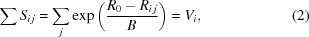
where *V*_*i*_ is the oxidation state of ion *i* (sometimes called the formal valence), and where the sum is taken over the *j* bonds involving ion *i*. For structure verification, deviation up to ∼6–7% is deemed acceptable for any given site of a crystal structure (Gagné & Hawthorne, 2015 ▸). This variation cannot be removed from the model, and is largely due to the effect of structure type on mean bond-length variation (Gagné & Hawthorne, 2017*b* ▸), discussed as causal mechanism (4) in the present work. We have used equation (2) throughout this series to spot possible experimental errors and oversights (*e.g.* substitutional disorder) to remove doubtful data from our dataset; similarly, equation (2) is used as a screening criterion in *pymatgen* (Ong *et al.*, 2013 ▸), the analysis code powering the Materials Project (Jain *et al.*, 2013 ▸). In addition, the valence-sum rule allows one to infer the oxidation state of redox-active ions (Shields *et al.*, 2000 ▸; Wood *et al.*, 2000 ▸; Roulhac & Palenik, 2003 ▸; Reeves *et al.*, 2019 ▸), which is particularly relevant for confirming the oxidation state of transition metals and resolving mixed-valence site occupancy.

Of greatest relevance to this work is that the bond-topological underpinnings of the bond-valence model allow prediction of the *a priori* bond valences of crystal structures (Gagné *et al.*, 2018 ▸). Within the framework of the bond-valence model, one may enumerate a finite set of linearly independent equations for the valence-sum and path rules (defined above) in terms of constituent bond valences. These are collectively called *network equations*, and can be solved simultaneously for a specific bond topology to calculate its *a priori* bond valences, defined as the ideal (theoretical) bond valences intrinsic to a crystal structure. *A priori* bond valences depend only on the formal valences (oxidation states) of the ions at the sites in the structure and the connectivity of chemical bonds in the structure (*i.e.* the bond topology). Similarly, *a priori* bond lengths are defined as the ideal (theoretical) bond lengths intrinsic to a given site assignment for a crystal structure, and are obtained via the conversion of *a priori* bond valences using equation (1). *A priori* bond lengths further depend on the identity of the site occupants, due to their dependence on the bond-valence parameters *R*_0_ and *B* in equation (1).

For example, let us consider the *Pnma* perovskite crystal structure, with two crystallographically distinct cation (*A* and *B*) and anion (*X*1 and *X*2) sites, *e.g. ^A^*Gd^3+*B*^Mn^3+^O_3_ (ICSD refcode 95493; Mori *et al.*, 2002 ▸). The bond topology is as follows: *A* makes two bonds to *X*1 and six bonds to *X*2, *B* makes two bonds to *X*1 and four bonds to *X*2, *X*1 makes two bonds to *A* and two bonds to *B*, and *X2* makes three bonds to *A* and two bonds to *B* (Table 5 ▸). Denoting the *A*—*X*1 bonds as ‘a’, *A*—*X*2 bonds as ‘b’, *B*—*X*1 bonds as ‘c’ and *B*—*X*2 bonds as ‘d’ valence units in strength, we derive the valence-sum equations 2a + 6b = 3 v.u., 2c + 4d = 3 v.u., 2a + 2c = 2 v.u. and 3b + 2d = 2 v.u., and the path equation a − c + d − b = 0 v.u. [full details of the method, as well as a suite of calculations for increasingly complex crystal structures, are given by Gagné *et al.* (2018 ▸)]. Thus, the *a priori* bond valences of this structure and charge assignment are a = 0.441, b = 0.353, c = 0.559 and d = 0.471 v.u. One may then convert these values into *a priori* bond lengths, using equation (1) and the appropriate bond-valence parameters (*R*_0_ = 1.988 and *B* = 0.433 v.u. for Gd^3+^, and *R*_0_ = 1.823 and *B* = 0.247 v.u. for Mn^3+^; Gagné & Hawthorne, 2015 ▸), to obtain a = 2.343, b = 2.439, c = 1.966 and d = 2.009 Å (Table 5 ▸).

As we show below, crystal structures often have intrinsic requirements for an uneven distribution of bond valences (and thus bond lengths). Next, we investigate the inner workings of this phenomenon and the extent to which it results in bond-length variation for transition metals bonded to O^2−^.

#### 4.1.2. Non-local bond-topological asymmetry   

**Figure 5:**
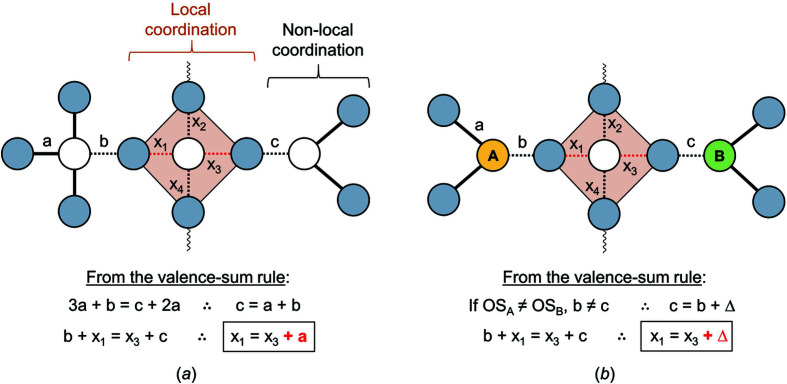
Inherent bond-valence (and thus bond-length) variation resulting from the valence-sum rule for (*a*) different coordination numbers and (*b*) different oxidation states (OS) of next-nearest neighbours for a simple bond topology. Grey circles are anions. White circles are cations of the same oxidation state and coloured circles are cations of different oxidation states. The fragment shown is self-contained; wavy lines indicate further bonds which are inconsequential to x_1_ and x_3_. Black bonds are terminal and consequently equal to the oxidation state of the anion, ‘a’; from here, ‘b’ and ‘c’ are deduced, and x_1_ and x_3_ are shown to be necessarily unequal in strength, thus causing bond-length variation within the local coordination.

In the bond-valence model, the principle of maximum symmetry states that a system in stable equilibrium adopts the highest symmetry consistent with the constraints acting on it (Brown, 2014 ▸). These constraints include crystal-chemical and thermodynamics factors not limited to peculiar electronic behaviour, *T*–*P* stability ranges, rate of crystallization, external field(s) *etc*. Where such constraints are absent or have negligible effect, a crystal structure is observed with the lowest possible number of crystallographically distinct sites, *i.e.* equal to the number of distinct elements in the compound (*e.g.* for spinel, MgAl_2_O_4_). In this configuration, cations and anions distribute their *a priori* (ideal) bond valences evenly [see Gagné *et al.* (2018 ▸) for their calculation], resulting in coordination polyhedra with identical or similar bond lengths. With considerable constraints acting on the system at the time of crystallization, crystallographically distinct sites may rapidly outgrow the number of distinct elements in the compound. The decrease in crystallographic symmetry inherently results in increasingly asymmetric patterns of *a priori* bond valences [thus *a priori* bond lengths, equation (1)] for cation and anion polyhedra, increasing the potential for large bond-length variations within those polyhedra. We term this phenomenon *non-local bond-topological asymmetry*, where the variation in bond lengths does not originate from the local bond topology (the coordination polyhedron), but rather from asymmetry elsewhere in the bond topology, either in the form of varying coordination number(s) or ion identity (Fig. 5 ▸).

**Figure 6:**
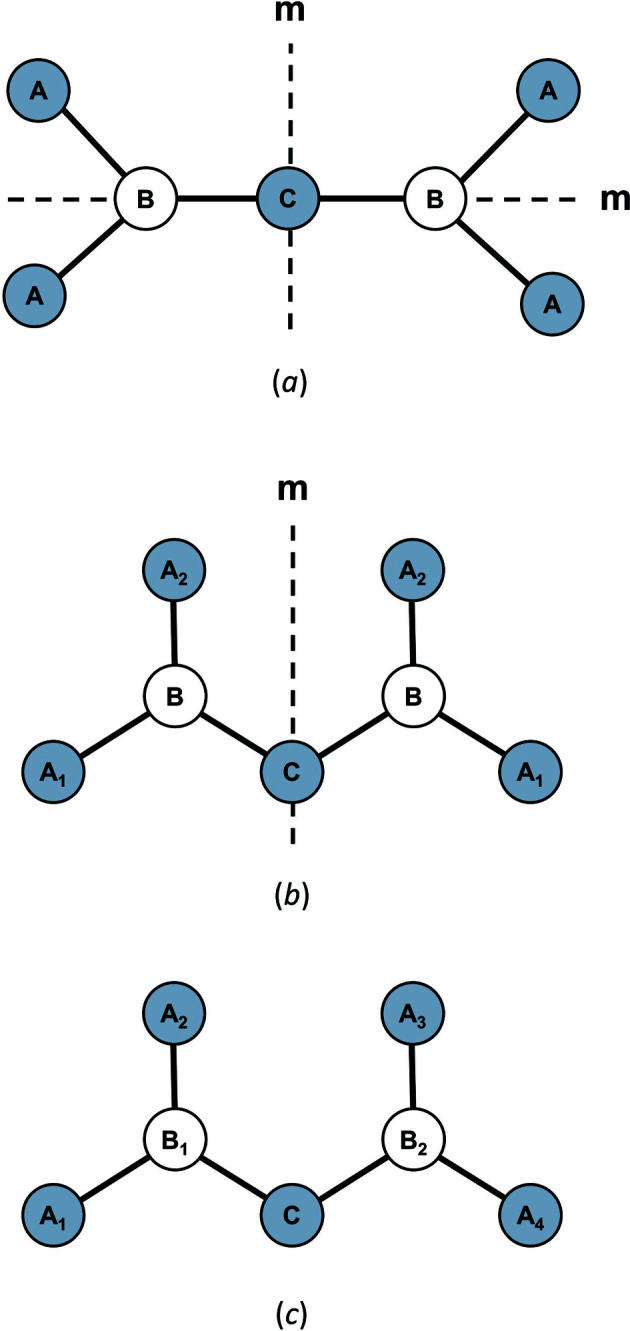
A demonstration of bond-topological and crystallographic equivalence for the ions of a dimeric unit (cations = white, anions = grey). Topologically equivalent ions share the same lettering, and crystallographically equivalent ions share the same lettering and subscript. Crystallographic equivalence of the constituent ions lowers from (*a*) the configuration of maximum symmetry about ion C to (*c*) the point of minimum crystallographic symmetry. Intermediate configurations are observed with progressively fewer symmetry operators (m = mirror plane), thus lowering the number of equivalent metrics (bond lengths, bond angles) from configurations (*a*) to (*c*). Bond-topological equivalence is unchanged from (*a*) to (*c*).

We emphasize that while crystallographically distinct variables (*e.g.* sites) may be bond-topologically equivalent (*i.e.* independent of physical metrics), the opposite is not true: variables that are bond-topologically distinct cannot be crystallographically equivalent. For example, the introduction of one or more symmetry-breaking elements acting on sites that are bond-topologically equivalent will inevitably break their crystallographic equivalence, while retaining bond-topological equivalence (Fig. 6 ▸). This concept is an important demonstration of the hierarchy between bond-topological and crystallographic equivalence in crystal structures. As we show next, the distinction between bond-topological and crystallographic equivalence defines two classes of causal mechanisms underlying bond-length variation in inorganic solids.

*Quantifying bond-length variation as a result of bond-topological versus crystallographic effects*. Mechanisms underlying bond-length variation rarely occur in isolation. As a result, it is often difficult to pin-point the source(s) of bond-length variation in a coordination polyhedron, and the relative extent to which they operate. As we show below, calculation of the *a priori* bond valences of a crystal structure is a useful approach for resolving and quantifying sources of bond-length variation in extended solids, as it allows separation of the causal mechanisms underlying bond-length variation into those that are bond-topological and crystallographic in nature.

We may quantitatively assess the amount of bond-length variation caused by bond-topological asymmetry, Δ_topol_, as the mean (absolute) weighted deviation between the bond valences of a given polyhedron and those of its regular variant with equal bond lengths, *i.e.* its Pauling bond strength:
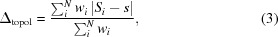
where *S*_*i*_ is the *a priori* bond valence, *s* is the Pauling bond strength and *w*_*i*_ is the multiplicity of the bond in the coordination polyhedron, and where the sum is taken over the *N* crystallographically distinct bonds in the polyhedron.

Conversely, we may quantify bond-valence deviations of crystallographic origin as
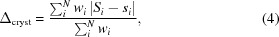
where the difference is between the *a priori* bond valences *S*_*i*_ and the observed bond valences *s*_*i*_. The quantity represented by the Δ_cryst_ index may be interpreted as the mean distortion caused by those effects that are not of bond-topological origin, *i.e.* whose bond-length variations are not captured/predicted via *a priori* bond valences. Both Δ_topol_ and Δ_cryst_ can be considered significant when >0.05 v.u., large when >0.10 v.u. and very large when >0.20 v.u.

The Δ_topol_ index quantifies bond-length variation caused by (1) non-local bond-topological asymmetry and (2) multiple-bond formation; Δ_cryst_ quantifies bond-length variation caused by (3) electronic effects (inclusive of effects such as lone-pair stereoactivity and magnetism, but with an inherent focus on coupled electronic vibrational degeneracy in this work) and (4) crystal-structure effects. Our choice of splitting the bond-topological mechanisms (1) and (2) is arbitrary, and follows historical practices of treating multiple-bond formation as an independent mechanism rooted in molecular-orbital theory (see Section 4.2). However, it is impossible to clearly resolve the contributions of (1) and (2) to the Δ_topol_ index, because the arrangement of *a priori* bond valences is disconnected from the underlying physical processes leading to the crystal structure at hand. In accordance with the hierarchy between bond-topological and crystallographic factors (discussed above), multiple-bond formation is primarily driven by the bond-topological requirements of the crystal structure, the consideration of which precedes the bond-valence stability range of ions. In practice, both the requirements of the crystal structure and those of the ions must coincide for the observation of (bond-topologically driven) multiple bonds, otherwise the structure will simply not occur, *i.e.* the ions will crystallize in a different structure type (or types). While bond topology primarily dictates the observation of multiple bonds in solids, there are cases where multiple-bond formation clearly results from crystallographic mechanisms, *e.g.* the pseudo Jahn–Teller effect. In those cases, bond-length variation escapes prediction via *a priori* bond valences, and instead is amalgamated into the Δ_cryst_ index under a different causal mechanism. As such, it is impossible to generalize the origins of multiple-bond formation in solids, but our method allows one to resolve whether this phenomenon results from a bond-topological or crystallographic mechanism on a structure-by-structure basis.

**Figure 7:**
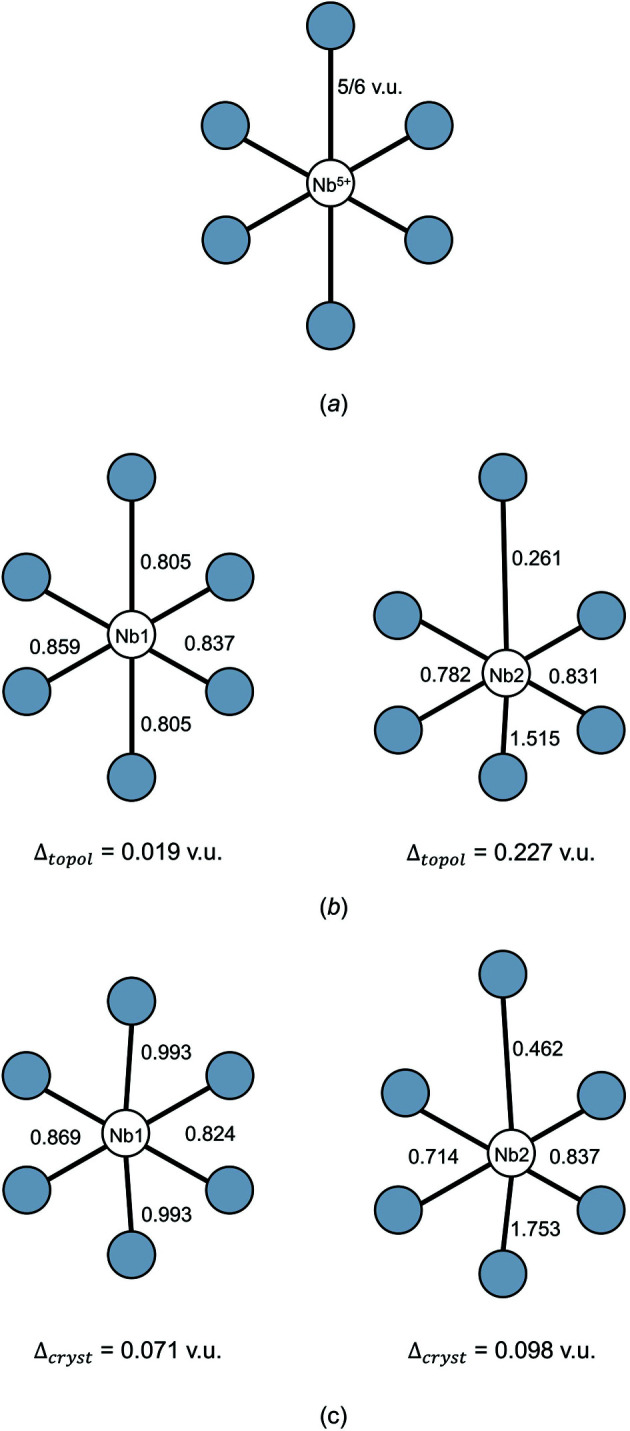
The local coordination of Nb1 and Nb2 in CaNb^5+^_2_(P_4_O_13_)(P_2_O_7_)O. (*a*) In this structure, the two crystallographically distinct Nb^5+^O_6_ octahedra do not have equal bonds 5/6 v.u. in strength. Instead, (*b*) non-local bond-topological asymmetry imposes bond-valence (and thus bond-length) variability within the polyhedra, which (*c*) serve as starting configurations for crystallographic effects to lead to the observed local geometries. The shaded spheres represent O^2−^.

The effect of bond-topological and crystallographic mechanisms on bond-length variation is well illustrated by CaNb^5+^_2_(P_4_O_13_)(P_2_O_7_)O (ICSD 62577; Averbuch-Pouchot, 1987 ▸), with two crystallographically distinct sites fully occupied by monomeric Nb^5+^ ions in octahedral coordination to O^2−^. For Nb1, all bonds are to non-bridging O^2−^ ions from P_2_O_7_ dimers; for Nb2, five bonds are to non-bridging O^2−^ ions of P_2_O_7_ dimers, and O13 has only one bond to Ca^2+^. The *a priori* (observed) bond valences are as follows (Table 6 ▸; Fig. 7 ▸): 2 × 0.805 (0.993), 2 × 0.837 (0.824) and 2 × 0.859 (0.869) v.u. for Nb1, with Δ_topol_ = 0.019 and Δ_cryst_ = 0.071 v.u., and 2 × 0.782 (0.714), 2 × 0.831 (0.837), 0.261 (0.462) and 1.515 v.u. (1.753) for Nb2, with Δ_topol_ = 0.227 and Δ_cryst_ = 0.098 v.u. With regard to crystallographic effects, the strongest and weakest bonds involving Nb2 (0.261 and 1.515 v.u., for bond lengths 2.406 and 1.756 Å) are in the *trans* configuration; the discrepancy between the *a priori* and observed bond valences for these two bonds is caused by the off-centring of Nb2 toward O13 via the pseudo Jahn–Teller effect (more on this below). With regard to bond-topological effects, we can see from the *a priori* bond valences and the Δ_topol_ index that the formation of a highly distorted octahedron on the one hand (Nb2), and of a regular octahedron on the other, for two monomers of the same cation in the same structure, simply results from non-local bond-topological asymmetry. Thus, we conclude from the Δ_topol_ and Δ_cryst_ values calculated for CaNb_2_(P_4_O_13_)(P_2_O_7_)O that (i) the main driver of bond-length variation is the pseudo Jahn–Teller effect (PJTE) for Nb1 and non-local bond-topological asymmetry for Nb2, and (ii) the magnitude of the PJTE is similar in both octahedra.

The insight provided by the *a priori* bond valences and the Δ_topol_ and Δ_cryst_ indices should be useful to experimentalists when refining, proposing and describing new crystal structures; calculating these values should become routine practice where possible, in the same way that the calculation of observed bond-valence sums is routine practice today. These analyses should further be useful for identifying the structural and electronic underpinnings of functional properties linked to asymmetric coordination units. At present, no model rigorously defines the extent to which functional properties may be optimized via compositional and/or structural modifications. As we discuss later in this article, the calculation of the Δ_topol_ and Δ_cryst_ indices resolves and quantifies the extent to which bond-topological and/or crystallographic phenomena are responsible for a given functional property in a crystal structure. Such knowledge allows informed optimization of the proper causal mechanisms giving rise to these properties, and sets expectation limits with regard to the optimizable extent of these functional properties.

To resolve the main cause(s) of bond-length variation underlying the numerous multi-modal bond-length distributions identified in this work (Fig. 1 ▸), we calculated values of Δ_topol_ and Δ_cryst_ for 268 transition metal coordination polyhedra representing some of the largest bond-length variations observed for factors (1)–(4) above. These data cover 85 transition metal ion configurations taken from 140 specific crystal structures which we solved for the *a priori* bond valences (Table S1). Values of Δ_topol_ and Δ_cryst_ are given in Table S2, and certain values are included in the following sections to aid our analysis. From a representative cross section of factors (1)–(4) in the set of selected crystal structures, we find 〈Δ_topol_〉 = 0.102 v.u. and 〈Δ_cryst_〉 = 0.113 v.u. for the 268 transition metal coordination polyhedra, giving a glimpse into the extent and magnitude by which bond-length variations are affected by non-local bond-topological asymmetry. These values will be discussed in greater detail below (Section 6.1). Next, we discuss two special cases where non-local bond-topological asymmetry has a particularly marked effect on bond-length variations for strongly bonded units.

#### 4.1.3. Special case (i): Polymerization of strongly bonded units where 〈BV〉_cat_ is greater than 〈BV〉_br an_   

Bond-length variation driven by bond-topological asymmetry can be plainly illustrated via localized bonding interactions in strongly bonded units, for which bond-length variations result from the inter-connectedness of the bond-valence constraints of cations and anions with regard to the valence-sum rule [equation (2)]. For certain conditions, competition between the bond-valence constraints of cations and their bonded anions necessarily requires an uneven distribution of bond valences in cation and/or anion coordination polyhedra. These conditions are typical of oxide and oxysalt structures, in which the combination of high cation oxidation states and low coordination numbers results in high mean bond valences and thus strongly bonded oxyanions. Where the mean bond valence of a cation configuration (〈BV〉_cat_) exceeds that of the mean bond valence of the bridging anion (〈BV〉_br an_), polymerization of the oxyanion requires significant weakening (*i.e.* lengthening) of the bridging *M*—O—*M* bonds for the valence-sum rule to hold at the bridging O^2−^ ion. For example, ^[4]^Cr^6+^ readily polymerizes into corner-sharing dimers (*e.g.* Ag^+^_2_Cr^6+^_2_O_7_; ICSD 2433; Durif & Averbuch-Pouchot, 1978 ▸) where the bridging ^[2]^O^2−^ ion forms two bonds 1 v.u. in strength (bond-valence sum 2 v.u.). Ideally, the Cr^6+^O_4_ tetrahedron would have four bonds 1.5 v.u. in strength, but this is prohibited by the bond-valence constraints of the bridging ^[2]^O^2−^. As a result, Cr^6+^O_4_ dimers form three bonds 5/3 v.u. (1.608 Å) in strength and one bridging bond 1 v.u. (1.799 Å) in strength, thus resulting in a bimodal distribution of bond lengths for corner-sharing Cr^6+^O_4_ dimers. While this mechanism may seem to be different from that of non-local bond-topological asymmetry (see above), the difference is a matter of interpretation; bridging and non-bridging bonds are necessarily bond-topologically inequivalent. As such, we sometimes use the terms ‘bond-topological asymmetry’ and ‘bond-topological effects’ interchangeably in this article, although we tend to use the latter for more general discussion.

**Figure 8:**
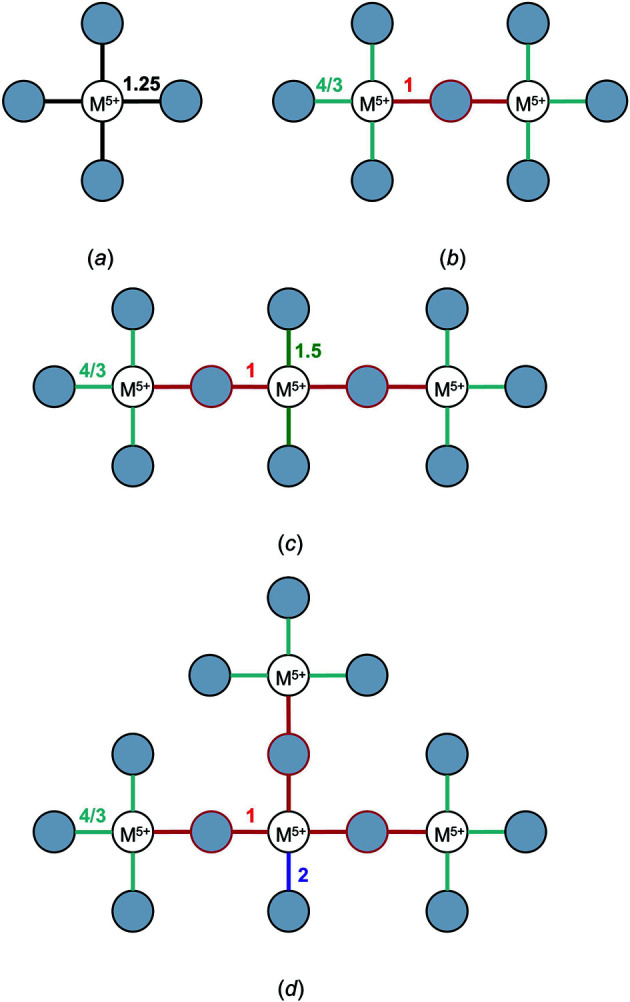
Bond-valence patterns arising from various corner-sharing tetrahedra for the +5 oxidation state for (*a*) monomers, (*b*) dimers, (*c*) linear oligomers and chains, and (*d*) branched polymers. Shaded spheres represent O^2−^.

One may list all possible bond-valence patterns arising from various polyhedra, polyhedral connectivity and degree of polymerization to realize the richness of bond-valence constraints in the solid state and the scale of bond-length variation that arises solely from bond-topological constraints. For example, we enumerate possible bond-valence patterns arising from various corner-sharing tetrahedra for +5 and +6 oxidation states in Figs. 8 ▸ and 9 ▸, respectively. For a central cation with oxidation state +5, isolated tetrahedra ideally form bonds of 1.25 v.u. For a dimer, the bridging *M*—O—*M* bond is 1 v.u. and the other three bonds therefore adjust to 4/3 v.u. For a trimer, the central tetrahedron forms two bonds of 1 v.u. (both bridging) and the other two bonds adjust to 1.5 v.u. For a branched tetramer (a linear/cyclic tetramer does not lead to new bond-valence constraints), the central tetrahedron makes three bonds of 1 v.u. and its fourth bond is 2 v.u., *i.e.* the maximum bond valence achievable by O^2−^. For a +6 oxidation state, monomers ideally form four bonds 1.5 v.u. in strength, while dimers make bonds 1 v.u. + 3 × 5/3 v.u. in strength. For trimers, the doubly bridged central tetrahedron forms two bonds of 2 v.u. and a branched tetramer is therefore impossible to achieve. These bond-valence patterns increase in complexity when considering (i) polymerization with different (but similarly strongly bonded) ions and ion configurations, (ii) polymerization via multiple bridging anions, *i.e.* edge- and face-sharing, including the formation of complex oxygen-sharing clusters typical of ^[5–6]^V^5+^, ^[6]^W^6+^, ^[6]^Mo^6+^, and sometimes ^[6]^Ti^4+^, ^[6]^Nb^5+^ and ^[6]^Ta^5+^, and (iii) additional (non-bridging) bonds made by the bridging anion(s) *etc*. In other words, these bond-valence constraints will experience further variability based on the exact bond topology of the crystal structure. Fortunately, one does not have to keep track of all such variables, unless one wishes to rationalize the exact pattern of *a priori* bond valences, whose simple calculation is otherwise sufficient for all intents and purposes. Thus for oxides, polymerization of strongly bonded units invariably leads to bond-length variation when OS/CN > 1 for ^[2]^O^2−^_br_, > 2/3 for ^[3]^O^2−^_br_ *etc*. This condition is necessary but not sufficient, as some combinations of charge and coordination number cannot result in polymerization of the strongly bonded unit, *e.g.* for ^[4]^Os^8+^.

We illustrate this concept with Li_3_Nb^5+^O_4_ (ICSD 75264; Ukei *et al.*, 1994 ▸) which consists of edge-sharing [Nb^5+^_4_O_16_]^12−^ clusters interconnected via Li^+^. The mean bond valence to the bridging ion (O1; Table 7 ▸) is roughly 2/3 v.u. when ignoring the weak bonds made to Li^+^. Thus, Nb^5+^ adjusts from its mean bond valence of 0.833 v.u. to make three weaker bonds of 0.708 v.u. to the bridging O1, with the other three bonds adjusting to 0.958 v.u. (with corresponding *a priori* bond lengths of 2.037 and 1.925 Å, respectively). This split is further accentuated by displacement of the Nb^5+^ ion toward the face made by the O1 ions as a result of a pseudo Jahn–Teller effect, resulting in an effective split in the bond lengths of 1.858–2.130 Å (Δ_topol_ = 0.125, Δ_cryst_ = 0.174 v.u.).

#### 4.1.4. Special case (ii): Polymerization of strongly bonded units where 〈BV〉_cat_ is smaller than 〈BV〉_br an_   

In the previous section, we showed that bond-length variation driven by bond-topological asymmetry is inherent in cases where 〈BV〉_cat_ is greater than 〈BV〉_br an_. In those cases, the bond-valence constraints on bridging anions induce an increase in the bond valences at the non-bridging bonds, leading to considerable bond-length variation. The inverse situation, where 〈BV〉_cat_ is smaller than 〈BV〉_br an_, also results in a clear-cut bond-length variation driven by bond-topological asymmetry, this time in the form of a decrease in the bond valences at the non-bridging bonds. This phenomenon may lead to bond-length variability of similar magnitude; although the bond-valence variability is typically less, the cations involved are of lower Lewis acidity (defined as the ratio of oxidation state and mean observed coordination number; see Gagné & Hawthorne, 2017*a* ▸), resulting in larger bond-length variations for a given bond-valence variation.

A simple illustration of this effect is seen in PW^5+^O_5_ (ICSD 203048; Wang *et al.*, 1989 ▸), the structure of which consists of chains of corner-sharing W^5+^O_6_ octahedra. The *a priori* bond valences for W^5+^ are 2 × 1 v.u. for the bridging bonds and 4 × 0.75 v.u. for the non-bridging bonds (Table 8 ▸), compared with six bonds of 0.833 v.u. (1.949 Å) for holosymmetric coordination. These compare with the experimental values of 0.949 and 1.027 v.u. for the bridging bonds, and 0.823, 0.811, 0.809 and 0.753 for the non-bridging bonds, showing how ^[6]^W^5+^ adjusts to the anion bond-valence requirements by making two stronger bridging bonds and four weaker non-bridging bonds to O^2−^ (Δ_topol_ = 0.111, Δ_cryst_ = 0.046 v.u.). The resulting dispersion of bond lengths for W^5+^ is 1.833–2.005 Å.

To show this effect for a cation of lower Lewis acidity, we calculate the *a priori* bond valences for Fe^3+^ in aegirine, NaFe^3+^Si_2_O_6_ (ICSD 157733; Nestola *et al.*, 2007 ▸). In this structure, Fe^3+^ forms chains of edge-sharing Fe^3+^O_6_ octahedra sharing via four bridging O1 ions (which further bond to Si^4+^ and Na^+^), while the two non-bridging O2 sites bond only to Si^4+^ and Na^+^ (Table 9 ▸). To accommodate the different bond-valence requirements of O^2−^, the *a priori* bond valences for the bonds to O2 increase to 2 × 0.6 v.u., from 6 × 0.5 v.u. (2.016 Å) in holosymmetric coordination, whereas bonds to O1 decrease to 4 × 0.45 v.u. The predicted dispersion of bond lengths is 1.950–2.053 Å (observed 1.939–2.113 Å), with Δ_topol_ = 0.067 and Δ_cryst_ = 0.038 v.u.

### 4.2. Mechanism (2): Multiple-bond formation   

Many molecular features of coordination complexes are preserved as they are incorporated into crystal structures, and their electronic properties are often more important than the ensuing steric constraints of the crystal structure (Bersuker, 2010 ▸). A simplifying assumption commonly made in studying the geometry of transition metal complexes in crystals is to overlook translational symmetry, treating these complexes as ‘molecules in solids’ (Burdett, 1981 ▸, 1984 ▸; Roesky *et al.*, 2003 ▸), which allows a more chemically intuitive treatment of chemical bonding of the crystal’s molecular fragments via ligand-field theory.

**Figure 10:**
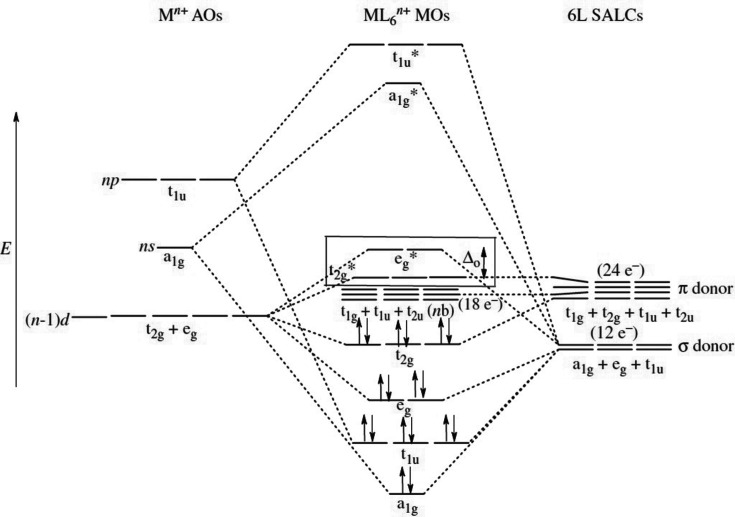
A molecular orbital (MO) diagram for [*ML*_6_]^*n*+^ compounds where the ligands (O^2−^) can act as both σ and π donors. Transition metal *d* electrons fill levels starting from *t*_2g_* in a way that progressively negates favourable π interaction, thus favouring complexes with few to no *d* electrons. Reproduced with permission from Pfennig (2015 ▸; Fig. 160.26, p. 531), copyright (2015) Wiley.

Fig. 10 ▸ shows the standard molecular-orbital (MO) bonding scheme for a transition metal and its oxygen ligands in octahedral coordination (reproduced with permission from Pfennig, 2015 ▸). Symmetry-adapted linear combinations (SALCs) of atomic orbitals for the O^2−^ ligands show that π donor orbitals *t*_1*g*_, *t*_2*g*_, *t*_1*u*_ and *t*_2*u*_ are sufficiently close in energy to interact with the atomic orbitals (AOs) of the transition metal. However, only the *t*_2*g*_ orbital has the appropriate symmetry and spatial overlap to mix with that of the transition metal, and the other three orbitals remain non-bonded. The MO levels are filled with ligand electrons up to *t*_2*g*_ (18 electrons, not counting an additional 18 non-bonded electrons), at which point transition metal *d* electrons begin to fill levels starting from *t*_2g_* in a way that progressively negates the favourable π interaction, *i.e.* a *t*_2*g*_ state of lower energy in comparison with non-bonding. For this reason, the most favourable π-bonding interactions for octahedrally coordinated transition metal oxyanions (called oxo complexes in coordination chemistry) involve transition metals with few to no *d* electrons; this is well supported by our data, as we will see below. Following favourable π-type interaction, the complex is described as forming ‘multiple bonds’ to one or more of its ligands (all ligands forming primary bonds to the transition metal via their σ-donor orbitals; Fig. 10 ▸). These are sometimes described as ‘yl’ complexes, *e.g.* vanadyl (Schindler *et al.*, 2000 ▸) and uranyl (Lussier *et al.*, 2016 ▸). The additional bonding component shortens the bonded distance to the ligand(s) involved, thus resulting in bond-length variation. This phenomenon usually manifests itself in our data in the form of a multi-modal distribution of bond lengths, typically with a mode at unusually short bond lengths.

We note that the above treatment is best-suited to coval­ently bonded molecules. This description therefore holds to the extent for which crystals can be described as (strongly bonded) molecular fragments. Additional bonding schemes, *e.g.* those that arise in extended solids as a result of substantial electron delocalization, and ionic interactions, complicate the bonding picture. An important result of their consideration is the observation of non-integer bond orders in crystals (first described by Pauling in metals; Pauling, 1947 ▸), which is well captured by the bond-valence model via the observation of non-integer bond valences, which may be interpreted as resulting in part from a continuum of orbital spatial overlap along the bond axis.

As discussed in Section 4.1.2, multiple-bond formation is inherently modelled in the bond-topological component Δ_topol_. The continuous (non-integer) nature of bond valences, combined with the overlapping effects of non-local bond-topological asymmetry and multiple-bond formation, renders a clear-cut divide of whether or not multiple-bond formation is the main reason for bond-length variation difficult to achieve. Our analysis suggests that multiple-bond formation may be the main reason underlying bond-length variation where (i) Δ_topol_ > Δ_cryst_, (ii) BV_max_ > 1.25 MBV (mean bond valence) > 1.75 v.u. and (iii) the formation of π bonds is not an inherent result of the polymerization of strongly bonded units, in which case we consider non-local bond-topological asymmetry as the main cause of bond-length variation. For (ii), the condition BV_max_ > 1.25 MBV is implemented so as not to mistakenly classify those ion configurations that form strong π bonds by necessity (*e.g.* ^[4]^Re^7+^) as due to multiple-bond formation. For example, we calculated the *a priori* bond valences for seven ^[6]^V^4+^ polyhedra in six crystal structures to find 〈Δ_topol_〉 = 0.207 and 〈Δ_cryst_〉 = 0.145 v.u. For this ion configuration, BV_max_ is frequently >1.75 v.u., higher than 1.25 × 2/3 v.u. [V^4+^O_6_]^8−^ units are often observed as monomers. As the formation of strong π bonds is not an inherent product of polymerization, we conclude that the formation of π bonds is the main factor underlying bond-length variation for this ion configuration when bonded to O^2−^. Following similar logic, we find bond-length variation to result from bond-topological asymmetry for [Cr^6+^O_4_]^2−^, under the special case ‘Polymerization of strongly bonded units where 〈BV〉_cat_ is greater than 〈BV〉_br an_’, as the formation of the strong π bonds is indeed caused by polymerization of the [Cr^6+^O_4_]^2−^ unit. When Δ_cryst_ > Δ_topol_, multiple-bond formation is always a result of the pseudo Jahn–Teller effect (below) for *d* ^0^ transition metals.

### 4.3. Mechanism (3): Electronic effects   

In recent decades, significant developments in electronic structure theory have reduced the problem of molecular engineering and materials design to increasingly quantitative calculations of the electronic structure of both known and hypothetical compounds. It is expected that electronic effects represent the dominant force underlying bond-length variation *for a given crystal structure*, *i.e.* for which the effect of non-local bond-topological asymmetry on bond lengths is disregarded as a quantifiable and predictable constant. However, the extent to which electronic effects affect bond lengths in solids has yet to be quantified on a large scale, hindering the rapid identification of these phenomena in crystal structures and clouding the extent to which bond-length variations may be expected from these effects within the constraints of physically realistic crystal structures (with direct applications in materials design and the verification of computational results).

The two most common types of electronic effects present in inorganic solids are (i) lone-pair stereoactivity and (ii) coupled electronic vibrational degeneracy. Lone-pair stereoactivity results from strong interaction between cation *s* and anion *p* orbitals, leading to a high-energy antibonding state which, via structure distortion, may interact with empty cation *p* orbitals to form a localized electronic state where the lone pair resides (Walsh *et al.*, 2011 ▸). The extent to which this phenomenon leads to bond-length variation in oxide and oxysalt structures was discussed for *ns* ^2^ *np* ^0^ *p*-block cations earlier in this series (Gagné & Hawthorne, 2018*a* ▸,*b* ▸). Because transition metals are not subject to lone-pair stereoactivity, our discussion of electronic effects is limited to (ii) coupled electronic vibrational degeneracy, below. Other common electronic phenomena which may result in bond-length variation (*e.g.* inductive effects) are typically only relevant to hetereo-ligand coordination centres and are not considered here.

#### 4.3.1. Coupled electronic vibrational degeneracy: the Jahn–Teller effect   

The Jahn–Teller effect is a mechanism of symmetry breaking in molecules and solids, and results from strong electron–vibrational (vibronic) and electron–phonon interactions in molecules and crystals, respectively (Bersuker, 2006 ▸). The phenomenon was first described from group-theoretical arguments by Jahn and Teller, who showed that nonlinear molecules cannot be stable if they have energetically degenerate electronic states, resulting in their spontaneous distortion to a lower-symmetry configuration with split (near-degenerate) states (Jahn *et al.*, 1937 ▸). An energetically favourable occupancy of the non-degenerate states, which depends on the number of electrons available to populate them, characterizes the Jahn–Teller effect (herein abbreviated as JTE). The recognition of similar mechanisms for near-degenerate orbital electronic states later resulted in a significantly broader definition of the JTE (Öpik & Pryce, 1957 ▸; Longuet-Higgins & Salem, 1959 ▸; Bader, 1960 ▸), inclusive of pseudo-degenerate states where the energy gap between the mixing states is sufficiently small in comparison with other vibronic parameters of the system (Bersuker, 2006 ▸). In this work, we avoid the ‘first-’ and ‘second-’ order terminology commonly used to describe the JTE for degenerate and near-degenerate energy states, respectively. This terminology originates from a perturbation-theoretical treatment of the JTE (Bader, 1960 ▸; Pearson, 1969 ▸), which, despite contributing significantly to the understanding of many chemical questions, has problems (Bersuker, 2013 ▸). We use the term ‘JTE’ as inclusive of both cases of degeneracy, and ‘pseudo JTE’ (PJTE) for near-degenerate electronic states. One mechanism is not exclusive of the other, as the PJTE may still be an important source of instability in the presence of electronic degeneracy (Bersuker, 2013 ▸).

The JTE has been proposed to be the only source of instability and distortion for polyatomic systems in near-degenerate states (Bersuker, 2006 ▸, 2013 ▸) and, more generally, to be the only source of spontaneous symmetry breaking in matter in all its forms (Bersuker, 2016 ▸). In the light of a previous section (Section 4.1), we find this statement to be incorrect: distortion away from the configuration of highest symmetry may arise from asymmetry in the bond network, a phenomenon that, as we will see below (Section 5.1), occurs much more frequently than coupled electronic vibrational degeneracy, with no *a priori* limitation with regard to ion identity.

**Figure 11:**
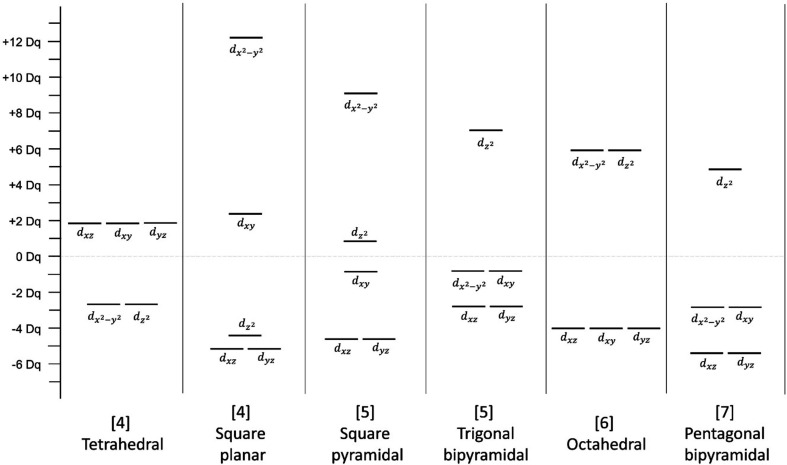
Crystal-field splitting for the five *d* orbitals for some of the most frequently observed coordinations in this work.

*Degenerate electronic states*. The classic interpretation of the JTE deals with electron occupancy of degenerate electronic states. It is traditionally described in the context of octahedral and tetrahedral coordination, for they are frequently observed coordinations that are geometrically apt to distortion as a result of the shape and orientation of *d* orbitals. Energy changes for the five degenerate *d* orbitals of the transition metals (*d_xy_*, *d_xz_*, *d_yz_*, 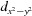 and 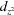) upon their surrounding by an array of ligands (here, O^2−^) is most succinctly described via crystal-field theory (CFT). Fig. 11 ▸ shows the crystal-field splitting of energy levels for some of the most frequently observed coordinations of this work (*Dq* values from Bersuker, 2010 ▸).

In the classic description of the JTE, degenerate *d* electronic states split into triply degenerate *t*_2*g*_ (*d_xy_*, *d_xz_*, *d_yz_*) and doubly degenerate *e*_*g*_ (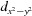 and 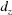) energy levels for an octahedral coordination of ligands; for this coordination, the *e*_*g*_ orbitals are higher in energy as they point directly at the ligands, resulting in electrostatic repulsion with the bonding electrons. For a tetrahedral coordination, the *d* electronic states split into doubly degenerate *e* (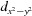 and 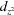) and triply degenerate *t*_2_ (*d_xy_*, *d_xz_*, *d_yz_*) electronic states, with the *t*_2_ orbital higher in energy (Fig. 11 ▸). The occurrence of a JT distortion depends on the occupancy of these electronic states, which in turn depends on the number of *d* electrons available. Where the degeneracy occurs in the orbital set of higher energy, distortion resulting from the JTE is ‘strong’ (with relatively large bond-length variation), and ‘weak’ otherwise. For an octahedral crystal field, degeneracy is strong for electron configurations HS *d* ^4^, LS *d* ^7^ and *d* ^9^; those prone to weak JTE are *d* ^1^, *d* ^2^, LS *d* ^4^, LS *d* ^5^, HS *d* ^6^ and HS *d* ^7^ (HS and LS denote high spin and low spin, respectively). For a tetrahedral field, the largest distortions (*i.e.* bond-length variations) are expected for configurations HS *d* ^3^, HS *d* ^4^, *d* ^8^ and *d* ^9^, and so on and so forth for every coordination geometry. For example, the strong JTE is observed for ^[6]^Mn^3+^ (*d* ^4^) in Gd^3+^Mn^3+^O_3_ (ICSD 95493; Mori *et al.*, 2002 ▸), with *a priori* (observed) bond valences 4 × 0.471 (2 × 0.194 and 2 × 0.700) and 2 × 0.559 (0.612) v.u. for Mn^3+^, and Δ_topol_ = 0.039 and Δ_cryst_ = 0.186 v.u. As a side note, the wide discrepancy seen between the *a priori* and observed bond valences in this example emphasizes the inability of *a priori* bond valences to model the Jahn–Teller distortion (more on this below).

After examining our dataset in detail for the strong and weak JTE (JTEs and JTEw, respectively) in various crystal fields, and comparing the magnitude of the effect with that of distortion of bond-topological origin via the Δ_topol_ and Δ_cryst_ indices, we identify three ion configurations from Table 4 ▸ whose main underlying cause of bond-length variation is the strong JTE (^[6]^Mn^3+^, ^[6]^Cr^2+^ and ^[6]^Cu^2+^) and none whose main cause is the weak JTE. We observe these effects as minor contributors to bond-length variation for ^[6]^Co^2+^ (JTEs) and ^[6]^V^3+^ and ^[6]^Mo^5+^ (JTEw). These will be discussed in Section 5.3 below.

**Figure 12:**
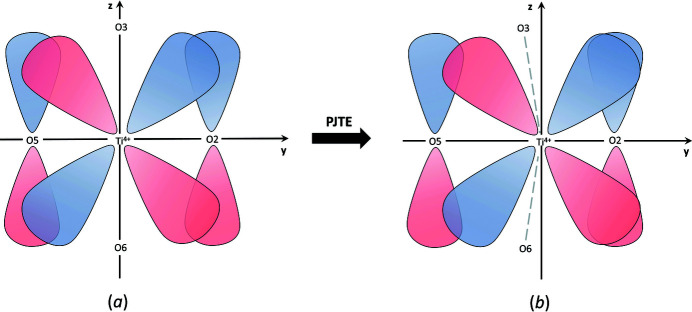
Visual representation of the pseudo Jahn–Teller effect for a TiO_6_ octahedron. O1 and O4 are directly above and below Ti^4+^ in (*a*); blue lobes are (+) and red (−). In the high-symmetry configuration (*a*), the overlap integral between the HOMO |*t*_1*u*_ *z*〉 (from O^2−^) and LUMO |3*d_yz_*〉 (from Ti^4+^) is null. However, displacement of Ti^4+^ along the *y* axis (*b*) results in both an increase in positive overlap (+/+ and −/−) and a decrease in negative overlap (+/−), thus resulting in spontaneous distortion.

*Near-degenerate electronic states*. The pseudo Jahn–Teller effect (PJTE) results from the vibronic mixing of two (or more) near-degenerate electronic states under nuclear displacement (Bersuker, 2006 ▸). As such, the PJTE is not encumbered by *a priori* limitations as is the case for the classic interpretation of the JTE. However, the energy gap between the interacting states, usually (but not always) the highest occupied molecular orbital (HOMO) and the lowest unoccupied molecular orbital (LUMO), must be small, and there must be a distortion mode that has the same symmetry as the HOMO to LUMO transition (the energy gap is a function of ligand identity) (Kunz & Brown, 1995 ▸). Fig. 12 ▸ gives a simple visual representation of the PJTE for a TiO_6_ octahedron [adapted from Bersuker (2006 ▸)]. We plot the HOMO |*t*_1*u*_ *z*〉 (from O^2−^) and the LUMO |3*d_yz_*〉 (from Ti^4+^) of the system. It can then be seen that favourable vibronic mixing results in a positive overlap integral upon displacement of the Ti^4+^ ion along the *y* axis (*i.e.* ‘off-centring’), resulting in an energetically favoured lower-symmetry configuration. For example, Mo^6+^ displaces toward a corner in Cs[Mo^6+^_2_O_3_(PO_4_)_2_] (ICSD 79517; Hoareau *et al.*, 1995 ▸), with *a priori* (observed) bond valences 0.724 (0.702), 0.731 (0.541), 2 × 0.768 (0.640 and 0.730), 0.772 (0.678) and 1.237 (1.988) v.u., and Δ_topol_ = 0.134 and Δ_cryst_ = 0.204 v.u. In this example, Mo^6+^ displaces toward O1 along the O6—Mo1—O1 axis, resulting in strong/weak bonds of 1.988/0.541 v.u.

The PJTE is widely observed in transition metals with a *d* ^0^ electronic configuration, although it should be noted that its occurrence is not limited to this configuration (Bersuker, 2013 ▸; Reinen & Atanasov, 1991 ▸; Reinen & Friebel, 1984 ▸; examples below). In turn, the *d* ^0^ electronic configuration is relatively well studied *vis à vis* symmetry-breaking bond-length variation due to the wide-ranging technologically relevant properties of compounds containing *d* ^0^ transition metals in asymmetric coordination environments (see Section 2). For example, the frequently encountered bistable behaviour of crystal structures with *d* ^0^ transition metals is exploited in the design of atomic switches (Szymanski *et al.*, 2019 ▸) and artificial neurons (Yang *et al.*, 2013 ▸), and could foreseeably be used to control the sorption characteristics of catalysts to move beyond the Sabatier principle (Kakekhani & Ismail-Beigi, 2015 ▸). In addition to being inversely proportional to the HOMO–LUMO gap, the magnitude of polyhedral distortion follows electronegativity (Halasyamani, 2004 ▸), and the commonly observed *d* ^0^ ions have been quantified as strong (Mo^6+^ and V^5+^), moderate (W^6+^, Ti^4+^, Nb^5+^ and Ta^5+^) and weak (Zr^4+^ and Hf^4+^) distorters (Ok *et al.*, 2006 ▸). It has further been suggested that bond topology influences the occurrence and magnitude of the PJTE in a primary (Kunz & Brown, 1995 ▸) and secondary (Welk *et al.*, 2002 ▸) capacity, whereby the PJTE either results from, or is affected by, the arrangement of *a priori* bond valences in structures with *d* ^0^ transition metals. We investigate this issue via calculation of *a priori* bond valences for over 130 *d* ^0^ transition metal oxide polyhedra, below.

### 4.4. Mechanism (4): Crystal-structure effects   

The constraints of long-range order and periodicity have important implications with regard to bond distances. Variations in external conditions (*e.g.* temperature, pressure, applied field) may further result in a variability in bond lengths up to the point of phase transition [beyond which bond-length variations are accounted for via mechanism (1), non-local bond-topological asymmetry]. Here, we group these effects under the designation of ‘crystal-structure effects’. These effects do not lead to multi-modality of the bond-length distributions, and they cause bond-length variations of significantly lower magnitude than those of mechanisms (1)–(3). As such, quantifying bond-length variations due to crystal-structure effects can only be done in the absence of other crystallographic mechanisms of bond-length variation for a given polyhedron (*e.g.* lone-pair stereoactivity, coupled electronic vibrational degeneracy) which otherwise overwhelm the Δ_cryst_ effect. Moreover, bond-length variation caused by these mechanisms is expected to fall semi-randomly about the mean bond length in a way that does not significantly alter the shape of bond-length distributions.

#### 4.4.1. Structural strain   

Some polyhedra are inherently unable to adopt their configuration of highest symmetry as a result of the imperfect projection of their *a priori* bond lengths into three-dimensional space (Kunz & Brown, 1995 ▸; Bosi, 2014 ▸). This was recently demonstrated by Gagné and Hawthorne, who showed the inability of a crystal structure to attain its *a priori* bond lengths within the constraints of space-group symmetry and periodicity by showing excellent agreement between observed and *a priori* bond lengths *within* a structure type, and the loss of this agreement *across* structure types (Gagné & Hawthorne, 2017*b* ▸). The mismatch between *a priori* and observed bond lengths may be used to quantify structural strain via distortion indices, *e.g.* the Global Instability Index (Salinas-Sanchez *et al.*, 1992 ▸) and the Bond Strain Index (Preiser *et al.*, 1999 ▸). Structural strain may be isotropic or anisotropic in nature; Fig. 4 ▸ provides evidence for the isotropic working of this phenomenon whereby nearly a dozen transition metal ion configurations are observed to cover a surprisingly wide range (∼0.1 Å) of mean bond lengths for Δ = 0 (*e.g.* ^[6]^Mn^2+^, ^[6]^Fe^3+^, ^[4,6]^Co^2+^, ^[4]^Cu^2+^).

#### 4.4.2. Next-nearest-neighbour interactions   

Sometimes called steric effects, factors such as metal–metal and anion–anion repulsion and nearby stereoactive lone-pair electrons have been shown to influence bond-length variations and polyhedral distortion (Halasyamani, 2004 ▸; Kunz & Brown, 1995 ▸), for example resulting in preferential displacement of *d* ^0^ transition metals under the PJTE. We observe only one ion configuration in Table 4 ▸ where next-nearest-neighbour interactions seem to be the underlying cause of polyhedral interaction (Ru^5+^–Ru^5+^ interactions for ^[6]^Ru^5+^), discussed in Section 5.5 below.

## 5. Part III: Determination of the causal mechanism(s) underlying bond-length variation for ion configurations with anomalous bond-length distributions   

In this section, we identify the principal and minor causal mechanism(s) underlying bond-length variation for the 52 ion configurations listed in Table 4 ▸. We further quantify the extent to which these causal mechanisms affect bond-length variation for those ion configurations. Our discussion is arranged into four subsections based on causal mechanism: (1) non-local bond-topological effects, (2) multiple-bond formation, (3) electronic effects (coupled electronic vibrational degeneracy) and (4) crystal-structure effects. The ion configurations of Table 4 ▸ are discussed in the subsection which corresponds to their principal cause of bond-length variation. This Section 5 (*i.e.* Part III) may be skipped on first reading, until the reader requires further detail on specific ion configurations.

Our discussion of ion configurations follows a consistent form throughout: we (i) calculate *a priori* bond valences and indices Δ_topol_ and Δ_cryst_ for polyhedra with anomalous bond-length dispersion; (ii) calculate 〈Δ_topol_〉 and 〈Δ_cryst_〉 values to determine whether the bond-length distribution is primarily irregular as a result of bond-topological or crystallographic effects; (iii) identify the main and minor causes of bond-length variation based on frequency of observation and magnitude; (iv) describe the effect of causal mechanisms on the shape and range of the bond-length distribution; and (v) compare main and minor causes of bond-length variation with similar ion configurations where pertinent, and/or other relevant information.

### 5.1. Mechanism (1): Ion configurations primarily distorted via non-local bond-topological effects   

^[6]^Sc^3+^ [Fig. 1 ▸(*a*)] has a subtly bimodal distribution of bond lengths. The main distribution peaks at 2.09 Å (0.50 v.u.) and the other maximum is at 2.12 Å (0.47 v.u.), and this occurs in conjunction with a hidden peak at 2.07 Å (0.53 v.u.). For example, Na_3_Sc_2_(PO_4_)_3_ (ICSD 65407; Collin *et al.*, 1986 ▸) has *a priori* (observed) bond valences 3 × 0.519 (0.555) and 3 × 0.481 (0.477) v.u., with Δ_topol_ = 0.019 and Δ_cryst_ = 0.020 v.u. The tail at shorter bond lengths is longer than expected; constituent data include those of LiSc(SiO_3_)_2_ (ICSD 200128; Hawthorne & Grundy, 1977 ▸), with *a priori* (observed) bond valences 4 × 0.45 (2 × 0.491 and 2 × 0.383) and 2 × 0.6 (0.607) v.u., with Δ_topol_ = 0.067 and Δ_cryst_ = 0.038 v.u. Thus, bond-length variations for this ion configuration mainly result from non-local bond-topological asymmetry, and to a lesser extent from the pseudo Jahn–Teller effect.

^[6]^V^3+^ [Fig. 1 ▸(*e*)] occurs as monomers, oligomers, chains, sheets and frameworks. Despite the proclivity of the [V^3+^O_6_]^9−^ unit for polymerization, its bond-length distribution is rather regular and does not cover an overly large range of bond lengths that is typical of other ions exhibiting this behaviour (0.399 Å, versus 0.826 Å for ^[6]^Ti^4+^, for example). There is, however, a subtle second maximum in Fig. 1 ▸(*e*) at 2.06 Å (0.44 v.u.). This maximum arises from structures in which [V^3+^O_6_]^9−^ polymerizes into oligomers, chains, sheets and frameworks, all of which result in weaker bonds 0.4–0.5 v.u. (versus 0.55–0.65 v.u. for the strongest bonds). This for example agrees with *a priori* (observed) bond valences calculated for distorted polyhedra of edge-sharing chains in LiV^3+^(Si_2_O_6_) (ICSD 59244; Satto *et al.*, 1997 ▸), 4 × 0.45 (0.429) and 2 × 0.6 (0.612) v.u., with Δ_topol_ = 0.067 and Δ_cryst_ = 0.018 v.u. Variations in bond lengths roughly result from bond-topological and crystallographic constraints in equal proportions for chains of edge-sharing octahedra in SrV^3+^_2_O(PO_4_)_2_ (ICSD 82685; Boudin *et al.*, 1996 ▸), with Δ_topol_ = 0.040 and Δ_cryst_ = 0.071 v.u., and Δ_topol_ = 0.072 and Δ_cryst_ = 0.052 v.u. for V1 and V2, respectively, showing that some variation in bond lengths may be due to the weak JTE (*d* ^2^ electronic configuration) for this ion configuration. Average values of Δ_topol_ and Δ_cryst_ for five polyhedra in four structures are 0.064 and 0.040 v.u., respectively.

^[4]^V^5+^ [Fig. 1 ▸(*h*)] has a somewhat symmetrical distribution of bond lengths when bonded to O^2−^. The [V^5+^O_4_]^3−^ unit polymerizes into various linear oligomers, chains and rings, with important implications regarding O_br_ as the mean bond valence for this ion configuration is >1 v.u. [special case (i) of non-local bond-topological asymmetry]. Symmetrization of the ^[4]^V^5+^—O^2−^ bond-length distribution is well illustrated from the bond-length pattern that results from V_4_O_12_ and V_6_O_18_ rings in K_3_CaV_5_O_15_ (ICSD 401203; Martin & Müller-Buschbaum, 1995 ▸): the two bridging bond valences of ∼1 v.u. result in observed bond lengths of 1.76–1.81 Å, while the two non-bridging O atoms adjust to ∼1.5 v.u. for observed bond lengths of 1.61–1.65 Å. These bond lengths fall on each side of the predicted maximum for monomer units, 1.71 Å (5/4 v.u.). We calculated the *a priori* (observed) bond valences for 13 coordination polyhedra in six structures; the mean values of Δ_topol_ and Δ_cryst_ of 0.122 and 0.099 v.u. indicate that, despite a high value for Δ_cryst_ (largely attributed to the PJTE), non-local bond-topological asymmetry is the main reason underlying bond-length variation for this ion configuration. We do not find coordination polyhedra in our data where multiple-bond formation is the principal driver of bond-length variation; the strong π bonds are a product of polymerization and displace­ment of the cation away from the centre of the polyhedron via the PJTE. In KCu^2+^_5_V^5+^_3_O_13_ (ICSD 400802; Martin & Müller-Buschbaum, 1994*a* ▸), with monomeric [V^5+^O_4_]^3−^ units, the *a priori* bond valences for V3 are 1.151 (O1), 1.121 (O8) and 2 × 1.364 (O10, O12) v.u. In this tetrahedron, V^5+^ moves off-centre toward O8 and away from O1, resulting in observed bond valences of 1.448 (O8) and 0.738 v.u. (O1) for a particularly strong case of PJTE; Δ_topol_ = 0.081 and Δ_cryst_ = 0.123 v.u. In Cu^2+^_2_V^5+^_2_O_7_ (ICSD 171028; Krivovichev *et al.*, 2005 ▸), the *a priori* (observed) bond valences are 0.980 (0.908), 1.274 (1.279), 1.288 (1.205) and 1.291 (1.329) v.u. for V1, with Δ_topol_ = 0.135 and Δ_cryst_ = 0.039 v.u., and 0.976 (1.054), 1.271 (1.166), 1.288 (1.205) and 1.466 (1.478) v.u. for V2, with Δ_topol_ = 0.137 and Δ_cryst_ = 0.069 v.u.

^[6]^Cr^3+^ [Fig. 1 ▸(*l*)] has a somewhat regular distribution of bond lengths, with two anomalies: (i) a spike of bond lengths at 1.99 Å (v.u.) and (ii) a relatively long tail at shorter bond lengths. For (i), the bond distances originate from a study of the (Mg,Fe^2+^)Cr_2_^3+^O_4_ solid-solution series in spinels, whereby 11 structures were refined with holosymmetric [Cr^3+^O_6_]^9−^ units, thus providing 66 distances of 1.99 Å for our dataset (Lenaz *et al.*, 2004 ▸). For (ii), we calculated the *a priori* bond valences for the structures which have the shortest distances observed. The shortest distances are observed in Cr^2+^_3_Cr^3+^_4_(PO_4_)_6_ (ICSD 73261; Glaum, 1993 ▸), with *a priori* (observed) bond valences between 0.409–0.639 (0.383–0.617) v.u., with Δ_topol_ = 0.074 and Δ_cryst_ = 0.038 v.u. for Cr1, and 0.415–0.715 (0.396–0.643) v.u., with Δ_topol_ = 0.089 and Δ_cryst_ = 0.061 v.u. for Cr2, thus showing the non-local bond-topological asymmetry as the origin of bond-length variation. For a total of seven coordination polyhedra for which we calculated Δ_topol_ and Δ_cryst_, the mean values for these indices are 0.077 and 0.056 v.u., respectively. With a *d* ^3^ electronic configuration, Cr^3+^ is not susceptible to the JTE, suggesting that crystal-structure effects are a significant cause of bond-length variation for this ion configuration.

^[4]^Cr^6+^ [Fig. 1 ▸(*m*)] has a multimodal distribution of bond lengths where bonded to O^2−^. [Cr^6+^O_4_]^2−^ units polymerize as linear oligomers (primarily dimers), in addition to forming very strong π bonds in monomers. As discussed for ^[4]^V^5+^ above, polymerization of the [Cr^6+^O_4_]^2−^ unit inherently results in varying bond lengths whereby the mean bond-valence for this ion configuration is >1 v.u. [special case (i) of non-local bond-topological asymmetry]. Thus, the maxima in Fig. 1 ▸(*m*) result from the superposition of the bond-length constraints of dimers, 1 v.u. (1.799 Å) and 3 × (5/3) v.u. (1.608 Å), on the normal distribution of bond lengths for monomers (1.65 Å; 1.5 v.u.). We calculated the *a priori* (observed) bond valences for eight coordination polyhedra from three structures; average values of Δ_topol_ and Δ_cryst_ are 0.127 and 0.085 v.u., respectively, confirming that the primary reason for bond-length variation is non-local bond-topological asymmetry [special case (i)] for this ion configuration, closely followed by the PJTE which presumably accounts for most of Δ_cryst_. The structure of Tl^3+^_2_(Cr^6+^O_4_)_3_ (ICSD 201793; Riou *et al.*, 1986 ▸), made up of two crystallographically distinct monomeric units, is a case where bond-topological asymmetry is the root cause of bond-length variation in one polyhedron and the PJTE in the other. Its *a priori* (observed) bond valences are 1.2 (1.053), 2 × 1.4 (1.533 and 1.617) and 2 (1.843) v.u. for Cr1, and 4 × 1.5 (2 × 1.477 and 2 × 1.639) v.u. for Cr2. These values give Δ_topol_ = 0.250 and Δ_cryst_ = 0.164 for Cr1, and Δ_topol_ = 0 and Δ_cryst_ = 0.081 for Cr2. Multiple-bond formation appears to be the main reason underlying bond-length variation in a small number of structures, for example in (NH_4_)Fe^3+^(Cr^6+^O_4_)_2_ (ICSD 934; Gravereau *et al.*, 1977 ▸) which is made up of monomeric units, where each corner of the Cr1 tetrahedron shares one O^2−^ with Fe^3+^O_6_ octahedra. Mean bond valences of 1.5 v.u. for Cr^6+^ and 0.5 v.u. for Fe^3+^ do not result in a constraint of polymerization at O_br_, and thus the formation of a strong π bond (2.02 v.u.) by Cr^6+^ must result from proper multiple-bond formation.

^[4]^Fe^3+^ [Fig. 1 ▸(*u*)] has a unimodal distribution with anomalously low kurtosis, with a maximum at 1.85 Å (0.791 v.u., higher than the expected value of 0.75 v.u.). [Fe^3+^O_4_]^5−^ units polymerize into various oligomers, chains and frameworks via corner and/or edge-sharing, thus creating a wide range of bond-length constraints around that of 0.75 v.u. for a regular tetrahedron. These constraints effectively ‘flatten’ the shape of the distribution, similar to what happens in ^[4]^V^5+^. With a mean bond valence of 0.75 v.u., ^[4]^Fe^3+^ sometimes redistributes its bond valences to satisfy O_br_ requirements [special case (ii) of non-local bond-topological asymmetry, above]. For example, Fe—O—Fe bonds adjust to 1.764 Å (1.004 v.u.) (and 3 × 1. 884 Å) for Fe3 in K(Fe^3+^_11_O_17_) (ICSD 83285; Ito *et al.*, 1996 ▸), exactly satisfying the bond-valence requirement of O_br_ for this corner-sharing dimer. We calculated *a priori* bond valences for three coordination polyhedra in two structures: K(Fe_11_O_17_) (above) and NaZnFe^3+^_2_(PO_4_)_3_ (ICSD 280902; Hidouri *et al.*, 2002 ▸), with average values of Δ_topol_ and Δ_cryst_ of 0.055 and 0.048, respectively. Here, Δ_cryst_ is due to the selection of structures where the bond-valence sums deviate slightly from 3 v.u. (<10%) and not to crystal-structure effects.

^[6]^Co^2+^ [Fig. 1 ▸(*z*)] has a regular bond-length distribution when bonded to O^2−^, although with a very long tail at longer bond lengths. This ion is susceptible to the strong JTE when in the low-spin state (*d* ^7^) and to the weak JTE when in the high-spin state. Co^2+^ is usually presumed to be HS (Rulíšek & Vondrášek, 1998 ▸), in agreement with a regular distribution of bond lengths [Fig. 1 ▸(*z*)], in contrast to the classic bimodal distribution observed for ions under the effect of the strong JTE. Of the ten longest bonds observed for this ion configuration (with one bond length >2.35 Å), spin is only reported for one coordination polyhedron, in Co^2+^_3_(BPO_7_) (ICSD 51317; Yilmaz *et al.*, 2001 ▸), where Co^2+^ is in a low-spin state in the shape of an elongated polyhedron (bond lengths 2.018, 2.024, 2.035, 2.039, 2.346 and 2.448 Å). Similar bond-length patterns occur in other structures with no report of spin state, *e.g.* in SrCo^2+^_2_(As^5+^O_4_)_2_ (ICSD 400764; Osterloh & Müller-Buschbaum, 2014 ▸) and Co^2+^(SiO_3_) (ICSD 1705; Sasaki & Takéuchi, 2015 ▸), with bond lengths 4 × 2.01–2.09 and 2 × 2.41–2.45, and 4 × 1.98–2.11 and 2 × 2.39–2.52 Å, respectively. We calculated *a priori* (observed) bond valences for five coordination polyhedra from four crystal structures, namely YCo^2+^(BO_2_)_5_ (ICSD 20670; Abdullaev *et al.*, 1980 ▸), Co^2+^_2_Si(P_2_O_7_)_2_ (ICSD 82403; Glaum & Schmidt, 1996 ▸), Cu^2+^_2_Co^2+^O(B_2_O_5_) (ICSD 400438; Schaefer & Bluhm, 1994 ▸) and SrCo^2+^_2_(As^5+^O_4_)_2_ (ICSD 400764; Osterloh & Müller-Buschbaum, 2014 ▸). On average, Δ_topol_ = 0.055 and Δ_cryst_ = 0.056. This suggests that, while non-local bond-topological asymmetry is the main cause of bond-length variation for this ion configuration, the JTE still has a marked effect, especially where Co^2+^ is (presumably) in a low-spin state.

^[6]^Zn^2+^ [Fig. 1 ▸(*ai*)] has a regular distribution of bond lengths when bonded to O^2−^, with a very long tail at longer bond lengths. We calculated the *a priori* bond valences for five polyhedra from five crystal structures, and obtained average Δ_topol_ and Δ_cryst_ values of 0.056 and 0.050 v.u., respectively. In NaZnFe^3+^_2_(PO_4_)_3_ (ICSD 280902; Hidouri *et al.*, 2002 ▸), *a priori* (observed) bond valences are 2 × 0.252 (0.168 and 0.274), 0.263 (0.212), 0.266 (0.412) 0.416 (0.433) and 0.550 (0.558) v.u., with Δ_topol_ = 0.100 and Δ_cryst_ = 0.055 v.u. In Mn^2+^Zn_2_Ta^5+^_2_O_8_ (ICSD 85042; Rohweder & Müller-Buschbaum, 1989 ▸), *a priori* (observed) bond valences are 3 × 0.256 (0.092, 0.103 and 0.491) and 3 × 0.410 (0.306, 0.447 and 0.491) v.u., with Δ_topol_ = 0.077 and Δ_cryst_ = 0.129 v.u. The large Δ_cryst_ value is attributable to the PJTE for this polyhedron, as is the case for Zn4 in Zn_3_(Se^4+^O_3_)·3H_2_O (ICSD 280151; Harrison, 1999 ▸), which is displaced 0.34 Å off centre in its polyhedron, resulting in a wide range of bond lengths (1.985–2.484 Å). Bond-topological and PJT effects seem to be of a similar magnitude for this ion configuration.

^[10]^Y^3+^ [Fig. S1(*bv*)] is only found in one crystal structure, YCo^2+^(BO_2_)_5_ (ICSD 20670; Abdullaev *et al.*, 1980 ▸), although it covers a large range of bond distances. Calculation of *a priori* (observed) bond valences gives Δ_topol_ = 0.086 and Δ_cryst_ = 0.079 v.u. Thus, despite the strong distorting effect of the PJTE, bond-length variations primarily result from non-local bond-topological asymmetry for this ion configuration (this result may change with the refinement of additional crystal structures with this ion configuration). This configuration is the only one for Y^3+^ where Δ_cryst_ > Δ_topol_; bond-length variations in coordinations [6] to [9] primarily result from the PJTE.

^[6]^Mo^4+^ [Fig. S1(*cj*)] has a bimodal distribution of bond lengths with maxima at 1.85 (0.96) and 2.03 Å (0.62 v.u.). [Mo^6+^O_4_]^2−^ units occur as monomers and corner-sharing dimers. The monomers are more or less regular. In corner-sharing dimers, the [Mo^6+^O_4_]^2−^ units adjust their bond valences to 1 × 1 v.u. at O_br_ and 3 × 0.66 v.u. for the three non-bridging bonds. This rearrangement, a result of non-local bond-topological asymmetry [special case (ii)], results in a bimodal distribution of bond lengths. For example, *a priori* (observed) bond valences in Pb^2+^_2_[Mo^4+^_2_O(PO_4_)_2_(P_2_O_7_)] (ICSD 96454; Leclaire *et al.*, 2003 ▸) are 0.518 (0.548), 2 × 0.612 (0.599 and 0.612), 2 × 0.629 (0.586 and 0.594) and 1 (0.985) v.u., with Δ_topol_ = 0.111 and Δ_cryst_ = 0.023 v.u.

^[4]^Mo^6+^ [Fig. 1 ▸(*at*)] forms a unimodal bond-length distribution with a very wide dispersion of bond lengths when bonded to O^2−^, with the majority of MoO_4_ tetrahedra occurring as monomers. We calculated the *a priori* bond valences for eight polyhedra from five crystal structures, and obtained average Δ_topol_ and Δ_cryst_ values of 0.104 and 0.087 v.u., respectively. Although 〈Δ_cryst_〉 is much smaller than for ^[5]^Mo^6+^ and ^[6]^Mo^6+^, it shows that a considerable amount of bond-length variation is due to the PJTE for this ion configuration. Nonetheless, the effect of bond-topological asymmetry is stronger in most cases. One convincing example is for the structure of Zr(Mo^6+^O_4_)_2_ (ICSD 65512; Serezhkin *et al.*, 1987 ▸), with *a priori* (observed) bond valences 3 × 1.333 (1.296, 1.314, 1.629) and 2 (1.918) v.u., with Δ_topol_ = 0.250 and Δ_cryst_ = 0.109 v.u.

^[6]^Cd^2+^ [Fig. 1 ▸(*bd*)] forms a unimodal bond-length distribution with a few anomalously long bonds. Calculation of *a priori* (observed) bond valences for two structures gave average values of Δ_topol_ and Δ_cryst_ of 0.044 and 0.040 v.u., respectively. In Cd_3_Te^6+^O_6_ (ICSD 35084; Burckhardt *et al.*, 1982 ▸), *a priori* (observed) bond valences are 4 × 0.314 (2 × 0.324 and 2 × 0.409) and 2 × 0.373 (0.296) v.u., with Δ_topol_ = 0.026 and Δ_cryst_ = 0.061 v.u. Presumably, the contribution to Δ_cryst_ is due to the PJTE, which is not uncommon in *d* ^10^ transition metal complexes (Strömberg *et al.*, 1990 ▸; see also ^[6]^Hg^2+^ below). Anomalously long bonds, *e.g.* 2.754 Å in Cd_8_(OH)_12_(SO_4_)_2_(H_2_O) (ICSD 27222; Louër *et al.*, 1982 ▸) and 2.622 Å in BaCd(P_2_O_7_) (ICSD 39397; Murashova *et al.*, 1991 ▸), are valid and result from a mixture of unusual bond topologies and the PJTE.

^[4]^Re^7+^ [Fig. 1 ▸(*bl*)] forms what appears to be a multi-modal distribution of bond lengths made entirely of [Re^7+^O_4_]^−^ monomeric units, with a main maximum at 1.72 Å (1.73 v.u.) and other maxima at 1.69 (1.86) and 1.75 (1.61 v.u.) Å. We calculated the *a priori* (observed) bond valences for two coordination polyhedra in (V^4+^O)(Re^7+^O_4_)_2_ (ICSD 92317; Bastide *et al.*, 2000 ▸), with 3 × 1.667 (2 × 1.668 and 1.819) and 2 (1.819) Å for Re1, and 2 × 1.5 (1.632) and 2 × 2 (1.814 and 1.855) Å for Re2. The mean values of Δ_topol_ and Δ_cryst_ are 0.188 and 0.116 v.u. While the bond-length variation here is caused mainly by bond-topological asymmetry, Δ_cryst_ shows that the PJTE also has a marked effect for this ion configuration.

^[6]^Os^6+^ [Fig. S1(*dy*)] has a distinctly bimodal distribution of bond lengths, although it consists of one crystal structure, Rb_2_Na_4_[(Os^6+^O_2_)[(HO)_2_Te^6+^O_4_]_2_](H_2_O)_16_ (ICSD 78359; Levason *et al.*, 1994 ▸). The H positions were not refined for this structure, and we cannot calculate the *a priori* bond valences. We may only speculate that bond-topological asymmetry is at the root of bond-length variation, as the weak JTE (*d* ^2^ electronic configuration) is unlikely to result in such a marked variation (>0.25 Å).

^[6]^Hg^2+^ [Fig. 1 ▸(*bq*)] has a very messy distribution of bond lengths when bonded to O^2−^. This is partly due to the difficulty of defining a coordination polyhedron for this ion configuration across different structure types. Hg^2+^ typically forms two relatively short bonds (and a series of longer bonds for coordination numbers [3] to [7]), although this is not always true for [6]. For example, [Hg^2+^(H_2_O)_6_](Cl^7+^O_4_)_2_ (ICSD 1640; Johansson & Sandström, 1978 ▸) reports a holo-symmetric octahedron with bond lengths 6 × 2.342 Å (0.34 v.u.), in accord with our calculation of *a priori* bond valences for this structure. For a slightly irregular polyhedron (but not quite a [2+4] coordination) in Hg^2+^(PO_3_)_2_ (ICSD 280292; Weil & Glaum, 2000 ▸), *a priori* (observed) bond valences are 4 × 0.286 (0.222, 0.228, 0.320, 0.402) and 2 × 0.429 (0.439 and 0.543) v.u., with Δ_topol_ = 0.063 and Δ_cryst_ = 0.066 v.u. In this structure, the Hg^2+^ ion presumably moves off-centre as a result of the PJTE (Strömberg *et al.*, 1990 ▸; discussed further in Section 6.4).

### 5.2. Mechanism (2): Ion configurations primarily distorted via multiple-bond formation   

Ion configurations with BV_max_ 1.33–1.75 v.u. are ^[5–7]^Ti^4+^, ^[4]^Mn^5+^, ^[4]^Mn^6+^, ^[4,6–7]^Nb^5+^, ^[6]^Ta^5+^, ^[7]^Re^7+^, ^[6]^Os^6+^ and ^[5–6]^Os^7+^. Those with BV_max_ > 1.75 v.u. are ^[5–6]^V^4+^, ^[4–6]^V^5+^, ^[4]^Cr^6+^, ^[4]^Mn^7+^, ^[5–6]^Mo^5+^, ^[4–7]^Mo^6+^, ^[4]^Tc^7+^, ^[4–6]^W^6+^, ^[4–5]^Re^7+^ and ^[4–6]^Os^8+^. From the latter group, bond-length distributions for which the main driver of bond-length variation is the formation of π bonds according to the criteria described above are ^[5–6]^V^4+^, ^[5–6]^V^5+^, ^[5–6]^Mo^5+^ and ^[5]^Mo^6+^. These are discussed below.

^[5]^V^4+^ [Fig. 1 ▸(*f*)] forms a bimodal distribution of bond lengths with maxima at 1.61 (1.58) and 1.96 Å (0.60 v.u.), integrating for one and four bonds, respectively. ^[5]^V^4+^ forms one (vanadyl) π bond for all but one structure in our dataset, that of NaV_6_O_11_ (ICSD 202215; de Roy *et al.*, 1987 ▸), where [V^4+^O_5_]^6−^ occurs as a trigonal bipyramid (as opposed to the common square-pyramidal configuration) with bond valences 3 × 0.975 (1.785) and 2 × 0.423 v.u. (2.089 Å). We calculated *a priori* (observed) bond valences for two polyhedra from two crystal structures. In V^3+^_2_[V^4+^O(P_2_O_7_)_2_] (ICSD 64634; Johnson *et al.*, 1988 ▸), they are 4 × 0.5 (0.537) and 2 (1.823) v.u., with Δ_topol_ = 0.480 and Δ_cryst_ = 0.065 v.u., clearly demonstrating the bond-topological effect of π-bond formation and its effect on bond-length variation. In Pb^2+^_2_[V^4+^O(PO_4_)_2_] (ICSD 249142; Shpanchenko *et al.*, 2006 ▸), *a priori* (observed) bond valences are 2 × 0.648 (0.506 and 0.542), 2 × 0.690 (0.579 and 0.613) and 1.325 (1.818) v.u., with Δ_topol_ = 0.210 and Δ_cryst_ = 0.186 v.u. The high value of Δ_topol_ is primarily driven by π-bond formation, but presumably also by non-local bond-topological asymmetry (their respective contributions cannot be resolved). The very high value of Δ_cryst_ is somewhat suspicious. While the ^[5]^V^4+^ ion (*d* ^1^) is not susceptible to the JTE in a square-pyramidal coordination, some of the bond-length variation due to crystallographic effects is probably due to the PJTE, whereby the V^4+^ ion typically moves off-centre toward the O^2−^ ion of the vanadyl bond. In this particular structure, however, a more important factor is at play. We may trace the value of Δ_cryst_ to a calculated *a priori* bond valence of 1.325 v.u. for the π bond; this value is very low because the O^2−^ ion involved in the π bond is predicted to form a bond of 0.675 v.u. to Pb^2+^ (observed 0.167 v.u.). These values are not those observed in practice. Instead, the [V^4+^O_5_]^6−^ units form corrugated (and presumably highly strained) layers, and the high value of Δ_cryst_ results from structural incommensuration.

^[6]^V^4+^ [Fig. 1 ▸(*g*)] forms a trimodal distribution of bond lengths, with maxima around 1.61 (1.58), 2.00 (0.54) and 2.25 Å (0.27 v.u.). [V^4+^O_6_]^8−^ units occur as monomers, oligomers, chains, sheets and frameworks where bonded to O^2−^. By and large, these polyhedra adopt a [1+4+1] octahedral coordination, described by Schindler and co-workers as the number of vanadyl, equatorial and *trans* bonds of the polyhedron (listed in order), where the *trans* bond is the weakest bond formed (Schindler *et al.*, 2000 ▸). We calculated *a priori* (observed) bond valences for four polyhedra from as many crystal structures. In (V^4+^O)(Re^7+^O_4_)_2_ (ICSD 92317; Bastide *et al.*, 2000 ▸), made up of monomeric units, they are 3 × 0.333 (0.242 and 2 × 0.502), 2 × 0.5 (0.528) and 2 (1.744) v.u., with Δ_topol_ = 0.444 and Δ_cryst_ = 0.123 v.u. In V^4+^O(HPO_4_)(H_2_O)_0.5_ (ICSD 201658; Leonowicz *et al.*, 1985 ▸), made up of face-sharing dimers, they are 0.261 (0.207), 2 × 0.348 (0.453), 2 × 0.522 (0.621) and 2 (1.763) v.u., with Δ_topol_ = 0.444 and Δ_cryst_ = 0.117 v.u. These structures show that the formation of π bonds is clearly the main driver of bond-length variation for this ion configuration, in addition to non-local bond-topological effects which create variability among the non-vanadyl bonds. However, in Ca(V^4+^O)_2_(PO_4_)_2_ (ICSD 72886; Lii *et al.*, 1992 ▸), made up of corner-sharing dimers, the *a priori* (observed) bond valences are 4 × 0.5 (0.464, 0.472, 0.502 and 0.513) and 2 × 1 (0.388 and 1.624) v.u., with Δ_topol_ = 0.222 and Δ_cryst_ = 0.219 v.u. In this structure, the large value of Δ_topol_ results from non-local bond-topological asymmetry alone; however, the V^4+^ ion moves off-centre in the direction of the strong bond, resulting in one much stronger and one much weaker bond than predicted. It is unclear whether this phenomenon results from the weak JTE, the PJTE or a combination of both (they are not exclusive; see Bersuker, 2013 ▸). We presume that the PJTE is responsible for the off-centring, as we do not observe any similarly strong distorting effects from the weak JTE in the entirety of our dataset. Interestingly, we observe [V^4+^O_6_]^8−^ units to be quasi-regular in Sr_2_(V^4+^O_4_) (ICSD 71450; Range *et al.*, 1991 ▸), made up of corner-sharing sheets of octahedra. The near regularity is predicted via *a priori* (observed) bond valences 4 × 0.641 (0.675) and 2 × 0.718 (0.563) v.u., with Δ_topol_ = 0.034 and Δ_cryst_ = 0.074 v.u.

^[5]^V^5+^ [Fig. 1 ▸(*i*)] forms a bimodal distribution of bond lengths with maxima at about 1.64 (1.50) and 1.92 Å (0.73 v.u.), and two superimposed peaks at about 1.89 (0.79) and 1.99 Å (0.61 v.u.). [V^5+^O_5_]^5−^ units occur primarily in a square-pyramidal coordination (seldom with a sixth O^2−^ ion nearby, far too long for consideration as a bonded distance) but also as triangular-bipyramidal and intermediate coordinations. They occur as monomers, oligomers, chains, sheets, clusters and frameworks, sharing corners and/or edges. We calculated *a priori* (observed) bond valences for three polyhedra in as many crystal structures. In K_4_(Cu^2+^V^5+^_5_O_15_Cl) (ICSD 401042; Martin & Müller-Buschbaum, 1994*b* ▸), made up of [V^5+^O_5_]^5−^ monomer units, they are 4 × 0.75 (0.810) and 2 (1.873) v.u., with Δ_topol_ = 0.400 and Δ_cryst_ = 0.073 v.u. In V^5+^AlMo^6+^O_7_ (ICSD 280775; Galy *et al.*, 2002 ▸), made up of edge-sharing chains, they are 4 × 0.75 (2 × 0.628 and 2 × 0.943) and 2 (1.738) v.u, with Δ_topol_ = 0.400 and Δ_cryst_ = 0.179 v.u. While both these examples show the strong distorting effect of π-bond formation, significant off-centring via the PJTE also proves to be a significant cause of bond-length variation in V^5+^AlMo^6+^O_7_. In Cs(V^5+^_3_O_8_) (ICSD 50010; Oka *et al.*, 1997 ▸), made up of sheets of edge-sharing square pyramids with V^5+^O_6_, they are 2 × 0.750 (0.601 and 0.657), 0.875 (0.827), 1.188 (1.190) and 1.438 (1.703) v.u., with Δ_topol_ = 0.250 and Δ_cryst_ = 0.112 v.u. In this structure, bond-length variation associated with the strong π bond results from non-local bond-topological asymmetry and the PJTE.

^[6]^V^5+^ [Fig. 1 ▸(*j*)] forms a very messy distribution of bond lengths. We primarily observe the [V^5+^O_6_]^7−^ units as clusters, but also as monomers, dimers, chains, sheets and frameworks, sharing corners and/or edges. Of the 293 coordination polyhedra in our dataset, 251 originate from edge-sharing clusters and 223 from decavanadate clusters. As we show below, cluster-type polyanions have specific bond-valence constraints for each of their crystallographically distinct polyhedra, which further vary as a function of the symmetry of the structure. These constraints result in a very messy (although resolvable) appearance for Fig. 1 ▸(*j*), with added variability resulting from a suite of other effects. To elucidate the occurrence, preval­ence and magnitude of these effects, we calculated *a priori* (observed) bond valences for 11 polyhedra from six crystal structures containing this ion configuration, including a structure containing the decavanadate cluster. First, we show the effect of π-bond formation in V^5+^_2_Se^4+^_2_O_9_ (ICSD 89466; Millet *et al.*, 1999 ▸), with *a priori* (observed) bond valences 2 × 0.4 (0.176/0.230 and 0.685/0.651), 2 × 0.6 (0.549/0.683 and 0.685/0.746), 1 (1.121/0.934) and 2 (1.743/1.756) v.u. for V1/V2, with Δ_topol_ = 0.444/0.444 and Δ_cryst_ = 0.171/0.160 v.u. In addition, a significant amount of bond-length variation seems to be caused by non-local bond-topological asymmetry and the PJTE in this structure. The strong distorting effect of the PJTE is shown in Hg^2+^(V^5+^_2_O_6_) (ICSD 409521; Mormann & Jeitschko, 2001 ▸), with *a priori* (observed) bond valences 3 × 0.667 (0.201, 0.712 and 1.068), 2 × 0.917 (0.516 and 0.846) and 1.167 (1.621) v.u., with Δ_topol_ = 0.167 and Δ_cryst_ = 0.306 v.u. In hummerite, KMg(V^5+^_5_O_14_)(H_2_O)_8_ (ICSD 95929; Hughes *et al.*, 2002 ▸), made up of decavanadate clusters (the most complex calculation done for this work, solving for 77 *a priori* bond valences), the *a priori* (observed) bond valences are 2 × 0.553 (0.433 an 0.449), 0.836 (0.758), 0.845 (0.710), 1.102 (1.350) and 1.111 (1.282) v.u. for V1, with Δ_topol_ = 0.187 and Δ_cryst_ = 0.143 v.u., 0.280 (0.319), 0.564 (0.624), 0.573 (0.590), 0.870 (0.922), 1.203 (0.919) and 1.510 (1.638) v.u. for V2, with Δ_topol_ = 0.361 and Δ_cryst_ = 0.097 v.u., 0.316 (0.329), 0.600 (0.608), 0.609 (0.573), 0.920 (0.891), 0.988 (0.986) and 1.568 (1.643) v.u. for V3, with Δ_topol_ = 0.325 and Δ_cryst_ = 0.027 v.u., 0.132 (0.273), 0.682 (0.513), 0.804 (0.752), 0.876 (0.917), 1.055 (0.901) and 1.451 (1.668) v.u. for V4, with Δ_topol_ = 0.294 and Δ_cryst_ = 0.129 v.u., and 0.166 (0.246), 0.725 (0.557), 0.756 (0.831), 0.770 (0.798), 0.910 (0.901), 1.673 (1.672) v.u. for V5, with Δ_topol_ = 0.305 and Δ_cryst_ = 0.060 v.u. Thus, bond lengths for [V^5+^O_6_]^7−^ in the decavanadate cluster largely vary as a result of bond-topological asymmetry, including the formation of moderately strong π bonds (〈Δ_topol_〉 = 0.295 v.u.), and to a more modest extent as a result of the PJTE (〈Δ_cryst_〉 = 0.091 v.u.). The average values of Δ_topol_ and Δ_cryst_ are 0.290 and 0.173 v.u. for the 13 polyhedra considered for this ion configuration.

^[5]^Mo^5+^ [Fig. S1(*ck*)] occurs in two structures in our dataset. In Pb^2+^(Mo^5+^O)_10_(P_2_O_7_) (ICSD 417729; Leclaire *et al.*, 2007 ▸), it forms square-pyramidal monomeric units with bond lengths (bond valences) of 1.691 (1.872), 2 × 1.974 (0.760) and 2 × 1.981 Å (0.744 v.u.). The sixth shortest interatomic distance is at 3.155 Å, far too long to be considered a bond. The O^2−^ ion participating in the strong π bond does not bond to other cations. Thus, bond-length variation is primarily a result of π-bond formation for this ion configuration.

^[6]^Mo^5+^ [Fig. 1 ▸(*as*)] forms a bimodal distribution of bond lengths, with maxima at 1.68 (1.95) and 2.04 Å (0.62 v.u.). This ion configuration is characterized by the formation of a strong double bond (1.953 v.u. on average), sometimes followed by polymerization into corner-sharing or edge-sharing dimers. We calculated *a priori* (observed) bond valences for two polyhedra in as many crystal structures. In Ba(Mo^5+^_2_P_4_O_16_) (ICSD 69088; Costentin *et al.*, 1990 ▸), made up of [Mo^5+^O_6_]^7−^ monomeric units, they are 2 × 0.577 (0. 391 and 0.534), 2 × 0.604 (0.651 and 0.659), 0.639 (0.676) and 2 (2.079) v.u., with Δ_topol_ = 0.389 and Δ_cryst_ = 0.075 v.u., showing the strong distorting effect of π-bond formation for this ion configuration. In Pr_3_Mo^5+^O_7_ (ICSD 281197; Barrier & Gougeon, 2003 ▸), a less common chain structure, *a priori* (observed) bond valences are 2 × 0.746 (0.663 and 0.810) and 4 × 0.877 (0.529, 0.682, 0.906 and 1.114) v.u., with Δ_topol_ = 0.058 and Δ_cryst_ = 0.159 v.u. Thus no π bond is predicted for this structure, and bond-length variation is primarily driven by crystallographic factors, most likely the weak JTE (*d* ^1^ electronic configuration).

^[5]^Mo^6+^ [Fig. 1 ▸(*au*)] forms what appears to be a unimodal bond-length distribution, despite the formation of a strong π bond in all of its structures (1.738 v.u. on average). The somewhat unimodal appearance is due to a continuous series of observed bond valences whereby [Mo^6+^O_5_]^4−^ units form multiple strong bonds (*e.g.* 1.552 v.u. on average for the second shortest bond). [Mo^6+^O_5_]^4−^ units occur as monomers with square-pyramidal and triangular-bipyramidal shape, sometimes polymerizing into chains, sheets and frameworks with Mo^6+^O_6_ octahedra. We calculated *a priori* (observed) bond valences for three polyhedra from as many crystal structures. In Cs_2_(Mo^6+^_3_O_10_) (ICSD 280066; Enjalbert *et al.*, 1999 ▸), a representative example, they are 2 × 0.742 (0.653 and 0.903), 1.285 (0.970), 1.593 (1.706) and 1.638 (1.755) v.u., with Δ_topol_ = 0.367 and Δ_cryst_ = 0.159 v.u. Thus, bond-length variations result primarily from a mixture of π-bond formation and non-local bond-topological asymmetry, followed by off-centring of the cation via the PJTE. In Cs[Mo^6+^_2_O_3_(PO_4_)_2_] (ICSD 79517; Hoareau *et al.*, 1995 ▸), *a priori* (observed) bond valences are 0.915 (0.850), 1.026 (0.801), 1.078 (0.735) and 2 × 1.491 (1.735 and 1.891) v.u., with Δ_topol_ = 0.232 and Δ_cryst_ = 0.256 v.u. In Cs(Np^5+^O_2_)(Mo^6+^O_4_) (ICSD 66994; Grigor’ev *et al.*, 1991 ▸), they are 2 × 1.022 (0.615 and 1.007), 2 × 1.177 (1.252 and 1.539) and 1.601 (1.579) v.u., with Δ_topol_ = 0.161 and Δ_cryst_ = 0.176 v.u. Thus the PJTE has a more important effect on bond-length variation than the combined effect of π-bond formation and non-local bond-topological asymmetry in these two structures.

### 5.3. Mechanism (3)(i): Ion configurations primarily distorted via coupled electronic vibrational degeneracy   

^[6]^Cr^2+^ [Fig. 1 ▸(*k*)] forms a clear bimodal distribution, typical of that observed for octahedrally coordinated cations under the influence of the strong JTE. We calculated *a priori* (observed) bond valences for two of the seven structures in our dataset containing this ion configuration (the two an­hydrous structures). In Cr^2+^_3_Cr^3+^_4_(PO_4_)_6_ (72302; Glaum, 1992 ▸), the *a priori* (observed) bond valences are 4 × ∼0.25 (0.08–0.45) and 2 × ∼0.5 (0.48–0.58) v.u., with Δ_topol_ = 0.170 and Δ_cryst_ = 0.118 v.u. In Cr^2+^_3_Cr^3+^_4_(PO_4_)_6_ (ICSD 73261; Glaum, 1993 ▸), they are 2 × 0.568 (0.484), 2 × 0.174 (0.199) and 2 × 0.259 (0.244) v.u., with Δ_topol_ = 0.156 and Δ_cryst_ = 0.041. The fact that Δ_topol_ > Δ_cryst_ in these structures probably results from structure selection (*a priori* bond valences could not be calculated for the hydrated structures); the other nine polyhedra for this ion configuration clearly follow the [4+2] coordination expected of a ‘*z*-out’ Jahn–Teller distortion. We list the JTE as the main factor underlying bond-length variation for this ion configuration, although it is closely followed by the effect of non-local bond-topological asymmetry.

^[6]^Mn^3+^ [Fig. 1 ▸(*q*)] forms a bimodal distribution of bond lengths, typical of the strong JTE. However, inspection of the data making up the distribution reveals that of the 82 coordination polyhedra, only 39 have a distinct [4+2] (‘*z*-out’) coordination, while 17 have a [2+2+2] coordination, six have a [2+4] (‘*z*-in’) and two are regular (18 are ambiguous). A [4+2] coordination is easily explained by the strong JTE, for example in KMn^3+^(Se^6+^O_4_)_2_ (ICSD 80430; Giester, 1995 ▸), with Δ_topol_ = 0.008 and Δ_cryst_ = 0.211 v.u., or Gd(Mn^3+^O_3_) (ICSD 95493; Mori *et al.*, 2002 ▸), with Δ_topol_ = 0.039 and Δ_cryst_ = 0.186 v.u. On the other hand, [2+2+2]-coordinated polyhedra appear to result as a mixture of the strong JTE and bond-topological effects. For example, edge-shared chains in gaudefroyite, Ca_4_Mn^3+^_3_B_3_O_12_CO_3_ (ICSD 24973; Yakubovich *et al.*, 1975 ▸), have *a priori* (observed) bond valences 2 × 0.404 (0.155 and 0.230), 2 × 0.480 (0.506 and 0.874) and 2 × 0.616 (0.806 and 0.846) v.u., with Δ_topol_ = 0.077 and Δ_cryst_ = 0.211 v.u. Regular polyhedra are probably due to disordered JT distortion, as shown by high anisotropic displacement parameters in Mn^2+^_3_Mn^3+^_2_(SiO_4_)_3_ (ICSD 86935; Arlt *et al.*, 1998 ▸).

^[6]^Cu^2+^ [Fig. 1 ▸(*af*)] forms a smooth bimodal distribution of bond lengths. Inspection of the data making up the distribution shows that of the 365 coordination polyhedra, 269 have distinct [4+2] (‘*z*-out’) coordination, 49 have a [2+2+2] coordination, ten have a [2+4] (‘*z*-in’) coordination and seven are regular (30 are ambiguous). [4+2] coordination follows classic (strong) JTE arguments, for example in Cu^2+^_2_V^5+^_2_O_7_ (ICSD 171028; Krivovichev *et al.*, 2005 ▸), with Δ_topol_ = 0.096 and Δ_cryst_ = 0.127 v.u., and in Cu^2+^_2_Co^2+^O(B_2_O_5_) (ICSD 400438; Schaefer & Bluhm, 1994 ▸), with Δ_topol_ = 0.046 and Δ_cryst_ = 0.161 v.u. An example of [2+4] coordination is that of vesignieite, Cu^2+^_3_Ba(V^5+^O_4_)_2_(OH)_2_ (ICSD 67726; Zhesheng *et al.*, 1991 ▸), with *a priori* (observed) bond valences 2 × 0.459 (0.54) and 4 × 0.270 (2 × 0.246 and 2 × 0.253) v.u., with Δ_topol_ = 0.084 and Δ_cryst_ = 0.039. It is interesting that Δ_topol_ > Δ_cryst_ for two of the three coordination polyhedra above. While this may be accidental, this result shows two things: (i) bond-topological effects are an important driver of bond-length variation for this ion configuration, and (ii) some structures appear to accommodate the bond-valence constraints of JTEs ions (how much of this is a result of observational bias is currently unclear). We further calculated the *a priori* (observed) bond valences for a regular octahedron in Ba_3_Cu^2+^(Sb^5+^_2_O_9_) (ICSD 2279; Køuhl, 1978 ▸) as 3 × 0.270 (0.340) and 3 × 0.396 (0.330) v.u., with Δ_topol_ = 0.063 and Δ_cryst_ = 0.070 v.u. It is therefore interesting that, for this structure, Cu^2+^O_6_ is indeed expected to distort for bond-topological reasons, but does not for crystallographic reasons (dynamic JTE).

### 5.4. Mechanism (3)(ii): Ion configurations primarily distorted via coupled electronic vibrational near-degeneracy   

^[4]^Ti^4+^ [Fig. S1(*g*)] occurs in four coordination polyhedra in three crystal structures. The largest bond-length variation is observed in chains of corner-sharing octahedra in Rb_2_(Ti^4+^O_3_) (ICSD 78842; Weiß & Hoppe, 1996 ▸), where it forms its strongest observed bond (1.233 v.u.; 1.747 Å). *A priori* (observed) bond valences for this structure are 2 × 0.926 (0.858) and 2 × 1.074 (1.208 and 1.233) v.u., with Δ_topol_ = 0.074 and Δ_cryst_ = 0.107 v.u. The PJTE has slightly stronger distorting power than the effect of non-local bond-topological asymmetry for this ion configuration.

^[5]^Ti^4+^ [Fig. 1 ▸(*c*)] forms a peculiar bond-length distribution with a maximum at 1.96 Å (0.66 v.u.) and a very long tail at shorter bond lengths. The shape of the distribution results mainly from the formation of a strongly bonded axial ligand in square-pyramidal geometry, relative to the equatorial ligands. [Ti^4+^O_5_]^6−^ units range in shape from square-pyramidal to distorted square-pyramidal to triangular-bipyramidal, with the strongest bond decreasing in strength along that series. In Cs_2_(Ti^4+^O)(P_2_O_7_) (ICSD 72682; Protas *et al.*, 1991 ▸), [Ti^4+^O_5_]^6−^ units are square-pyramidal with *a priori* (observed) bond valences 4 × 0.739 (0.587, 0.617, 0.622 and 0.663) and 1.044 (1.66) v.u., with Δ_topol_ = 0.098 and Δ_cryst_ = 0.217 v.u. In La_3_Ti^4+^O_4_Cl_5_ (ICSD 33800; Hübner *et al.*, 1990 ▸), [Ti^4+^O_5_]^6−^ units are triangular-bipyramidal with *a priori* (observed) bond valences 2 × 0.625 (0.509), 2 × 0.915 (1.013 and 1.031) and 0.920 (1.019) v.u., with Δ_topol_ = 0.140 and Δ_cryst_ = 0.109 v.u. These examples show that the strongest cause underlying bond-length variation for this ion configuration may vary between non-local bond-topological asymmetry and the PJTE as a function of polyhedral shape. Overall, the PJTE results in larger bond-length variations than bond-topological asymmetry, with mean Δ_topol_ and Δ_cryst_ values of 0.119 and 0.163 v.u., respectively. For the square-pyramidal units, there is sometimes (but not always) a possibility of a sixth bond to complete the octahedron. However, these distances range from 2.70–3.77 Å in different structures, and we consider them far too long for inclusion as bonds.

^[6]^Ti^4+^ [Fig. 1 ▸(*d*)] forms a multi-modal bond-length distribution with subtle maxima arising from different effects. Primarily, [Ti^4+^O_6_]^8−^ units form oligomers, chains, rings, sheets, clusters and frameworks, both as strictly corner-sharing or edge-sharing units, and sometimes as a mixture of both, resulting in a wide range of bond-valence constraints. Adding to the intrinsic bond-valence constraints is the off-centring of Ti^4+^ due to the PJTE, resulting in a very large range of observed bond lengths (1.648–2.474 Å). The near-uniqueness of the bonding environment of each and every TiO_6_ octa­hedron in our dataset somewhat detracts from the usefulness of rationalizing the shape of its compound bond-length distribution. Some of the noteworthy features of Fig. 1 ▸(*d*) include a small maximum between 1.7–1.8 Å with a maximum at 1.74 Å, a strong maximum at ∼1.95 Å and a subtle maximum at 2.10 Å (0.44 v.u.). These maxima originate from the strongest and weakest bonds of corner-sharing chains, for which we typically observe one bond of 1.25–1.35 v.u. (1.72–1.74 Å) and five bonds of gradually decreasing strength in the ∼0.40–0.70 v.u. (1.94–2.13 Å) range, with an average bond length of 2.10 Å for the longest bond. A representative example is that of (Cs_0.07_Rb_0.95_)(Ti^4+^O)(As^5+^O_4_) (ICSD 280501; Womersley *et al.*, 1998 ▸), with *a priori* (observed) bond valences of 2 × 0.363 (0.397 and 0.673), 0.570 (0.631), 0.856 (0.525), 0.883 (0.474) and 0.964 (1.318) v.u. for Ti1, with Δ_topol_ = 0.235 and Δ_cryst_ = 0.250, and 0.379 (0.522), 0.449 (0.577), 0.603 (1.218), 2 × 0.786 (0.615 and 0.654) and 0.997 (0.418) v.u. for Ti2, with Δ_topol_ = 0.190 and Δ_cryst_ = 0.295 v.u. We may compare these numbers with the *a priori* (observed) bond valences of sheets of edge-sharing octahedra in Sm^3+^(Ti^4+^O_3_Cl) (ICSD 36608; Hübner & Gruehn, 1991 ▸): 3 × 0.560 (0.455 and 2 × 0.555), 2 × 0.747 (0.515 and 0.978) and 0.827 (0.858) v.u., with Δ_topol_ = 0.107 and Δ_cryst_ = 0.102 v.u. Furthermore, we calculated the *a priori* (observed) bond valences for sheets of edge- and corner-sharing octahedra of [Ti^4+^O_6_]^8−^ in La_2_(Ti^4+^_2_SiO_9_) (ICSD 75583; Benbertal *et al.*, 1994 ▸): 2 × 0.619 (0.663), 2 × 0.688 (0.619) and 2 × 0.693 (0.821) v.u. for Ti1, with Δ_topol_ = 0.032 and Δ_cryst_ = 0.080 v.u., and 0.544 (0.281), 2 × 0.625 (0.791), 2 × 0.685 (0.554) and 0.731 (0.871) v.u. for Ti2, with Δ_topol_ = 0.055 and Δ_cryst_ = 0.162 v.u. Altogether, we get average values of Δ_topol_ and Δ_cryst_ of 0.111 and 0.163 v.u., respectively, confirming that the PJTE has a larger effect on bond-length variation than non-local bond-topological asymmetry, overall.

^[7]^Ti^4+^ [Fig. S1(*j*)] is the only example of this coordination polyhedron, in [C(NH_2_)_3_]_4_[Ti^4+^O(CO_3_)_3_](H_2_O)_2_ (ICSD 66308; Li *et al.*, 1990 ▸), bonding to six O^2−^ ions from CO_3_ groups (0.30–0.46 v.u.) and making a partial double bond (1.50 v.u.) to a seventh O^2−^ ion (in turn forming two hydrogen bonds to NH_2_ groups). The positions of the H atoms were not refined, and we cannot calculate the *a priori* bond valences for this structure. There is, however, significant displacement of Ti^4+^ toward O1 and away from O9 (the apical bonds), suggesting the PJTE as the main reason underlying bond-length variation for this ion configuration.

^[6]^Y^3+^ [Fig. 1 ▸(*aj*)] has a somewhat regular distribution of bond lengths, with a main maximum at 2.27 Å (0.49 v.u.) and a more subtle maximum at 2.22 Å (0.55 v.u.). This latter maximum does not occur as a result of polymerization constraints; it either results from non-local bond-topological asymmetry, or because of slight (but consistent) off-centring of the central cation via the PJTE, resulting in some bonds in the range 2.20–2.24 Å. We calculated the *a priori* (observed) bond valences for olivine-structured NaY(GeO_4_) (ICSD 85497; Dudka *et al.*, 1986 ▸): 2 × 0.561 (0.490 and 0.548) and 4 × 0.470 (2 × 0.399 and 2 × 0.495) v.u., with Δ_topol_ = 0.040 and Δ_cryst_ = 0.046, showing non-local bond-topological asymmetry to be about equally responsible for bond-length variations for this ion configuration.

^[7]^Y^3+^ [Fig. 1 ▸(*ak*)] forms a regular distribution of bond lengths, with a ‘wide’ maximum, as also observed for ^[7]^Y^3+^ and ^[9]^Y^3+^. We calculated *a priori* (observed) bond valences for three coordination polyhedra from two polymorphs of Y_2_Ba_2_Cu^2+^Pt^4+^O_8_ [ICSD 63103 (Swinnea & Steinfink, 1987 ▸) and ICSD 65614 (Calestani *et al.*, 1988 ▸)]. In the first structure, Δ_topol_ = 0.001 and Δ_cryst_ = 0.044 v.u. for Y1, and Δ_topol_ = 0.004 and Δ_cryst_ = 0.056 v.u. for Y2. In the second structure, we calculate Δ_topol_ = 0.020 and Δ_cryst_ = 0.077 v.u. Together, these values show that the wide maximum observed in the bond-length distribution of this ion configuration is probably due to slight off-centring of Y^3+^, as opposed to resulting from non-local bond-topological asymmetry.

^[8]^Y^3+^ [Fig. 1 ▸(*al*)] forms a very wide bond-length distribution with high kurtosis. A sharp maximum at 2.39 Å (0.36 v.u.) accounts for the majority of polyhedra where Y^3+^ makes eight equal bonds (*i.e.* does not move off-centre). A slightly distorted variant of a regular polyhedron can be seen in paraniite-(Y), Ca_2_Y(As^5+^O_4_)(W^6+^O_4_)_2_ (ICSD 71562; Demartin *et al.*, 1992 ▸), with *a priori* (observed) bond valences of 4 × 0.317 (0.422) and 0.433 (0.362) v.u., with Δ_topol_ = 0.072 and Δ_cryst_ = 0.131 v.u. *A priori* (observed) bond valences are much more scattered in Y_3_Re^7+^O_8_ (ICSD 15505; Baud *et al.*, 1981 ▸), ranging from 0.254–0.486 (0.269–0.478) v.u., with Δ_topol_ = 0.111 and Δ_cryst_ = 0.083 for Y1, to 0.302–0.533 (0.251–0.571) v.u., with Δ_topol_ = 0.085 and Δ_cryst_ = 0.071 v.u. for Y2. In KY(W^6+^O_4_) (ICSD 411285; Gallucci *et al.*, 2000 ▸) *a priori* (observed) bond valences are 2 × 0.230 (0.495), 4 × 0.400 (2 × 0.174 and 2 × 0.427) and 2 × 0.470 (0.475) v.u., with Δ_topol_ = 0.058 and Δ_cryst_ = 0.088 v.u. The mean values of Δ_topol_ and Δ_cryst_ are 0.082 and 0.093 v.u. over those structures, showing the PJTE and non-local bond-topological asymmetry to be approximately equally responsible for bond-length variations for this ion configuration.

^[9]^Y^3+^ [Fig. 1 ▸(*am*)] forms a regular distribution of bond lengths with a wide maximum, as observed for other coordination numbers of this cation. *A priori* (observed) bond valences in Y_3_Re^7+^O_8_ (ICSD 15505; Baud *et al.*, 1981 ▸) are 0.270–0.495 (0.133–0.560) v.u., with Δ_topol_ = 0.078 and Δ_cryst_ = 0.110 v.u. Thus, it seems that both the PJTE and non-local bond-topological asymmetry cause bond-length variation for this ion configuration, and the widening of the maximum.

^[6]^Zr^4+^ [Fig. 1 ▸(*an*)] forms a regular distribution of bond lengths when bonded to O^2−^. We calculated *a priori* (observed) bond valences for five coordination polyhedra in four structures: Na_4_Zr_2_(SiO_4_)_3_ (ICSD 15545; Tran Qui *et al.*, 1981 ▸), with Δ_topol_ = 0.021 and Δ_cryst_ = 0.054 v.u., painite, (Ca_0.81_Na_0.19_)ZrB(Al_8.82_Ti^4+^_0.18_O_18_) (ICSD 55272; Armbruster *et al.*, 2004 ▸), with Δ_topol_ = 0 and Δ_cryst_ = 0.003 v.u. for Zr1 and Δ_topol_ = 0 and Δ_cryst_ = 0.041 v.u. for Zr2, Zr(Mo^6+^O_4_)_2_ (ICSD 65512; Serezhkin *et al.*, 1987 ▸), with Δ_topol_ = 0 and Δ_cryst_ = 0.041 v.u., and woehlerite, Na_2_Ca_4_ZrNb^5+^(Si_2_O_7_)_2_FO_3_ (ICSD 100158; Mellini & Merlino, 1979 ▸), with Δ_topol_ = 0.221 and Δ_cryst_ = 0.186 v.u. The average values of Δ_topol_ and Δ_cryst_ are 0.048 and 0.117 v.u., respectively, for these structures, showing that the PJTE accounts for more variability in bond lengths than non-local bond-topological asymmetry for this ion configuration.

^[4]^Nb^5+^ [Fig. S1(*cd*)] occurs in a single structure in our dataset, that of Cs_2_Nb^5+^_4_O_11_ (ICSD 26379; Gasperin, 1981 ▸), where Nb^5+^O_4_ and Nb^5+^O_6_ polyhedra link together to form a framework structure. For the Nb5 site ([4]-coordinated), two of the O^2−^ ions (O3 and O17) bond to 2 × ^[6]^Nb^5+^ and 1 × ^[4]^Nb^5+^, while the other two (O21) bond only to 2 × ^[6]^Nb^5+^. This creates a strong bond-topological mismatch between the bond-valence requirement of O^2−^ (2 v.u.) and an even distribution of bond valences for the NbO_6_ and NbO_4_ polyhedra. Thus, the [Nb^5+^O_4_]^3−^ unit makes two bridging bonds via O21 of 1.573 v.u. (1.742 Å), while the two bridging bonds made via O3 and O17 are weaker at 0.961 v.u. (1.926 Å). Although we could not calculate the *a priori* bond valences of this structure, we assume that the PJTE has a similarly strong effect on bond-length variability as bond-topological asymmetry for this ion configuration.

^[5]^Nb^5+^ [Fig. S1(*ce*)] occurs in four structures in our dataset. We calculated the *a priori* (observed) bond valences for two of these structures: for Na_5_Nb^5+^O_5_ (ICSD 24819; Darriet *et al.*, 1982 ▸) they are 3 × 0.981 (2 × 0.797 and 1.083) and 2 × 1.029 (1.028) v.u., with Δ_topol_ = 0.023 and Δ_cryst_ = 0.094 v.u., and for NaKLaNb^5+^O_5_ (ICSD 94743; Liao & Tsai, 2002 ▸) they are 4 × 0.978 (0.877) and 1.088 (1.243) v.u., with Δ_topol_ = 0.035 and Δ_cryst_ = 0.112 v.u. These values show that the PJTE is the main reason underlying bond-length variation for ^[5]^Nb^5+^, with a non-negligible contribution from non-local bond-topological asymmetry.

^[6]^Nb^5+^ [Fig. 1 ▸(*ar*)] forms a symmetric distribution of bond lengths with a maximum at 1.98 Å (5/6 v.u.). As we have seen above for ^[6]^Ti^4+^ and ^[4]^V^5+^, a symmetric distribution typically results from an ion configuration with a strong proclivity for polymerization. [Nb^5+^O_6_]^7−^ units polymerize into dimers, chains, rings, clusters, sheets and frameworks, via corners, edges, faces and combinations of these. All of these polymerization circumstances lead to different *a priori* bond valences (and thus bond lengths) for the constituent crystal structures; the resulting distribution of these *a priori* bond lengths approaches that of a Gaussian distribution, in contrast to the positively skewed shape of simpler bond-length distributions driven by a two-body Morse potential. For example, monomeric [Nb^5+^O_6_]^7−^ units are evenly distributed between [1+4+1] coordination and regular octahedra in our dataset. The off-centring of the Nb^5+^ ion via the PJTE, with no preferential off-centring direction (Ok *et al.*, 2006 ▸), contributes to further symmetrization of the distribution. The combined effect of these phenomena gives a wide range of observed bond lengths of 1.702–2.479 Å (∼0.06 Å smaller than ^[6]^Ti^4+^). To compare the magnitude of bond-length variability caused by bond-topological asymmetry and the PJTE, we calculated the *a priori* (observed) bond valences for 15 polyhedra in 12 structures containing ^[6]^Nb^5+^. In La(Nb^5+^_5_O_14_) (ICSD 33783; Hofmann & Gruehn, 1990 ▸), they are 0.727 (0.607), 2 × 0.796 (0.610 and 1.068), 0.893 (0.891) and 2 × 0.894 (0.573 and 1.250) v.u., with Δ_topol_ = 0.061 and Δ_cryst_ = 0.210 v.u. In CaNb^5+^_2_(P_4_O_13_)(P_2_O_7_)O (ICSD 62577; discussed above), they are 2 × 0.805 (0.993), 2 × 0.837 (0.824) and 2 × 0.859 (0.869) v.u. for Nb1, with Δ_topol_ = 0.019 and Δ_cryst_ = 0.071 v.u., and 0.261 (0.462), 2 × 0.782 (0.714), 2 × 0.831 (0.837) and 1.515 (1.753) v.u. for Nb2, with Δ_topol_ = 0.227 and Δ_cryst_ = 0.098 v.u. In Li(Nb^5+^U^6+^O_6_) (ICSD 416590; Surblé *et al.*, 2006 ▸), they are 2 × 0.627 (0.835 and 0.893), 2 × 0.647 (0.810), 0.971 (0.248) and 1.480 (1.408) v.u., with Δ_topol_ = 0.261 and Δ_cryst_ = 0.266 v.u. We obtain average values of Δ_topol_ and Δ_cryst_ of 0.125 and 0.167 v.u. over the 15 polyhedra, respectively. These values demonstrate the substantial role of bond-topological asymmetry in driving bond-length variation in [Nb^5+^O_6_]^7−^ units, while confirming the PJTE as the main source of variation.

^[7]^Nb^5+^ [Fig. S1(*cg*)] occurs in three coordination polyhedra in two crystal structures. In La(Nb^5+^_5_O_14_) (ICSD 33783; Hofmann & Gruehn, 1990 ▸), *a priori* (observed) bond valences are 2 × 0.591 (0.240 and 0.849), 0.688 (0.590), 2 × 0.739 (0.497 and 1.037) and 2 × 0.826 (0.865 and 0.888) v.u. for Nb1, with Δ_topol_ = 0.078 and Δ_cryst_ = 0.192 v.u., and 0.545 (0.506), 4 × 0.614 (2 × 0.613 and 2 × 0.617) and 2 × 1 (0.582 and 1.470) v.u., with Δ_topol_ = 0.163 and Δ_cryst_ = 0.133 v.u. Average values for Δ_topol_ and Δ_cryst_ are 0.121 and 0.163, respectively.

^[6]^Mo^6+^ [Fig. 1 ▸(*av*)] forms a peculiar trimodal distribution of bond lengths when bonded to O^2−^, with maxima at 1.71 (1.74), 1.92 (0.95) and 2.30 Å (0.32 v.u). The [Mo^6+^O_6_]^6−^ units occur as monomers, dimers and other oligomers, chains, rings, sheets, clusters and frameworks, sharing corners, edges and/or faces. The near-uniqueness of the bonding environment of each and every Mo^6+^O_6_ octahedron in our dataset renders the elucidation of all bond-valence constraints leading to the observed bond-length distribution very lengthy and tedious. Here, we will simply calculate *a priori* (observed) bond valences for various modes of polymerization (from ten coordination polyhedra and eight structures) to determine whether bond-topological asymmetry, or the PJTE, is the main reason underlying bond-length variation for this ion configuration. Some of the results are given below, ordered in increasing degree of polymerization of the [Mo^6+^O_6_]^6−^ unit. In K_2_Mo^6+^O_2_(I^5+^O_3_)_4_ (ICSD 170119; Ok & Halasyamani, 2005*a* ▸), with monomeric [Mo^6+^O_6_]^6−^ units, the *a priori* (observed) bond valences are 2 × 0.951 (1.740), 2 × 1.001 (0.384) and 2 × 1.048 (0.862) v.u., with Δ_topol_ = 0.033 and Δ_cryst_ = 0.531 v.u.; this is the largest value we calculate for our entire dataset. In Na_2_Mo^6+^_3_Te^4+^_3_O_16_ (ICSD 171758; Chi *et al.*, 2006 ▸), made up of trimers of edge-sharing octahedra, Δ_topol_ = 0.152 and Δ_cryst_ = 0.340 v.u. for Mo1, and Δ_topol_ = 0.224 and Δ_cryst_ = 0.261 v.u. for Mo2. In Rb_2_Se^4+^Mo^6+^O_6_ (ICSD 413000; Porter & Halasyamani, 2003 ▸), made up of chains of corner-sharing octahedra, we get Δ_topol_ = 0.102 and Δ_cryst_ = 0.496 v.u. In Cs_2_(Mo^6+^_3_O_10_) (ICSD 280066; Enjalbert *et al.*, 1999 ▸), made up of chains of edge-sharing Mo^6+^O_6_ and Mo^6+^O_5_ polyhedra, Δ_topol_ = 0.322 and Δ_cryst_ = 0.215 v.u. In Pr^3+^_2_(Mo^6+^_4_O_15_) (ICSD 68279; Efremov *et al.*, 1988 ▸), made up of sheets of corner- and edge-sharing Mo^6+^O_4_ and Mo^6+^O_6_ polyhedra, Δ_topol_ = 0.418 and Δ_cryst_ = 0.235 v.u. Finally, in BaTe^4+^Mo^6+^_2_O_9_ (ICSD 281503; Ra *et al.*, 2003 ▸), a framework of corner-sharing [Mo^6+^O_6_]^6−^ octahedra, we have Δ_topol_ = 0.282 and Δ_cryst_ = 0.214 v.u. for Mo1, and Δ_topol_ = 0.328 and Δ_cryst_ = 0.161 v.u. for Mo2. Altogether, 〈Δ_topol_〉 = 0.218 and 〈Δ_cryst_〉 = 0.298 v.u.; this 〈Δ_cryst_〉 value is the largest of our dataset, out of the 52 transition metal configurations for which we calculated these values in two or more structures. Next comes a closer examination of the data to rationalize the shape of the bond-length distribution. We find our dataset to be made of ∼50% clusters, ∼30% chains/rings, ∼10% monomers and 10% of other degrees of polymerization. We find that for clusters, chains/rings and monomers, our data form three groups of two bonds, in agreement with Ok and co-workers who found that octa­hedrally coordinated Mo^6+^ preferentially moves off-centre toward an edge (and sometimes a face; Ok *et al.*, 2006 ▸). The mean bond valences are 1.706, 0.926, and 0.362 v.u. for clusters, and 1.715, 0.901, and 0.362 v.u. for chains and rings. These values compare exceptionally well with the three observed maxima of 1.74, 0.95 and 0.32 v.u. On the other hand, monomers have mean bond valences of 1.708, 0.821 and 0.473 v.u. Taken together, these values indicate that the trimodal shape of the bond-length distribution arises as a result of the combination of bond-topological constraints of polymerization (for clusters, chains and rings) and the preferential off-centring of Mo^6+^ toward an edge of the octahedron as a result of the PJTE.

^[6]^Hf^4+^ [Fig. 1 ▸(*bf*)] forms a regular distribution of bond lengths. We calculated *a priori* (observed) bond valences for three polyhedra in two structures. In Pb^2+^HfO_3_ (ICSD 33194; Zaitsev *et al.*, 1979 ▸), Δ_topol_ = 0.017 and Δ_cryst_ = 0.178 v.u. for Zr1, and Δ_topol_ = 0.003 and Δ_cryst_ = 0.162 v.u. for Zr2. In Ni^2+^_5_HfB_2_O_10_ (ICSD 65476; Bluhm & Müller-Buschbaum, 1989 ▸), Δ_topol_ = 0.063 and Δ_cryst_ = 0.026 v.u. These values indicate that the PJTE can have a very strong distorting effect on Hf^4+^O_6_ octahedra, and that non-local bond-topological asymmetry has some effect on bond-length variations for this ion configuration.

^[6]^Ta^5+^ forms a somewhat symmetrical unimodal distribution of bond lengths, reminiscent of that of ^[6]^Ti^4+^ [Fig. 1 ▸(*g*)]. Similar to ^[6]^Ti^4+^, ^[6]^Nb^5+^ and other ions with similarly symmetrical bond-length distributions, [Ta^5+^O_6_]^7−^ units are very susceptible to polymerization. [Ta^5+^O_6_]^7−^ octahedra occur as monomers, dimers, chains, sheets, clusters and frameworks, either sharing corners, edges and/or faces. We calculated *a priori* (observed) bond valences for six coordination polyhedra in as many crystal structures. In Na_2_Ca_3_Ta^5+^_2_O_9_ (ICSD 280154; Yamane *et al.*, 1999 ▸), made up of face-sharing dimers, they are 6 × 0.833 (3 × 0.533 and 3 × 1.023) v.u., with Δ_topol_ = 0 and Δ_cryst_ = 0.245 v.u. Comparing two structures where the [Ta^5+^O_6_]^7−^ units form chains of either corner- or edge-sharing octahedra, we get 4 × 0.75 (2 × 0.796 and 2 × 0.847) and 2 × 1 (0.762 and 1.029) v.u. for chains of corner-sharing octahedra in CsTa^5+^(B_2_O_5_) (ICSD 80423; Akella & Keszler, 1995 ▸), with Δ_topol_ = 0.111 and Δ_cryst_ = 0.092 v.u., and 4 × 0.75 (2 × 0.429 and 2 × 0.849) and 2 × 1 (1.167) v.u. for chains of edge-sharing octahedra in Yb(Ta^5+^O_4_) (ICSD 415460; Hartenbach *et al.*, 2005 ▸). Including the other structures not discussed here, we obtain average values for Δ_topol_ and Δ_cryst_ of 0.070 and 0.140 v.u. for this ion configuration. Thus the PJTE is the main reason underlying bond-length variation for this ion configuration.

^[7]^Ta^5+^ [Fig. 1 ▸(*bh*)] forms what appears to be a bimodal distribution of bond lengths when bonded to O^2−^, with a main maximum at 2.01 Å (0.76 v.u.) and a secondary maximum at 2.43 Å (0.22 v.u.). Pentagonal bipyramids typically polymerize into sheet and framework structures with TaO_6_ octahedra. We calculated the *a priori* (observed) bond valences for one polyhedron, in DyTa^5+^_7_O_19_ (ICSD 203232; Guo *et al.*, 1996 ▸), 0.583 (0.640), 4 × 0.667 (0.210, 0.606, 0.756, and 1.153), 0.750 (0.680) and 1 (1.075) v.u., with Δ_topol_ = 0.092 and Δ_cryst_ = 0.185 v.u. Thus the formation of the long and weak bond for this ion configuration is not a result of non-local bond-topological asymmetry, but rather results from the PJTE.

^[5]^W^6+^ [Fig. 1 ▸(*bj*)] has a regular distribution of bond lengths. We calculated the *a priori* (observed) bond valences for two polyhedra in as many structures. In KNa_3_(W^6+^O_5_) (ICSD 40249; Hoffmann & Hoppe, 1989 ▸), they are 2 × 1.192 (1.004 and 1.146), 2 × 1.197 (1.184) and 1.221 (1.393) v.u., with Δ_topol_ = 0.008 and Δ_cryst_ = 0.086 v.u. In La_2_(W^6+^O_4_)_3_ (ICSD 78180; Gärtner *et al.*, 1994 ▸), they are 2 × 0.952 (0.469 and 1.139), 1.238 (1.290) and 2 × 1.429 (1.512 and 1.652) v.u., with Δ_topol_ = 0.149 and Δ_cryst_ = 0.169 v.u. Thus the PJTE is probably the main driver of bond-length variation for this ion configuration, although the effect of non-local bond-topological asymmetry can be significant in some structures.

^[6]^W^6+^ [Fig. 1 ▸(*bk*)] has a peculiar trimodal distribution of bond lengths when bonded to O^2−^, with two maxima at 1.74 (1.65) and 1.92 Å (0.97 v.u.) and a third, very broad, maximum around 2.18 Å (0.45 v.u.). The [W^6+^O_6_]^6−^ units occur as monomers, chains, rings, sheets, clusters and frameworks, sharing corners and/or edges. The shape of the bond-length distribution is influenced by the fact that the majority of our dataset (roughly 2/3) involve clusters of more or less similar bond-valence constraints. The strongest and weakest bonds of these clusters are 1.69 (1.73) and 0.38 v.u. (2.24 Å) on average, with significant variability in between, presumably as a result of bond-topological constraints. The bonds in chains and rings tend to split into three groups, with mean bond valences of 2 × 1.5, 2 × 1 and 2 × 0.5 v.u. The main maximum at 1.92 Å (0.97 v.u.) represents the mean bond valence (statistically the most probable observation) for this ion configuration, although the [W^6+^O_6_]^6−^ unit is regular in only a few structures. We calculated the *a priori* (observed) bond valences for five polyhedra from four crystal structures. In K_2_Ni^2+^[W^6+^O_2_(PO_4_)_2_] (ICSD 79702; Peuchert *et al.*, 1995 ▸), made up of monomeric [W^6+^O_6_]^6−^ units, Δ_topol_ = 0.122 and Δ_cryst_ = 0.262 v.u. In KY(W^6+^O_4_)_2_ (ICSD 411285; Gallucci *et al.*, 2000 ▸), made up of chains of edge- and/or corner-sharing octahedra, Δ_topol_ = 0.075 and Δ_cryst_ = 0.357 v.u. In Cu^+^La(W^6+^_2_O_8_) (ICSD 68614; Boehlke & Müller-Buschbaum, 1990 ▸), made up of tetrameric clusters of edge-sharing octa­hedra, Δ_topol_ = 0.204 and Δ_cryst_ = 0.170 v.u. for W1 and Δ_topol_ = 0.183 and Δ_cryst_ = 0.239 v.u. for W2. Finally, in WO_3_ (ICSD 86144; Aird *et al.*, 1998 ▸), a framework of corner-sharing octahedra, *a priori* (observed) bond valences are 6 × 1 (0.443, 2 × 0.927, 2 × 1.174 and 1.865) v.u., with Δ_topol_ = 0 and Δ_cryst_ = 0.319 v.u. Altogether, the average values of Δ_topol_ and Δ_cryst_ are 0.117 and 0.269 v.u. for these structures, showing that the PJTE is the principal cause of bond-length variation for this ion configuration, with bond-topological asymmetry having a lesser role. Although many polyhedra have bond valences >1.75 v.u., these strong bonds result either from polymerization or the PJTE.

^[5]^Re^7+^ [Fig. S1(*dv*)] forms a regular but slightly negatively skewed distribution of bond lengths when bonded to O^2−^. The largest bond-length variation is in Ba_10_(Re^7+^O_5_)_6_Br_2_ (ICSD 100571; Baud *et al.*, 1979 ▸), with *a priori* (observed) bond valences of 2 × 1.391 (1.263), 2 × 1.402 (1.178) and 1.414 (1.882) v.u., with Δ_topol_ = 0.007 and Δ_cryst_ = 0.235 v.u. These values clearly show the PJTE to be the main cause of bond-length variation for this ion configuration.

^[6]^Re^7+^ [Fig. 1 ▸(*bm*)] forms a regular distribution of bond lengths when bonded to O^2−^. We calculated *a priori* (observed) bond valences for two coordination polyhedra from as many crystal structures. In Pr_3_(Re^7+^O_8_) (ICSD 92508; Jeitschko *et al.*, 2000 ▸), they vary between 1.134–1.228 (1.019–1.467) v.u., with Δ_topol_ = 0.040 and Δ_cryst_ = 0.077 v.u. In Y_3_Re^7+^O_8_ (ICSD 15505; Baud *et al.*, 1981 ▸), they vary between 1.143–1.240 (0.928–1.320) v.u., with Δ_topol_ = 0.025 and Δ_cryst_ = 0.128 v.u. Thus the PJTE is the main cause of bond-length variation for this ion configuration.

^[5]^Os^8+^ [Fig. S1(*ec*)] occurs in two structures in our dataset. In Rb[Os^8+^_2_O_8_(OH)] (ICSD 20611; Nevskii & Porai Koshits, 1983 ▸), the *a priori* (observed) bond valences are 1.235 (0.624), 2 × 1.647 (1.735 and 1.739) and 2 × 1.735 (1.693 and 2.140) v.u., with Δ_topol_ = 0.146 and Δ_cryst_ = 0.248 v.u. Thus the PJTE is the main cause of bond-length variation for this ion configuration.

^[6]^Os^8+^ [Fig. S1(*ed*)] forms a multi-modal distribution of bond lengths when bonded to O^2−^. In Li_2_[Os^8+^O_4_(OH)_2_] (ICSD 20540; Nevskii *et al.*, 1982 ▸), the *a priori* (observed) bond valences are 2 × 1.033 (0.607), 2 × 1.383 (1.756) and 2 × 1.583 (1.743) v.u., with Δ_topol_ = 0.200 and Δ_cryst_ = 0.320 v.u. While the PJTE is the main driver of bond-length variation for this ion configuration, bond-topological asymmetry also contributes a significant amount.

#### 5.4.1. Ion configurations primarily distorted via coupled electronic vibrational near-degeneracy for non-*d* ^0^ transition metals   

A variety of structural problems have been resolved via the pseudo Jahn–Teller effect for transition metals with non-*d* ^0^ electronic configurations in various coordination numbers (Reinen & Friebel, 1984 ▸; Reinen & Atanasov, 1991 ▸; Bersuker, 2013 ▸, and references therein). In our dataset, there are three [5]-coordinated ion configurations with a peculiar distribution of bond lengths whose shape may not be explained via bond-topological or classical Jahn–Teller arguments. While we observe the PJTE in a variety of ion configurations (*e.g.* in ^[6]^Zn^2+^, ^[6]^Hg^2+^ and others), listed below are the three non-*d* ^0^ non-octahedrally coordinated configurations of our dataset for which the PJTE is the main cause of bond-length variation.

^[5]^Cr^2+^ [Fig. S1(*r*)] occurs in two structures in our dataset. In Cr^2+^(HPO_3_)(H_2_O)_2_ (ICSD 63466; Brynda *et al.*, 1987 ▸), Cr^2+^ forms four bonds of 0.425–0.482 v.u. and one longer bond of 0.150 v.u. for a bond-valence sum of 2.001 v.u. In SrCr^2+^(P_2_O_7_) (ICSD 280309; Maaß & Glaum, 2000 ▸), the four strongest bonds of the [Cr^2+^O_5_]^8−^ unit vary over a wider range; *a priori* (observed) bond valences are 0.349 (0.369), 0.412 (0.514) and 3 × 0.413 (0.180, 0.427 and 0.430) v.u., with Δ_topol_ = 0.020 and Δ_cryst_ = 0.077 v.u. The effect of the PJTE on bond-length variation seems to be weaker than in Cr^2+^(HPO_3_)(H_2_O)_2_ but is clearly present. The four shortest bonded distances for these two polyhedra range between 1.994–2.110 Å, and the fifth distance is between 2.361–2.426 Å. The sixth shortest inter­atomic distance is between 2.964–3.3.053 Å, far too long to be a bond.

^[5]^Co^2+^ [Fig. 1 ▸(*y*)] forms a unimodal distribution with anomalously long bond lengths. There are five bonds making up the tail at longer bond lengths, representing the five (out of 15) polyhedra where [Co^2+^O_5_]^8−^ units appear to distort as a result of the PJTE. In BaCo^2+^_2_(Si_2_O_7_) (ICSD 81473; Adams *et al.*, 1996 ▸), *a priori* (observed) bond valences are 0.362 (0.397), 2 × 0.379 (0.178 and 0.442), 0.432 (0.448) and 0.449 (0.483) v.u., with Δ_topol_ = 0.032 and Δ_cryst_ = 0.070 v.u., showing that the PJTE is the main driver of bond-length variation for this ion configuration. The four shortest bonds in these five polyhedra range between 1.946–2.045 Å, and the fifth distance is between 2.345–2.574 Å. The sixth shortest interatomic distance is between 2.898–3.444 Å, far too long to be considered as a bond.

^[5]^Cu^2+^ [Fig. 1 ▸(*ae*)] forms a unimodal distribution of bond lengths when bonded to O^2−^, with a very long and flat tail for longer bonds. This ion configuration is very common (218 coordination polyhedra), making it an excellent candidate for probing the magnitude of the PJTE for [5]-coordinated cations. We calculated *a priori* (observed) bond valences for 13 polyhedra from nine crystal structures. In Cu^2+^_4_O(PO_4_)_2_ (ICSD 50459; Schwunck *et al.*, 1998 ▸), they are 0.445 (0.591), 3 × 0.335 (0.263 and 2 × 0.308) and 0.551 (0.658) v.u., with Δ_topol_ = 0.078 and Δ_cryst_ = 0.076 v.u. for Cu1 (distorted square pyramid), 0.293 (0.482), 2 × 0.391 (0.253), 0.408 (0.517) and 0.517 (0.427) v.u., with Δ_topol_ = 0.050 and Δ_cryst_ = 0.133 v.u. for Cu2 (trigonal bipyramid), and 0.295 (0.159), 0.384 (0.458), 0.393 (0.446), 0.409 (0.418) and 0.519 (0.564) v.u., with Δ_topol_ = 0.051 and Δ_cryst_ = 0.063 v.u. for Cu3 (square pyramid). In Cu^2+^_5_O_2_(PO_4_)_2_ (ICSD 1292; Brunel-Laügt & Guitel, 1977 ▸), they are 0.329 (0.101), 0.385 (0.486), 2 × 0.367 (0.445 and 0.458) and 0.551 (0.554) v.u., with Δ_topol_ = 0.060 and Δ_cryst_ = 0.100 v.u. for Cu2 (distorted square pyramid), and 2 × 0.277 (0.203 and 0.261), 0.444 (0.502) and 2 × 0.501 (0.486 and 0.505) v.u., with Δ_topol_ = 0.099 and Δ_cryst_ = 0.033 v.u. for Cu3 (distorted trigonal bipyramid). Although there is a weak correlation in which the weakest bond of the polyhedron becomes progressively stronger from a square-pyramidal to a triangular-bipyramidal configuration, there is no correlation between Δ_topol_ and Δ_cryst_ as a function of polyhedron shape. We obtain average values of Δ_topol_ and Δ_cryst_ of 0.061 and 0.084 v.u., with ranges of 0.016–0.099 and 0.033–0.133 v.u., respectively, for the 13 polyhedra. Thus, it seems that the long tail results from a mixture of continuous off-centring of Cu^2+^ via the PJTE, and non-local bond-topological asymmetry. One more structure worth discussing is that of PbCu^2+^(Cu^2+^Te^6+^O_7_) (ICSD 405329; Wedel & Müller-Buschbaum, 2014 ▸) with a particularly long bond (2.687 Å). The *a priori* (observed) bond valences in this structure are 0.265 (0.060), 0.372 (0.342), 2 × 0.399 (0.498) and 0.565 (0.590) v.u., with Δ_topol_ = 0.066 and Δ_cryst_ = 0.091 v.u. Thus, the weak bond in this structure results from a mixture of non-local bond-topological asymmetry (*a priori* bond valence 0.265 v.u. < 2/5 v.u.) and the PJTE.

### 5.5. Mechanism (4): Ion configurations primarily distorted via crystal-structure effects   

^[6]^Ru^5+^ [Fig. 1 ▸(*aw*)] occurs as monomers, chains of corner- and edge-sharing octahedra and face-sharing oligomers. Face-sharing octahedra have O_br_ bond valences 3 × 0.53–0.78 v.u., with O_br_ bonding to other cations. Non-bridging bonds vary between 0.83–1.17 v.u. in these structures. For example, *a priori* (observed) bond valences are 3 × 0.786 (0.667) v.u. for bridging and 3 × 0.881 (1.075) v.u. for non-bridging bonds for Ru^5+^ in Ba_3_Ca(Ru^5+^_2_O_9_) (ICSD 73183; Wilkens & Müller-Buschbaum, 1993 ▸), with Δ_topol_ = 0.048 and Δ_cryst_ = 0.156 v.u. In Ba_6_Ru^5+^_2_Na_2_Mn^5+^_2_O_17_ (ICSD 97525; Quarez *et al.*, 2003 ▸), *a priori* (observed) bond valences are 3 × 0.767 (0.633) and 3 × 0.900 (1.047) v.u., with Δ_topol_ = 0.067 and Δ_cryst_ = 0.140 v.u. The reason for the large values of Δ_cryst_ is unclear, but they may be due to slight Ru^5+^–Ru^5+^ interactions between the dimers (crystal-structure effects); Ru^5+^ is JT-inactive with a *d* ^3^ electronic configuration. Otherwise, monomers and chains of corner-sharing octahedra have regular polyhedra with a mean bond length of 1.957 Å (5/6 v.u.).

## 6. General discussion   

### 6.1. Bond-topological *versus* crystallographic effects   

**Figure 13:**
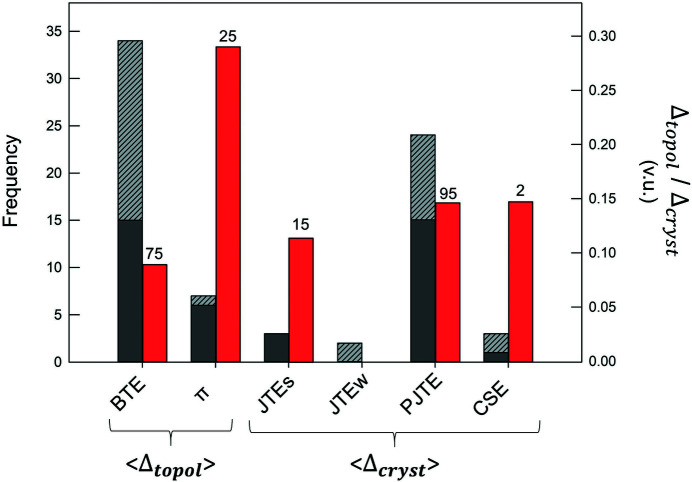
Frequency for which bond-topological effects (BTE), multiple-bond formation (π), the strong (JTEs) and weak (JTEw) Jahn–Teller effects, the pseudo Jahn–Teller effect (PJTE) and crystal-structure effects (CSE) are the main (dark grey) and minor (light grey) causes of bond-length variation underlying the ion configurations of Table 4 ▸. These data are necessarily biased toward anomalous bond-length distributions, whereas the BTE is ubiquitous across all ion configurations. Mean Δ_topol_ and Δ_cryst_ values (right-hand axis) are calculated using those polyhedra for which the given effect is the main cause of bond-length variation. The numbers atop each bar represent the sample size.

Of the 52 ion configurations of Table 4 ▸, 39 bond-length distributions may be considered to have a shape that deviates significantly from that expected for a two-body Morse potential. Non-local bond-topological effects are assigned as the main (minor) driving factor for 15 (19) of those 39 bond-length distributions, the strong JTE 3 (0), the weak JTE 0 (2), the PJTE 17 (9), π-bond formation 6 (1) and crystal-structure effects 1 (2). Similarly, 39 of the 52 ion configurations may be considered to have an anomalously large range of observed bond lengths (Table 4 ▸). Of those configurations, non-local bond-topological effects are assigned as the main (minor) driving factor for 14 (23), the strong JTE 3 (1), the weak JTE 0 (2), the PJTE 23 (8), π-bond formation 6 (1) and crystal-structure effects 1 (1). Fig. 13 ▸ summarizes these numbers, taking into consideration the overlapping nature of these datasets.

**Figure 14:**
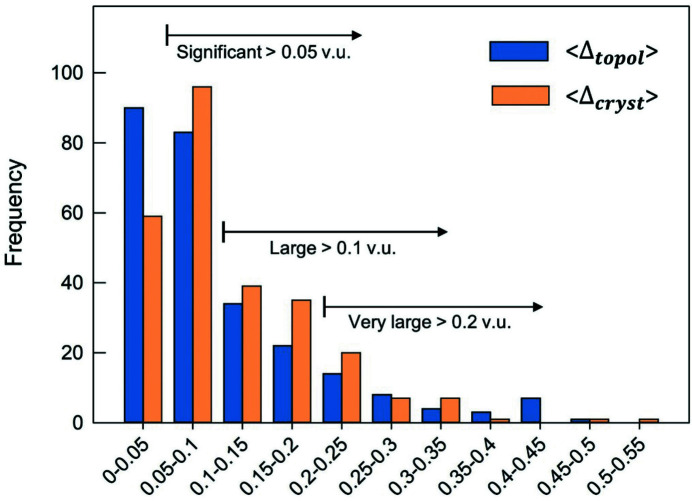
Distribution of observed Δ_topol_ and Δ_cryst_ values for the 266 transition metal polyhedra of this work for which *a priori* bond valences were calculated.

The distribution of observed Δ_topol_ and Δ_cryst_ values is given in Fig. 14 ▸ for the 266 coordination polyhedra for which *a priori* bond valences were calculated (Table S2); 〈Δ_topol_〉 = 0.102 and 〈Δ_cryst_〉 = 0.113 v.u. For the 235 coordination polyhedra with Δ_topol_ and/or Δ_cryst_ > 0.05 v.u., 〈Δ_topol_〉 = 0.113 and 〈Δ_cryst_〉 = 0.123 v.u. Next, we wish to calculate the average magnitude of these indices between the main two factors identified in transition metal oxyanions: non-local bond-topological effects and the PJTE. We remove the polyhedra of this dataset in which π bonding is the main cause of bond-length variation, *i.e.* those for ^[5]^V^4+^, ^[6]^V^4+^, ^[5]^V^5+^, ^[6]^V^5+^, ^[5]^Mo^5+^, ^[6]^Mo^5+^ and ^[5]^Mo^6+^, to get 〈Δ_topol_〉 = 0.091 and 〈Δ_cryst_〉 = 0.120 v.u. (these values are 0.291 and 0.155 v.u. for the 25 coordination polyhedra where π bonding is the main factor). Similarly, if we remove those coordination polyhedra where the main factor underlying bond-length variation is the strong or weak JTE (^[6]^Cr^2+^, ^[6]^Mn^3+^and ^[6]^Cu^2+^), 〈Δ_topol_〉 = 0.093 and 〈Δ_cryst_〉 = 0.120 v.u. for the remaining 195 polyhedra (those values are 0.076 and 0.114 v.u. for 16 coordination polyhedra where the strong/weak JTE is the main cause of bond-length variation). From here, it is not possible to remove the component of Δ_cryst_ which is due to crystal-structure effects, as those effects are both widespread and structure dependent. While Δ_cryst_ may be interpreted as mainly driven by the PJTE in this subset, a non-negligible component is due to crystal-structure effects. Thus, we are left comparing 〈Δ_topol_〉 = 0.091 and 〈Δ_cryst_〉 = 0.120 v.u. for 195 polyhedra. In terms of frequency, Δ_topol_ and Δ_cryst_ are >0.05 v.u. for 141 and 171 polyhedra, respectively, while Δ_topol_ > Δ_cryst_ for 78 of those polyhedra; however, we emphasize that this subset of data is biased toward ion configurations with a *d* ^0^ electronic configuration, to address other questions in this work. In terms of magnitude, eight of the 15 widest ranges of observed bond lengths in Table 4 ▸ result primarily from the PJTE, while only one is due to non-local bond-topological effects; however, non-local bond-topological effects act as a minor contributor in every case.

Thus we conclude the following: (i) non-local bond-topological asymmetry is the most frequently encountered cause of bond-length variation in transition metal oxides and oxysalts, closely followed by the PJTE; and (ii) bond-length variations resulting from the PJTE are slightly larger than those resulting from non-local bond-topological asymmetry, comparable with those resulting from the strong JTE, and less than those induced by π-bond formation. We further suggest non-local bond-topological asymmetry to be the most widespread cause of spontaneous distortion (and bond-length variation) in the solid state, with no *a priori* limitations with regard to ion identity.

### 6.2. Are bond-topological and crystallographic effects mutually supportive?   

Kunz and Brown suggested that the structure of the bond network influences the occurrence and magnitude of the PJTE, whereby the PJTE either results from, or is affected by, the arrangement of *a priori* bond valences in crystal structures with *d* ^0^ transition metals (Kunz & Brown, 1995 ▸). In this work, we calculated *a priori* and observed bond valences for 15 coordination polyhedra in which the strong JTE is the leading cause of bond-length variation, and 132 coordination polyhedra where the leading cause is the PJTE. Where Jahn–Teller distortions are accommodated by the crystal structure, we expect a high value of Δ_topol_, indicative of the polyhedron distorting away from a regular polyhedron toward a configuration compatible with the Jahn–Teller distortion, and low values of Δ_cryst_, as the distortion caused by the JTE is captured by Δ_topol_.

For the 15 coordination polyhedra where the strong JTE is the leading cause of polyhedral distortion, 〈Δ_topol_〉 = 0.081 v.u. and 〈Δ_cryst_〉 = 0.115 v.u., and Δ_cryst_ > Δ_topol_ for ten polyhedra. For the 132 coordination polyhedra where the leading cause is the PJTE, 〈Δ_topol_〉 = 0.129 v.u. and 〈Δ_cryst_〉 = 0.145 v.u., and Δ_cryst_ *>* Δ_topol_ for 79 polyhedra. Thus, while many individual polyhedra support a mutually supportive relation between bond-topological requirements of the structure and the JTE, many more do not.

For the strong JTE, strong correlation is observed for ^[6]^Cr^2+^ in Cr^2+^_3_Cr^3+^_4_(PO_4_)_6_ (ICSD 73261; Glaum, 1993 ▸), with *a priori* (observed) bond valences 2 × 0.568 (0.484), 2 × 0.174 (0.199) and 2 × 0.259 (0.244) v.u. Thus, this polyhedron strongly deviates from regularity (6 × 2/3 v.u.) in a way that accommodates the JT distortion; Δ_topol_ = 0.156 and Δ_cryst_ = 0.041 v.u. However, in Gd(Mn^3+^O_3_) (ICSD 95493; Mori *et al.*, 2002 ▸), *a priori* (observed) bond valences for Mn^3+^ are 4 × 0.471 (2 × 0.194 and 2 × 0.700) and 2 × 0.559 (0.612) v.u. for Mn^3+^. Thus, deviation from regularity (6 × 0.5 v.u.) is not captured by the bond topology; Δ_topol_ = 0.039 and Δ_cryst_ = 0.186 v.u. Another such example is that of Cu^2+^_2_Co^2+^O(B_2_O_5_) (ICSD 400438; Schaefer & Bluhm, 1994 ▸), with *a priori* (observed) bond valences of 3 × 0.304 (0.061, 0.436 and 0.469), 2 × 0.309 (0.099 and 0.453) and 0.470 (0.540) v.u. for ^[6]^Cu^2+^, with Δ_topol_ = 0.046 and Δ_cryst_ = 0.161 v.u.

For the PJTE, we find few structures where bond-topological and crystallographic effects appear to work in cooperation. Nonetheless, the decavanadate cluster represents a very good example of apparent cooperation. In hummerite, KMg(V^5+^_5_O_14_)(H_2_O)_8_ (ICSD 95929; Hughes *et al.*, 2002 ▸), Δ_topol_ > Δ_cryst_ for the five crystallographically distinct ^[6]^V^5+^ sites. The Jahn–Teller requirements are nearly perfectly matched for V3, with *a priori* (observed) bond valences 0.316 (0.329), 0.600 (0.608), 0.609 (0.573), 0.920 (0.891), 0.988 (0.986) and 1.568 (1.643) v.u., with Δ_topol_ = 0.325 and Δ_cryst_ = 0.027 v.u. We also showed the case for CaNb^5+^_2_(P_4_O_13_)(P_2_O_7_)O (ICSD 62577; Averbuch-Pouchot, 1987 ▸) earlier in the text, where of the two crystallographically distinct ^[6]^Nb^5+^ sites, one is significantly distorted as a result of bond-topological asymmetry, with Δ_topol_ = 0.227 and Δ_cryst_ = 0.098, while the other is not (Δ_topol_ = 0.019 and Δ_cryst_ = 0.071). However, the number of cases in which the JT distortion functions independent of (or against) bond-topological requirements is overwhelmingly large. In painite, (Ca_0.81_Na_0.19_)ZrB(Al_8.82_Ti^4+^_0.18_O_18_) (ICSD 55272; Armbruster *et al.*, 2004 ▸), *a priori* (observed) bond valences are 6 × 0.667 (3 × 0.187 and 3 × 0.599) v.u., with Δ_topol_ = 0 and Δ_cryst_ = 0.274 v.u. In K_2_Mo^6+^O_2_(I^5+^O_3_)_4_ (ICSD 170119; Ok & Halasyamani, 2005*a* ▸), *a priori* (observed) bond valences for ^[6]^Mo^6+^ are 2 × 0.951 (1.740), 2 × 1.001 (0.384) and 2 × 1.048 (0.862) v.u., with Δ_topol_ = 0.033 and Δ_cryst_ = 0.531 v.u. In WO_3_ (ICSD 86144; Aird *et al.*, 1998 ▸), *a priori* (observed) bond valences are 6 × 1 (0.443, 2 × 0.927, 2 × 1.174 and 1.865) v.u. for ^[6]^W^6+^, with Δ_topol_ = 0 and Δ_cryst_ = 0.319 v.u.

From the comparison of *a priori* and observed bond valences for ∼150 coordination polyhedra where either the strong JTE or pseudo JTE is the main reason underlying polyhedral distortion, we conclude that the Jahn–Teller effect does not have a cooperative relation with the bond-topological requirements of crystal structures.

### 6.3. PJTE: octahedrally coordinated *d* ^0^ transition metal complexes   

Octahedrally coordinated ions with *d* ^0^ electronic configuration are often ranked as a function of their ‘distorting power’ (the magnitude of their bond-length variation) on a qualitative scale, *e.g.* strong for Mo^6+^ and V^5+^, moderate for W^6+^, Ti^4+^, Nb^5+^ and Ta^5+^, and weak for Zr^4+^ and Hf^4+^ (Ok *et al.*, 2006 ▸). Our calculations allow a more quantitative ranking of distorting power via 〈Δ_cryst_〉 values taken from the data for octahedrally coordinated *d* ^0^ ions in Table S2. Thus ordering 〈Δ_cryst_〉 values in decreasing magnitude gives Os^8+^ (0.320, *n* = 1), Mo^6+^ (0.298, *n* = 10), W^6+^ (0.269, *n* = 5), V^5+^ (0.173, *n* = 11), Nb^5+^ (0.167, *n* = 15), Ti^4+^ (0.163, *n* = 6), Ta^5+^ (0.140, *n* = 6), Hf^4+^ (0.122, *n* = 3), Zr^4+^ (0.117, *n* = 5), Re^7+^ (0.103, *n* = 2), Y^3+^ (0.046, *n* = 1) and Sc^3+^ (0.029, *n* = 2). These 〈Δ_cryst_〉 values are not set in stone; they may vary slightly as more *a priori* bond valences are calculated for these ion configurations.

Moreover, we find the magnitude of bond-length variations to vary as a function of coordination number for *d* ^0^ ions. For [4]-coordination (〈Δ_cryst_〉, in v.u.), we have the following order: Re^7+^ 0.116 (*n* = 2), Ti^4+^ 0.107 (*n* = 1), V^5+^ 0.099 (*n* = 13), W^6+^ 0.098 (*n* = 2), Mo^6+^ 0.087 (*n* = 8), Cr^6+^ 0.085 (*n* = 8), Os^8+^ 0.077 (*n* = 1) and Mn^7+^ 0.028 (*n* = 1). For [5]-coordination, we observe Os^8+^ 0.248 (*n* = 1), Re^7+^ 0.235 (*n* = 1), Mo^6+^ 0.197 (*n* = 3), Ti^4+^ 0.163 (*n* = 2), W^6+^ 0.127 (*n* = 2), V^5+^ 0.121 (*n* = 3) and Nb^5+^ 0.103 (*n* = 2).

### 6.4. PJTE: beyond octahedrally coordinated *d* ^0^ transition metal complexes   

The inseparability of electronic and nuclear coordinates in Jahn–Teller systems makes JT-active compounds unsuited to density functional theory (DFT), whose foundation rests on the Born–Oppenheimer approximation (Bersuker, 1997 ▸, 2017 ▸). We have further showed that neither the JTE nor the PJTE may be modelled bond-topologically. As a result of these difficulties, materials design for JT-active compounds is often relegated to heuristic methods. Thus, we identify in our dataset the ion configurations for which the PJTE is observed to an appreciable extent in ‘non-traditional’ configurations. These data shine light onto new and potentially promising compositional spaces for materials discovery, notably for non-centrosymmetric structures.

Table S2 includes values of Δ_topol_ and Δ_cryst_ for 14 ions with *d* ^0^ electronic configuration with coordination numbers ranging from [4] to [10]. We summarize values of 〈Δ_topol_〉, 〈Δ_cryst_〉 and the maximum observed value of 〈Δ_cryst_〉 for these ions in Table 10 ▸, together with four ion configurations with non-*d* ^0^ electronic configuration whose primary cause of bond-length variation is the PJTE.

For [6]-coordination, with *n* = 67 polyhedra, 〈Δ_cryst_〉 = 0.181 v.u., with the highest Δ_cryst_ value of 0.531 v.u. in K_2_Mo^6+^O_2_(I^5+^O_3_)_4_ (ICSD 170119; Ok & Halasyamani, 2005*a* ▸). The value of 〈Δ_cryst_〉 is surprisingly close for [5]-coordination, 0.159 v.u., despite a much lower sample size (*n* = 14), followed by [7]-coordination with 〈Δ_cryst_〉 = 0.115 v.u. (*n* = 6), [9]-coordination with 〈Δ_cryst_〉 = 0.110 v.u. (*n* = 1), [4]-coordination with 〈Δ_cryst_〉 = 0.092 v.u. (*n* = 36), [8]-coordination with 〈Δ_cryst_〉 = 0.087 v.u. (*n* = 7) and [10]-coordination with 〈Δ_cryst_〉 = 0.079 v.u. (*n* = 1). Considering that crystal structures containing octahedrally coordinated *d* ^0^ ions have been the subject of intense scrutiny and targeted syntheses (*e.g.* Ok & Halasyamani, 2005*a* ▸,*b* ▸), it seems that non-traditional coordinations of *d* ^0^ ion configurations are likely to have a similarly large potential for bond-length variation via the PJTE to that of octahedrally coordinated complexes.

The PJTE is the main reason underlying bond-length variation for five non-*d* ^0^ ion configurations: ^[5]^Cr^2+^ (*d* ^4^), ^[5]^Co^2+^ (*d* ^7^), ^[5]^Cu^2+^ (*d* ^9^), and ^[6]^Zn^2+^ and ^[6]^Hg^2+^ (*d* ^10^). The PJTE for [5]-coordinated square-pyramidal complexes has been demonstrated for ^[5]^Cu^2+^ and several ligands (Reinen & Atanasov, 1991 ▸; Reinen & Friebel, 1984 ▸), and here we observe the same behaviour for [5]-coordinated complexes of what are otherwise octahedrally coordinated cations with electronic configurations prone to a strong JTE. The case for *d* ^10^ transition metal oxides is also interesting. The PJTE has been shown to be a significant cause of bond-length variation for Hg^2+^ in hexahydrate complexes (Strömberg *et al.*, 1990 ▸), with less unequivocal results for analogous Zn^2+^ (and Cd^2+^) complexes. While the bond-length distributions of ^[6]^Zn^2+^ and ^[6]^Cd^2+^ are regular, the range of bond lengths for ^[6]^Zn^2+^ is much larger than that for ^[6]^Cd^2+^ (0.868 versus 0.591 Å), suggesting that the PJTE is stronger for Zn^2+^ than it is for Cd^2+^ when bonded to O^2−^. However, both these ions (and other *d* ^10^ transition metals) have been shown to exhibit the PJTE in S^2−^ structures of various *d* ^10^ transition metal ions (Boucher *et al.*, 1994 ▸; Fan *et al.*, 2017 ▸); the extent to which the PJTE affects bond lengths for *d* ^10^ transition metal oxyanions awaits further work.

For the five ion configurations with non-*d* ^0^ electronic configuration whose main causal mechanism for bond-length variation is the PJTE, 〈Δ_cryst_〉 values are 0.084 v.u. (*n* = 13) for ^[5]^Cu^2+^, 0.077 v.u. for ^[5]^Cr^2+^ (*n* = 2), 0.07 v.u. for ^[5]^Co^2+^ (*n* = 1), 0.050 v.u. for ^[6]^Zn^2+^ (*n* = 5) and 0.038 v.u. for ^[6]^Hg^2+^ (*n* = 2). Thus, it seems that the distorting power of non-*d* ^0^ ion configurations is slightly less than that of their *d* ^0^ counterparts, although the sample size is far from significant.

### 6.5. Comparing bond-length variation and distorting power across ligand type   

Comparing the magnitude of bond-length variations across ligand type is clouded by ligand size differences (*e.g.* the ionic radius of S^2−^ is larger than that of O^2−^ by roughly 0.5 Å; Shannon, 1976 ▸) and the scaling of these differences. Ligand size differences become particularly problematic when dealing with cations bonded to more than one ligand type, *e.g.* NbO_6_ versus NbO_4_S_2_ polyhedra. For example, in Sm^3+^3Nb^5+^O_4_S_3_ (Boyer-Candalen *et al.* 2000 ▸), Nb^5+^ makes four bonds to O^2−^ (1.889, 1.904, 1.929 and 2.101 Å) and two bonds to S^2−^ (2.586 and 2.719 Å). Is this polyhedron distorted, or is the bond-length variation simply due to ligand size differences? Can we quantitively resolve the distortion power of this PJTE-active cation in this configuration?

A great advantage of the Δ_topol_ and Δ_cryst_ indices is that they are independent of cation/anion type. In Table 11 ▸, we give the *a priori* bond valences for the Sm^3+^3Nb^5+^O_4_S_3_ structure, with observed bond valences in parentheses for Nb^5+^ (Nb^5+^–S^2−^ bond-valence parameters are those of the authors; article in preparation). Thus, we calculate Δ_topol_ = 0.049 and Δ_cryst_ = 0.148 v.u. for Nb^5+^, indicating that the distorting power of the PJTE is approximately the same here as it is for the average NbO_6_ polyhedron (0.167 v.u.; Table S2). As such, the introduction of S^2−^ seemingly has little effect on the distorting power of Nb^5+^, a result whose generalization may bear important consequences for the design of hetero-ligand materials.

### 6.6. Optimizing material properties linked to bond-length variation   

Calculation of the Δ_topol_ and Δ_cryst_ indices allows one to pinpoint the causal mechanism(s) underlying material properties linked to bond-length variations, and to determine *if*, and *how*, optimization of these properties may be done. Understanding the extent to which these mechanisms mater­ialize into bond-length variations is further crucial to maximize the harnessing of these effects within the constraints of physically realistic crystal structures.

For instance, seeking to optimize functional properties associated with non-centrosymmetric behaviour via compositional variations will have little effect if those properties are associated with coordination polyhedra where Δ_topol_ >> Δ_cryst_, as said property would arise primarily from the bond-topological underpinnings of the crystal structure. Along those lines, optimization via compositional variation would have maximum potential where the coordination unit responsible for the functional property has Δ_topol_ = 0. For example, Δ_topol_ = 0.000 and Δ_cryst_ = 0.017 v.u for the *B* site of the *Pnma* perovskite *^A^*Ca*^B^*Ti^4+^O_3_ (ICSD 74212; Liu & Liebermann, 1993 ▸). Introducing compositional variation, Δ_topol_ = 0.039 and Δ_cryst_ = 0.186 v.u. for the *B* site of the *Pnma* perovskite *^A^*Gd^3+*B*^Mn^3+^O_3_ (ICSD 95493; Mori *et al.*, 2002 ▸), leading to multiferroic behaviour (Li *et al.*, 2014 ▸).

Values given in this work may be used to set expectation limits regarding the maximum values of Δ_cryst_ attainable on the basis of ion configuration (Table S2) or coordination number (*e.g.* Table 10 ▸ for the PJTE), although we note that larger values of Δ_topol_ and Δ_cryst_ will probably be observed in the future; as such, Table S2 should be used as an evolving guide. In addition, minimum/maximum bond lengths listed in Table 1 ▸ are useful for framing marginal compositional substitutions within the realm of physically realistic crystal structures, and this dataset also has the advantage of being more exhaustive than our list of Δ_topol_ and Δ_cryst_ values (Table S2). Such compositional variation analyses may be complemented by DFT calculations, where constrained to values outlined in Tables 1 ▸ and S2. Going back to Gd^3+^Mn^3+^O_3_, with Δ_cryst_ = 0.186 v.u., consulting Table S2 informs us that little optimization of the functional properties resulting from Mn^3+^ seems possible; Table S2 lists three values of Δ_cryst_ in as many crystal structures for ^[6]^Mn^3+^: 0.186, 0.211 and 0.211 v.u. In this instance, increasing Δ_cryst_ would necessarily follow from substitution of *^A^*Gd^3+^, whereby a small amount of bond-length variation via mechanism (4) (crystal-structure effects) may trickle down to the Δ_cryst_ index of *^B^*Mn^3+^.

In the reverse scenario, where the functional property is linked to non-local bond-topological asymmetry (*i.e.* Δ_topol_ > Δ_cryst_), optimization is complicated by the fact that spontaneous distortion of crystal structures via non-local bond-topological asymmetry is a static emergent phenomenon which can only be predicted from *a priori* knowledge of ion connectivity. As a result, properties arising from non-local bond-topological asymmetry are less tunable, for they require subtle and less predictable changes in ion connectivity. These changes may occur either at the site of interest (*e.g.* substituting for a cation of significantly different size, which may change the coordination number of the site) or at other sites in the structure, where subtle changes in coordination number via homovalent and/or (multi-site) heterovalent substitution may have an effect on the *a priori* bond valences of the crystal structure which carries through to the Δ_topol_ index of the (functional) site of interest. While these substitutions can be modelled with relative ease, a significant constraint is that such fine tuning must not result in the crystallization of a different structure type. As a result, the optimization of functional properties linked to non-local bond-topological asymmetry is significantly more challenging than that for crystallographic effects.

We further point out that it is not uncommon for the mechanisms underlying the Δ_topol_ and Δ_cryst_ indices to work together toward the expression or suppression of a functional property, depending on their relative spatial expression within the polyhedron and unit cell. In such cases, calculation of Δ_topol_ and Δ_cryst_ values is useful for resolving the anomalous magnitude of functional properties. For example, for *P*31*m* K_3_V^5+^_5_O_14_ (ICSD 248227; Yeon *et al.*, 2010 ▸), *a priori* (observed bond valences are 1.430 (1.601) and 3 × 1.190 (1.169) v.u., with Δ_topol_ = 0.090 and Δ_cryst_ = 0.059 v.u for V1, and 1.220 (1.647), 2 × 0.980 (0.938) and 2 × 0.910 (0.738) v.u., with Δ_topol_ = 0.088 and Δ_cryst_ = 0.171 v.u for V2. For V1, non-local bond-topological asymmetry is mainly responsible for variation away from a regular tetrahedron with four bonds of 1.25 v.u.; for V2, the PJTE is the principal reason for variation away from observing five bonds of 1 v.u. More importantly, both mechanisms cause variation in the same spatial direction within both their polyhedron and the unit cell; both strongest bonds (V1—O1 and V2—O2) point in the same direction, along the *c* axis. As a result, the effect of Δ_topol_ and Δ_cryst_ is entirely additive, and K_3_V^5+^_5_O_14_ is observed with a series of marked functional properties, *i.e.* second-harmonic generation, piezoelectricity and polarization (Yeon *et al.*, 2010 ▸).

## 7. Conclusions   

In this work, we have resolved the causal mechanisms of bond-length variation for transition metals in oxide and oxysalt structures, and further quantified the extent to which these mechanisms result in bond-length variation for transition metal configurations with anomalous bond-length distributions.

One of the principal findings presented in this work regards the unrealized extent to which crystal structures spontaneously distort as a result of non-local bond-topological asymmetry – a mechanism we have shown to be entirely separate from and independent of electronic and crystal-structure effects. The demonstrated ubiquity of this phenomenon, as well as the magnitude of the bond-valence variations it generates, challenge the common assumption of bond-length transferability in solids. This finding further conflicts with the widespread approximation of bond lengths via the addition of constituent ionic radii, and provides quantitative evidence of the ‘non-spherical’ nature of coordination environments. While the addition of ionic radii certainly remains a useful approximation of bond lengths for yet-to-be-observed ion pairs, it has become evident that the practice should be avoided where comprehensive bond-length statistics are available (such as those given in this work, and throughout this series).

Perhaps one of the most promising opportunities resulting from this work regards the strategic use of the newly proposed Δ_topol_ and Δ_cryst_ indices for the optimization of functional properties tied to bond-length variations. Calculation of these indices allows identification of the causal mechanism(s) upon which optimization should be focused, while the magnitude of these values is used to gauge qualitatively the extent to which these values (typically Δ_cryst_) may be maximized. Along those lines, examination of the relation between Δ_topol_ and Δ_cryst_ and the magnitude of various functional properties linked to bond-length variation, and the optimization of these properties, seems warranted.

## Supplementary Material

Additional figures and tables. DOI: 10.1107/S2052252520005928/lt5028sup1.pdf

Raw data bond-length files. DOI: 10.1107/S2052252520005928/lt5028sup2.zip

## Figures and Tables

**Figure 9:**
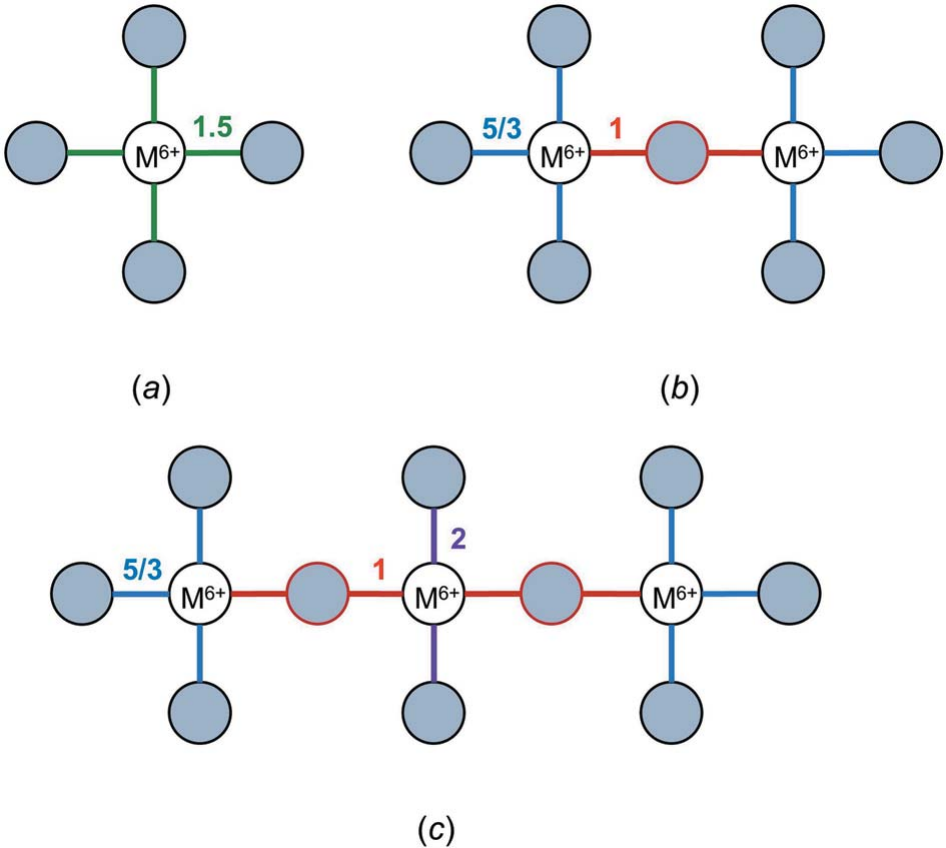
Bond-valence patterns arising from various corner-sharing tetrahedra for the +6 oxidation state for (*a*) monomers, (*b*) dimers and (*c*) linear oligomers and chains. Shaded spheres represent O^2−^.

**Table 1:** Bond-length statistics for the transition metal ions bonded to O^2−^

Ion	Coordination number	Number of bonds	Number of coordination polyhedra	Mean bond length (Å)	Standard deviation (Å)	Range (Å)	Maximum bond length (Å)	Minimum bond length (Å)	Skewness	Kurtosis
Sc^3+^	6	450	75	2.098	0.041	0.236	2.231	1.995	0.429	0.610
	7	35	5	2.163	0.056	0.268	2.315	2.047	−0.242	0.731
	8	64	8	2.234	0.101	0.497	2.554	2.057	1.352	2.513
Ti^3+^	6	126	21	2.037	0.051	0.263	2.167	1.904	−0.115	0.160
	7	14	2	2.108	0.022	0.077	2.134	2.057	−1.113	0.575
	8	8	1	2.195	0.077	0.153	2.271	2.118	0.000	−2.800
Ti^4+^	4	16	4	1.821	0.038	0.159	1.906	1.747	0.220	1.124
	5	85	17	1.917	0.106	0.405	2.050	1.645	−1.256	0.139
	6	1758	293	1.971	0.107	0.826	2.474	1.648	0.124	1.531
	7	7	1	2.064	0.165	0.550	2.230	1.680	−2.098	5.088
V^3+^	6	402	67	2.007	0.051	0.339	2.224	1.885	0.479	0.681
V^4+^	5	475	95	1.893	0.147	0.577	2.116	1.539	−1.348	0.163
	6	768	128	1.980	0.202	1.030	2.588	1.558	−0.173	0.559
V^5+^	4	1380	345	1.717	0.056	0.354	1.917	1.563	0.300	−0.250
	5	325	65	1.827	0.147	0.801	2.352	1.551	−0.068	−0.607
	6	1758	293	1.924	0.213	0.993	2.547	1.554	0.324	−0.519
Cr^2+^	4	24	6	2.004	0.010	0.028	2.025	1.997	1.441	0.378
	5	10	2	2.113	0.144	0.432	2.426	1.994	1.637	1.313
	6	54	9	2.188	0.193	0.696	2.651	1.955	0.809	−0.889
Cr^3+^	6	624	104	1.976	0.026	0.190	2.074	1.884	0.277	1.855
Cr^4+^	4	4	1	1.784	0.035	0.086	1.844	1.758	1.825	3.332
	6	36	6	1.950	0.032	0.094	1.988	1.894	−0.362	−1.336
Cr^5+^	4	4	1	1.693	0.006	0.012	1.699	1.687	0.000	−6.000
Cr^6+^	4	676	169	1.652	0.059	0.357	1.892	1.535	1.593	2.852
Mn^2+^	4	40	10	2.046	0.039	0.241	2.194	1.953	1.304	4.668
	5	120	24	2.141	0.053	0.243	2.267	2.024	0.264	−0.341
	6	1908	318	2.199	0.085	0.830	2.798	1.968	2.070	7.951
	7	14	2	2.352	0.240	0.663	2.782	2.119	0.756	−1.323
	8	144	18	2.321	0.104	0.539	2.691	2.152	0.899	0.927
Mn^3+^	4	8	2	1.901	0.046	0.099	1.951	1.852	0.015	−2.750
	5	50	10	1.959	0.075	0.255	2.109	1.854	0.301	−1.091
	6	492	82	2.031	0.149	0.755	2.598	1.843	0.841	−0.500
Mn^4+^	4	4	1	1.750	0.000	0.000	1.750	1.750	−	−
	6	120	20	1.903	0.030	0.167	2.008	1.841	0.552	1.613
Mn^5+^	4	32	8	1.698	0.014	0.076	1.725	1.649	−1.042	3.241
Mn^6+^	4	8	2	1.662	0.012	0.034	1.687	1.653	1.599	1.380
Mn^7+^	4	28	7	1.610	0.009	0.041	1.633	1.592	0.868	1.384
Fe^2+^	3	24	8	1.844	0.029	0.127	1.918	1.791	0.578	0.456
	4	24	6	1.985	0.027	0.141	2.041	1.900	−1.051	0.726
	5	95	19	2.097	0.099	0.572	2.493	1.921	1.370	3.133
	6	876	146	2.147	0.089	0.713	2.646	1.933	1.440	4.840
	8	40	5	2.333	0.188	0.684	2.722	2.038	0.598	−0.400
Fe^3+^	4	260	65	1.875	0.033	0.201	1.965	1.764	−0.060	0.471
	5	105	21	1.966	0.070	0.350	2.207	1.857	1.245	2.428
	6	2268	378	2.015	0.064	0.539	2.391	1.852	0.971	2.078
	8	16	2	2.125	0.029	0.095	2.173	2.078	0.036	−1.333
Co^2+^	3	42	14	1.854	0.058	0.223	1.980	1.757	0.457	−0.754
	4	108	27	1.967	0.022	0.189	2.081	1.892	0.502	5.715
	5	80	16	2.066	0.117	0.628	2.574	1.946	2.717	7.945
	6	1458	243	2.108	0.062	0.571	2.516	1.945	1.612	5.825
	8	8	1	2.272	0.185	0.517	2.573	2.056	0.203	−1.481
Co^3+^	6	90	15	1.908	0.021	0.108	1.969	1.861	0.717	1.335
Co^4+^	6	6	1	1.874	0.000	0.000	1.874	1.874	−1.369	−3.333
Ni^2+^	2	4	2	1.686	0.001	0.002	1.687	1.685	0.000	−6.000
	4	12	3	1.950	0.039	0.092	1.982	1.890	−0.800	−1.573
	5	40	8	2.028	0.041	0.186	2.149	1.963	0.597	0.354
	6	1452	242	2.070	0.054	0.589	2.462	1.873	0.874	4.880
Ni^4+^	6	30	5	1.870	0.012	0.067	1.906	1.839	0.446	3.019
Cu^+^	2	84	42	1.839	0.024	0.123	1.911	1.788	−0.080	−0.134
	3	6	2	1.969	0.076	0.183	2.077	1.894	0.742	−1.897
	4	52	13	2.084	0.110	0.541	2.438	1.897	0.554	0.966
Cu^2+^	4	516	129	1.943	0.029	0.180	2.055	1.875	0.910	2.172
	5	1090	218	2.037	0.155	0.865	2.700	1.835	1.784	2.494
	6	2190	365	2.130	0.232	0.893	2.748	1.855	0.969	−0.494
	8	32	4	2.302	0.304	0.794	2.743	1.949	0.052	−1.973
Cu^3+^	4	44	11	1.850	0.029	0.135	1.946	1.811	1.485	3.710
Zn^2+^	4	908	227	1.952	0.031	0.229	2.076	1.847	0.331	0.887
	5	180	36	2.051	0.082	0.619	2.525	1.906	1.609	5.499
	6	1158	193	2.110	0.086	0.810	2.696	1.886	2.005	6.961
Y^3+^	6	150	25	2.264	0.041	0.226	2.400	2.174	0.695	1.020
	7	245	35	2.332	0.082	0.499	2.661	2.162	1.248	2.181
	8	800	99	2.390	0.065	0.507	2.729	2.222	1.353	6.271
	9	135	15	2.422	0.092	0.585	2.799	2.214	1.005	3.144
	10	10	1	2.496	0.187	0.604	2.857	2.253	0.715	−0.598
	12	12	1	2.541	0.000	0.000	2.541	2.541	−	−
Zr^4+^	6	438	73	2.078	0.031	0.218	2.224	2.006	1.291	3.830
	7	91	13	2.146	0.048	0.233	2.283	2.050	0.000	−0.356
	8	168	21	2.199	0.053	0.313	2.407	2.094	0.536	0.471
	9	27	3	2.263	0.138	0.472	2.593	2.121	1.385	1.474
	10	70	7	2.283	0.046	0.204	2.415	2.211	1.150	0.597
Nb^4+^	6	18	3	2.054	0.080	0.241	2.163	1.922	−0.125	−1.553
Nb^5+^	4	8	2	1.831	0.068	0.184	1.926	1.742	0.167	−1.224
	5	20	4	1.926	0.054	0.164	1.993	1.829	−0.467	−1.286
	6	1440	240	1.993	0.115	0.742	2.444	1.702	0.646	1.077
	7	21	3	2.069	0.163	0.668	2.435	1.767	0.469	0.090
	8	8	1	2.080	0.001	0.002	2.081	2.079	0.000	−2.800
Mo^3+^	6	30	5	2.095	0.024	0.112	2.163	2.051	0.347	1.099
Mo^4+^	6	54	9	2.003	0.065	0.264	2.077	1.813	−1.750	2.047
Mo^5+^	5	10	2	1.916	0.122	0.328	1.981	1.653	−1.799	1.552
	6	444	74	1.992	0.167	0.806	2.429	1.623	−0.627	0.085
Mo^6+^	4	1736	434	1.764	0.033	0.268	1.905	1.637	0.520	0.770
	5	75	15	1.872	0.146	0.620	2.286	1.666	0.679	−0.042
	6	3036	506	1.972	0.232	1.005	2.608	1.603	0.431	−1.027
Tc^7+^	4	24	6	1.705	0.019	0.068	1.740	1.672	−0.044	−0.591
Ru^3+^	6	18	3	2.025	0.043	0.191	2.107	1.916	−1.010	2.152
Ru^4+^	6	48	8	1.982	0.025	0.134	2.070	1.936	1.317	5.102
Ru^5+^	6	138	23	1.964	0.076	0.273	2.113	1.840	0.384	−0.755
Rh^3+^	6	66	11	2.025	0.023	0.093	2.071	1.978	0.647	−0.607
Rh^4+^	6	18	3	2.007	0.014	0.032	2.020	1.988	−0.442	−1.946
Pd^2+^	4	116	29	2.011	0.024	0.104	2.060	1.956	0.347	−0.596
Pd^4+^	6	12	2	2.000	0.027	0.094	2.059	1.965	0.959	0.035
Ag^+^	2	10	5	2.136	0.018	0.053	2.164	2.111	0.435	−1.386
	3	45	15	2.278	0.112	0.391	2.535	2.144	0.888	−0.181
	4	148	37	2.402	0.127	0.601	2.741	2.140	−0.057	−0.002
	5	225	45	2.489	0.152	0.801	2.975	2.174	0.657	0.058
	6	378	63	2.537	0.135	0.652	2.894	2.242	0.330	−0.288
	7	63	9	2.589	0.155	0.564	2.888	2.324	0.308	−0.888
	8	104	13	2.656	0.175	0.708	3.083	2.375	0.736	0.205
	9	27	3	2.704	0.143	0.402	2.863	2.461	−0.894	−0.873
Cd^2+^	5	20	4	2.257	0.078	0.342	2.486	2.144	1.352	2.401
	6	810	135	2.302	0.069	0.591	2.754	2.163	1.472	4.484
	7	42	6	2.377	0.134	0.713	2.888	2.175	2.033	5.213
	8	144	18	2.432	0.118	0.531	2.724	2.193	0.312	−0.575
	9	9	1	2.530	0.214	0.500	2.826	2.326	0.688	−1.714
Hf^4+^	6	66	11	2.082	0.051	0.322	2.241	1.919	0.195	4.551
	7	28	4	2.128	0.019	0.072	2.167	2.095	0.016	−0.842
	8	56	7	2.190	0.064	0.256	2.324	2.068	−0.012	−0.840
Ta^5+^	6	828	138	1.988	0.076	0.585	2.386	1.801	1.474	4.235
	7	98	14	2.057	0.155	0.619	2.486	1.867	1.763	2.077
W^5+^	6	24	4	1.956	0.095	0.448	2.140	1.692	−0.821	1.154
W^6+^	4	140	35	1.773	0.027	0.141	1.846	1.705	−0.220	0.095
	5	60	12	1.859	0.072	0.467	2.166	1.699	0.885	4.719
	6	2178	363	1.951	0.182	0.919	2.557	1.638	0.750	−0.146
Re^5+^	6	18	3	1.940	0.068	0.183	2.027	1.844	0.045	−1.764
Re^7+^	4	164	41	1.716	0.021	0.150	1.790	1.640	−0.114	1.066
	5	40	8	1.810	0.052	0.275	1.904	1.629	−1.249	3.058
	6	60	10	1.882	0.046	0.195	1.982	1.787	0.380	−0.224
Os^5+^	6	24	4	1.960	0.044	0.180	2.044	1.864	−0.486	0.512
Os^6+^	6	6	1	1.926	0.125	0.266	2.015	1.749	−0.968	−1.875
Os^7+^	5	5	1	1.825	0.034	0.092	1.855	1.763	−1.448	2.287
	6	18	3	1.887	0.021	0.058	1.923	1.865	0.864	−0.668
Os^8+^	4	4	1	1.698	0.014	0.027	1.711	1.684	0.000	−6.000
	5	15	3	1.793	0.162	0.569	2.227	1.658	2.132	3.720
	6	24	4	1.880	0.170	0.442	2.169	1.727	0.747	−1.167
Ir^3+^	6	6	1	2.042	0.000	0.000	2.042	2.042	−	−
Ir^4+^	4	20	5	1.909	0.008	0.032	1.929	1.897	1.151	1.194
	6	72	12	2.015	0.024	0.176	2.096	1.920	−0.698	4.820
Ir^5+^	6	36	6	1.990	0.013	0.039	2.010	1.971	−0.237	−1.358
Pt^2+^	4	12	3	2.007	0.009	0.022	2.017	1.995	−0.473	−1.650
Pt^4+^	6	198	33	2.021	0.020	0.142	2.087	1.945	0.222	2.499
Au^3+^	4	96	24	1.999	0.023	0.153	2.082	1.929	0.879	2.577
Hg^2+^	2	2	1	1.955	0.000	0.000	1.955	1.955	−	−
	4	24	6	2.316	0.311	0.862	2.834	1.972	0.427	−1.568
	5	15	3	2.380	0.271	0.688	2.726	2.038	−0.031	−1.928
	6	150	25	2.429	0.249	0.912	2.932	2.020	−0.041	−1.051
	7	63	9	2.505	0.267	0.998	2.988	1.990	−0.409	−1.095
	8	64	8	2.502	0.163	0.685	2.906	2.221	0.493	−0.182

**Table 2:** Minimum sample size required to satisfy given variability thresholds for bond-length distributions (mean bond-length distributions) Ion configurations with multi-modal bond-length distributions (here caused by lone-pair stereoactivity) are shown in bold font.

	Sample size (parent population)	Mean bond valence (v.u.)	Mean bond length (±0.005 Å)	Skewness (±0.2) and kurtosis (±0.6)	Reference
^[6]^Na^+^	920	0.17	200	225 (400)	Gagné & Hawthorne (2016 ▸)
**^[8]^Bi^3+^**	**84**	**0.38**	**70**	**7** (**60**)	Gagné & Hawthorne (2018*a* ▸)
^[8]^La^3+^	78	0.38	20	30 (60)	Gagné (2018 ▸)
^[6]^Ti^4+^	298	0.67	20	115 (130)	This work
**^[6]^I^5+^**	**77**	**0.83**	**40**	**2** (**50**)	Gagné & Hawthorne (2018*b* ▸)
^[4]^Si^4+^	2506	1	25	70 (400)	Gagné & Hawthorne (2018*a* ▸)
^[4]^S^6+^	906	1.5	5	300 (700)	Gagné & Hawthorne (2018*b* ▸)

**Table 3:** Mean bond-length statistics for the transition metal ions bonded to O^2−^

Ion	Coordination number	Number of coordination polyhedra	Grand mean bond length (Å)	Standard deviation (Å)	Mean bond-length range (Å)	Maximum mean bond length (Å)	Minimum mean bond length (Å)	Skewness	Kurtosis
Sc^3+^	6	75	2.098	0.017	0.071	2.133	2.063	0.074	−0.586
	7	5	2.163	0.017	0.043	2.188	2.146	0.846	−0.505
	8	8	2.234	0.026	0.066	2.273	2.207	0.622	−1.817
Ti^3+^	6	21	2.037	0.019	0.081	2.072	1.991	−0.666	1.075
	7	2	2.108	0.008	0.012	2.114	2.102	–	–
	8	1	2.195	–	0.000	2.195	2.195	–	–
Ti^4+^	4	4	1.821	0.011	0.026	1.836	1.811	1.416	2.442
	5	17	1.917	0.014	0.048	1.940	1.892	0.241	−0.518
	6	293	1.971	0.017	0.094	2.020	1.926	−0.209	0.118
	7	1	2.064	–	0.000	2.064	2.064	–	–
V^3+^	6	67	2.007	0.017	0.079	2.048	1.968	0.084	−0.235
V^4+^	5	95	1.893	0.010	0.066	1.929	1.863	0.411	1.187
	6	128	1.980	0.019	0.103	2.023	1.921	−0.323	0.130
V^5+^	4	345	1.717	0.010	0.075	1.758	1.682	0.075	1.991
	5	65	1.827	0.013	0.073	1.869	1.796	0.569	1.397
	6	293	1.924	0.013	0.079	1.976	1.896	1.045	1.486
Cr^2+^	4	6	2.004	0.010	0.027	2.024	1.997	2.082	4.523
	5	2	2.113	0.004	0.006	2.116	2.110	–	–
	6	9	2.188	0.020	0.067	2.233	2.165	1.644	3.308
Cr^3+^	6	104	1.976	0.014	0.064	2.013	1.949	0.409	−0.583
Cr^4+^	4	1	1.784	–	0.000	1.784	1.784	–	–
	6	6	1.950	0.019	0.052	1.988	1.937	2.130	4.777
Cr^5+^	4	1	1.693	–	0.000	1.693	1.693	–	–
Cr^6+^	4	169	1.652	0.011	0.072	1.696	1.624	0.295	1.051
Mn^2+^	4	10	2.046	0.025	0.085	2.085	2.001	−0.040	−0.021
	5	24	2.141	0.023	0.096	2.175	2.079	−0.830	1.283
	6	318	2.199	0.027	0.172	2.305	2.134	0.434	0.597
	7	2	2.352	0.061	0.087	2.395	2.309	–	–
	8	18	2.321	0.025	0.081	2.356	2.275	−0.415	−0.636
Mn^3+^	4	2	1.901	0.003	0.005	1.903	1.898	–	–
	5	10	1.959	0.017	0.047	1.980	1.933	−0.240	−1.269
	6	82	2.031	0.019	0.094	2.086	1.992	0.106	−0.320
Mn^4+^	4	1	1.750	–	0.000	1.750	1.750	–	–
	6	20	1.903	0.012	0.043	1.928	1.885	0.202	−0.364
Mn^5+^	4	8	1.698	0.007	0.023	1.706	1.683	−1.095	1.556
Mn^6+^	4	2	1.662	0.008	0.015	1.670	1.655	–	–
Mn^7+^	4	7	1.610	0.007	0.020	1.622	1.603	0.913	0.421
Fe^2+^	3	8	1.844	0.018	0.049	1.860	1.811	−1.274	0.568
	4	6	1.985	0.021	0.066	2.008	1.942	−1.667	3.509
	5	19	2.097	0.027	0.117	2.142	2.024	−1.036	1.866
	6	146	2.147	0.028	0.156	2.246	2.090	0.543	0.419
	8	5	2.333	0.041	0.087	2.383	2.296	0.519	−2.945
Fe^3+^	4	65	1.875	0.016	0.070	1.910	1.840	0.086	−0.642
	5	21	1.966	0.015	0.049	1.984	1.935	−0.717	−0.491
	6	378	2.015	0.019	0.114	2.091	1.977	1.123	2.171
	8	2	2.125	0.008	0.011	2.130	2.119	–	–
Co^2+^	3	14	1.854	0.017	0.055	1.869	1.814	−1.659	1.756
	4	27	1.967	0.017	0.069	1.995	1.926	−0.648	0.341
	5	16	2.066	0.026	0.101	2.129	2.027	0.557	0.775
	6	243	2.108	0.021	0.121	2.182	2.061	0.678	0.947
	8	1	2.272	–	0.000	2.272	2.272	–	–
Co^3+^	6	15	1.908	0.013	0.049	1.942	1.893	1.575	2.638
Co^4+^	6	1	1.874	–	0.000	1.874	1.874	–	–
Ni^2+^	2	2	1.686	0.001	0.002	1.687	1.685	–	–
	4	3	1.950	0.047	0.082	1.977	1.895	−1.732	–
	5	8	2.028	0.012	0.031	2.044	2.013	0.089	−1.701
	6	242	2.070	0.020	0.118	2.148	2.030	0.900	1.669
Ni^4+^	6	5	1.870	0.004	0.011	1.875	1.865	0.408	1.817
Cu^+^	2	42	1.839	0.022	0.087	1.882	1.794	−0.286	−0.503
	3	2	1.969	0.002	0.003	1.971	1.968	–	–
	4	13	2.084	0.042	0.137	2.171	2.034	0.619	−0.190
Cu^2+^	4	129	1.943	0.017	0.085	1.986	1.901	0.285	0.160
	5	218	2.037	0.022	0.122	2.106	1.984	0.534	0.244
	6	365	2.130	0.030	0.174	2.225	2.051	0.678	0.322
	8	4	2.302	0.033	0.069	2.349	2.280	1.689	2.751
Cu^3+^	4	11	1.846	0.018	0.055	1.872	1.817	0.070	−1.052
Zn^2+^	4	227	1.952	0.016	0.115	2.020	1.905	0.166	1.334
	5	36	2.051	0.015	0.073	2.085	2.012	−0.304	0.138
	6	193	2.110	0.023	0.144	2.200	2.056	1.050	1.676
Y^3+^	6	25	2.264	0.019	0.069	2.291	2.222	−0.208	−0.894
	7	35	2.332	0.018	0.065	2.366	2.302	0.002	−0.708
	8	99	2.390	0.019	0.096	2.438	2.342	−0.690	0.473
	9	15	2.422	0.019	0.071	2.468	2.397	1.140	1.142
	10	1	2.496	–	0.000	2.496	2.496	–	–
	12	1	2.541	–	0.000	2.541	2.541	–	–
Zr^4+^	6	73	2.078	0.013	0.082	2.129	2.048	1.057	3.431
	7	13	2.146	0.012	0.032	2.162	2.129	0.187	−1.758
	8	21	2.199	0.006	0.022	2.211	2.189	−0.219	−0.686
	9	3	2.263	0.019	0.036	2.278	2.242	−1.132	–
	10	7	2.283	0.005	0.011	2.288	2.277	−0.147	−2.054
Nb^4+^	6	3	2.054	0.006	0.011	2.061	2.049	1.449	–
Nb^5+^	4	2	1.831	0.005	0.007	1.834	1.827	–	–
	5	4	1.926	0.011	0.025	1.933	1.907	−1.982	3.938
	6	240	1.993	0.018	0.102	2.046	1.944	0.137	0.150
	7	3	2.069	0.013	0.026	2.082	2.056	−0.217	–
	8	1	2.080	–	0.000	2.080	2.080	–	–
Mo^3+^	6	5	2.095	0.009	0.023	2.109	2.086	1.468	2.769
Mo^4+^	6	9	2.003	0.007	0.023	2.016	1.992	0.559	0.323
Mo^5+^	5	2	1.916	0.004	0.009	1.920	1.911	–	–
	6	74	1.992	0.015	0.070	2.026	1.955	−0.034	−0.410
Mo^6+^	4	434	1.764	0.009	0.057	1.790	1.732	−0.255	0.861
	5	15	1.872	0.016	0.053	1.898	1.845	0.069	−0.882
	6	506	1.972	0.015	0.091	2.024	1.933	0.096	−0.282
Tc^7+^	4	6	1.705	0.004	0.009	1.711	1.702	0.672	−1.320
Ru^3+^	6	3	2.025	0.002	0.003	2.027	2.024	–	–
Ru^4+^	6	8	1.982	0.011	0.034	1.995	1.961	−0.713	−0.066
Ru^5+^	6	23	1.964	0.013	0.044	1.987	1.943	0.241	−1.333
Rh^3+^	6	11	2.025	0.021	0.065	2.071	2.006	1.324	0.754
Rh^4+^	6	3	2.007	0.011	0.020	2.020	2.000	1.705	–
Pd^2+^	4	29	2.011	0.020	0.078	2.060	1.982	0.578	−0.419
Pd^4+^	6	2	1.999	0.004	0.005	2.001	1.996	–	–
Ag^+^	2	5	2.136	0.009	0.043	2.164	2.121	0.862	−1.700
	3	15	2.278	0.025	0.083	2.319	2.236	0.003	−0.865
	4	37	2.402	0.030	0.125	2.466	2.341	−0.180	−0.607
	5	45	2.489	0.036	0.147	2.555	2.408	−0.190	−0.438
	6	63	2.537	0.031	0.136	2.594	2.458	−0.292	−0.539
	7	9	2.589	0.028	0.084	2.618	2.534	−1.001	0.480
	8	13	2.656	0.030	0.113	2.730	2.617	1.199	1.882
	9	3	2.704	0.024	0.041	2.731	2.690	1.732	–
Cd^2+^	5	4	2.257	0.014	0.034	2.278	2.244	1.291	2.291
	6	135	2.302	0.021	0.108	2.368	2.260	0.618	0.035
	7	6	2.377	0.047	0.132	2.466	2.334	1.758	3.604
	8	18	2.432	0.027	0.086	2.469	2.383	−0.105	−1.040
	9	1	2.530	–	0.000	2.530	2.530	–	–
Hf^4+^	6	11	2.082	0.013	0.035	2.099	2.064	0.085	−1.193
	7	4	2.128	0.003	0.006	2.131	2.125	−0.017	−5.552
	8	7	2.190	0.004	0.013	2.199	2.186	1.365	2.285
Ta^5+^	6	138	1.988	0.018	0.100	2.052	1.951	0.477	0.131
	7	14	2.057	0.007	0.023	2.069	2.046	0.459	−0.831
W^5+^	6	4	1.956	0.019	0.043	1.976	1.933	−0.225	−2.734
W^6+^	4	35	1.773	0.012	0.050	1.797	1.747	−0.172	0.581
	5	12	1.859	0.013	0.040	1.879	1.839	0.133	−1.593
	6	363	1.951	0.017	0.100	1.995	1.894	−0.455	0.408
Re^5+^	6	3	1.940	0.005	0.009	1.945	1.936	1.597	–
Re^7+^	4	41	1.716	0.011	0.053	1.740	1.686	−0.443	0.444
	5	8	1.810	0.010	0.035	1.827	1.792	−0.155	1.651
	6	10	1.882	0.012	0.037	1.906	1.869	0.909	0.092
Os^5+^	6	4	1.960	0.004	0.008	1.963	1.954	−1.846	3.508
Os^6+^	6	1	1.926	–	0.000	1.926	1.926	–	–
Os^7+^	5	1	1.825	–	0.000	1.825	1.825	–	–
	6	3	1.887	0.014	0.034	1.904	1.870	–	–
Os^8+^	4	1	1.698	–	0.000	1.698	1.698	–	–
	5	3	1.793	0.020	0.039	1.811	1.772	−0.726	–
	6	4	1.885	0.018	0.040	1.902	1.862	−0.798	−0.968
Ir^3+^	6	1	2.042	–	0.000	2.042	2.042	–	–
Ir^4+^	4	5	1.909	0.007	0.017	1.919	1.902	0.448	−0.638
	6	12	2.015	0.010	0.034	2.031	1.997	−0.353	−0.043
Ir^5+^	6	6	1.990	0.013	0.030	2.001	1.971	−0.928	−1.725
Pt^2+^	4	2	2.007	0.011	0.022	2.017	1.995	−1.008	–
Pt^4+^	6	33	2.021	0.014	0.057	2.055	1.998	0.345	−0.452
Au^3+^	4	24	1.999	0.013	0.040	2.020	1.980	0.161	−1.363
Hg^2+^	2	1	1.955	–	0.000	1.955	1.955	–	–
	4	6	2.316	0.081	0.227	2.403	2.176	−1.116	1.176
	5	3	2.380	0.090	0.168	2.445	2.277	−1.565	–
	6	25	2.429	0.072	0.242	2.584	2.342	0.532	−0.474
	7	9	2.505	0.045	0.140	2.593	2.453	0.723	0.440
	8	8	2.502	0.021	0.050	2.530	2.479	0.435	−2.040

**Table 4:** Ion configurations with anomalous bond-length distribution when bonded to O^2−^ Mechanisms are listed in decreasing order of importance: BTE denotes bond-topological effects, π pi-bond formation, JTEs strong Jahn–Teller effect, JTEw weak Jahn–Teller effect, PJTE pseudo Jahn–Teller effect, CSE crystal-structure effects.

Ion configuration	Electronic configuration	Anomalous shape?	Sample size (No. of coordination polyhedra)	Bond-length range (Å)	Δ_topol_ > Δ_cryst_?	Mechanism(s)
^[6]^Sc^3+^	*d* ^0^	Yes	75	0.236	Yes	BTE, PJTE
^[4]^Ti^4+^	*d* ^0^		4	0.159		PJTE, BTE
^[5]^Ti^4+^	*d* ^0^	Yes	17	0.405		PJTE, BTE
^[6]^Ti^4+^	*d* ^0^	Yes	293	0.826		PJTE, BTE
^[7]^Ti^4+^	*d* ^0^	Yes	1	0.55	No data	PJTE
^[6]^V^3+^	*d* ^2^	Yes	67	0.399	Yes	BTE, JTEw
^[5]^V^4+^	*d* ^1^	Yes	95	0.577	Yes	π, PJTE/BTE/CSE
^[6]^V^4+^	*d* ^1^	Yes	128	1.030	Yes	π, PJTE/BTE
^[4]^V^5+^	*d* ^0^	Yes	355	0.391	Yes	BTE, PJTE
^[5]^V^5+^	*d* ^0^	Yes	65	0.801	Yes	π, PJTE, BTE
^[6]^V^5+^	*d* ^0^	Yes	293	0.993	Yes	π, BTE/PJTE
^[5]^Cr^2+^	*d* ^4^		2	0.432		PJTE, BTE
^[6]^Cr^2+^	*d* ^4^	Yes	9	0.696	Yes	JTEs/BTE
^[6]^Cr^3+^	*d* ^3^	Yes	104	0.19	Similar	BTE, CSE
^[4]^Cr^6+^	*d* ^0^	Yes	169	0.357	Yes	BTE, PJTE, π
^[6]^Mn^3+^	*d* ^4^	Yes	82	0.755		JTEs, BTE
^[4]^Fe^3+^	*d* ^5^	Yes	65	0.201	Yes	BTE
^[5]^Co^2+^	*d* ^7^		16	0.628		PJTE
^[6]^Co^2+^	*d* ^7^		243	0.614	Yes	BTE, JTEs
^[5]^Cu^2+^	*d* ^9^		218	0.865		PJTE, BTE
^[6]^Cu^2+^	*d* ^9^	Yes	365	0.893		JTEs, BTE
^[6]^Zn^2+^	*d* ^10^		193	0.868	Similar	BTE/PJTE
^[6]^Y^3+^	*d* ^0^	Yes	25	0.226	Similar	PJTE/BTE
^[7]^Y^3+^	*d* ^0^	Yes	35	0.499		PJTE
^[8]^Y^3+^	*d* ^0^	Yes	99	0.507		PJTE/BTE
^[9]^Y^3+^	*d* ^0^		15	0.585		PJTE, BTE
^[10]^Y^3+^	*d* ^0^		1	0.604	Yes	BTE/PJTE
^[6]^Zr^4+^	*d* ^0^	Yes	73	0.218		PJTE, BTE
^[4]^Nb^5+^	*d* ^0^		2	0.184	No data	PJTE/BTE
^[5]^Nb^5+^	*d* ^0^	Yes	4	0.164		PJTE/BTE
^[6]^Nb^5+^	*d* ^0^	Yes	240	0.742		PJTE, BTE
^[7]^Nb^5+^	*d* ^0^		3	0.668		PJTE, BTE
^[6]^Mo^4+^	*d* ^2^	Yes	9	0.264	Yes	BTE
^[5]^Mo^5+^	*d* ^1^	Yes	2	0.328	No data	π
^[6]^Mo^5+^	*d* ^1^	Yes	74	0.806	Yes	π, BTE, JTEw
^[4]^Mo^6+^	*d* ^0^	Possibly	434	0.268	Yes	BTE, PJTE
^[5]^Mo^6+^	*d* ^0^		15	0.620	Yes	π, BTE/PJTE
^[6]^Mo^6+^	*d* ^0^	Yes	506	1.005		PJTE, BTE
^[6]^Ru^5+^	*d* ^3^	Yes	23	0.305		CSE/BTE
^[6]^Cd^2+^	*d* ^10^		135	0.591	Yes	BTE, PJTE
^[6]^Hf^4+^	*d* ^0^	Possibly	11	0.322		PJTE, BTE
^[6]^Ta^5+^	*d* ^0^	Yes	138	0.585		PJTE, BTE
^[7]^Ta^5+^	*d* ^0^	Possibly	14	0.619		PJTE, BTE
^[5]^W^6+^	*d* ^0^		12	0.467		PJTE, BTE
^[6]^W^6+^	*d* ^0^	Yes	363	0.919		PJTE, BTE
^[4]^Re^7+^	*d* ^0^	Yes	41	0.150	Yes	BTE, PJTE
^[5]^Re^7+^	*d* ^0^	Possibly	8	0.275		PJTE
^[6]^Re^7+^	*d* ^0^		10	0.195	Yes	PJTE, BTE
^[6]^Os^6+^	*d* ^2^	Possibly	1	0.266	No data	BTE
^[5]^Os^8+^	*d* ^0^	Possibly	3	0.569		PJTE, BTE
^[6]^Os^8+^	*d* ^0^	Yes	4	0.442		PJTE, BTE
^[6]^Hg^2+^	*d* ^10^	Yes	25	0.912	Similar	BTE/PJTE

**Table 5:** Bond topology table for *^A^*Gd^3+*B*^Mn^3+^O_3_, showing numerical solution of its *a priori* bond valences (v.u.) and *a priori* bond lengths (in parentheses, in Å) Bonds are variables a to d, and arrows represent bond multiplicity.

	*A* (Gd^3+^)	*B* (Mn^3+^)	∑
*X*1 (O^2−^)	a ×2↓ ×2→	c ×2↓ ×2→	2
	0.441 (2.343)	0.559 (1.966)	
*X*2 (O^2−^)	b ×6↓×3	d ×4↓ ×2→	2
	0.353 (2.439)	0.471 (2.009)	
∑	3	3	

**Table 6:** *A priori* bond valences (v.u.) for CaNb^5+^_2_(P_4_O_13_)(P_2_O_7_)O

	Nb1	Nb2	Ca	P1	P2	P3	P4	∑
O1			0.267 ×2↓	1.733				2
O2		0.782 ×2↓		1.218				2
O3				1 ×2→				2
O4				1.049	0.951			2
O5	0.805 ×2↓				1.195			2
O6			0.316 ×2↓		1.684			2
O7		0.831 ×2↓			1.169			2
O8	0.837 ×2↓					1.163 ×2↓		2
O9			0.348			1.652		2
O10						1.169	0.978	2
O11	0.859 ×2↓						1.141 ×2↓	2
O12		0.261					1.739	2
O13		1.515	0.458					2
∑	5	5	2	5	5	5	5	

**Table 7:** *A priori* bond valences (v.u.) for Li_3_Nb^5+^O_4_ The subscript br denotes a bridging ion.

	Li	Nb	∑
O1_br_	−0.042 ×3→	0.708 ×3↓ ×3→	2
O2	0.208 ×5↓ ×5→	0.958 ×3↓	2
∑	1	5	

**Table 8:** *A priori* bond valences (v.u.) for PW^5+^O_5_

	*W*	P	∑
O1	0.75	1.25	2
O2	0.75	1.25	2
O3	0.75	1.25	2
O4	0.75	1.25	2
O5	1 ×2↓ ×2→		2
∑	5	5	

**Table 9:** *A priori* bond valences (v.u.) for NaFe^3+^Si_2_O_6_

	Na	Fe	Si	∑
O1	0.113 ×2↓	0.45 ×4↓ ×2→	0.988	2
O2	0.263 ×2↓	0.6 ×2↓	1.138	2
O3	0.062 ×4↓ ×2→		0.938 ×2↓ ×2→	2
∑	1	3	4	

**Table 10:** Mean values of Δ_topol_ and Δ_cryst_ for polyhedra distorted as a result of the pseudo-JTE

	No. of coordination polyhedra	〈Δ_topol_〉	〈Δ_cryst_〉	Maximum Δ_cryst_	Compound, ICSD refcode and reference
[4]	36 (8 ions)	0.11	0.092	0.217	KCu^2+^_5_V^5+^_3_O_13_ (400802; Martin & Müller-Buschbaum, 1994*a* ▸)
[5]	14 (7 ions)	0.173	0.159	0.256	Cs[Mo^6+^_2_O_3_(PO_4_)_2_] (79517; Hoareau *et al.*, 1995 ▸)
[6]	67 (12 ions)	0.144	0.181	0.531	K_2_Mo^6+^O_2_(I^5+^O_3_)_4_ (170119; Ok & Halasyamani 2005*a* ▸)
[7]	6 (3 ions)	0.06	0.115	0.192	La(Nb^5+^_5_O_14_) (33783; Hofmann & Gruehn, 1990 ▸)
[8]	7 (4 ions)	0.06	0.087	0.131	KY(W^6+^O_4_) (411285; Gallucci *et al.*, 2000 ▸)
[9]	1	0.078	0.11	0.11	Y_3_Re^7+^O_8_ (15505; Baud *et al.*, 1981 ▸)
[10]	1	0.086	0.079	0.079	YCo^2+^(BO_2_)_5_ (20670; Abdullaev *et al.*, 1980 ▸)
					
^[5]^Cr^2+^	2	0.039	0.077	0.077	SrCr^2+^(P_2_O_7_) (280309; Maaß & Glaum, 2000 ▸)
^[5]^Co^2+^	1	0.032	0.07	0.07	BaCo^2+^_2_(Si_2_O_7_) (81473; Adams *et al.*, 1996 ▸)
^[5]^Cu^2+^	13	0.061	0.084	0.133	Cu^2+^_4_O(PO_4_)_2_ (50459; Schwunck *et al.*, 1998 ▸)
^[6]^Zn^2+^	5	0.056	0.050	0.129	Mn^2+^Zn_2_Ta^5+^_2_O_8_ (85042; Rohweder & Müller-Buschbaum, 1989 ▸)
^[6]^Hg^2+^	2	0.032	0.038	0.066	Hg^2+^(PO_3_)_2_ (280292; Weil & Glaum, 2000 ▸)

**Table 11:** *A priori* bond valences for Sm^3+^3Nb^5+^O_4_S_3_ Observed bond valences for Nb^5+^ are given in parentheses.

	Sm1	Sm2	Sm3	Nb	∑
O1	0.392	0.402	0.369	0.837 (0.948)	2
O2	0.515	0.525		0.960 (1.057)	2
O3	0.400		0.377 ×2×2	0.845 (1.015)	2
O4	0.392	0.402	0.369	0.837 (0.595)	2
S1	0.337 ×2×2	0.348 ×2×2	0.315 ×2×2		2
S2		0.326 ×2×2	0.293 ×2×2	0.761 (0.702)	2
S3	0.313 ×2×2	0.324	0.291	0.759 (0.546)	2
∑	3	3	3	5	

## References

[bb1] Abdullaev, G. K., Mamedov, Kh. S., Dzhafarov, G. G. & Aliev, O. A. (1980). *Zh. Neorg. Khim.* **25**, 364–367.

[bb2] Adams, R. D., Payen, C. & Datta, T. (1996). *Inorg. Chem.* **35**, 3492–3497.

[bb3] Aird, A., Domeneghetti, M. C., Mazzi, F., Tazzoli, V. & Salje, E. K. H. (1998). *J. Phys. Condens. Matter*, **10**, L569–L574.

[bb4] Akella, A. & Keszler, D. A. (1995). *J. Solid State Chem.* **120**, 74–79.

[bb5] Allen, F. H., Kennard, O., Watson, D. G., Brammer, L., Orpen, A. G. & Taylor, R. (1987). *J. Chem. Soc. Perkin Trans. 2*, pp. S1–S19.

[bb6] Allen, F. H. & Motherwell, W. D. S. (2002). *Acta Cryst.* B**58**, 407–422. 10.1107/s0108768102004895 12037362

[bb7] Arlt, T., Armbruster, T., Miletich, R., Ulmer, P. & Peters, T. (1998). *Phys. Chem. Miner.* **26**, 100–106.

[bb8] Armbruster, T., Döbelin, N., Peretti, A., Günther, D., Reusser, E. & Grobéty, B. (2004). *Am. Mineral.* **89**, 610–613.

[bb9] Averbuch-Pouchot, M.-T. (1987). *Z. Anorg. Allg. Chem.* **545**, 118–124.

[bb10] Bader, R. F. W. (1960). *Mol. Phys.* **3**, 137–151.

[bb11] Barrier, N. & Gougeon, P. (2003). *Acta Cryst.* E**59**, 22–24.

[bb12] Bastide, B., Enjalbert, R., Fuess, H. & Galy, J. (2000). *Solid State Sci.* **2**, 545–552.

[bb13] Baud, G., Besse, J.-P., Chevalier, R. & Gasperin, M. (1981). *J. Solid State Chem.* **38**, 186–191.

[bb14] Baud, G., Besse, J.-P., Sueur, G. & Chevalier, R. (1979). *Mater. Res. Bull.* **14**, 675–682.

[bb15] Benbertal, D., Mosset, A. & Trombe, J. C. (1994). *Mater. Res. Bull.* **29**, 47–54.

[bb16] Bersuker, I. B. (1997). *J. Comput. Chem.* **18**, 260–267.

[bb17] Bersuker, I. B. (2006). *The Jahn–Teller Effect*. Cambridge University Press.

[bb18] Bersuker, I. B. (2010). *Electronic Structure and Properties of Transition Metal Compounds: Introduction to the Theory.* Hoboken, New Jersey, USA: John Wiley & Sons Inc.

[bb19] Bersuker, I. B. (2013). *Chem. Rev.* **113**, 1351–1390. 10.1021/cr300279n 23301718

[bb20] Bersuker, I. B. (2016). *Spontaneous Symmetry Breaking in Matter Induced by Degeneracies and Pseudodegeneracies. Advances in Chemical Physics*, Vol. 160, pp. 159–208. Chichester: John Wiley & Sons, Ltd.

[bb21] Bersuker, I. B. (2017). *J. Phys. Conf. Ser.* **833**, 012001.

[bb22] Bluhm, K. & Müller-Buschbaum, Hk. (1989). *Z. Anorg. Allg. Chem.* **575**, 26–30.

[bb23] Boehlke, A. & Müller-Buschbaum, Hk. (1990). *J. Less-Common Met.* **162**, 141–147.

[bb24] Bosi, F. (2014). *Acta Cryst.* B**70**, 697–704. 10.1107/S2052520614011470 25080248

[bb25] Boucher, F., Evain, M. & Brec, R. (1994). *J. Alloys Compd.* **215**, 63–70.

[bb26] Boudin, S., Grandin, A., Labbé, Ph., Provost, J. & Raveau, B. (1996). *J. Solid State Chem.* **127**, 325–330.

[bb27] Boyer-Candalen, C., Meerschaut, A. & Palvadeau, P. (2000). *Mater. Res. Bull.* **35**, 1593–1601.

[bb28] Brese, N. E. & O’Keeffe, M. (1991). *Acta Cryst.* B**47**, 192–197.

[bb29] Brown, I. D. (1978). *Chem. Soc. Rev.* **7**, 359–376.

[bb30] Brown, I. D. (2009). *Chem. Rev.* **109**, 6858–6919. 10.1021/cr900053k PMC2791485 19728716

[bb31] Brown, I. D. (2014). *Bond Valence Thoery. Bond Valences*, edited by I. D. Brown & K. R. Poeppelmeier, ch. 2, pp. 11–58. Berlin, Heidelberg: Springer.

[bb32] Brown, I. D. (2016). *The Chemical Bond in Inorganic Chemistry: The Bond Valence Model*. Oxford University Press.

[bb33] Brown, I. D. & Altermatt, D. (1985). *Acta Cryst.* B**41**, 244–247.

[bb34] Brown, I. D. & Shannon, R. D. (1973). *Acta Cryst.* A**29**, 266–282.

[bb35] Brunel-Laügt, M. & Guitel, J.-C. (1977). *Acta Cryst.* B**33**, 3465–3468.

[bb36] Brynda, J., Kratochvíl, B. & Císařová, I. (1987). *Collect. Czech. Chem. Commun.* **52**, 1742–1747.

[bb37] Burckhardt, H.-G., Platte, C. & Trömel, M. (1982). *Acta Cryst.* B**38**, 2450–2452.

[bb38] Burdett, J. K. (1981). *Inorg. Chem.* **20**, 1959–1962.

[bb39] Burdett, J. K. (1984). *Prog. Solid State Chem.* **15**, 173–255.

[bb40] Calestani, G., Rizzoli, C. & Andreetti, G. D. (1988). *Solid State Commun.* **66**, 223–226.

[bb41] Chen, M., Xia, Z., Molokeev, M. S., Wang, T. & Liu, Q. (2017). *Chem. Mater.* **29**, 1430–1438.

[bb42] Chi, E. O., Ok, K. M., Porter, Y. & Halasyamani, P. S. (2006). *Chem. Mater.* **18**, 2070–2074.

[bb43] Coey, J. M. D. (2005). *Solid State Sci.* **7**, 660–667.

[bb44] Collin, G., Comes, R., Boilot, J. P. & Colomban, Ph. (1986). *J. Phys. Chem. Solids*, **47**, 843–854.

[bb45] Costentin, G., Borel, M. M., Grandin, A., Leclaire, A. & Raveau, B. (1990). *J. Solid State Chem.* **89**, 83–87.

[bb46] Darriet, J., Maazaz, A., Bouloux, J. C. & Delmas, C. (1982). *Z. Anorg. Allg. Chem.* **485**, 115–121.

[bb47] Demartin, F., Gramaccioli, C. M. & Pilati, T. (1992). *Acta Cryst.* C**48**, 1357–1359. 10.1107/s0108768102017925 12456974

[bb48] Dudka, A. P., Kaminskii, A. A. & Simonov, V. I. (1986). *Phys. Status Solidi A*, **93**, 495–502.

[bb49] Durif, A. & Averbuch-Pouchot, M. T. (1978). *Acta Cryst.* B**34**, 3335–3337.

[bb50] Efremov, V. A., Davydova, N. N. & Trunov, V. K. (1988). *Zh Neorg Khim.* **33**, 3001–3004.

[bb51] Engh, R. A. & Huber, R. (1991). *Acta Cryst.* A**47**, 392–400.

[bb52] Enjalbert, R., Guinneton, F. & Galy, J. (1999). *Acta Cryst.* C**55**, 273–276.

[bb53] Fan, Y.-H., Jiang, X.-M., Liu, B.-W., Li, S.-F., Guo, W.-H., Zeng, H.-Y., Guo, G.-C. & Huang, J.-S. (2017). *Inorg. Chem.* **56**, 114–124. 10.1021/acs.inorgchem.6b01016 27983831

[bb54] Fukuzumi, S. (2001). *Fundamental Concepts of Catalysis in Electron Transfer. Electron Transfer in Chemistry*, edited by V. Balzani, Vol. 4, ch. 1, pp. 2–67. Chichester: John Wiley & Sons Ltd.

[bb55] Gagné, O. C. (2018). *Acta Cryst.* B**74**, 49–62.

[bb56] Gagné, O. C. (2020). ChemRxiv:11626974.

[bb57] Gagné, O. C. & Hawthorne, F. C. (2015). *Acta Cryst.* B**71**, 562–578. 10.1107/S2052520615016297 PMC4591556 26428406

[bb58] Gagné, O. C. & Hawthorne, F. C. (2016). *Acta Cryst.* B**72**, 602–625. 10.1107/S2052520616008507 PMC4971548 27484381

[bb59] Gagné, O. C. & Hawthorne, F. C. (2017*a*). *Acta Cryst.* B**73**, 956–961. 10.1107/S2052520617010988 28981002

[bb60] Gagné, O. C. & Hawthorne, F. C. (2017*b*). *Acta Cryst.* B**73**, 1019–1031.

[bb61] Gagné, O. C. & Hawthorne, F. C. (2018*a*). *Acta Cryst.* B**74**, 63–78.

[bb62] Gagné, O. C. & Hawthorne, F. C. (2018*b*). *Acta Cryst.* B**74**, 79–96.

[bb63] Gagné, O. C., Mercier, P. H. J. & Hawthorne, F. C. (2018). *Acta Cryst.* B**74**, 470–482.

[bb64] Gallucci, E., Goutaudier, C., Boulon, G., Cohen-Adad, M. Th. & Mentzen, B. F. (2000). *J. Cryst. Growth*, **209**, 895–905.

[bb65] Galy, J., Enjalbert, R., Rozier, P. & Millet, P. (2002). *Acta Cryst.* C**58**, i6–i8. 10.1107/s010827010101767x 11781447

[bb66] Garcia-Fernandez, P. & Bersuker, I. B. (2011). *Phys. Rev. Lett.* **106**, 246406. 10.1103/PhysRevLett.106.246406 21770587

[bb67] Gärtner, M., Abeln, D., Pring, A., Wilde, M. & Reller, A. (1994). *J. Solid State Chem.* **111**, 128–133.

[bb68] Gasperin, M. (1981). *Acta Cryst.* B**37**, 641–643.

[bb69] Giester, G. (1995). *Mineral. Petrol.* **53**, 165–171.

[bb70] Glaum, R. (1992). *Z. Anorg. Allg. Chem.* **616**, 46–52.

[bb71] Glaum, R. (1993). *Z. Kristallogr. Cryst. Mater.* **205**, 69–83.

[bb72] Glaum, R. & Schmidt, A. (1996). *Acta Cryst.* C**52**, 762–764.

[bb73] Gravereau, P., Hardy, A. & Bonnin, A. (1977). *Acta Cryst.* B**33**, 1362–1367.

[bb74] Grigor’ev, M. S., Baturin, N. A., Plotnikova, T. E., Fedoseev, A. M. & Budantseva, N. A. (1991). *Radiokhimiya*, **2**, 322.

[bb75] Groom, C. R. & Allen, F. H. (2014). *Angew. Chem. Int. Ed.* **53**, 662–671. 10.1002/anie.201306438 24382699

[bb76] Groom, C. R., Bruno, I. J., Lightfoot, M. P. & Ward, S. C. (2016). *Acta Cryst.* B**72**, 171–179. 10.1107/S2052520616003954 PMC4822653 27048719

[bb77] Guionneau, P. (2014). *Dalton Trans.* **43**, 382–393. 10.1039/c3dt52520a 24201509

[bb78] Guo, G.-C., Zhuang, J.-N., Wang, Y.-G., Chen, J.-T., Zhuang, H.-H., Huang, J.-S. & Zhang, Q.-E. (1996). *Acta Cryst.* C**52**, 5–7.

[bb79] Halasyamani, P. S. (2004). *Chem. Mater.* **16**, 3586–3592.

[bb80] Halasyamani, P. S. & Poeppelmeier, K. R. (1998). *Chem. Mater.* **10**, 2753–2769.

[bb81] Halcrow, M. A. (2013). *Spin-Crossover Materials*, ch. 5, pp. 147–169. Chichester: John Wiley & Sons Ltd.

[bb82] Harrison, W. T. A. (1999). *Acta Cryst.* C**55**, 1980–1983.

[bb83] Hartenbach, I., Lissner, F., Nikelski, T., Meier, S. F., Müller-Bunz, H. & Schleid, T. (2005). *Z. Anorg. Allg. Chem.* **631**, 2377–2382.

[bb84] Hawthorne, F. C. & Grundy, H. D. (1977). *Can. Mineral.* **15**, 50–58.

[bb85] Hidouri, M., Lajmi, B. & Ben Amara, M. (2002). *Acta Cryst.* C**58**, i147–i148. 10.1107/s010827010201541x 12415148

[bb86] Hoareau, T., Leclaire, A., Borel, M. M., Grandin, A. & Raveau, B. (1995). *J. Solid State Chem.* **116**, 87–91.

[bb87] Hoffmann, R. & Hoppe, R. (1989). *Z. Anorg. Allg. Chem.* **573**, 143–156.

[bb88] Hofmann, R. & Gruehn, R. (1990). *Z. Anorg. Allg. Chem.* **590**, 81–92.

[bb89] Hübner, N. & Gruehn, R. (1991). *Z. Anorg. Allg. Chem.* **602**, 119–128.

[bb90] Hübner, N., Schaffrath, U. & Gruehn, R. (1990). *Z. Anorg. Allg. Chem.* **591**, 107–117.

[bb91] Hughes, J., Schindler, M., Rakovan, J. & Cureton, F. (2002). *Can. Mineral.* **40**, 1429–1435.

[bb92] Ito, S., Kurosawa, H., Akashi, K., Michiue, Y. & Watanabe, M. (1996). *Solid State Ionics*, **86–88**, 745–750.

[bb93] Jahn, H. A., Teller, E. & Donnan, F. G. (1937). *Proc. R. Soc. Lond. Ser. A*, **161**, 220–235.

[bb94] Jain, A., Ong, S. P., Hautier, G., Chen, W., Richards, W. D., Dacek, S., Cholia, S., Gunter, D., Skinner, D., Ceder, G. & Persson, K. A. (2013). *APL Mater.* **1**, 011002.

[bb95] Jeitschko, W., Heumannskämper, D. H., Rodewald, U. C. & Schriewer-Pöttgen, M. S. (2000). *Z. Anorg. Allg. Chem.* **626**, 80–88.

[bb96] Johansson, G. & Sandström, M. (1978). *Acta Chem. Scand.* **32*a***, 109–113.

[bb97] Johnson, J. W., Johnston, D. C., King, H. E., Halbert, T. R., Brody, J. F. & Goshorn, D. P. (1988). *Inorg. Chem.* **27**, 1646–1648.

[bb98] Kakekhani, A. & Ismail-Beigi, S. (2015). *ACS Catal.* **5**, 4537–4545.

[bb99] Køuhl, P. (1978). *Z. Anorg. Allg. Chem.* **442**, 280–288.

[bb100] Krivovichev, S. V., Filatov, S. K., Cherepansky, P. N., Armbruster, T. & Pankratova, O. Yu. (2005). *Can. Mineral.* **43**, 671–677.

[bb101] Kudo, A. & Hijii, S. (1999). *Chem. Lett.* **28**, 1103–1104.

[bb102] Kunz, M. & Brown, I. D. (1995). *J. Solid State Chem.* **115**, 395–406.

[bb103] Lai, W., Wang, Y., Morelli, D. T. & Lu, X. (2015). *Adv. Funct. Mater.* **25**, 3648–3657.

[bb104] Laskowski, R. A., Moss, D. S. & Thornton, J. M. (1993). *J. Mol. Biol.* **231**, 1049–1067. 10.1006/jmbi.1993.1351 8515464

[bb105] Leclaire, A., Caignaert, V. & Raveau, B. (2007). *J. Solid State Chem.* **180**, 2044–2052.

[bb106] Leclaire, A., Chardon, J. & Raveau, B. (2003). *J. Solid State Chem.* **172**, 412–416.

[bb107] Lenaz, D., Skogby, H., Princivalle, F. & Hålenius, U. (2004). *Phys. Chem. Miner.* **31**, 633–642.

[bb108] Leonowicz, M. E., Johnson, J. W., Brody, J. F., Shannon, H. F. & Newsam, J. M. (1985). *J. Solid State Chem.* **56**, 370–378.

[bb109] Levason, W., Oldroyd, R. D. & Webster, M. (1994). *J. Chem. Soc. Dalton Trans.* pp. 2983–2988.

[bb110] Li, P.-J., Huang, S.-H., Huang, K.-Y., Ru-Ji, W. & Mak, T. C. W. (1990). *Inorg. Chim. Acta*, **175**, 105–110.

[bb111] Li, X., Lu, C., Dai, J., Dong, S., Chen, Y., Hu, N., Wu, G., Liu, M., Yan, Z. & Liu, J.-M. (2014). *Sci. Rep.* **4**, 7019. 10.1038/srep07019 PMC4228326 25387445

[bb112] Liao, J.-H. & Tsai, M.-C. (2002). *Cryst. Growth Des.* **2**, 83–85.

[bb113] Lii, K. H., Chueh, B. R., Kang, H. Y. & Wang, S. L. (1992). *J. Solid State Chem.* **99**, 72–77.

[bb114] Liu, X. & Liebermann, R. C. (1993). *Phys. Chem. Miner.* **20**, 171–175.

[bb115] Longuet-Higgins, H. C. & Salem, L. (1959). *Proc. R. Soc. Lond. Ser. A*, **251**, 172–185.

[bb116] Louër, D., Labarre, J., Auffredic, J.-P. & Louër, M. (1982). *Acta Cryst.* B**38**, 1079–1084.

[bb117] Lussier, A. J., Lopez, R. A. K. & Burns, P. C. (2016). *Can. Mineral.* **54**, 177–283.

[bb118] Maaß, K. & Glaum, R. (2000). *Acta Cryst.* C**56**, 404–406. 10.1107/S0108270100000226 10815187

[bb119] Marinkovic, B. A., Ari, M., de Avillez, R. R., Rizzo, F., Ferreira, F. F., Miller, K. J., Johnson, M. B. & White, M. A. (2009). *Chem. Mater.* **21**, 2886–2894.

[bb122] Martin, F.-D. & Miiller-Buschbaum, Hk. (1995). *Z. Naturforsch. Teil B*, **50**, 243–246.

[bb120] Martin, F.-D. & Müller-Buschbaum, Hk. (1994*a*). *Z. Naturforsch. Teil B*, **49**, 1137–1140.

[bb121] Martin, F.-D. & Müller-Buschbaum, Hk. (1994*b*). *Z. Naturforsch. Teil B*, **49**, 1459–1462.

[bb123] Mayer, J. M. (1988). *Inorg. Chem.* **27**, 3899–3903.

[bb124] Mellini, M. & Merlino, S. (1979). *TMPM Tschermaks Petr. Mitt.* **26**, 109–123.

[bb125] Millet, P., Galy, J. & Johnsson, M. (1999). *Solid State Sci.* **1**, 279–286.

[bb126] Mori, T., Kamegashira, N., Aoki, K., Shishido, T. & Fukuda, T. (2002). *Mater. Lett.* **54**, 238–243.

[bb127] Mormann, Th. J. & Jeitschko, W. (2001). *Z. Kristallogr. New Cryst. Struct.* **216**, 3–4.

[bb128] Murashova, E. V., Velikodnyi, Y. A. & Trunov, V. K. (1991). *Zh. Neorg. Khim.* **36**, 479–481.

[bb129] Nestola, F., Tribaudino, M., Boffa Ballaran, T., Liebske, C. & Bruno, M. (2007). *Am. Mineral.* **92**, 1492–1501.

[bb130] Nevskii, N. N., Ivanov-Emin, B. N., Nevskaya, N. A. & Belov, N. V. (1982). *Dokl. Akad. Nauk SSSR*, **266**, 628–630.

[bb131] Nevskii, N. N. & Porai Koshits, M. A. (1983). *Dokl. Akad. Nauk SSSR*, **270**, 1392–1395.

[bb132] Oka, Y., Yao, T. & Yamamoto, N. (1997). *Mater. Res. Bull.* **32**, 1201–1209.

[bb133] Ok, K. M. & Halasyamani, P. S. (2005*a*). *Inorg. Chem.* **44**, 2263–2271. 10.1021/ic048428c 15792461

[bb134] Ok, K. M. & Halasyamani, P. S. (2005*b*). *Inorg. Chem.* **44**, 9353–9359. 10.1021/ic051340u 16323920

[bb135] Ok, K. M., Halasyamani, P. S., Casanova, D., Llunell, M., Alemany, P. & Alvarez, S. (2006). *Chem. Mater.* **18**, 3176–3183.

[bb136] Ong, S. P., Richards, W. D., Jain, A., Hautier, G., Kocher, M., Cholia, S., Gunter, D., Chevrier, V. L., Persson, K. A. & Ceder, G. (2013). *Comput. Mater. Sci.* **68**, 314–319.

[bb137] Öpik, U. & Pryce, M. H. L. (1957). *Proc. R. Soc. Lond. Ser. A*, **238**, 425–447.

[bb138] Orpen, A. G., Brammer, L., Allen, F. H., Kennard, O., Watson, D. G. & Taylor, R. (1989). *J. Chem. Soc. Dalton Trans.* pp. S1–83.

[bb139] Osterloh, D. & Müller-Buschbaum, Hk. (2014). *Z. Naturforsch. Teil B*, **49**, 923–926.

[bb140] Pauling, L. (1947). *J. Am. Chem. Soc.* **69**, 542–553.

[bb141] Pearson, R. G. (1969). *J. Am. Chem. Soc.* **91**, 4947–4955.

[bb142] Peuchert, U., Bohatý, L. & Fröhlich, R. (1995). *Acta Cryst.* C**51**, 1719–1721.

[bb143] Pfennig, B. W. (2015). *Principles of Inorganic Chemistry*. Hoboken, New Jersey, USA: Wiley.

[bb144] Porter, Y. & Halasyamani, P. S. (2003). *J. Solid State Chem.* **174**, 441–449.

[bb145] Preiser, C., Lösel, J., Brown, I. D., Kunz, M. & Skowron, A. (1999). *Acta Cryst.* B**55**, 698–711. 10.1107/s0108768199003961 10927409

[bb146] Protas, J., Menaert, B., Marnier, G. & Boulanger, B. (1991). *Acta Cryst.* C**47**, 698–701.

[bb147] Quarez, E., Abraham, F. & Mentré, O. (2003). *J. Solid State Chem.* **176**, 137–150.

[bb148] Ra, H.-S., Ok, K. M. & Halasyamani, P. S. (2003). *J. Am. Chem. Soc.* **125**, 7764–7765. 10.1021/ja035314b 12822970

[bb149] Range, K.-J., Rau, F. & Klement, U. (1991). *Z. Naturforsch. Teil B*, **46**, 1315–1318.

[bb150] Reeves, M. G., Wood, P. A. & Parsons, S. (2019). *Acta Cryst.* B**75**, 1096–1105. 10.1107/S2052520619013040 32830689

[bb151] Reinen, D. & Atanasov, M. (1991). *Chem. Phys.* **155**, 157–171.

[bb152] Reinen, D. & Friebel, C. (1984). *Inorg. Chem.* **23**, 791–798.

[bb153] Richardson, I. G. (2013). *Acta Cryst.* B**69**, 150–162. 10.1107/S205251921300376X PMC3606179 23719702

[bb154] Riou, A., Gerault, Y. & Cudennec, Y. (1986). *Rev. Chim. Min.* **23**, 70–79.

[bb155] Roesky, H. W., Haiduc, I. & Hosmane, N. S. (2003). *Chem. Rev.* **103**, 2579–2595. 10.1021/cr020376q 12848580

[bb156] Rohweder, U. & Müller-Buschbaum, Hk. (1989). *Z. Anorg. Allg. Chem.* **572**, 102–108.

[bb157] Roulhac, P. L. & Palenik, G. J. (2003). *Inorg. Chem.* **42**, 118–121. 10.1021/ic025980y 12513085

[bb158] Roy, M. E. de, Besse, J. P., Chevalier, R. & Gasperin, M. (1987). *J. Solid State Chem.* **67**, 185–189.

[bb159] Rulíšek, L. & Vondrášek, J. (1998). *J. Inorg. Biochem.* **71**, 115–127. 10.1016/s0162-0134(98)10042-9 9833317

[bb160] Salinas-Sanchez, A., Garcia-Muñoz, J. L., Rodriguez-Carvajal, J., Saez-Puche, R. & Martinez, J. L. (1992). *J. Solid State Chem.* **100**, 201–211.

[bb161] Sasaki, S. & Takéuchi, Y. (2015). *Z. Kristallogr. Cryst. Mater.* **158**, 279–298.

[bb162] Satto, C., Millet, P. & Galy, J. (1997). *Acta Cryst.* C**53**, 1727–1728.

[bb163] Schaefer, J. & Bluhm, K. (1994). *Z. Anorg. Allg. Chem.* **620**, 1051–1055.

[bb164] Schindler, M., Hawthorne, F. C. & Baur, W. H. (2000). *Chem. Mater.* **12**, 1248–1259.

[bb165] Schwunck, H.-M., Moser, P. & Jung, W. (1998). *Z. Anorg. Allg. Chem.* **624**, 1262–1266.

[bb166] Senthil Kumar, K. & Ruben, M. (2017). *Coord. Chem. Rev.* **346**, 176–205.

[bb167] Serezhkin, V. N., Efremov, V. A. & Trunov, V. K. (1987). *Zh. Neorg. Khim.* **32**, 2695–2699.

[bb168] Shannon, R. D. (1976). *Acta Cryst.* A**32**, 751–767.

[bb169] Shields, G. P., Raithby, P. R., Allen, F. H. & Motherwell, W. D. S. (2000). *Acta Cryst.* B**56**, 455–465. 10.1107/s0108768199015086 10877354

[bb170] Shpanchenko, R. V., Kaul, E. E., Geibel, C. & Antipov, E. V. (2006). *Acta Cryst.* C**62**, i88–i90. 10.1107/S0108270106021597 17008726

[bb171] Strömberg, D., Sandström, M. & Wahlgren, U. (1990). *Chem. Phys. Lett.* **172**, 49–54.

[bb172] Surblé, S., Obbade, S., Saad, S., Yagoubi, S., Dion, C. & Abraham, F. (2006). *J. Solid State Chem.* **179**, 3238–3251.

[bb173] Swinnea, J. S. & Steinfink, H. (1987). *Acta Cryst.* C**43**, 2436–2437.

[bb174] Szymanski, N. J., Walters, L. N., Puggioni, D. & Rondinelli, J. M. (2019). *Phys. Rev. Lett.* **123**, 236402. 10.1103/PhysRevLett.123.236402 31868440

[bb175] Tran Qui, D., Capponi, J. J., Joubert, J. C. & Shannon, R. D. (1981). *J. Solid State Chem.* **39**, 219–229.

[bb176] Ukei, K., Suzuki, H., Shishido, T. & Fukuda, T. (1994). *Acta Cryst.* C**50**, 655–656.

[bb177] Walsh, A., Payne, D. J., Egdell, R. G. & Watson, G. W. (2011). *Chem. Soc. Rev.* **40**, 4455–4463. 10.1039/c1cs15098g 21666920

[bb178] Wang, L. H., Zhao, M. L., Wang, C. L., Wang, J., Kuai, W. J. & Tao, X. T. (2012). *Appl. Phys. Lett.* **101**, 062903.

[bb179] Wang, S. L., Wang, C. C. & Lii, K. H. (1989). *J. Solid State Chem.* **82**, 298–302.

[bb180] Waroquiers, D., Gonze, X., Rignanese, G.-M., Welker-Nieuwoudt, C., Rosowski, F., Göbel, M., Schenk, S., Degelmann, P., André, R., Glaum, R. & Hautier, G. (2017). *Chem. Mater.* **29**, 8346–8360.

[bb181] Wedel, B. & Müller-Buschbaum, Hk. (2014). *Z. Naturforsch. Teil B*, **51**, 1587–1590.

[bb183] Weiß C. & Hoppe, R. (1996). *Z. Anorg. Allg. Chem.* **622**, 1019–1026.

[bb182] Weil, M. & Glaum, R. (2000). *Acta Cryst.* C**56**, 133–135. 10.1107/s0108270199014067 10777861

[bb184] Welk, M. E., Norquist, A. J., Arnold, F. P., Stern, C. L. & Poeppelmeier, K. R. (2002). *Inorg. Chem.* **41**, 5119–5125. 10.1021/ic025622v 12354045

[bb185] Wilkens, J. & Müller-Buschbaum, Hk. (1993). *Z. Anorg. Allg. Chem.* **619**, 517–520.

[bb186] Womersley, M. N., Thomas, P. A. & Corker, D. L. (1998). *Acta Cryst.* B**54**, 635–644.

[bb187] Wood, R. M., Abboud, K. A., Palenik, R. C. & Palenik, G. J. (2000). *Inorg. Chem.* **39**, 2065–2068. 10.1021/ic990982c 12526513

[bb188] Wu, B.-L., Hu, C.-L., Mao, F.-F., Tang, R.-L. & Mao, J.-G. (2019). *J. Am. Chem. Soc.* **141**, 10188–10192. 10.1021/jacs.9b05125 31204805

[bb189] Yakubovich, O. V., Simonov, M. A. & Belov, N. V. (1975). *Kristallografiya*, **20**, 152–155.

[bb190] Yamane, H., Takahashi, H., Kajiwara, T. & Shimada, M. (1999). *Acta Cryst.* C**55**, 1978–1980. 10.1107/s0108270100009306 11025285

[bb191] Yang, R., Terabe, K., Yao, Y., Tsuruoka, T., Hasegawa, T., Gimzewski, J. K. & Aono, M. (2013). *Nanotechnology*, **24**, 384003. 10.1088/0957-4484/24/38/384003 23999098

[bb192] Yeon, J., Kim, S.-H. & Halasyamani, P. S. (2010). *Inorg. Chem.* **49**, 6986–6993. 10.1021/ic1008297 20608660

[bb193] Yilmaz, A., Bu, X., Kizilyalli, M., Kniep, R. & Stucky, G. D. (2001). *J. Solid State Chem.* **156**, 281–285.

[bb194] Yin, W.-J., Yang, J.-H., Kang, J., Yan, Y. & Wei, S.-H. (2015). *J. Mater. Chem. A*, **3**, 8926–8942.

[bb195] Zaitsev, S. M., Zhavoronko, G. P., Tatarenko, A. A., Kupriyanov, M. F., Filip’ev, V. S. & Fesenko, E. G. (1979). *Kristallografiya*, **24**, 826–828.

[bb196] Zhesheng, M., Ruilin, H. & Xiaoling, Z. (1991). *Acta Geol. Sin. Engl. Ed.* **4**, 145–151.

[bb200] Zunger, A. (2019). *Nature*, **566**, 447–450. 10.1038/d41586-019-00676-y 30814720

