# Supplementary material for: Bond-length distributions for ions bonded to oxygen: results for the transition metals and quantification of the factors underlying bond-length variation in inorganic solids
Source: IUCrJ. 2020 Jun 9;7(Pt 4):581–629. doi: 10.1107/S2052252520005928 (PMC7340253; doi:10.1107/S2052252520005928)
Supplement: Supplementary file 1 [file m-07-00581-sup1.pdf]

IUCrJ

Volume 7 (2020)

Supporting information for article:

Bond-length distributions for ions bonded to oxygen: results for the transition metals and quantification of the factors underlying bond-length variation in inorganic solids

Olivier Charles Gagné and Frank Christopher Hawthorne

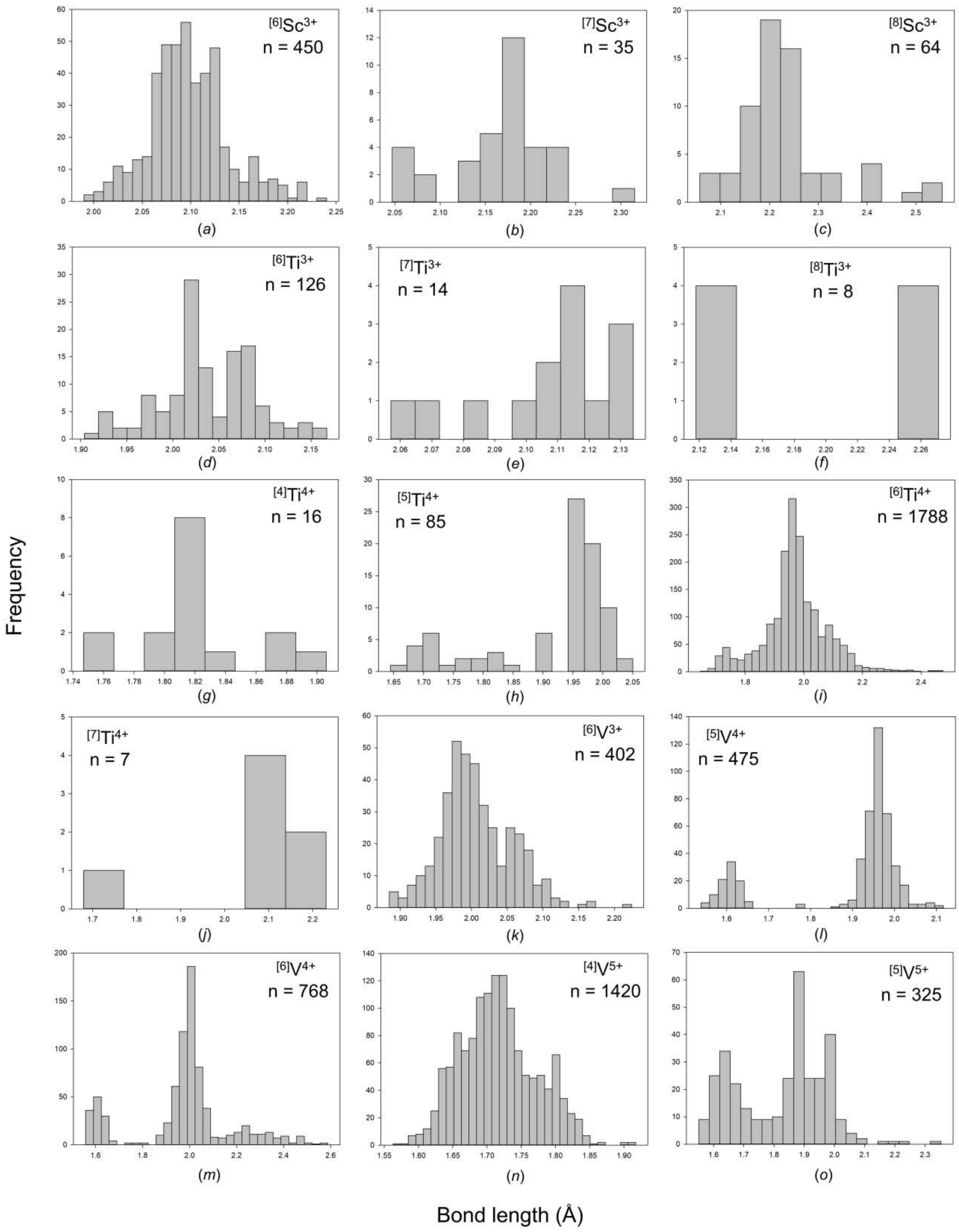

[6]Sc3+ n = 450
(a)
[7]Sc3+ n = 35
(b)
[8]Sc3+ n = 64
(c)
[6]Ti3+ n = 126
(d)
[7]Ti3+ n = 14
(e)
[8]Ti3+ n = 8
(f)
[4]Ti4+ n = 16
(g)
[5]Ti4+ n = 85
(h)
[6]Ti4+ n = 1788
(i)
[7]Ti4+ n = 7
(j)
[6]V3+ n = 402
(k)
[5]V4+ n = 475
(l)
[6]V4+ n = 768
(m)
[4]V5+ n = 1420
(n)
[5]V5+ n = 325
(o)
Frequency
Bond length (Å)

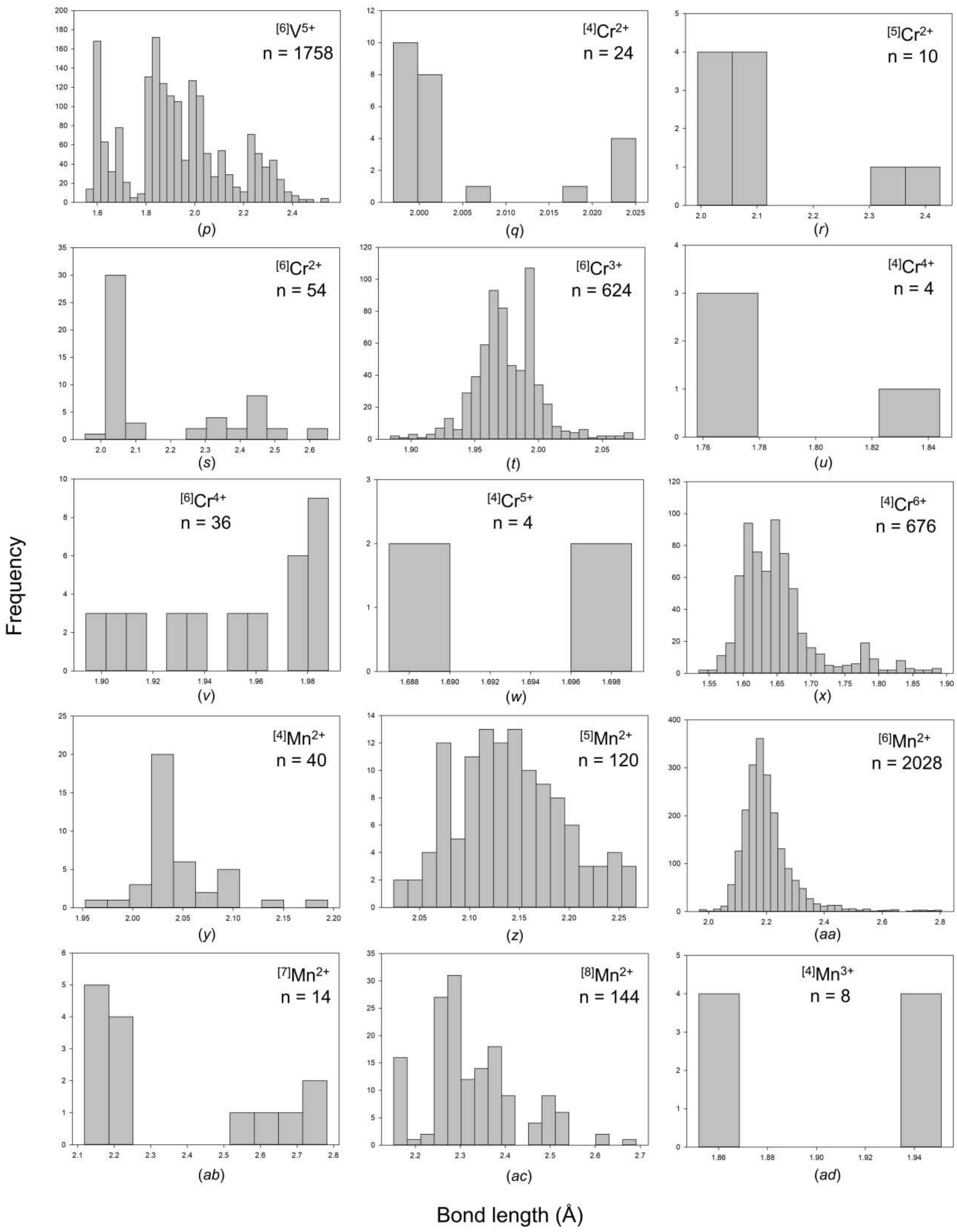
[6]V5+
n = 1758
(p)
[4]Cr2+
n = 24
(q)
[5]Cr2+
n = 10
(r)
[6]Cr2+
n = 54
(s)
[6]Cr3+
n = 624
(t)
[4]Cr4+
n = 4
(u)
[6]Cr4+
n = 36
(v)
[4]Cr5+
n = 4
(w)
[4]Cr6+
n = 676
(x)
[4]Mn2+
n = 40
(y)
[5]Mn2+
n = 120
(z)
[6]Mn2+
n = 2028
(aa)
[7]Mn2+
n = 14
(ab)
[8]Mn2+
n = 144
(ac)
[4]Mn3+
n = 8
(ad)
Frequency
Bond length (Å)

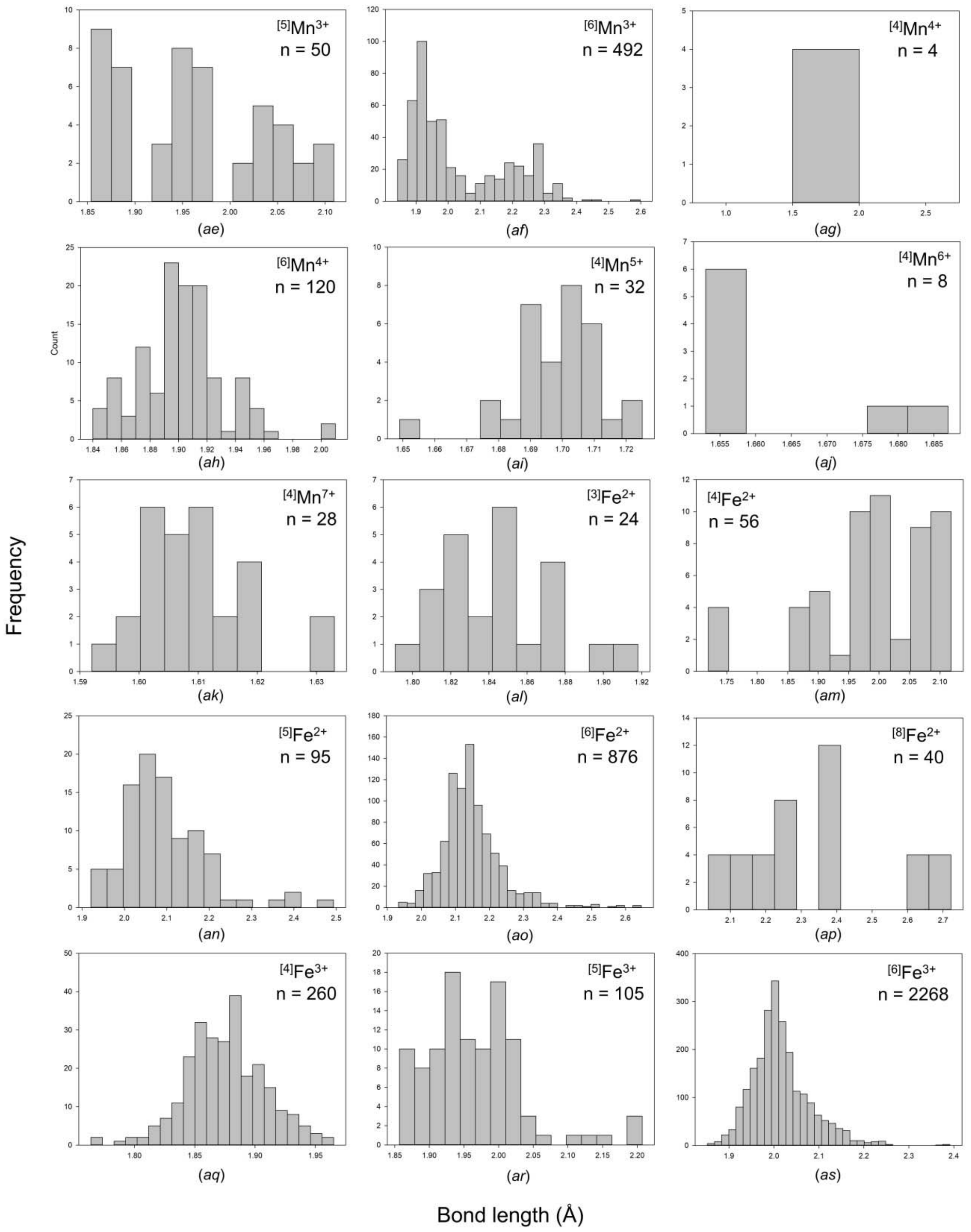
[5]Mn3+
n = 50
(ae)
[6]Mn3+
n = 492
(af)
[4]Mn4+
n = 4
(ag)
[6]Mn4+
n = 120
Count
(ah)
[4]Mn5+
n = 32
(ai)
[4]Mn6+
n = 8
(aj)
[4]Mn7+
n = 28
(ak)
[3]Fe2+
n = 24
(al)
[4]Fe2+
n = 56
(am)
[5]Fe2+
n = 95
(an)
[6]Fe2+
n = 876
(ao)
[8]Fe2+
n = 40
(ap)
[4]Fe3+
n = 260
(aq)
[5]Fe3+
n = 105
(ar)
[6]Fe3+
n = 2268
(as)
Frequency
Bond length (Å)

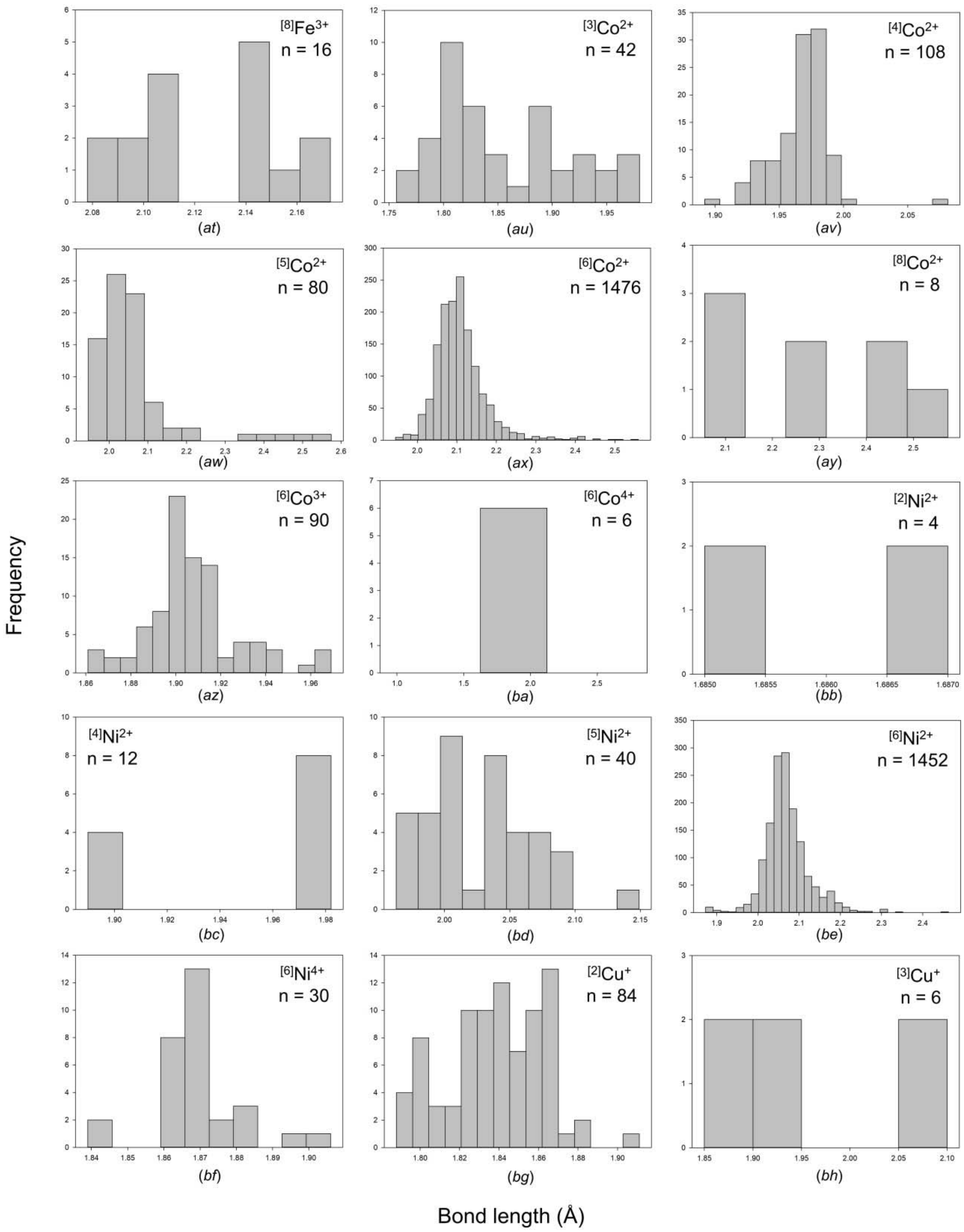

[8]Fe3+ n = 16
(at)
[3]Co2+ n = 42
(au)
[4]Co2+ n = 108
(av)
[5]Co2+ n = 80
(aw)
[6]Co2+ n = 1476
(ax)
[8]Co2+ n = 8
(ay)
[6]Co3+ n = 90
(az)
[6]Co4+ n = 6
(ba)
[2]Ni2+ n = 4
(bb)
[4]Ni2+ n = 12
(bc)
[5]Ni2+ n = 40
(bd)
[6]Ni2+ n = 1452
(be)
[6]Ni4+ n = 30
(bf)
[2]Cu+ n = 84
(bg)
[3]Cu+ n = 6
(bh)
Frequency
Bond length (Å)

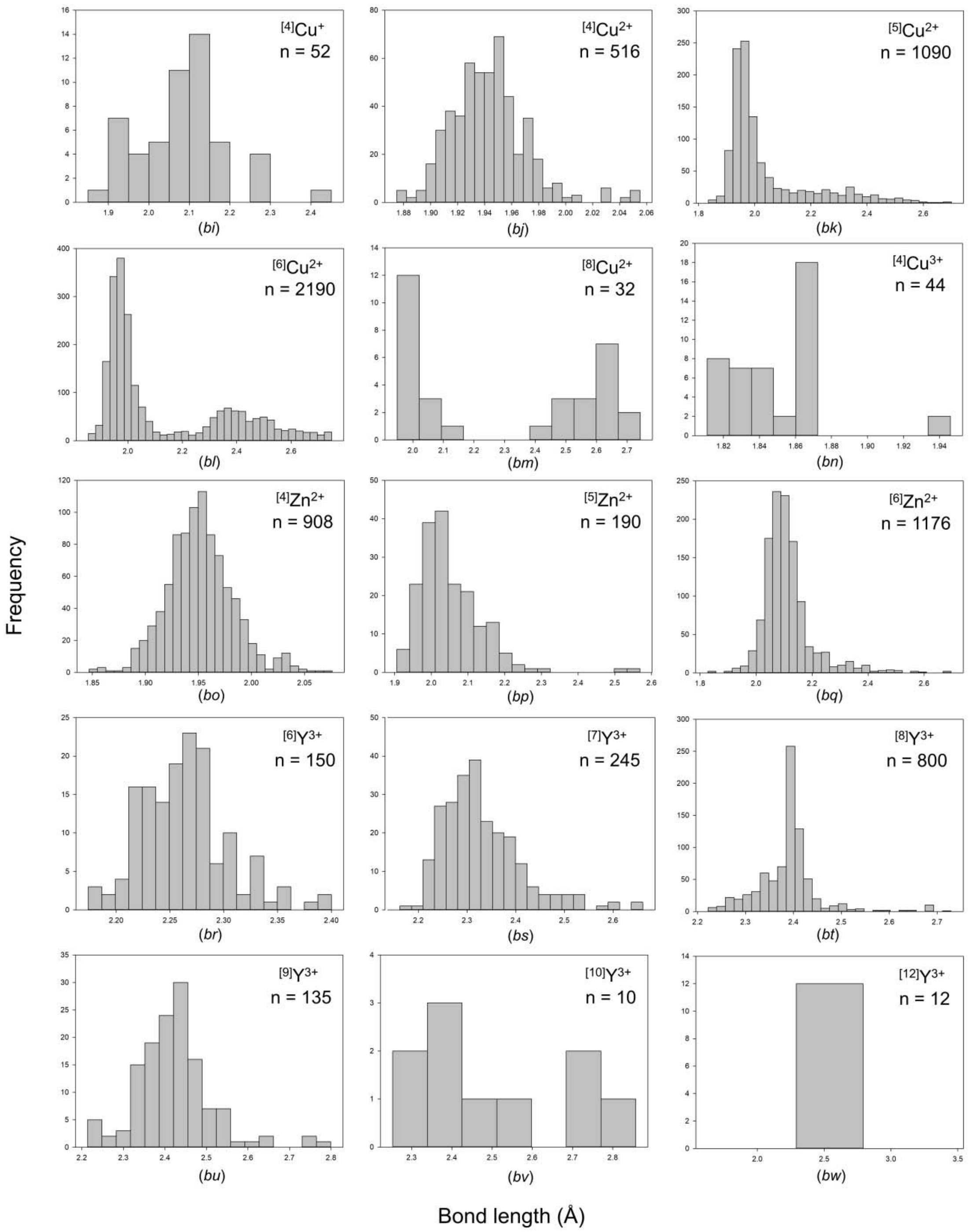
Frequency
Bond length (Å)
[4]Cu+ n = 52
[4]Cu2+ n = 516
[5]Cu2+ n = 1090
[6]Cu2+ n = 2190
[8]Cu2+ n = 32
[4]Cu3+ n = 44
[4]Zn2+ n = 908
[5]Zn2+ n = 190
[6]Zn2+ n = 1176
[6]Y3+ n = 150
[7]Y3+ n = 245
[8]Y3+ n = 800
[9]Y3+ n = 135
[10]Y3+ n = 10
[12]Y3+ n = 12
(bi)
(bj)
(bk)
(bl)
(bm)
(bn)
(bo)
(bp)
(bq)
(br)
(bs)
(bt)
(bu)
(bv)
(bw)

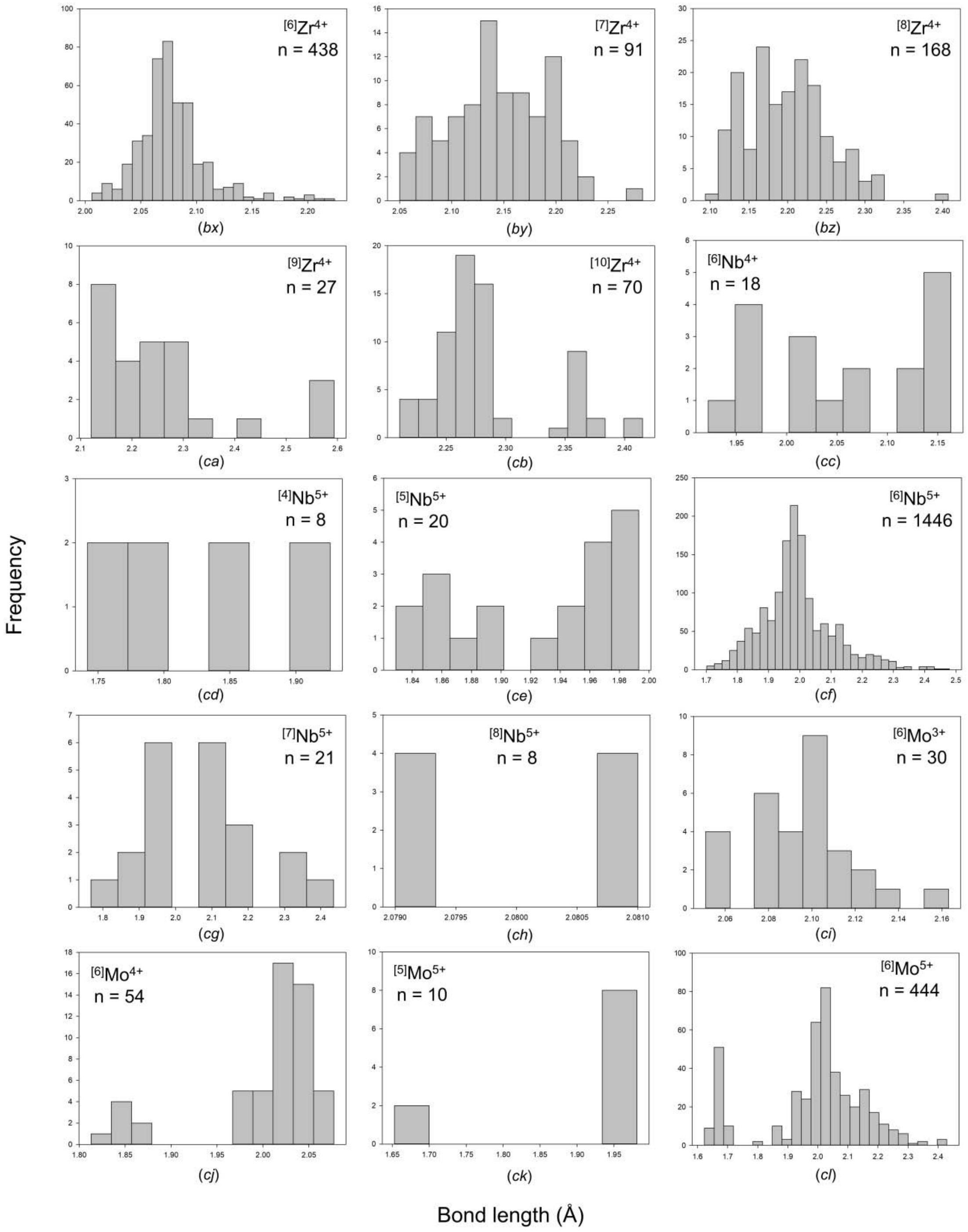
[6]Zr4+
n = 438
(bx)
[7]Zr4+
n = 91
(by)
[8]Zr4+
n = 168
(bz)
[9]Zr4+
n = 27
(ca)
[10]Zr4+
n = 70
(cb)
[6]Nb4+
n = 18
(cc)
[4]Nb5+
n = 8
(cd)
[5]Nb5+
n = 20
(ce)
[6]Nb5+
n = 1446
(cf)
[7]Nb5+
n = 21
(cg)
[8]Nb5+
n = 8
(ch)
[6]Mo3+
n = 30
(ci)
[6]Mo4+
n = 54
(cj)
[5]Mo5+
n = 10
(ck)
[6]Mo5+
n = 444
(cl)
Frequency
Bond length (Å)

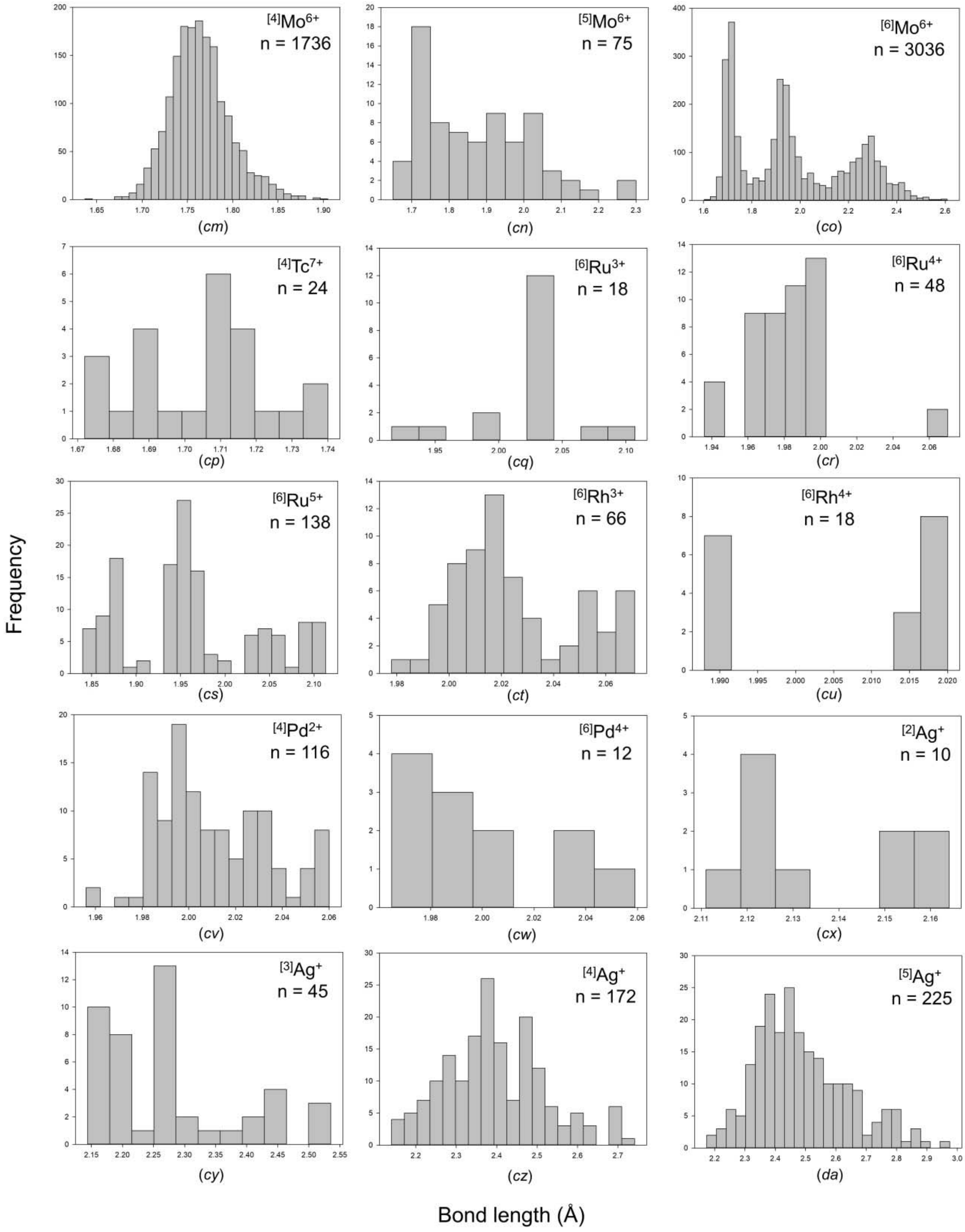
[4]Mo6+
n = 1736
(cm)
[5]Mo6+
n = 75
(cn)
[6]Mo6+
n = 3036
(co)
[4]Tc7+
n = 24
(cp)
[6]Ru3+
n = 18
(cq)
[6]Ru4+
n = 48
(cr)
[6]Ru5+
n = 138
(cs)
[6]Rh3+
n = 66
(ct)
[6]Rh4+
n = 18
(cu)
[4]Pd2+
n = 116
(cv)
[6]Pd4+
n = 12
(cw)
[2]Ag+
n = 10
(cx)
[3]Ag+
n = 45
(cy)
[4]Ag+
n = 172
(cz)
[5]Ag+
n = 225
(da)
Frequency
Bond length (Å)

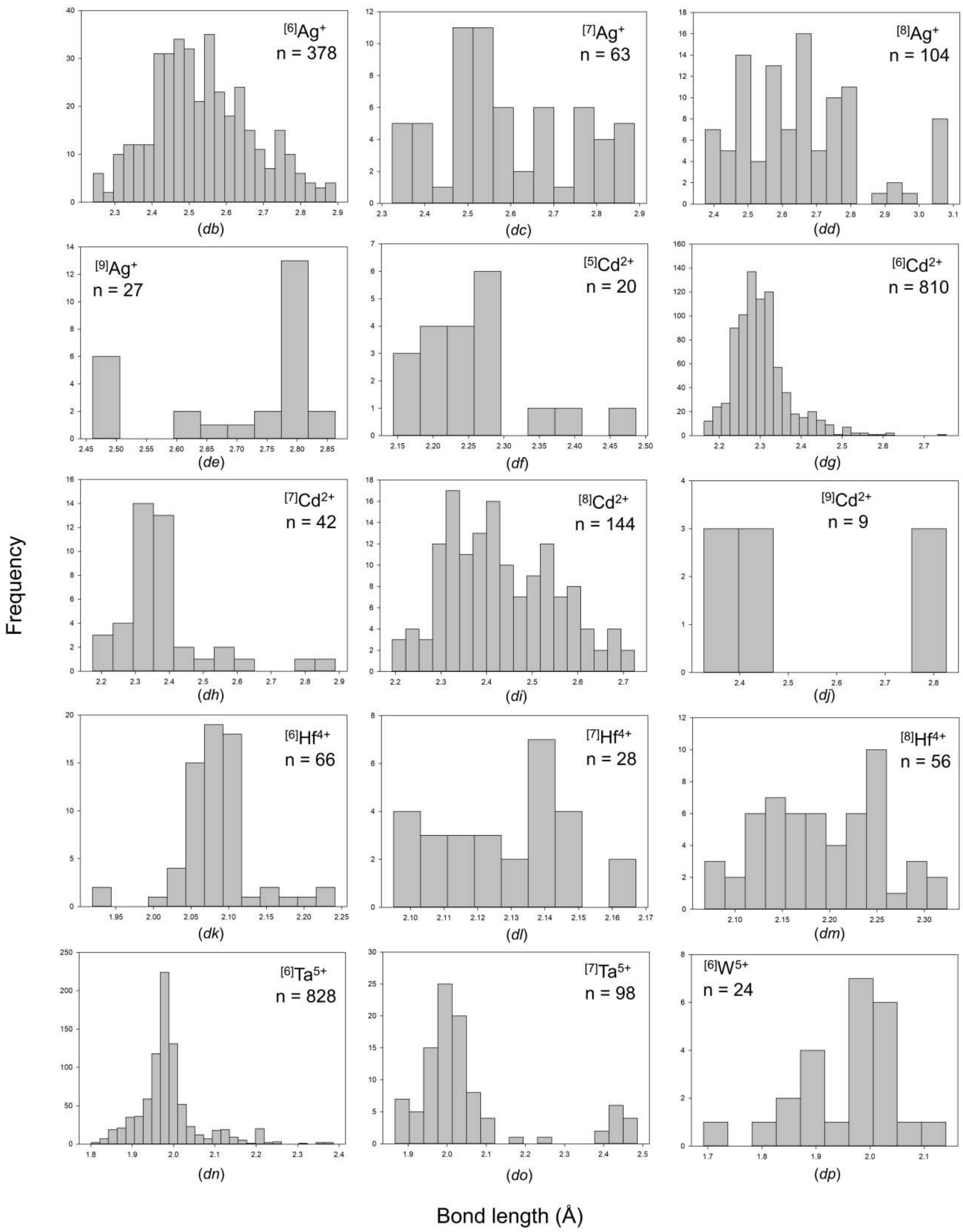

[6]Ag+
n = 378
(db)
[7]Ag+
n = 63
(dc)
[8]Ag+
n = 104
(dd)
[9]Ag+
n = 27
(de)
[5]Cd2+
n = 20
(df)
[6]Cd2+
n = 810
(dg)
[7]Cd2+
n = 42
(dh)
[8]Cd2+
n = 144
(di)
[9]Cd2+
n = 9
(dj)
[6]Hf4+
n = 66
(dk)
[7]Hf4+
n = 28
(dl)
[8]Hf4+
n = 56
(dm)
[6]Ta5+
n = 828
(dn)
[7]Ta5+
n = 98
(do)
[6]W5+
n = 24
(dp)
Frequency
Bond length (Å)

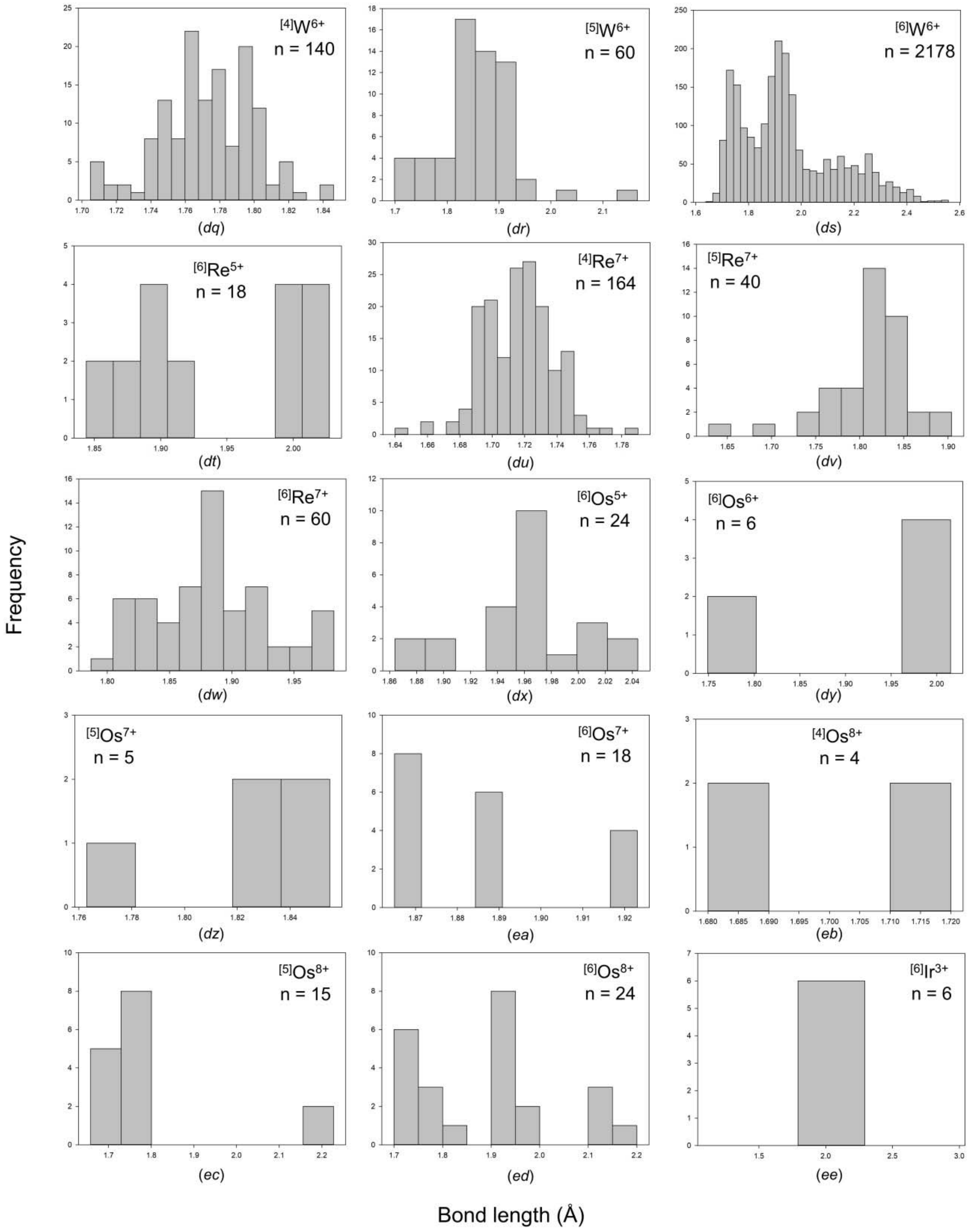
Frequency
Bond length (Å)
[4]W6+ n = 140
(dq)
[5]W6+ n = 60
(dr)
[6]W6+ n = 2178
(ds)
[6]Re5+ n = 18
(dt)
[4]Re7+ n = 164
(du)
[5]Re7+ n = 40
(dv)
[6]Re7+ n = 60
(dw)
[6]Os5+ n = 24
(dx)
[6]Os6+ n = 6
(dy)
[5]Os7+ n = 5
(dz)
[6]Os7+ n = 18
(ea)
[4]Os8+ n = 4
(eb)
[5]Os8+ n = 15
(ec)
[6]Os8+ n = 24
(ed)
[6]Ir3+ n = 6
(ee)

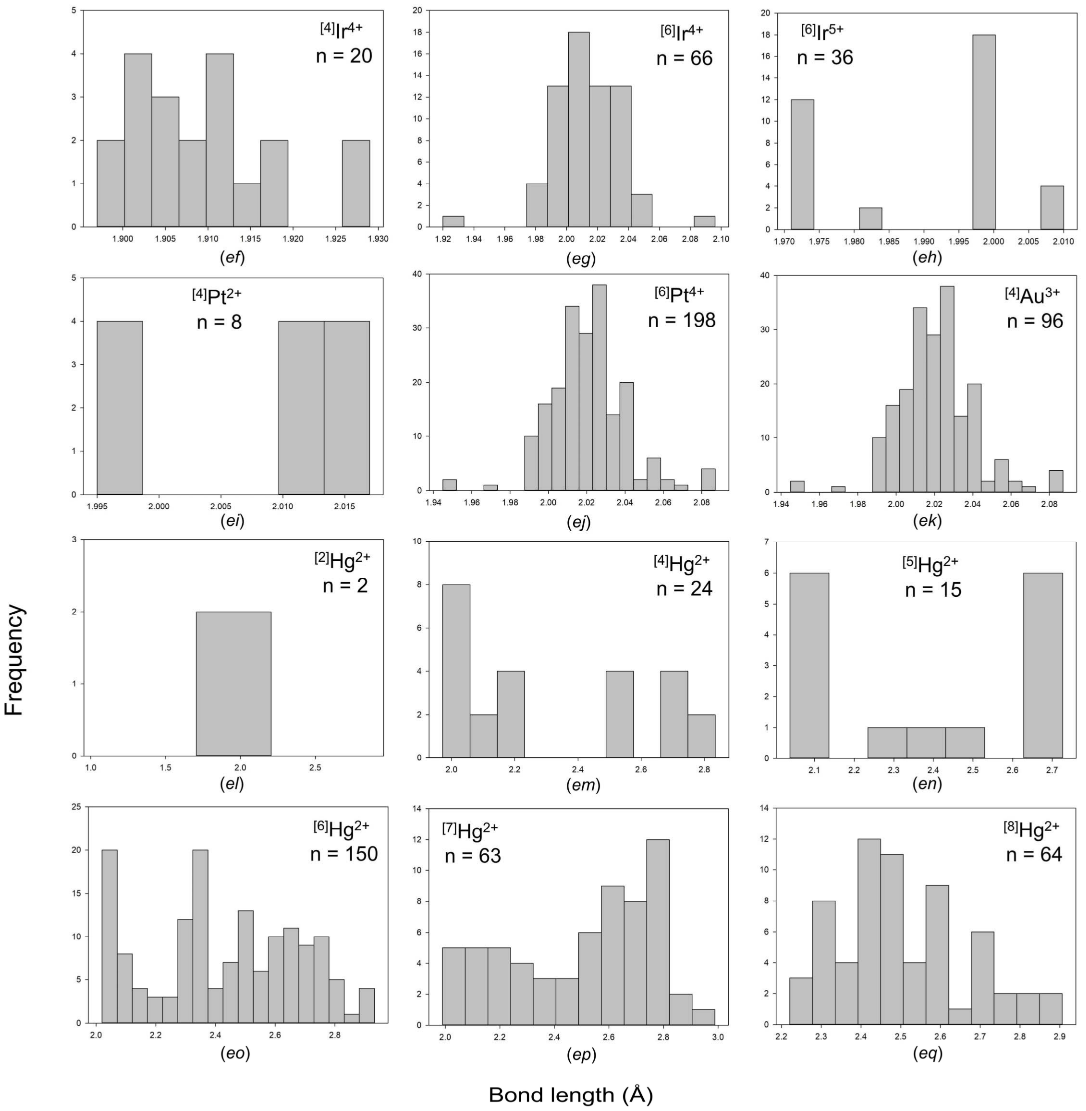

Figure S1 Bond-length distributions for all configurations of the transition metal ions bonded to O^{2-}: (*a*) $^{[6]}Sc^{3+}$, (*b*) $^{[7]}Sc^{3+}$, (*c*) $^{[8]}Sc^{3+}$, (*d*) $^{[6]}Ti^{3+}$, (*e*) $^{[7]}Ti^{3+}$, (*f*) $^{[8]}Ti^{3+}$, (*g*) $^{[4]}Ti^{4+}$, (*h*) $^{[5]}Ti^{4+}$, (*i*) $^{[6]}Ti^{4+}$, (*j*) $^{[7]}Ti^{4+}$, (*k*) $^{[6]}V^{3+}$, (*l*) $^{[5]}V^{4+}$, (*m*) $^{[6]}V^{4+}$, (*n*) $^{[4]}V^{5+}$, (*o*) $^{[5]}V^{5+}$, (*p*) $^{[6]}V^{5+}$, (*q*) $^{[4]}Cr^{2+}$, (*r*) $^{[5]}Cr^{2+}$, (*s*) $^{[6]}Cr^{2+}$, (*t*) $^{[6]}Cr^{3+}$, (*u*) $^{[4]}Cr^{4+}$, (*v*) $^{[6]}Cr^{4+}$, (*w*) $^{[4]}Cr^{5+}$, (*x*) $^{[4]}Cr^{6+}$, (*y*) $^{[4]}Mn^{2+}$, (*z*) $^{[5]}Mn^{2+}$, (*aa*) $^{[6]}Mn^{2+}$, (*ab*) $^{[7]}Mn^{2+}$, (*ac*) $^{[8]}Mn^{2+}$, (*ad*) $^{[4]}Mn^{3+}$, (*ae*) $^{[5]}Mn^{3+}$, (*af*) $^{[6]}Mn^{3+}$, (*ag*) $^{[4]}Mn^{4+}$, (*ah*) $^{[6]}Mn^{4+}$, (*ai*) $^{[4]}Mn^{5+}$, (*aj*) $^{[4]}Mn^{6+}$, (*ak*) $^{[4]}Mn^{7+}$, (*al*) $^{[3]}Fe^{2+}$, (*am*) $^{[4]}Fe^{2+}$, (*an*) $^{[5]}Fe^{2+}$, (*ao*) $^{[6]}Fe^{2+}$, (*ap*) $^{[8]}Fe^{2+}$, (*aq*) $^{[4]}Fe^{3+}$, (*ar*) $^{[5]}Fe^{3+}$, (*as*) $^{[6]}Fe^{3+}$, (*at*) $^{[8]}Fe^{3+}$, (*au*) $^{[3]}Co^{2+}$, (*av*) $^{[4]}Co^{2+}$, (*aw*) $^{[5]}Co^{2+}$, (*ax*) $^{[6]}Co^{2+}$, (*ay*) $^{[8]}Co^{2+}$, (*az*) $^{[6]}Co^{3+}$, (*ba*) $^{[6]}Co^{4+}$, (*bb*) $^{[2]}Ni^{2+}$, (*bc*) $^{[4]}Ni^{2+}$, (*bd*) $^{[5]}Ni^{2+}$, (*be*) $^{[6]}Ni^{2+}$, (*bf*) $^{[6]}Ni^{4+}$, (*bg*) $^{[2]}Cu^{+}$, (*bh*) $^{[3]}Cu^{+}$, (*bi*) $^{[4]}Cu^{+}$, (*bj*) $^{[4]}Cu^{2+}$, (*bk*) $^{[5]}Cu^{2+}$, (*bl*) $^{[6]}Cu^{2+}$, (*bm*) $^{[8]}Cu^{2+}$, (*bn*) $^{[4]}Cu^{3+}$, (*bo*) $^{[4]}Zn^{2+}$, (*bp*) $^{[5]}Zn^{2+}$, (*bq*) $^{[6]}Zn^{2+}$, (*br*) $^{[6]}Y^{3+}$, (*bs*) $^{[7]}Y^{3+}$, (*bt*) $^{[8]}Y^{3+}$,

(*bu*) $^{[9]}Y^{3+}$, (*bv*) $^{[10]}Y^{3+}$, (*bw*) $^{[12]}Y^{3+}$, (*bx*) $^{[6]}Zr^{4+}$, (*by*) $^{[7]}Zr^{4+}$, (*bz*) $^{[8]}Zr^{4+}$, (*ca*) $^{[9]}Zr^{4+}$, (*cb*) $^{[10]}Zr^{4+}$, (*cc*) $^{[6]}Nb^{4+}$, (*cd*) $^{[4]}Nb^{5+}$, (*ce*) $^{[5]}Nb^{5+}$, (*cf*) $^{[6]}Nb^{5+}$, (*cg*) $^{[7]}Nb^{5+}$, (*ch*) $^{[8]}Nb^{5+}$, (*ci*) $^{[6]}Mo^{3+}$, (*cj*) $^{[6]}Mo^{4+}$, (*ck*) $^{[5]}Mo^{5+}$, (*cl*) $^{[6]}Mo^{5+}$, (*cm*) $^{[4]}Mo^{6+}$, (*cn*) $^{[5]}Mo^{6+}$, (*co*) $^{[6]}Mo^{6+}$, (*cp*) $^{[4]}Tc^{7+}$, (*cq*) $^{[6]}Ru^{3+}$, (*cr*) $^{[6]}Ru^{4+}$, (*cs*) $^{[6]}Ru^{5+}$, (*ct*) $^{[6]}Rh^{3+}$, (*cu*) $^{[6]}Rh^{4+}$, (*cv*) $^{[4]}Pd^{2+}$, (*cw*) $^{[6]}Pd^{4+}$, (*cx*) $^{[2]}Ag^{+}$, (*cy*) $^{[3]}Ag^{+}$, (*cz*) $^{[4]}Ag^{+}$, (*da*) $^{[5]}Ag^{+}$, (*db*) $^{[6]}Ag^{+}$, (*dc*) $^{[7]}Ag^{+}$, (*dd*) $^{[8]}Ag^{+}$, (*de*) $^{[9]}Ag^{+}$, (*df*) $^{[5]}Cd^{2+}$, (*dg*) $^{[6]}Cd^{2+}$, (*dh*) $^{[7]}Cd^{2+}$, (*di*) $^{[8]}Cd^{2+}$, (*dj*) $^{[9]}Cd^{2+}$, (*dk*) $^{[6]}Hf^{4+}$, (*dl*) $^{[7]}Hf^{4+}$, (*dm*) $^{[8]}Hf^{4+}$, (*dn*) $^{[6]}Ta^{5+}$, (*do*) $^{[7]}Ta^{5+}$, (*dp*) $^{[6]}W^{5+}$, (*dq*) $^{[4]}W^{6+}$, (*dr*) $^{[5]}W^{6+}$, (*ds*) $^{[6]}W^{6+}$, (*dt*) $^{[6]}Re^{5+}$, (*du*) $^{[4]}Re^{7+}$, (*dv*) $^{[5]}Re^{7+}$, (*dw*) $^{[6]}Re^{7+}$, (*dx*) $^{[6]}Os^{5+}$, (*dy*) $^{[6]}Os^{6+}$, (*dz*) $^{[5]}Os^{7+}$, (*ea*) $^{[6]}Os^{7+}$, (*eb*) $^{[4]}Os^{8+}$, (*ec*) $^{[5]}Os^{8+}$, (*ed*) $^{[6]}Os^{8+}$, (*ee*) $^{[6]}Ir^{3+}$, (*ef*) $^{[4]}Ir^{4+}$, (*eg*) $^{[6]}Ir^{4+}$, (*eh*) $^{[6]}Ir^{5+}$, (*ei*) $^{[4]}Pt^{2+}$, (*ej*) $^{[6]}Pt^{4+}$, (*ek*) $^{[4]}Au^{3+}$, (*el*) $^{[2]}Hg^{2+}$, (*em*) $^{[4]}Hg^{2+}$, (*en*) $^{[5]}Hg^{2+}$, (*eo*) $^{[6]}Hg^{2+}$, (*ep*) $^{[7]}Hg^{2+}$, (*eq*) $^{[8]}Hg^{2+}$.

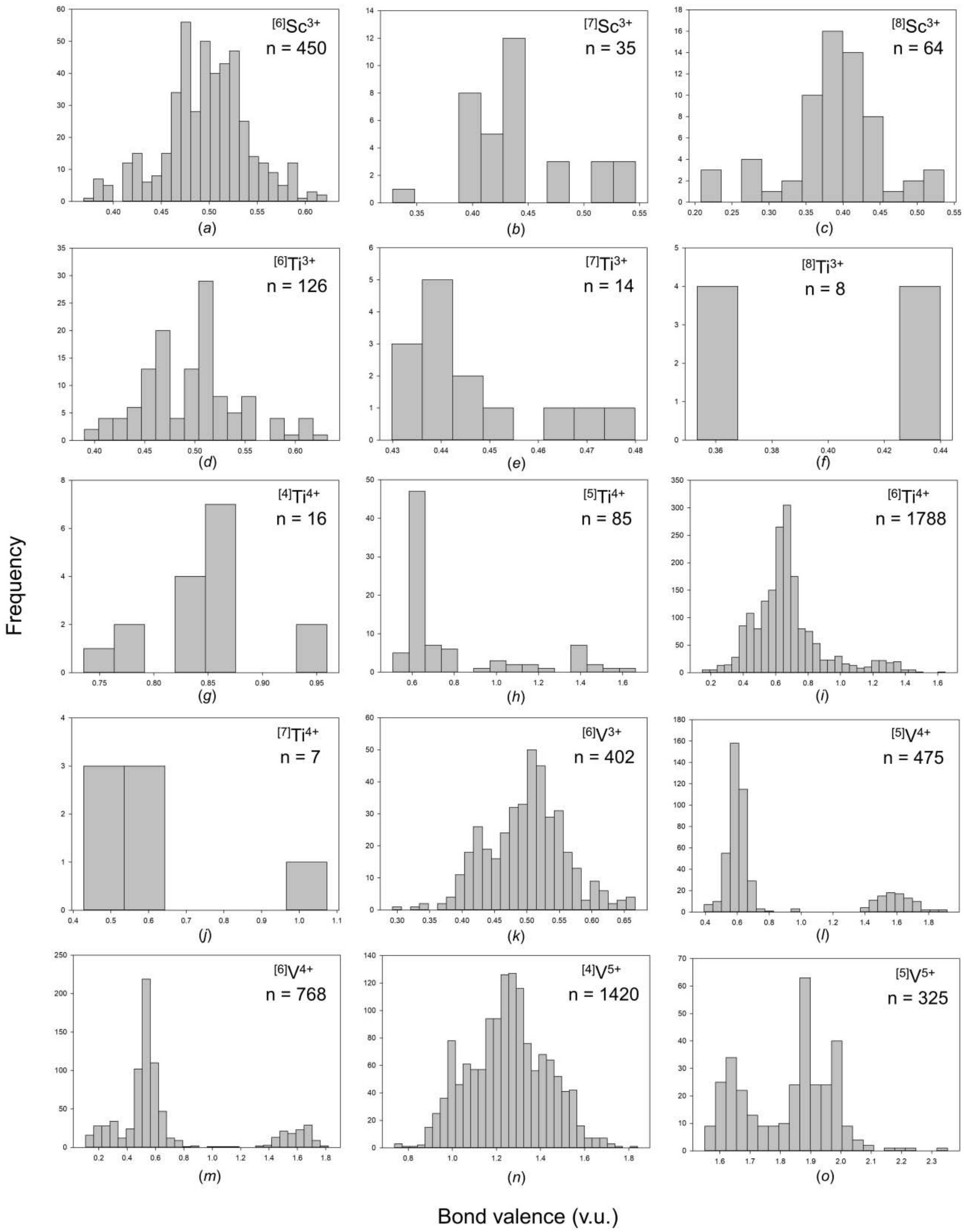
[6]Sc3+ n = 450
(a)
[7]Sc3+ n = 35
(b)
[8]Sc3+ n = 64
(c)
[6]Ti3+ n = 126
(d)
[7]Ti3+ n = 14
(e)
[8]Ti3+ n = 8
(f)
[4]Ti4+ n = 16
(g)
[5]Ti4+ n = 85
(h)
[6]Ti4+ n = 1788
(i)
[7]Ti4+ n = 7
(j)
[6]V3+ n = 402
(k)
[5]V4+ n = 475
(l)
[6]V4+ n = 768
(m)
[4]V5+ n = 1420
(n)
[5]V5+ n = 325
(o)
Frequency
Bond valence (v.u.)

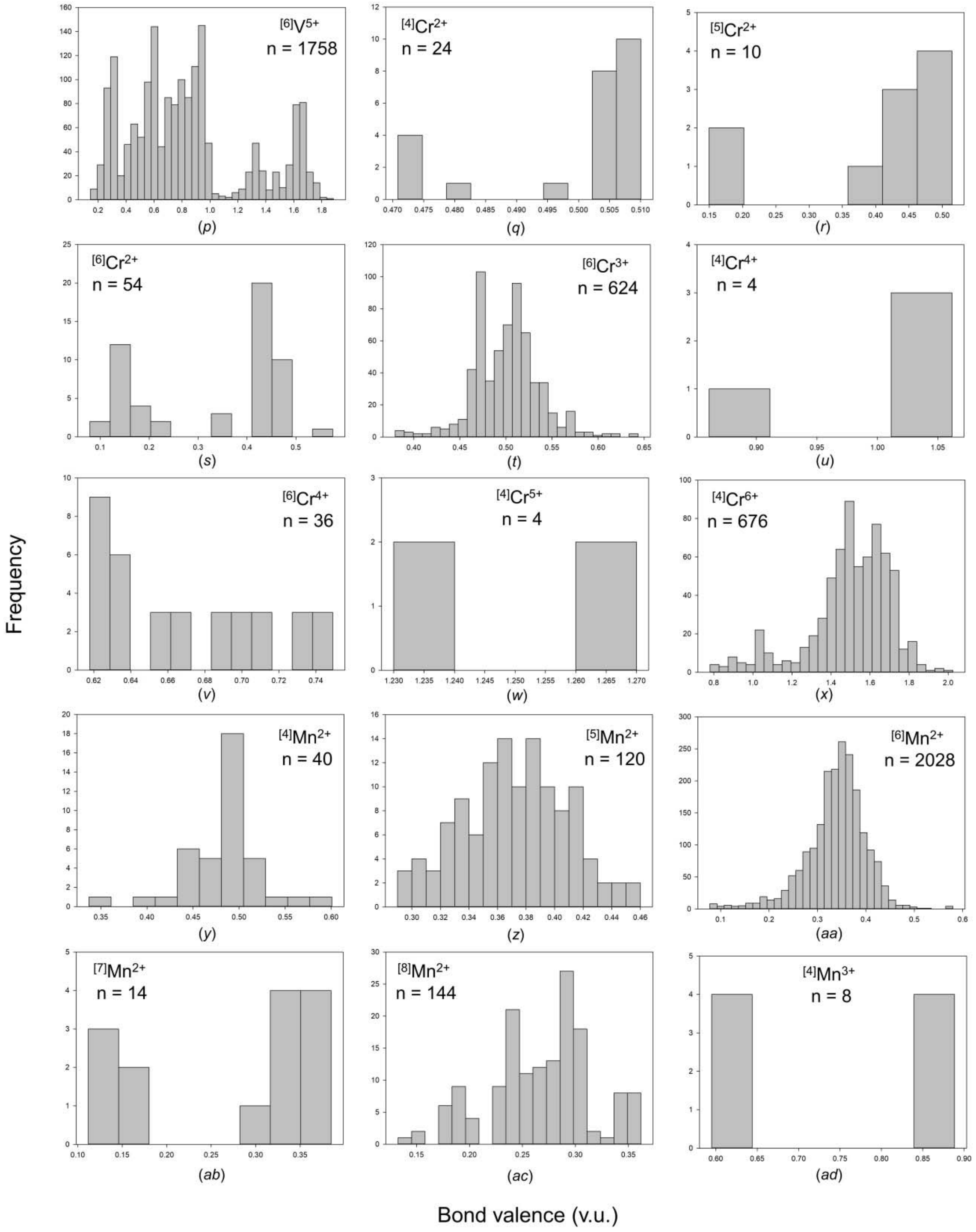
[6]V5+
n = 1758
(p)
[4]Cr2+
n = 24
(q)
[5]Cr2+
n = 10
(r)
[6]Cr2+
n = 54
(s)
[6]Cr3+
n = 624
(t)
[4]Cr4+
n = 4
(u)
[6]Cr4+
n = 36
(v)
[4]Cr5+
n = 4
(w)
[4]Cr6+
n = 676
(x)
[4]Mn2+
n = 40
(y)
[5]Mn2+
n = 120
(z)
[6]Mn2+
n = 2028
(aa)
[7]Mn2+
n = 14
(ab)
[8]Mn2+
n = 144
(ac)
[4]Mn3+
n = 8
(ad)
Frequency
Bond valence (v.u.)

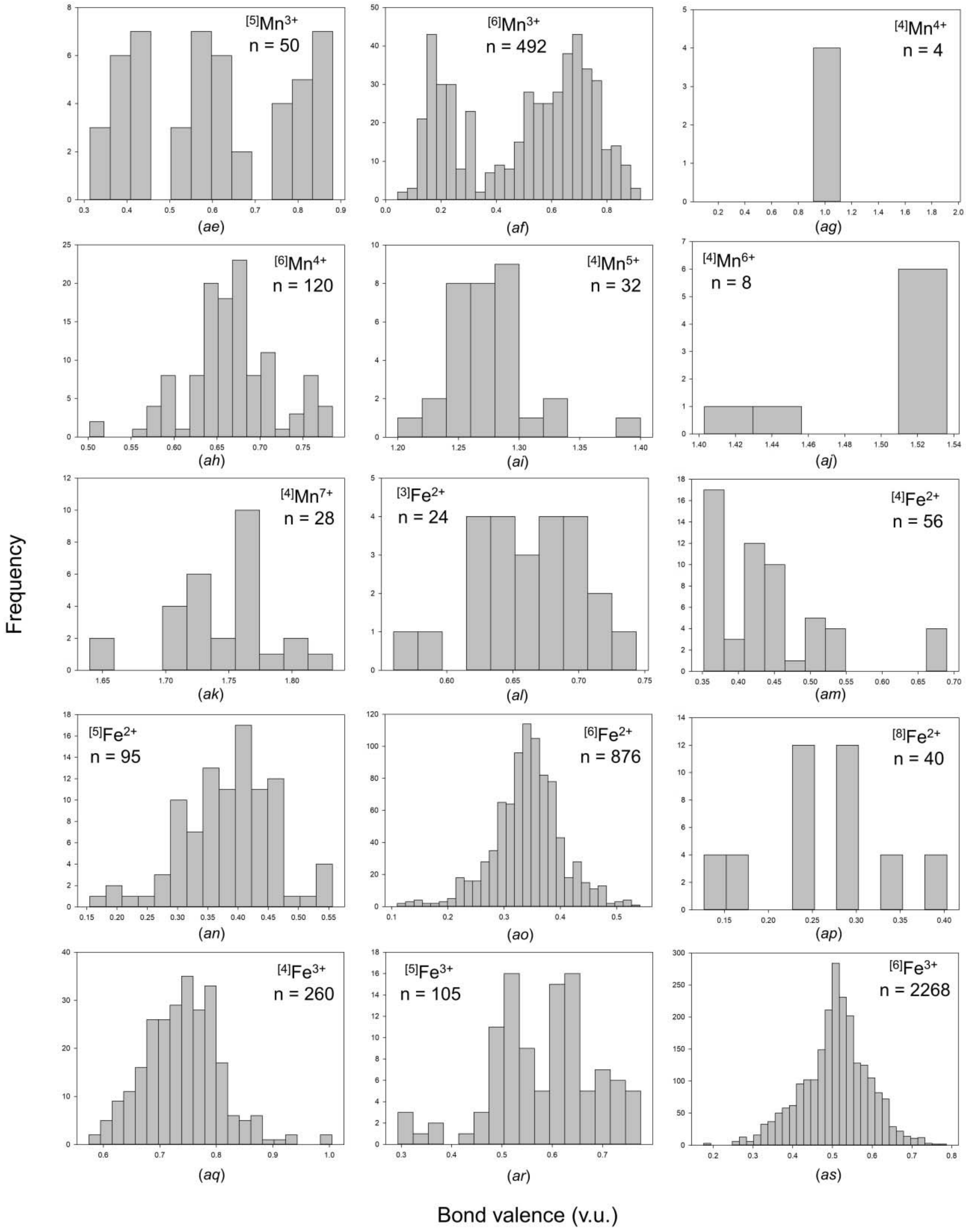
[5]Mn3+
n = 50
(ae)
[6]Mn3+
n = 492
(af)
[4]Mn4+
n = 4
(ag)
[6]Mn4+
n = 120
(ah)
[4]Mn5+
n = 32
(ai)
[4]Mn6+
n = 8
(aj)
[4]Mn7+
n = 28
(ak)
[3]Fe2+
n = 24
(al)
[4]Fe2+
n = 56
(am)
[5]Fe2+
n = 95
(an)
[6]Fe2+
n = 876
(ao)
[8]Fe2+
n = 40
(ap)
[4]Fe3+
n = 260
(aq)
[5]Fe3+
n = 105
(ar)
[6]Fe3+
n = 2268
(as)
Frequency
Bond valence (v.u.)

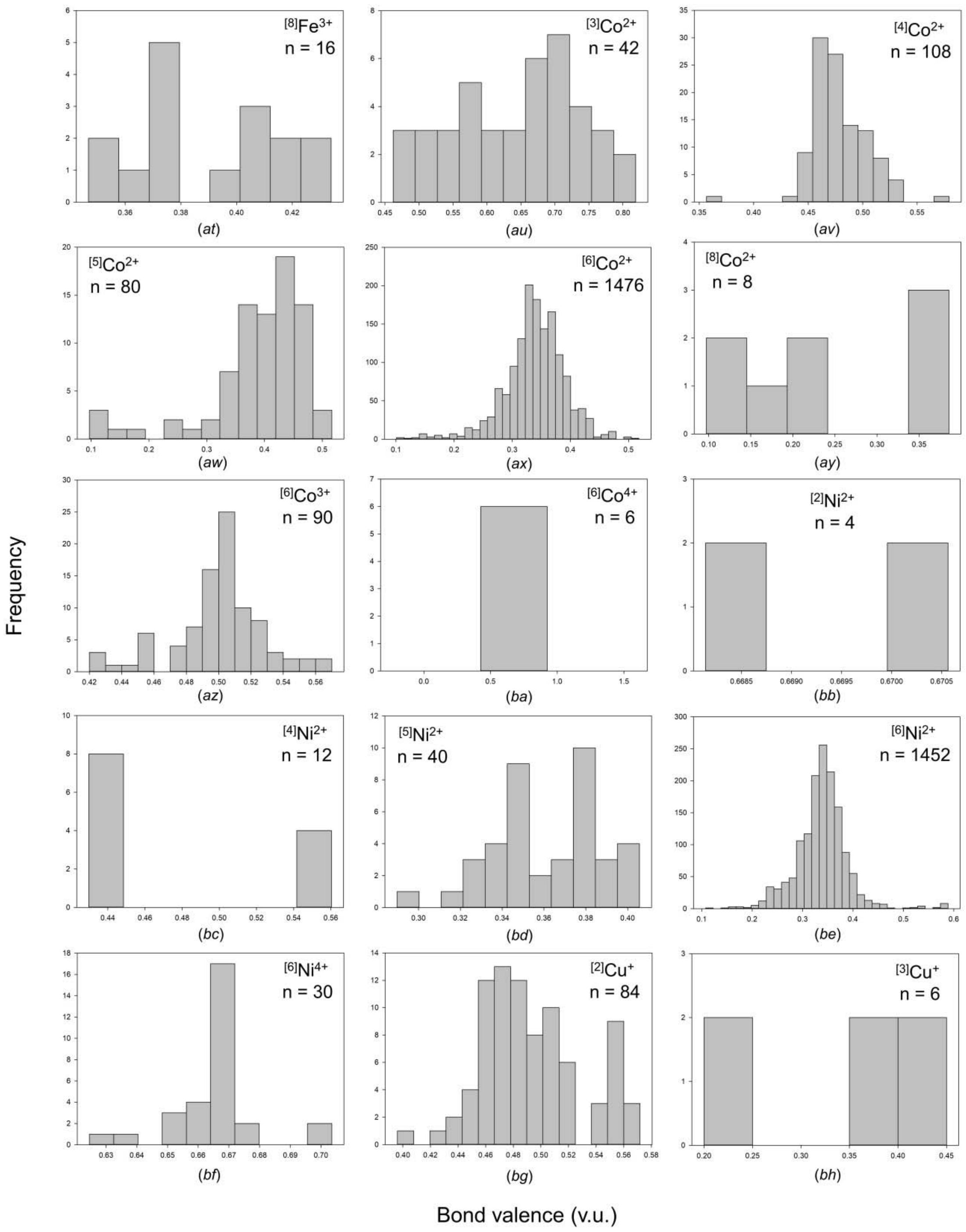
[8]Fe3+ n = 16
(at)
[3]Co2+ n = 42
(au)
[4]Co2+ n = 108
(av)
[5]Co2+ n = 80
(aw)
[6]Co2+ n = 1476
(ax)
[8]Co2+ n = 8
(ay)
[6]Co3+ n = 90
(az)
[6]Co4+ n = 6
(ba)
[2]Ni2+ n = 4
(bb)
[4]Ni2+ n = 12
(bc)
[5]Ni2+ n = 40
(bd)
[6]Ni2+ n = 1452
(be)
[6]Ni4+ n = 30
(bf)
[2]Cu+ n = 84
(bg)
[3]Cu+ n = 6
(bh)
Frequency
Bond valence (v.u.)

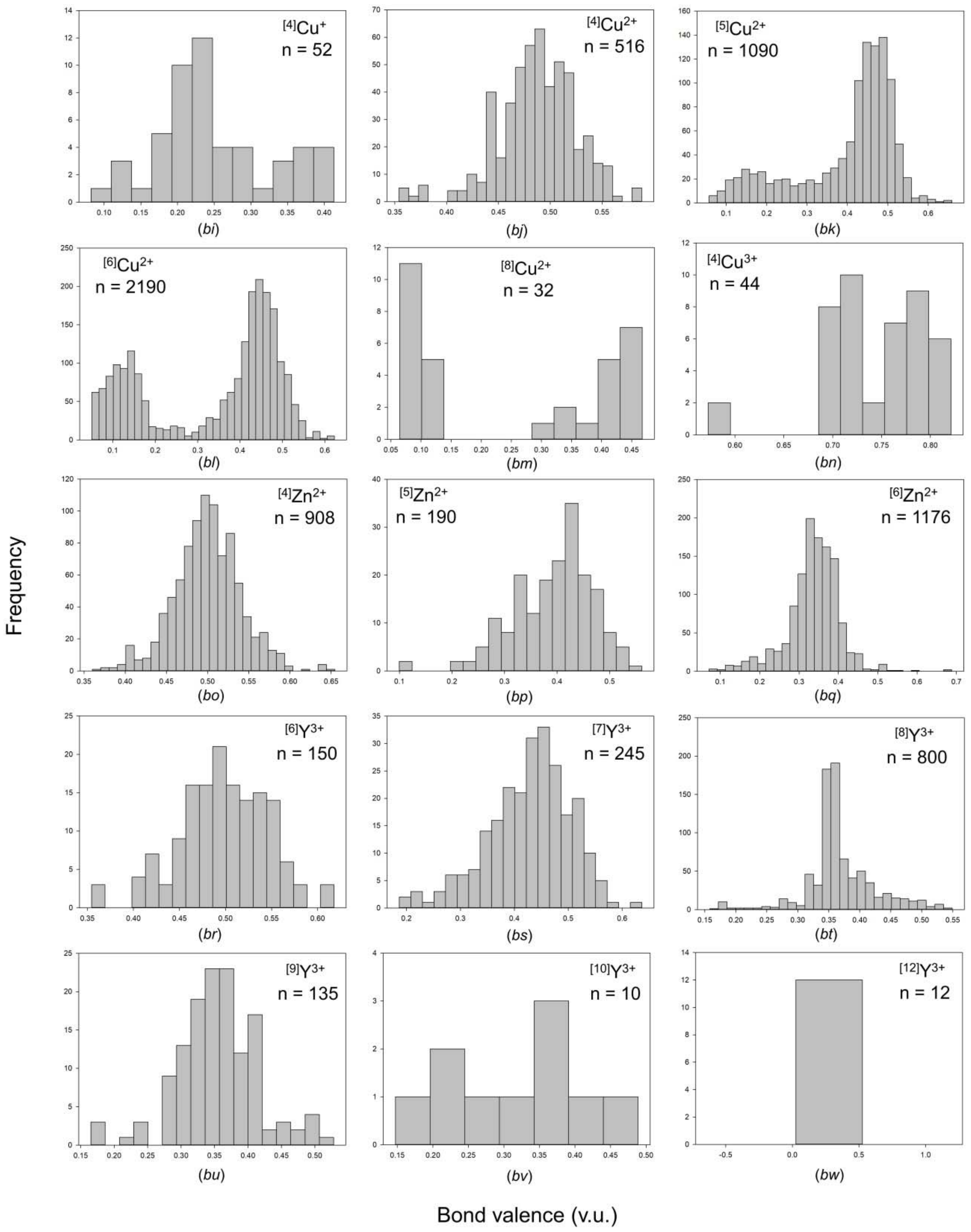
[4]Cu+
n = 52
(bi)
[4]Cu2+
n = 516
(bj)
[5]Cu2+
n = 1090
(bk)
[6]Cu2+
n = 2190
(bl)
[8]Cu2+
n = 32
(bm)
[4]Cu3+
n = 44
(bn)
[4]Zn2+
n = 908
(bo)
[5]Zn2+
n = 190
(bp)
[6]Zn2+
n = 1176
(bq)
[6]Y3+
n = 150
(br)
[7]Y3+
n = 245
(bs)
[8]Y3+
n = 800
(bt)
[9]Y3+
n = 135
(bu)
[10]Y3+
n = 10
(bv)
[12]Y3+
n = 12
(bw)
Frequency
Bond valence (v.u.)

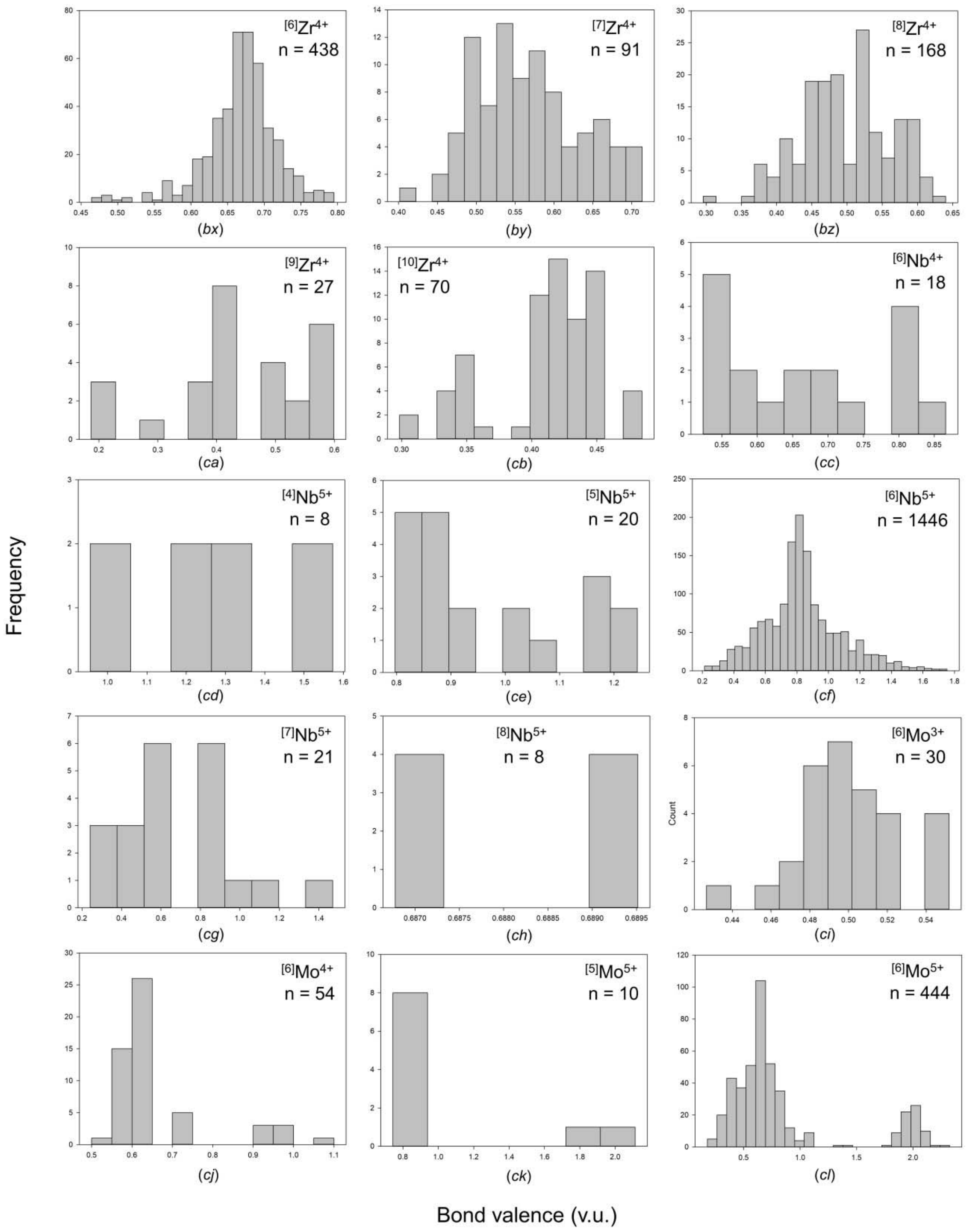
[6]Zr4+
n = 438
(bx)
[7]Zr4+
n = 91
(by)
[8]Zr4+
n = 168
(bz)
[9]Zr4+
n = 27
(ca)
[10]Zr4+
n = 70
(cb)
[6]Nb4+
n = 18
(cc)
[4]Nb5+
n = 8
(cd)
[5]Nb5+
n = 20
(ce)
[6]Nb5+
n = 1446
(cf)
[7]Nb5+
n = 21
(cg)
[8]Nb5+
n = 8
(ch)
[6]Mo3+
n = 30
Count
(ci)
[6]Mo4+
n = 54
(cj)
[5]Mo5+
n = 10
(ck)
[6]Mo5+
n = 444
(cl)
Frequency
Bond valence (v.u.)

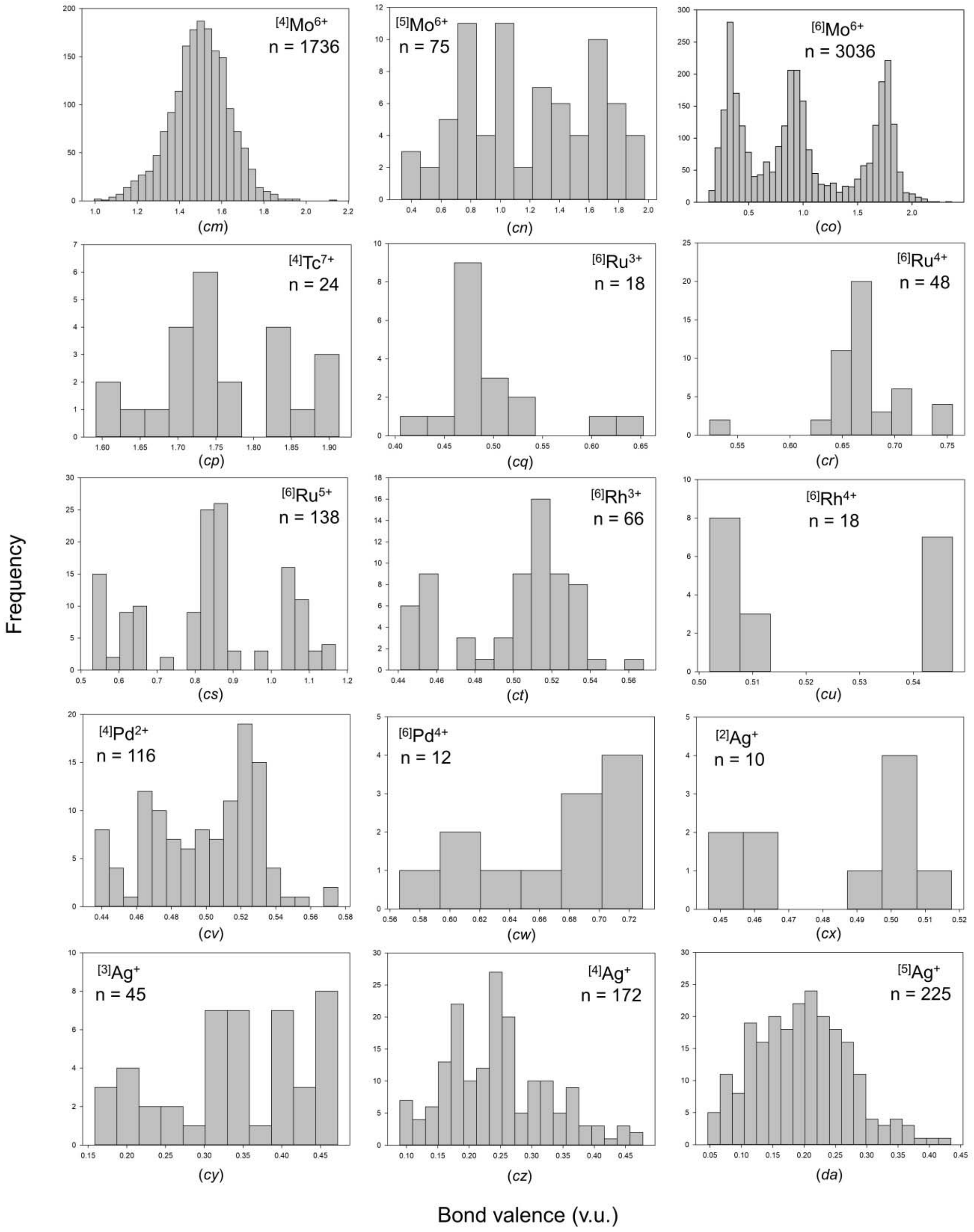
[4]Mo6+
n = 1736
(cm)
[5]Mo6+
n = 75
(cn)
[6]Mo6+
n = 3036
(co)
[4]Tc7+
n = 24
(cp)
[6]Ru3+
n = 18
(cq)
[6]Ru4+
n = 48
(cr)
[6]Ru5+
n = 138
(cs)
[6]Rh3+
n = 66
(ct)
[6]Rh4+
n = 18
(cu)
[4]Pd2+
n = 116
(cv)
[6]Pd4+
n = 12
(cw)
[2]Ag+
n = 10
(cx)
[3]Ag+
n = 45
(cy)
[4]Ag+
n = 172
(cz)
[5]Ag+
n = 225
(da)
Frequency
Bond valence (v.u.)

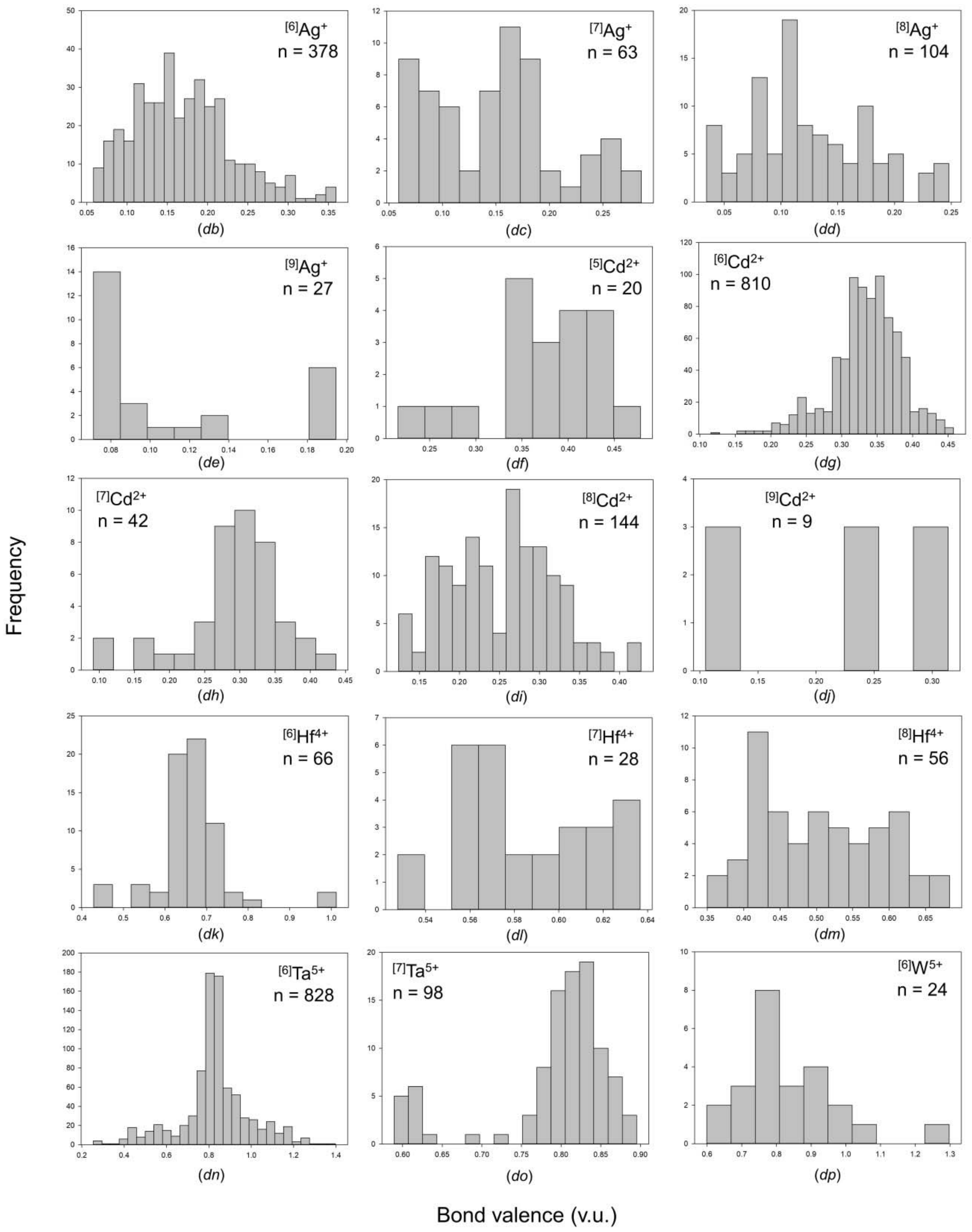
[6]Ag+
n = 378
(db)
[7]Ag+
n = 63
(dc)
[8]Ag+
n = 104
(dd)
[9]Ag+
n = 27
(de)
[5]Cd2+
n = 20
(df)
[6]Cd2+
n = 810
(dg)
[7]Cd2+
n = 42
(dh)
[8]Cd2+
n = 144
(di)
[9]Cd2+
n = 9
(dj)
[6]Hf4+
n = 66
(dk)
[7]Hf4+
n = 28
(dl)
[8]Hf4+
n = 56
(dm)
[6]Ta5+
n = 828
(dn)
[7]Ta5+
n = 98
(do)
[6]W5+
n = 24
(dp)
Frequency
Bond valence (v.u.)

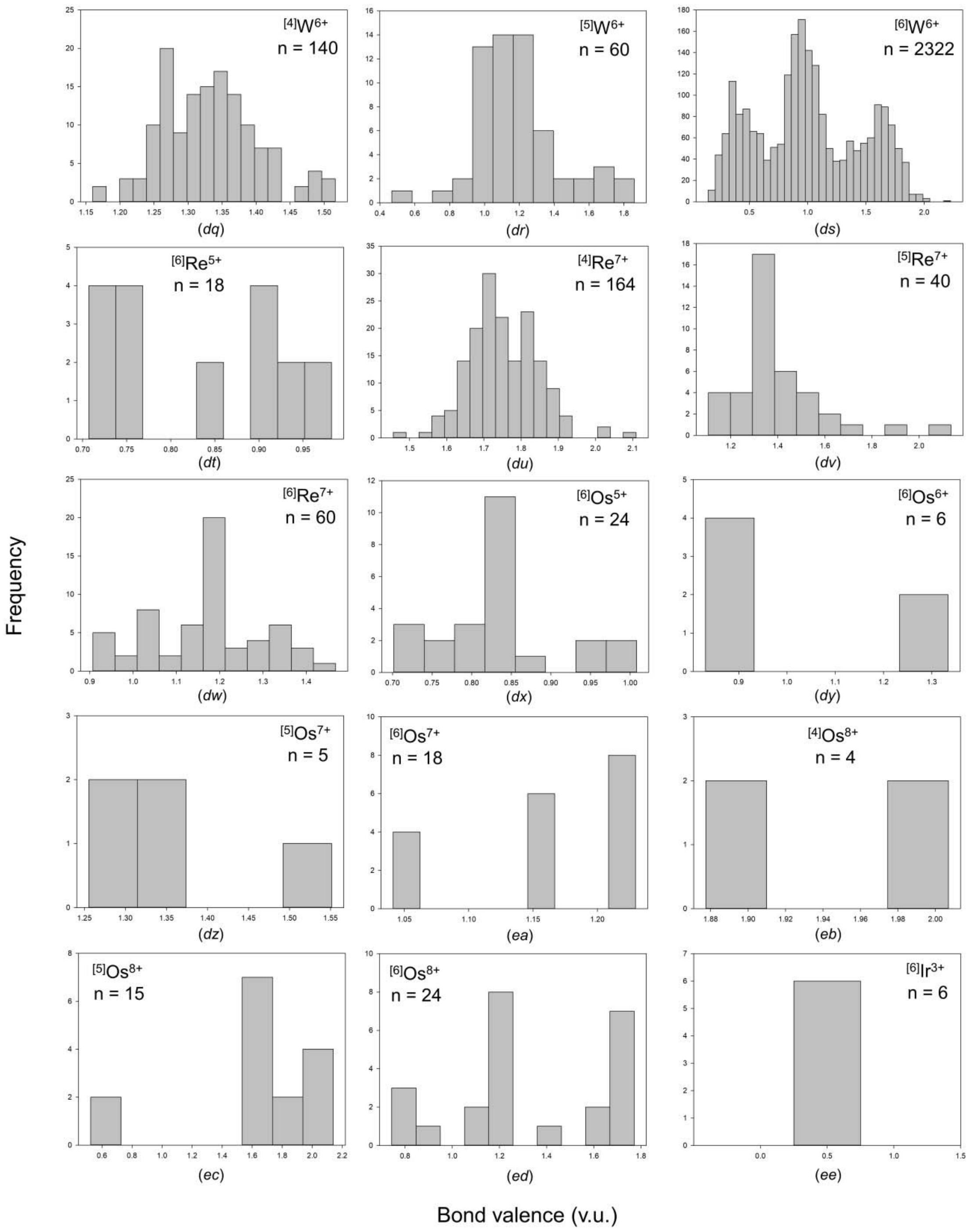

Frequency
Bond valence (v.u.)
[4]W6+ n = 140
(dq)
[5]W6+ n = 60
(dr)
[6]W6+ n = 2322
(ds)
[6]Re5+ n = 18
(dt)
[4]Re7+ n = 164
(du)
[5]Re7+ n = 40
(dv)
[6]Re7+ n = 60
(dw)
[6]Os5+ n = 24
(dx)
[6]Os6+ n = 6
(dy)
[5]Os7+ n = 5
(dz)
[6]Os7+ n = 18
(ea)
[4]Os8+ n = 4
(eb)
[5]Os8+ n = 15
(ec)
[6]Os8+ n = 24
(ed)
[6]Ir3+ n = 6
(ee)

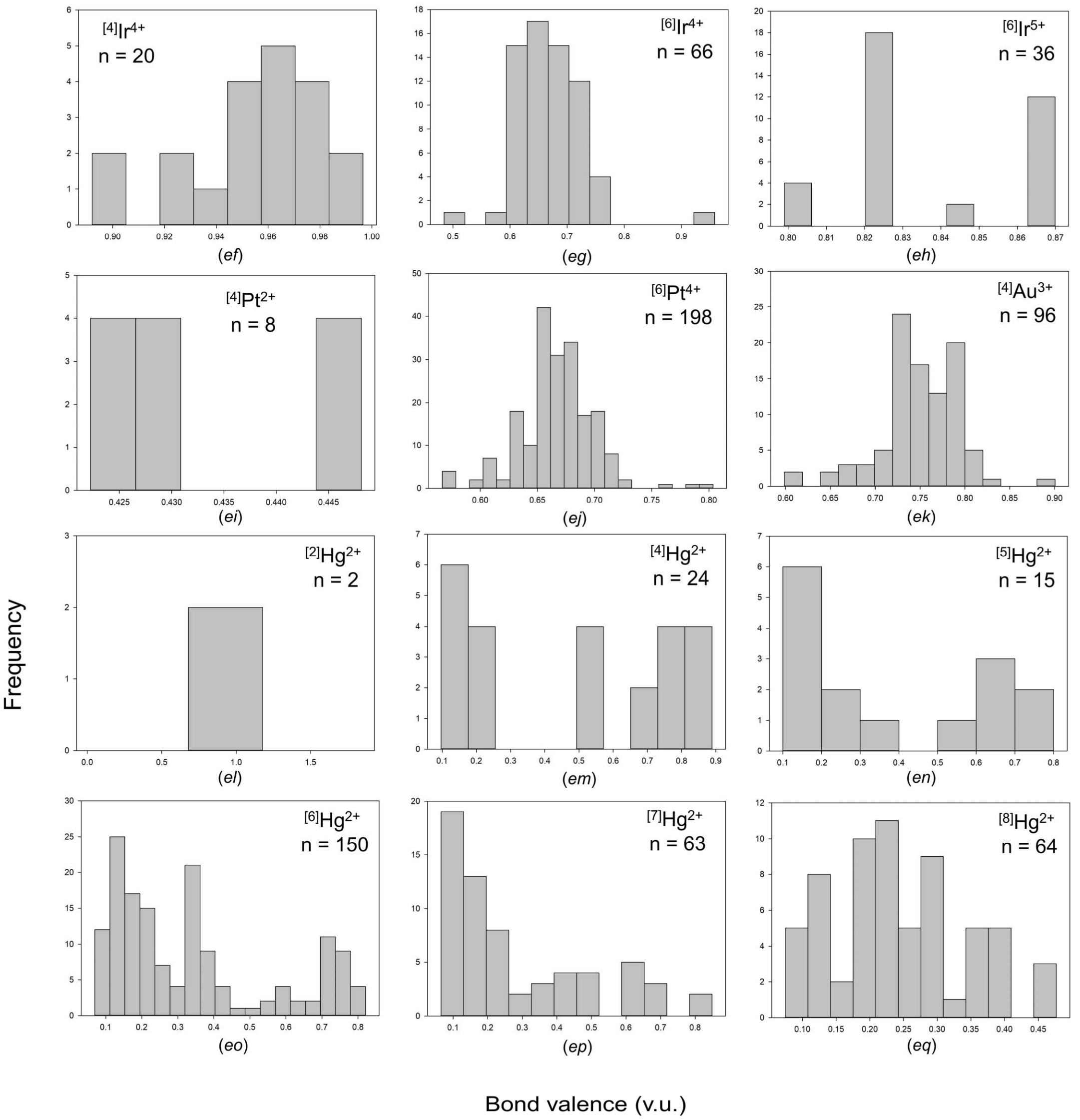

Figure S2 Bond-valence distributions for all configurations of the transition metal ions bonded to O^{2-}: (*a*) $^{[6]}Sc^{3+}$, (*b*) $^{[7]}Sc^{3+}$, (*c*) $^{[8]}Sc^{3+}$, (*d*) $^{[6]}Ti^{3+}$, (*e*) $^{[7]}Ti^{3+}$, (*f*) $^{[8]}Ti^{3+}$, (*g*) $^{[4]}Ti^{4+}$, (*h*) $^{[5]}Ti^{4+}$, (*i*) $^{[6]}Ti^{4+}$, (*j*) $^{[7]}Ti^{4+}$, (*k*) $^{[6]}V^{3+}$, (*l*) $^{[5]}V^{4+}$, (*m*) $^{[6]}V^{4+}$, (*n*) $^{[4]}V^{5+}$, (*o*) $^{[5]}V^{5+}$, (*p*) $^{[6]}V^{5+}$, (*q*) $^{[4]}Cr^{2+}$, (*r*) $^{[5]}Cr^{2+}$, (*s*) $^{[6]}Cr^{2+}$, (*t*) $^{[6]}Cr^{3+}$, (*u*) $^{[4]}Cr^{4+}$, (*v*) $^{[6]}Cr^{4+}$, (*w*) $^{[4]}Cr^{5+}$, (*x*) $^{[4]}Cr^{6+}$, (*y*) $^{[4]}Mn^{2+}$, (*z*) $^{[5]}Mn^{2+}$, (*aa*) $^{[6]}Mn^{2+}$, (*ab*) $^{[7]}Mn^{2+}$, (*ac*) $^{[8]}Mn^{2+}$, (*ad*) $^{[4]}Mn^{3+}$, (*ae*) $^{[5]}Mn^{3+}$, (*af*) $^{[6]}Mn^{3+}$, (*ag*) $^{[4]}Mn^{4+}$, (*ah*) $^{[6]}Mn^{4+}$, (*ai*) $^{[4]}Mn^{5+}$, (*aj*) $^{[4]}Mn^{6+}$, (*ak*) $^{[4]}Mn^{7+}$, (*al*) $^{[3]}Fe^{2+}$, (*am*) $^{[4]}Fe^{2+}$, (*an*) $^{[5]}Fe^{2+}$, (*ao*) $^{[6]}Fe^{2+}$, (*ap*) $^{[8]}Fe^{2+}$, (*aq*) $^{[4]}Fe^{3+}$, (*ar*) $^{[5]}Fe^{3+}$, (*as*) $^{[6]}Fe^{3+}$, (*at*) $^{[8]}Fe^{3+}$, (*au*) $^{[3]}Co^{2+}$, (*av*) $^{[4]}Co^{2+}$, (*aw*) $^{[5]}Co^{2+}$, (*ax*) $^{[6]}Co^{2+}$, (*ay*) $^{[8]}Co^{2+}$, (*az*) $^{[6]}Co^{3+}$, (*ba*) $^{[6]}Co^{4+}$, (*bb*) $^{[2]}Ni^{2+}$, (*bc*) $^{[4]}Ni^{2+}$, (*bd*) $^{[5]}Ni^{2+}$, (*be*) $^{[6]}Ni^{2+}$, (*bf*) $^{[6]}Ni^{4+}$, (*bg*) $^{[2]}Cu^{+}$, (*bh*) $^{[3]}Cu^{+}$, (*bi*) $^{[4]}Cu^{+}$, (*bj*) $^{[4]}Cu^{2+}$, (*bk*) $^{[5]}Cu^{2+}$, (*bl*) $^{[6]}Cu^{2+}$, (*bm*) $^{[8]}Cu^{2+}$, (*bn*) $^{[4]}Cu^{3+}$, (*bo*) $^{[4]}Zn^{2+}$, (*bp*) $^{[5]}Zn^{2+}$, (*bq*) $^{[6]}Zn^{2+}$, (*br*) $^{[6]}Y^{3+}$, (*bs*) $^{[7]}Y^{3+}$, (*bt*) $^{[8]}Y^{3+}$,

(*bu*) $^{[9]}Y^{3+}$, (*bv*) $^{[10]}Y^{3+}$, (*bw*) $^{[12]}Y^{3+}$, (*bx*) $^{[6]}Zr^{4+}$, (*by*) $^{[7]}Zr^{4+}$, (*bz*) $^{[8]}Zr^{4+}$, (*ca*) $^{[9]}Zr^{4+}$, (*cb*) $^{[10]}Zr^{4+}$, (*cc*) $^{[6]}Nb^{4+}$, (*cd*) $^{[4]}Nb^{5+}$, (*ce*) $^{[5]}Nb^{5+}$, (*cf*) $^{[6]}Nb^{5+}$, (*cg*) $^{[7]}Nb^{5+}$, (*ch*) $^{[8]}Nb^{5+}$, (*ci*) $^{[6]}Mo^{3+}$, (*cj*) $^{[6]}Mo^{4+}$, (*ck*) $^{[5]}Mo^{5+}$, (*cl*) $^{[6]}Mo^{5+}$, (*cm*) $^{[4]}Mo^{6+}$, (*cn*) $^{[5]}Mo^{6+}$, (*co*) $^{[6]}Mo^{6+}$, (*cp*) $^{[4]}Tc^{7+}$, (*cq*) $^{[6]}Ru^{3+}$, (*cr*) $^{[6]}Ru^{4+}$, (*cs*) $^{[6]}Ru^{5+}$, (*ct*) $^{[6]}Rh^{3+}$, (*cu*) $^{[6]}Rh^{4+}$, (*cv*) $^{[4]}Pd^{2+}$, (*cw*) $^{[6]}Pd^{4+}$, (*cx*) $^{[2]}Ag^{+}$, (*cy*) $^{[3]}Ag^{+}$, (*cz*) $^{[4]}Ag^{+}$, (*da*) $^{[5]}Ag^{+}$, (*db*) $^{[6]}Ag^{+}$, (*dc*) $^{[7]}Ag^{+}$, (*dd*) $^{[8]}Ag^{+}$, (*de*) $^{[9]}Ag^{+}$, (*df*) $^{[5]}Cd^{2+}$, (*dg*) $^{[6]}Cd^{2+}$, (*dh*) $^{[7]}Cd^{2+}$, (*di*) $^{[8]}Cd^{2+}$, (*dj*) $^{[9]}Cd^{2+}$, (*dk*) $^{[6]}Hf^{4+}$, (*dl*) $^{[7]}Hf^{4+}$, (*dm*) $^{[8]}Hf^{4+}$, (*dn*) $^{[6]}Ta^{5+}$, (*do*) $^{[7]}Ta^{5+}$, (*dp*) $^{[6]}W^{5+}$, (*dq*) $^{[4]}W^{6+}$, (*dr*) $^{[5]}W^{6+}$, (*ds*) $^{[6]}W^{6+}$, (*dt*) $^{[6]}Re^{5+}$, (*du*) $^{[4]}Re^{7+}$, (*dv*) $^{[5]}Re^{7+}$, (*dw*) $^{[6]}Re^{7+}$, (*dx*) $^{[6]}Os^{5+}$, (*dy*) $^{[6]}Os^{6+}$, (*dz*) $^{[5]}Os^{7+}$, (*ea*) $^{[6]}Os^{7+}$, (*eb*) $^{[4]}Os^{8+}$, (*ec*) $^{[5]}Os^{8+}$, (*ed*) $^{[6]}Os^{8+}$, (*ee*) $^{[6]}Ir^{3+}$, (*ef*) $^{[4]}Ir^{4+}$, (*eg*) $^{[6]}Ir^{4+}$, (*eh*) $^{[6]}Ir^{5+}$, (*ei*) $^{[4]}Pt^{2+}$, (*ej*) $^{[6]}Pt^{4+}$, (*ek*) $^{[4]}Au^{3+}$, (*el*) $^{[2]}Hg^{2+}$, (*em*) $^{[4]}Hg^{2+}$, (*en*) $^{[5]}Hg^{2+}$, (*eo*) $^{[6]}Hg^{2+}$, (*ep*) $^{[7]}Hg^{2+}$, (*eq*) $^{[8]}Hg^{2+}$.

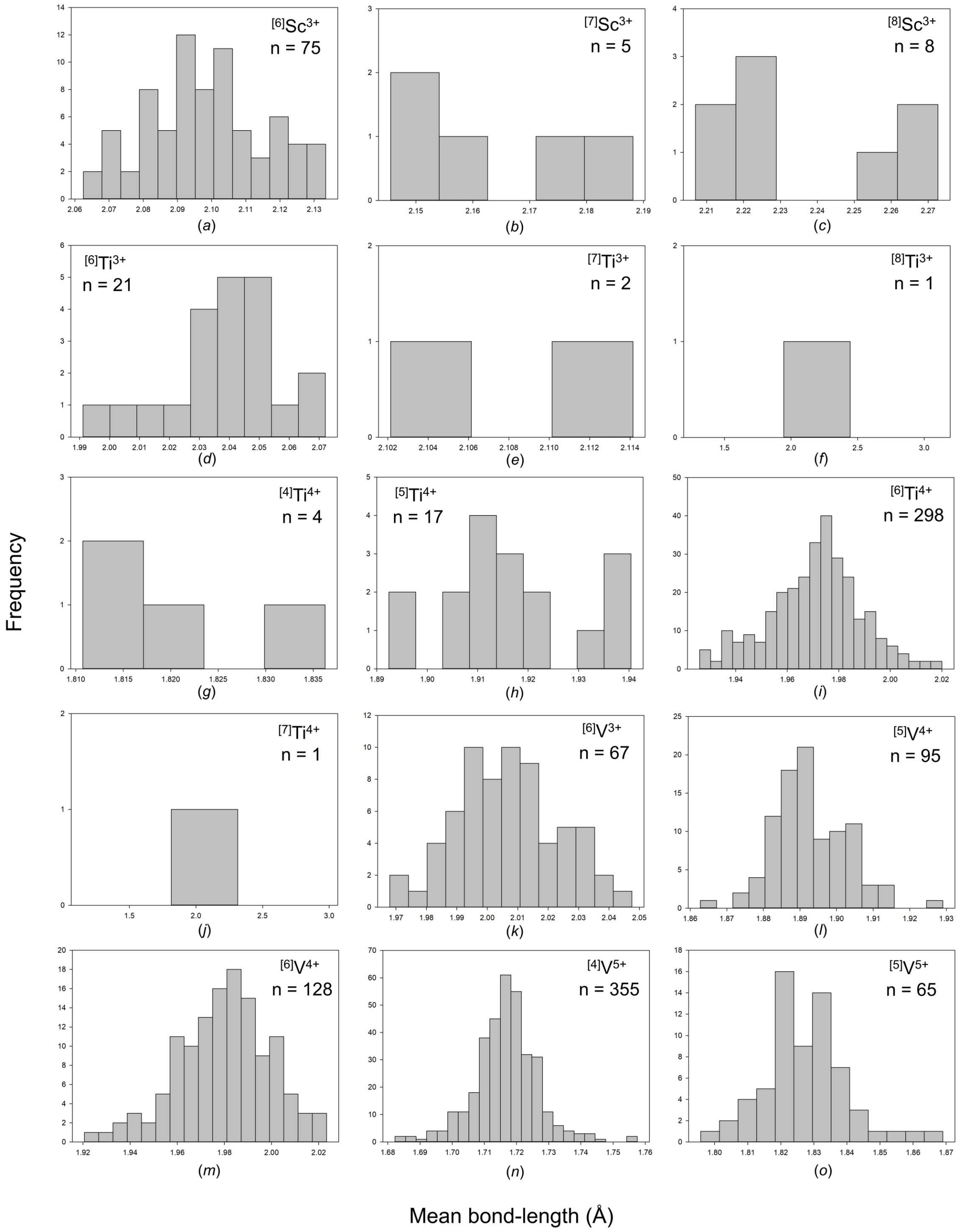
[6]Sc3+
n = 75
(a)
[7]Sc3+
n = 5
(b)
[8]Sc3+
n = 8
(c)
[6]Ti3+
n = 21
(d)
[7]Ti3+
n = 2
(e)
[8]Ti3+
n = 1
(f)
[4]Ti4+
n = 4
(g)
[5]Ti4+
n = 17
(h)
[6]Ti4+
n = 298
(i)
[7]Ti4+
n = 1
(j)
[6]V3+
n = 67
(k)
[5]V4+
n = 95
(l)
[6]V4+
n = 128
(m)
[4]V5+
n = 355
(n)
[5]V5+
n = 65
(o)
Frequency
Mean bond-length (Å)

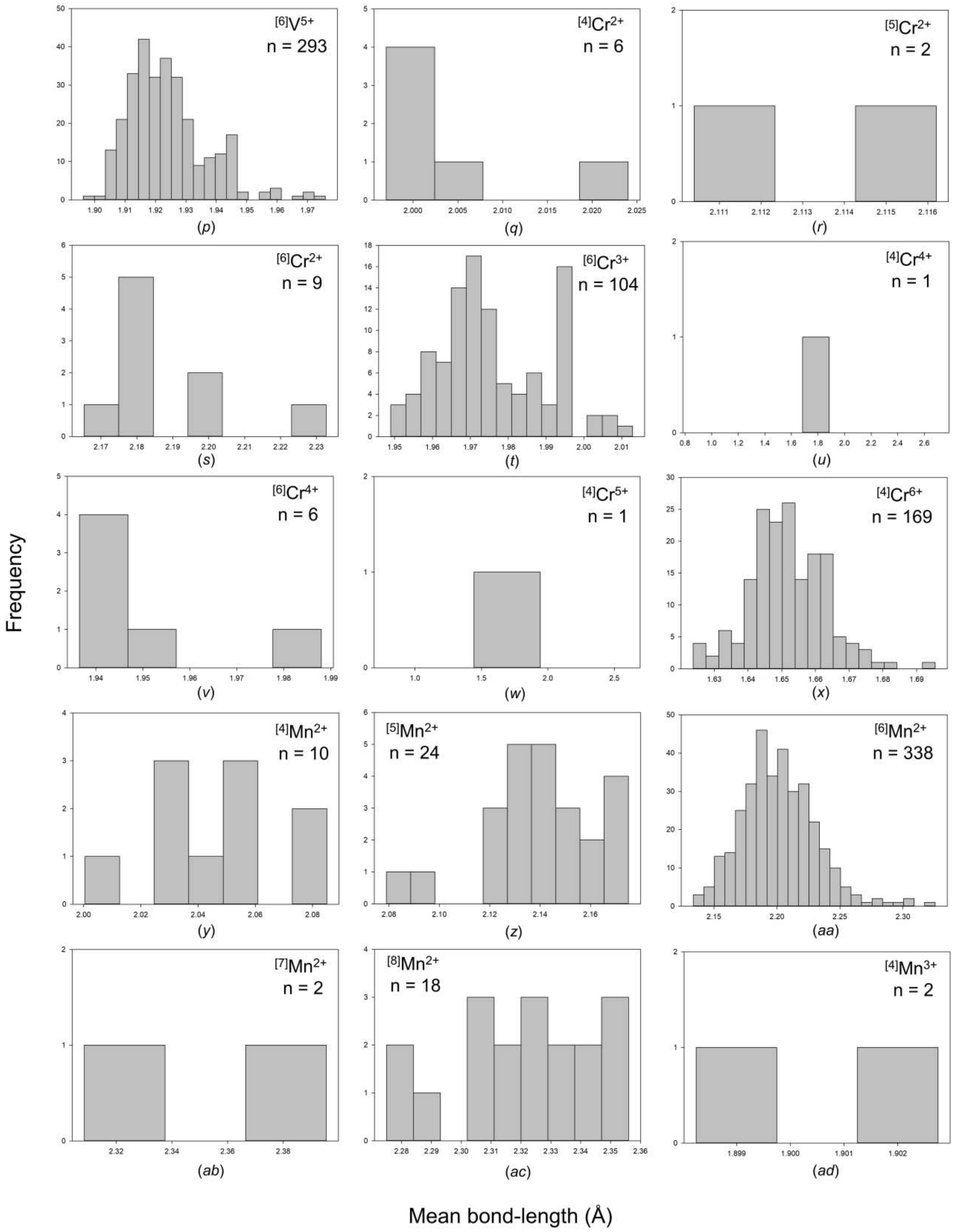
[6]V5+
n = 293
(p)
[4]Cr2+
n = 6
(q)
[5]Cr2+
n = 2
(r)
[6]Cr2+
n = 9
(s)
[6]Cr3+
n = 104
(t)
[4]Cr4+
n = 1
(u)
[6]Cr4+
n = 6
(v)
[4]Cr5+
n = 1
(w)
[4]Cr6+
n = 169
(x)
[4]Mn2+
n = 10
(y)
[5]Mn2+
n = 24
(z)
[6]Mn2+
n = 338
(aa)
[7]Mn2+
n = 2
(ab)
[8]Mn2+
n = 18
(ac)
[4]Mn3+
n = 2
(ad)
Frequency
Mean bond-length (Å)

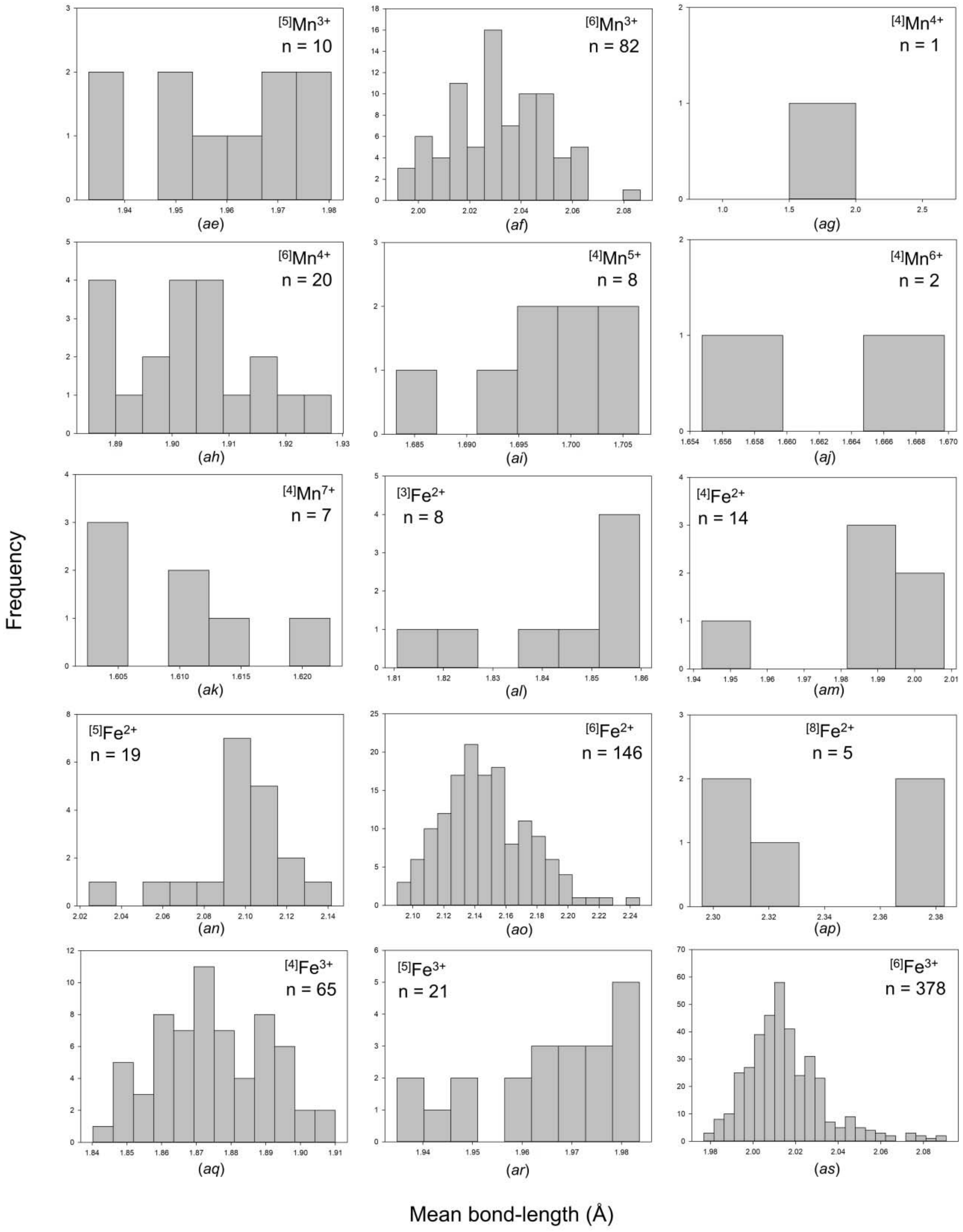
[5]Mn3+
n = 10
(ae)
[6]Mn3+
n = 82
(af)
[4]Mn4+
n = 1
(ag)
[6]Mn4+
n = 20
(ah)
[4]Mn5+
n = 8
(ai)
[4]Mn6+
n = 2
(aj)
[4]Mn7+
n = 7
(ak)
[3]Fe2+
n = 8
(al)
[4]Fe2+
n = 14
(am)
[5]Fe2+
n = 19
(an)
[6]Fe2+
n = 146
(ao)
[8]Fe2+
n = 5
(ap)
[4]Fe3+
n = 65
(aq)
[5]Fe3+
n = 21
(ar)
[6]Fe3+
n = 378
(as)
Frequency
Mean bond-length (Å)

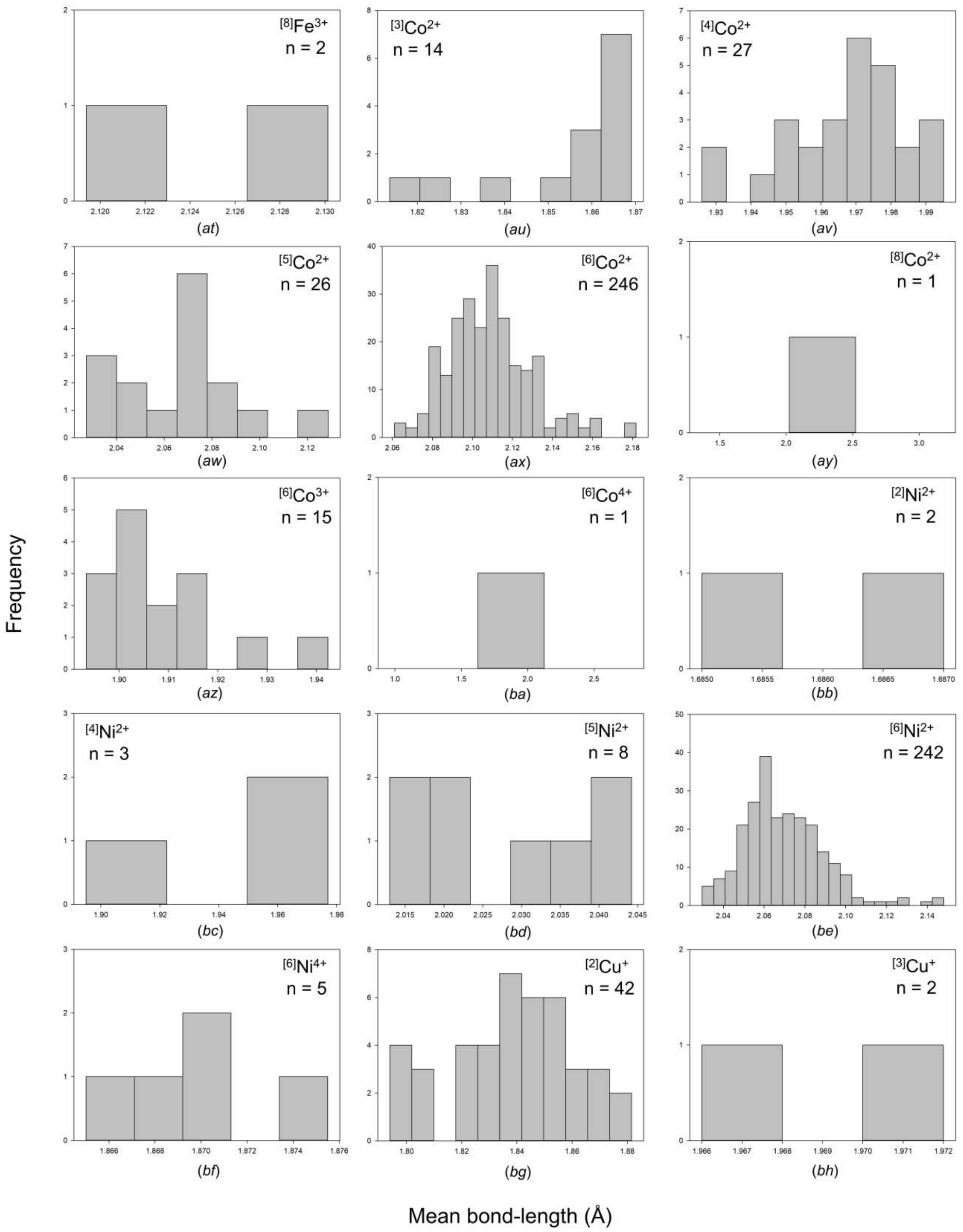

[8]Fe3+ n = 2
(at)
[3]Co2+ n = 14
(au)
[4]Co2+ n = 27
(av)
[5]Co2+ n = 26
(aw)
[6]Co2+ n = 246
(ax)
[8]Co2+ n = 1
(ay)
[6]Co3+ n = 15
(az)
[6]Co4+ n = 1
(ba)
[2]Ni2+ n = 2
(bb)
[4]Ni2+ n = 3
(bc)
[5]Ni2+ n = 8
(bd)
[6]Ni2+ n = 242
(be)
[6]Ni4+ n = 5
(bf)
[2]Cu+ n = 42
(bg)
[3]Cu+ n = 2
(bh)
Frequency
Mean bond-length (Å)

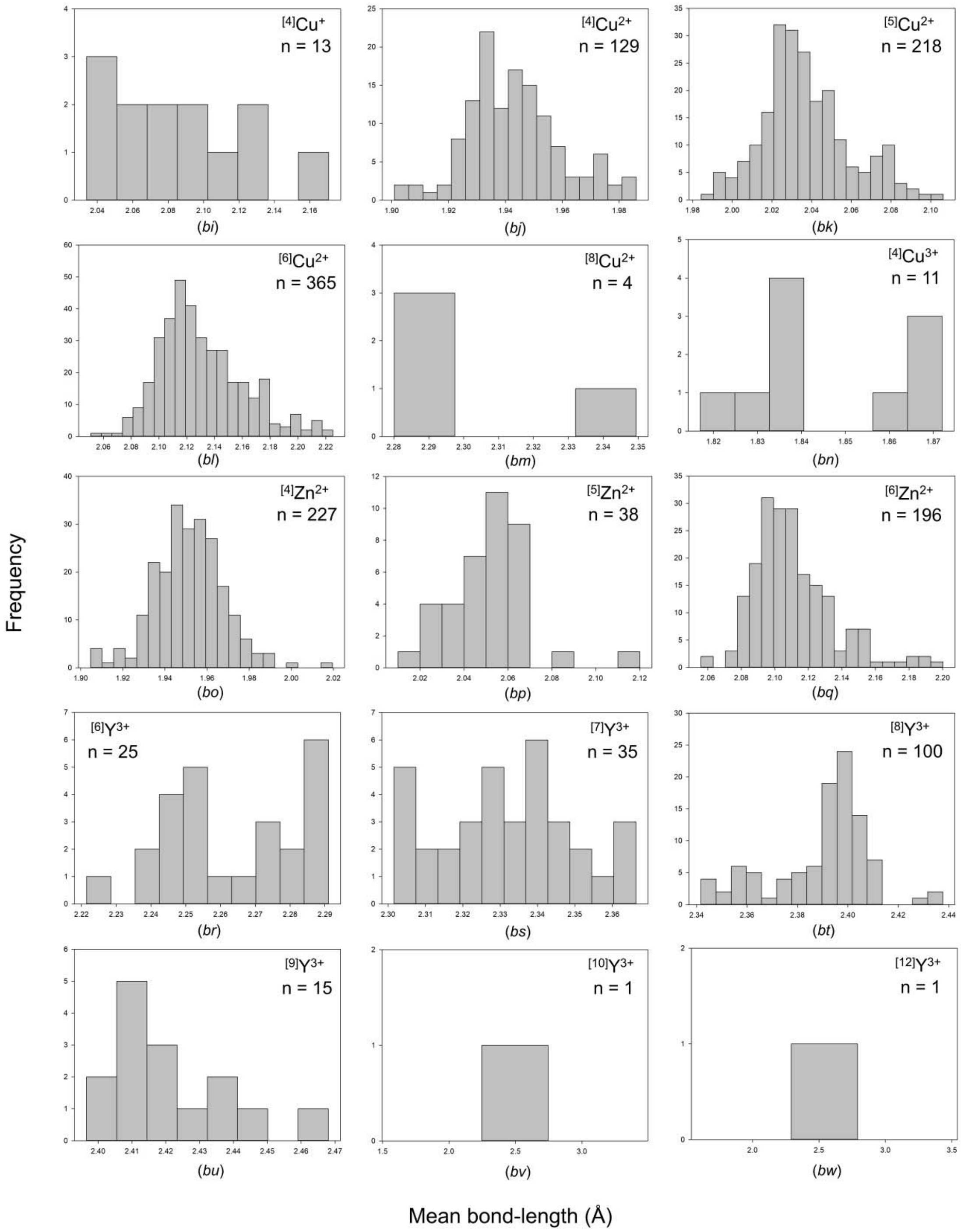
[4]Cu+
n = 13
(bi)
[4]Cu2+
n = 129
(bj)
[5]Cu2+
n = 218
(bk)
[6]Cu2+
n = 365
(bl)
[8]Cu2+
n = 4
(bm)
[4]Cu3+
n = 11
(bn)
[4]Zn2+
n = 227
(bo)
[5]Zn2+
n = 38
(bp)
[6]Zn2+
n = 196
(bq)
[6]Y3+
n = 25
(br)
[7]Y3+
n = 35
(bs)
[8]Y3+
n = 100
(bt)
[9]Y3+
n = 15
(bu)
[10]Y3+
n = 1
(bv)
[12]Y3+
n = 1
(bw)
Frequency
Mean bond-length (Å)

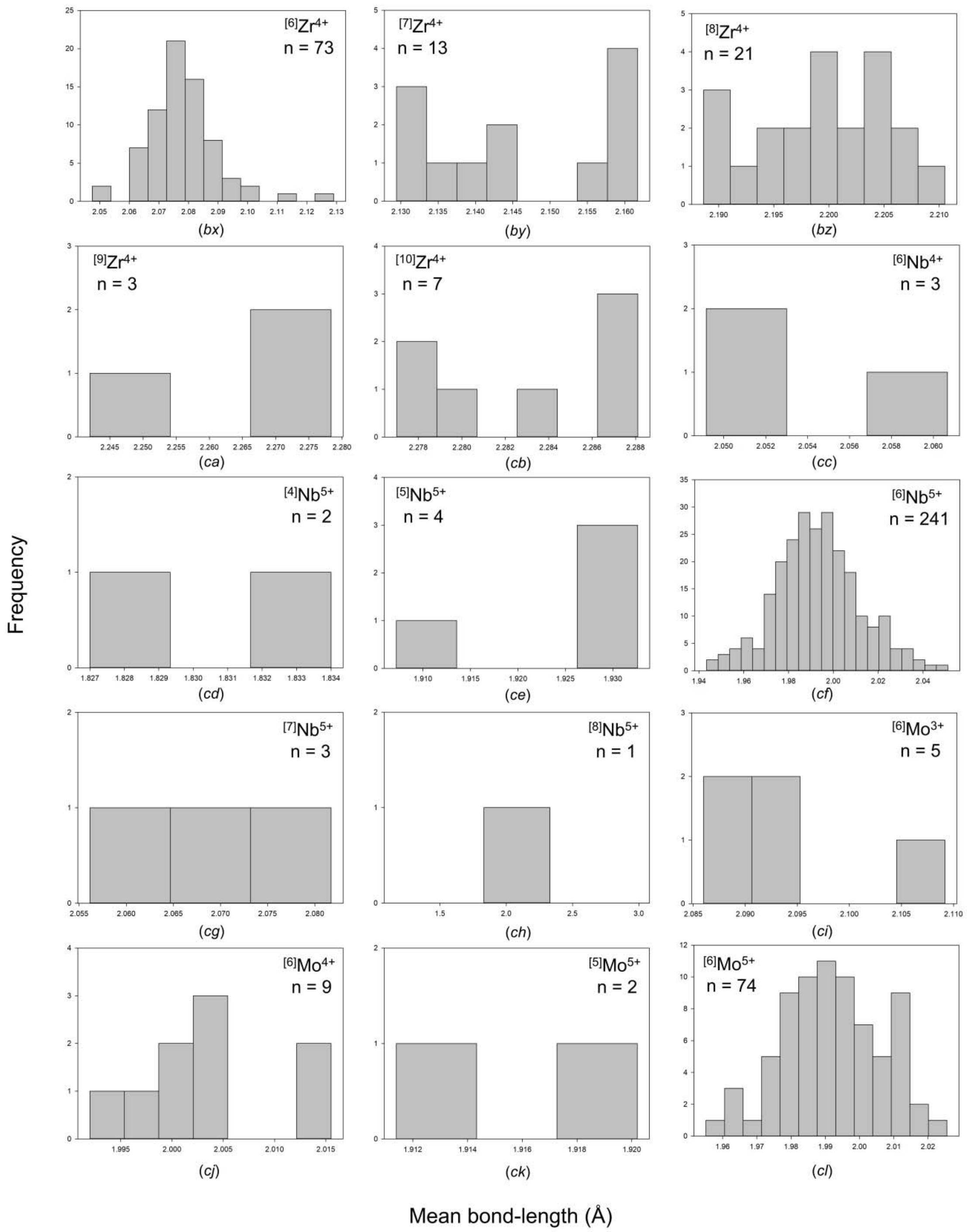
[6]Zr4+
n = 73
(bx)
[7]Zr4+
n = 13
(by)
[8]Zr4+
n = 21
(bz)
[9]Zr4+
n = 3
(ca)
[10]Zr4+
n = 7
(cb)
[6]Nb4+
n = 3
(cc)
[4]Nb5+
n = 2
(cd)
[5]Nb5+
n = 4
(ce)
[6]Nb5+
n = 241
(cf)
[7]Nb5+
n = 3
(cg)
[8]Nb5+
n = 1
(ch)
[6]Mo3+
n = 5
(ci)
[6]Mo4+
n = 9
(cj)
[5]Mo5+
n = 2
(ck)
[6]Mo5+
n = 74
(cl)
Frequency
Mean bond-length (Å)

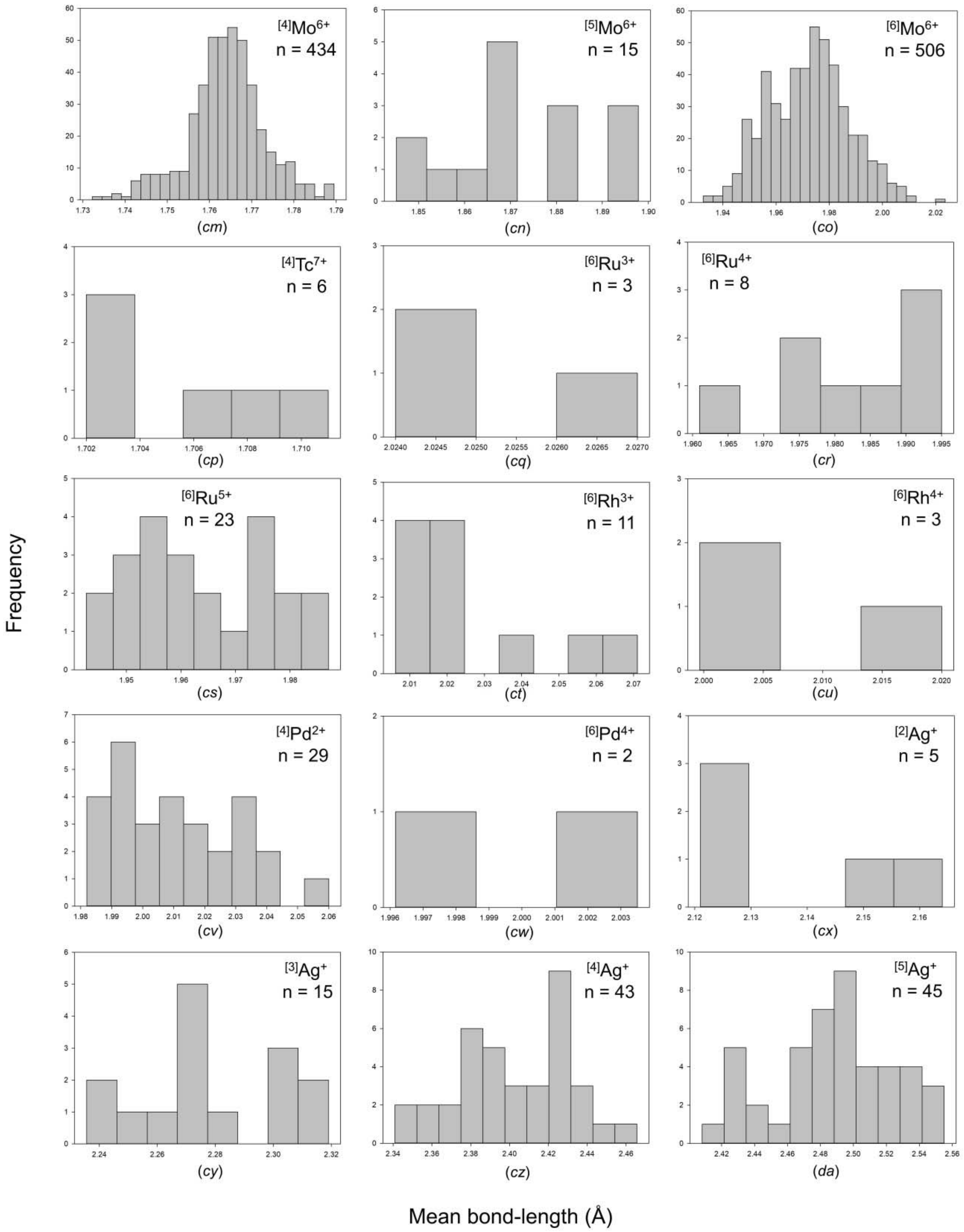
[4]Mo6+
n = 434
(cm)
[5]Mo6+
n = 15
(cn)
[6]Mo6+
n = 506
(co)
[4]Tc7+
n = 6
(cp)
[6]Ru3+
n = 3
(cq)
[6]Ru4+
n = 8
(cr)
[6]Ru5+
n = 23
(cs)
[6]Rh3+
n = 11
(ct)
[6]Rh4+
n = 3
(cu)
[4]Pd2+
n = 29
(cv)
[6]Pd4+
n = 2
(cw)
[2]Ag+
n = 5
(cx)
[3]Ag+
n = 15
(cy)
[4]Ag+
n = 43
(cz)
[5]Ag+
n = 45
(da)
Frequency
Mean bond-length (Å)

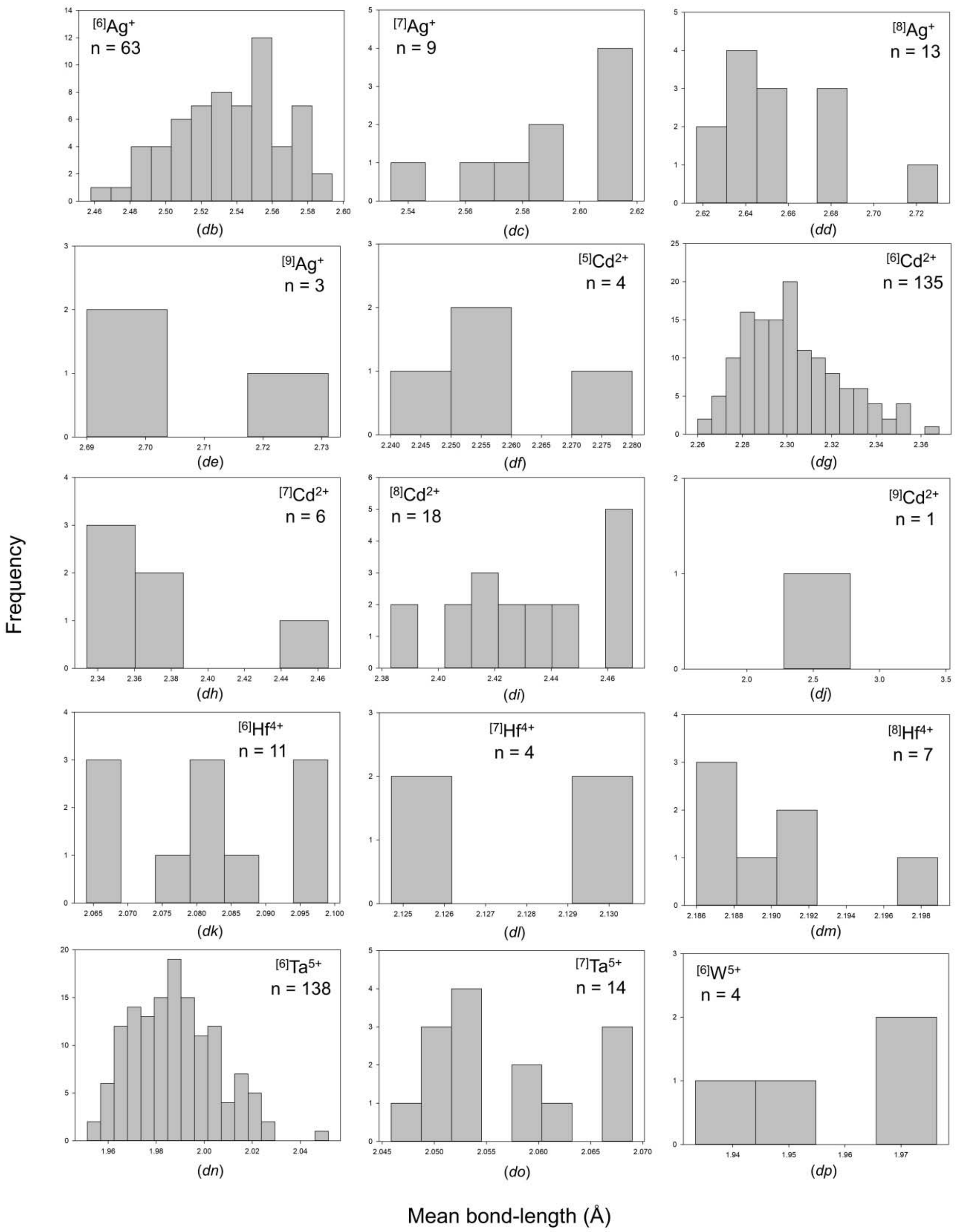

[6]Ag+
n = 63
(db)
[7]Ag+
n = 9
(dc)
[8]Ag+
n = 13
(dd)
[9]Ag+
n = 3
(de)
[5]Cd2+
n = 4
(df)
[6]Cd2+
n = 135
(dg)
[7]Cd2+
n = 6
(dh)
[8]Cd2+
n = 18
(di)
[9]Cd2+
n = 1
(dj)
[6]Hf4+
n = 11
(dk)
[7]Hf4+
n = 4
(dl)
[8]Hf4+
n = 7
(dm)
[6]Ta5+
n = 138
(dn)
[7]Ta5+
n = 14
(do)
[6]W5+
n = 4
(dp)
Frequency
Mean bond-length (Å)

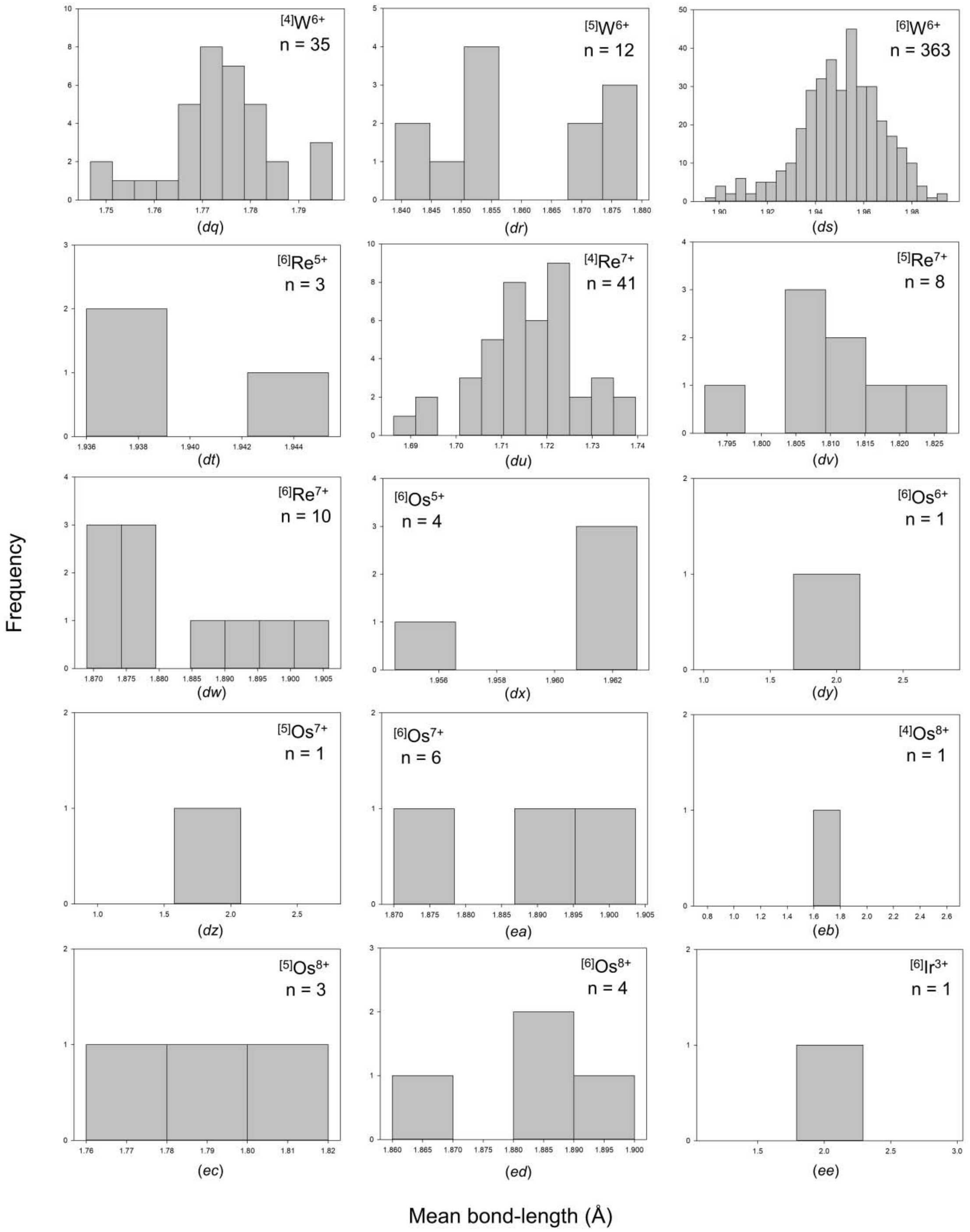
[4]W6+
n = 35
(dq)
[5]W6+
n = 12
(dr)
[6]W6+
n = 363
(ds)
[6]Re5+
n = 3
(dt)
[4]Re7+
n = 41
(du)
[5]Re7+
n = 8
(dv)
[6]Re7+
n = 10
(dw)
[6]Os5+
n = 4
(dx)
[6]Os6+
n = 1
(dy)
[5]Os7+
n = 1
(dz)
[6]Os7+
n = 6
(ea)
[4]Os8+
n = 1
(eb)
[5]Os8+
n = 3
(ec)
[6]Os8+
n = 4
(ed)
[6]Ir3+
n = 1
(ee)
Frequency
Mean bond-length (Å)

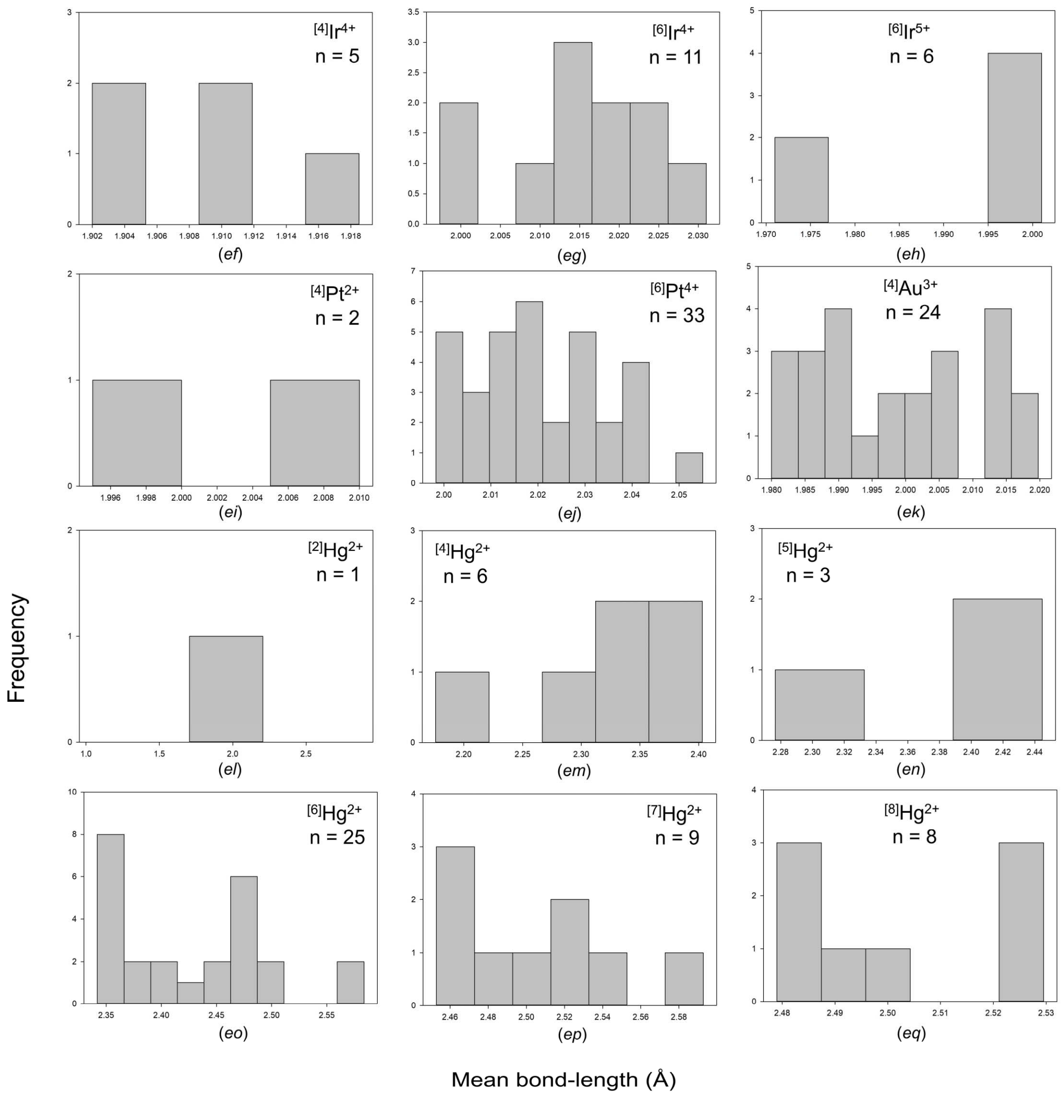

Figure S3 Mean bond-length distributions for all configurations of the transition metal ions bonded to O^{2-}: (*a*) $^{[6]}Sc^{3+}$, (*b*) $^{[7]}Sc^{3+}$, (*c*) $^{[8]}Sc^{3+}$, (*d*) $^{[6]}Ti^{3+}$, (*e*) $^{[7]}Ti^{3+}$, (*f*) $^{[8]}Ti^{3+}$, (*g*) $^{[4]}Ti^{4+}$, (*h*) $^{[5]}Ti^{4+}$, (*i*) $^{[6]}Ti^{4+}$, (*j*) $^{[7]}Ti^{4+}$, (*k*) $^{[6]}V^{3+}$, (*l*) $^{[5]}V^{4+}$, (*m*) $^{[6]}V^{4+}$, (*n*) $^{[4]}V^{5+}$, (*o*) $^{[5]}V^{5+}$, (*p*) $^{[6]}V^{5+}$, (*q*) $^{[4]}Cr^{2+}$, (*r*) $^{[5]}Cr^{2+}$, (*s*) $^{[6]}Cr^{2+}$, (*t*) $^{[6]}Cr^{3+}$, (*u*) $^{[4]}Cr^{4+}$, (*v*) $^{[6]}Cr^{4+}$, (*w*) $^{[4]}Cr^{5+}$, (*x*) $^{[4]}Cr^{6+}$, (*y*) $^{[4]}Mn^{2+}$, (*z*) $^{[5]}Mn^{2+}$, (*aa*) $^{[6]}Mn^{2+}$, (*ab*) $^{[7]}Mn^{2+}$, (*ac*) $^{[8]}Mn^{2+}$, (*ad*) $^{[4]}Mn^{3+}$, (*ae*) $^{[5]}Mn^{3+}$, (*af*) $^{[6]}Mn^{3+}$, (*ag*) $^{[4]}Mn^{4+}$, (*ah*) $^{[6]}Mn^{4+}$, (*ai*) $^{[4]}Mn^{5+}$, (*aj*) $^{[4]}Mn^{6+}$, (*ak*) $^{[4]}Mn^{7+}$, (*al*) $^{[3]}Fe^{2+}$, (*am*) $^{[4]}Fe^{2+}$, (*an*) $^{[5]}Fe^{2+}$, (*ao*) $^{[6]}Fe^{2+}$, (*ap*) $^{[8]}Fe^{2+}$, (*aq*) $^{[4]}Fe^{3+}$, (*ar*) $^{[5]}Fe^{3+}$, (*as*) $^{[6]}Fe^{3+}$, (*at*) $^{[8]}Fe^{3+}$, (*au*) $^{[3]}Co^{2+}$, (*av*) $^{[4]}Co^{2+}$, (*aw*) $^{[5]}Co^{2+}$, (*ax*) $^{[6]}Co^{2+}$, (*ay*) $^{[8]}Co^{2+}$, (*az*) $^{[6]}Co^{3+}$, (*ba*) $^{[6]}Co^{4+}$, (*bb*) $^{[2]}Ni^{2+}$, (*bc*) $^{[4]}Ni^{2+}$, (*bd*) $^{[5]}Ni^{2+}$, (*be*) $^{[6]}Ni^{2+}$, (*bf*) $^{[6]}Ni^{4+}$, (*bg*) $^{[2]}Cu^{+}$, (*bh*) $^{[3]}Cu^{+}$, (*bi*) $^{[4]}Cu^{+}$, (*bj*) $^{[4]}Cu^{2+}$, (*bk*) $^{[5]}Cu^{2+}$, (*bl*) $^{[6]}Cu^{2+}$, (*bm*) $^{[8]}Cu^{2+}$, (*bn*) $^{[4]}Cu^{3+}$, (*bo*) $^{[4]}Zn^{2+}$, (*bp*) $^{[5]}Zn^{2+}$, (*bq*) $^{[6]}Zn^{2+}$, (*br*) $^{[6]}Y^{3+}$, (*bs*) $^{[7]}Y^{3+}$,

(*bt*) $^{[8]}Y^{3+}$, (*bu*) $^{[9]}Y^{3+}$, (*bv*) $^{[10]}Y^{3+}$, (*bw*) $^{[12]}Y^{3+}$, (*bx*) $^{[6]}Zr^{4+}$, (*by*) $^{[7]}Zr^{4+}$, (*bz*) $^{[8]}Zr^{4+}$, (*ca*) $^{[9]}Zr^{4+}$, (*cb*) $^{[10]}Zr^{4+}$, (*cc*) $^{[6]}Nb^{4+}$, (*cd*) $^{[4]}Nb^{5+}$, (*ce*) $^{[5]}Nb^{5+}$, (*cf*) $^{[6]}Nb^{5+}$, (*cg*) $^{[7]}Nb^{5+}$, (*ch*) $^{[8]}Nb^{5+}$, (*ci*) $^{[6]}Mo^{3+}$, (*cj*) $^{[6]}Mo^{4+}$, (*ck*) $^{[5]}Mo^{5+}$, (*cl*) $^{[6]}Mo^{5+}$, (*cm*) $^{[4]}Mo^{6+}$, (*cn*) $^{[5]}Mo^{6+}$, (*co*) $^{[6]}Mo^{6+}$, (*cp*) $^{[4]}Tc^{7+}$, (*cq*) $^{[6]}Ru^{3+}$, (*cr*) $^{[6]}Ru^{4+}$, (*cs*) $^{[6]}Ru^{5+}$, (*ct*) $^{[6]}Rh^{3+}$, (*cu*) $^{[6]}Rh^{4+}$, (*cv*) $^{[4]}Pd^{2+}$, (*cw*) $^{[6]}Pd^{4+}$, (*cx*) $^{[2]}Ag^{+}$, (*cy*) $^{[3]}Ag^{+}$, (*cz*) $^{[4]}Ag^{+}$, (*da*) $^{[5]}Ag^{+}$, (*db*) $^{[6]}Ag^{+}$, (*dc*) $^{[7]}Ag^{+}$, (*dd*) $^{[8]}Ag^{+}$, (*de*) $^{[9]}Ag^{+}$, (*df*) $^{[5]}Cd^{2+}$, (*dg*) $^{[6]}Cd^{2+}$, (*dh*) $^{[7]}Cd^{2+}$, (*di*) $^{[8]}Cd^{2+}$, (*dj*) $^{[9]}Cd^{2+}$, (*dk*) $^{[6]}Hf^{4+}$, (*dl*) $^{[7]}Hf^{4+}$, (*dm*) $^{[8]}Hf^{4+}$, (*dn*) $^{[6]}Ta^{5+}$, (*do*) $^{[7]}Ta^{5+}$, (*dp*) $^{[6]}W^{5+}$, (*dq*) $^{[4]}W^{6+}$, (*dr*) $^{[5]}W^{6+}$, (*ds*) $^{[6]}W^{6+}$, (*dt*) $^{[6]}Re^{5+}$, (*du*) $^{[4]}Re^{7+}$, (*dv*) $^{[5]}Re^{7+}$, (*dw*) $^{[6]}Re^{7+}$, (*dx*) $^{[6]}Os^{5+}$, (*dy*) $^{[6]}Os^{6+}$, (*dz*) $^{[5]}Os^{7+}$, (*ea*) $^{[6]}Os^{7+}$, (*eb*) $^{[4]}Os^{8+}$, (*ec*) $^{[5]}Os^{8+}$, (*ed*) $^{[6]}Os^{8+}$, (*ee*) $^{[6]}Ir^{3+}$, (*ef*) $^{[4]}Ir^{4+}$, (*eg*) $^{[6]}Ir^{4+}$, (*eh*) $^{[6]}Ir^{5+}$, (*ei*) $^{[4]}Pt^{2+}$, (*ej*) $^{[6]}Pt^{4+}$, (*ek*) $^{[4]}Au^{3+}$, (*el*) $^{[2]}Hg^{2+}$, (*em*) $^{[4]}Hg^{2+}$, (*en*) $^{[5]}Hg^{2+}$, (*eo*) $^{[6]}Hg^{2+}$, (*ep*) $^{[7]}Hg^{2+}$, (*eq*) $^{[8]}Hg^{2+}$.

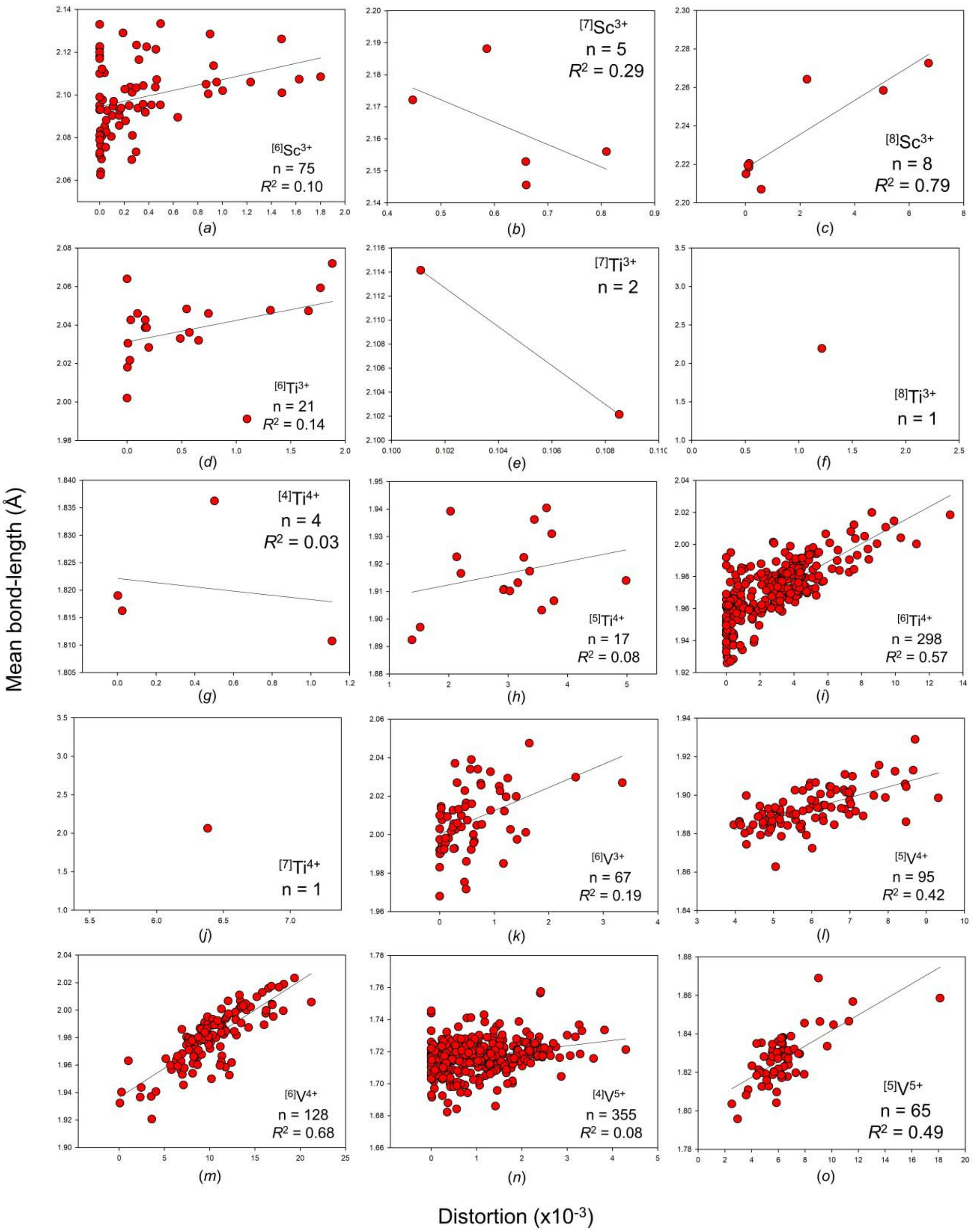
[6]Sc3+
n = 75
R2 = 0.10
(a)
[7]Sc3+
n = 5
R2 = 0.29
(b)
[8]Sc3+
n = 8
R2 = 0.79
(c)
[6]Ti3+
n = 21
R2 = 0.14
(d)
[7]Ti3+
n = 2
(e)
[8]Ti3+
n = 1
(f)
[4]Ti4+
n = 4
R2 = 0.03
(g)
[5]Ti4+
n = 17
R2 = 0.08
(h)
[6]Ti4+
n = 298
R2 = 0.57
(i)
[7]Ti4+
n = 1
(j)
[6]V3+
n = 67
R2 = 0.19
(k)
[5]V4+
n = 95
R2 = 0.42
(l)
[6]V4+
n = 128
R2 = 0.68
(m)
[4]V5+
n = 355
R2 = 0.08
(n)
[5]V5+
n = 65
R2 = 0.49
(o)
Mean bond-length (Å)
Distortion (x10-3)

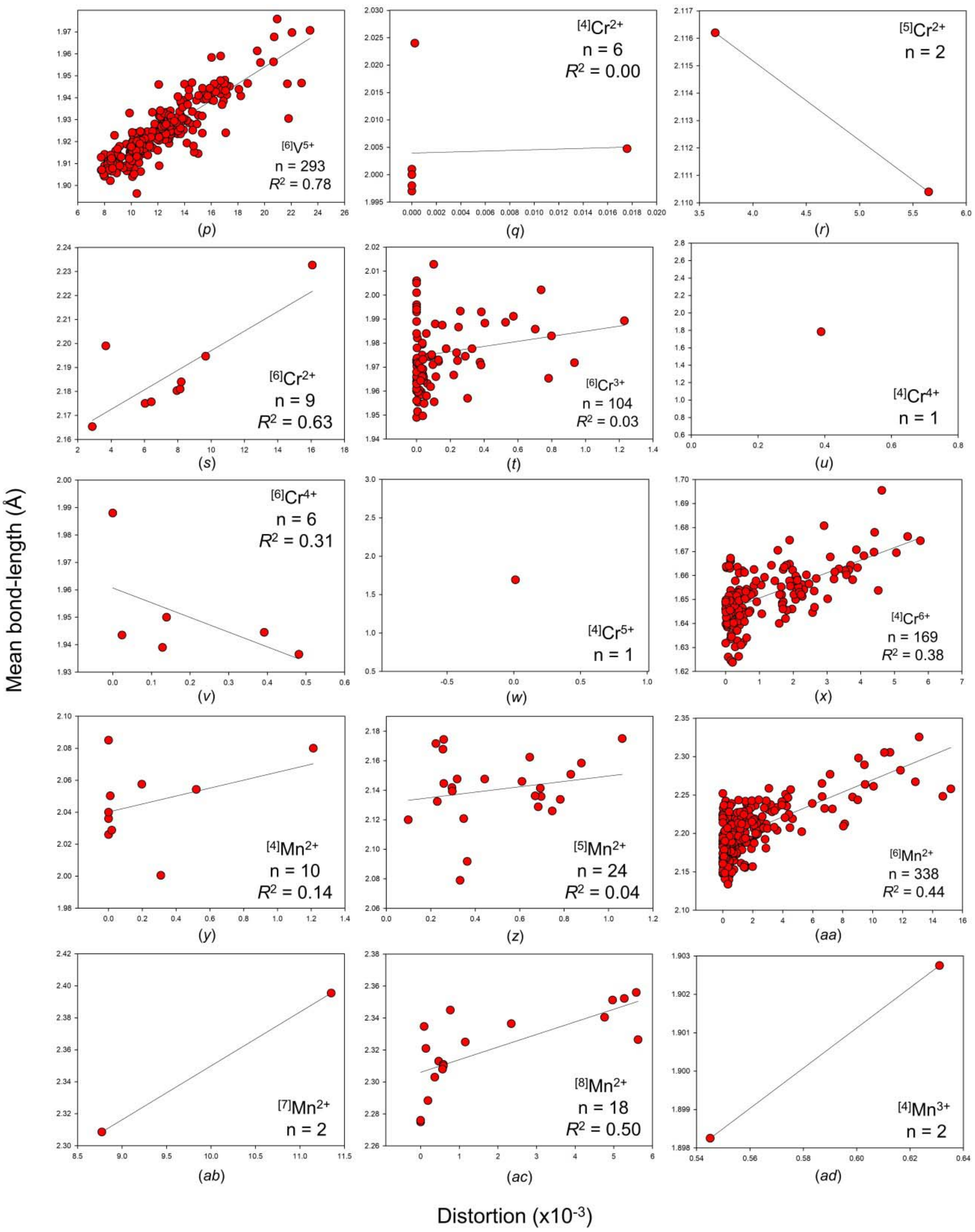
[6]V5+
n = 293
R2 = 0.78
(p)
[4]Cr2+
n = 6
R2 = 0.00
(q)
[5]Cr2+
n = 2
(r)
[6]Cr2+
n = 9
R2 = 0.63
(s)
[6]Cr3+
n = 104
R2 = 0.03
(t)
[4]Cr4+
n = 1
(u)
[6]Cr4+
n = 6
R2 = 0.31
(v)
[4]Cr5+
n = 1
(w)
[4]Cr6+
n = 169
R2 = 0.38
(x)
[4]Mn2+
n = 10
R2 = 0.14
(y)
[5]Mn2+
n = 24
R2 = 0.04
(z)
[6]Mn2+
n = 338
R2 = 0.44
(aa)
[7]Mn2+
n = 2
(ab)
[8]Mn2+
n = 18
R2 = 0.50
(ac)
[4]Mn3+
n = 2
(ad)
Mean bond-length (Å)
Distortion (x10-3)

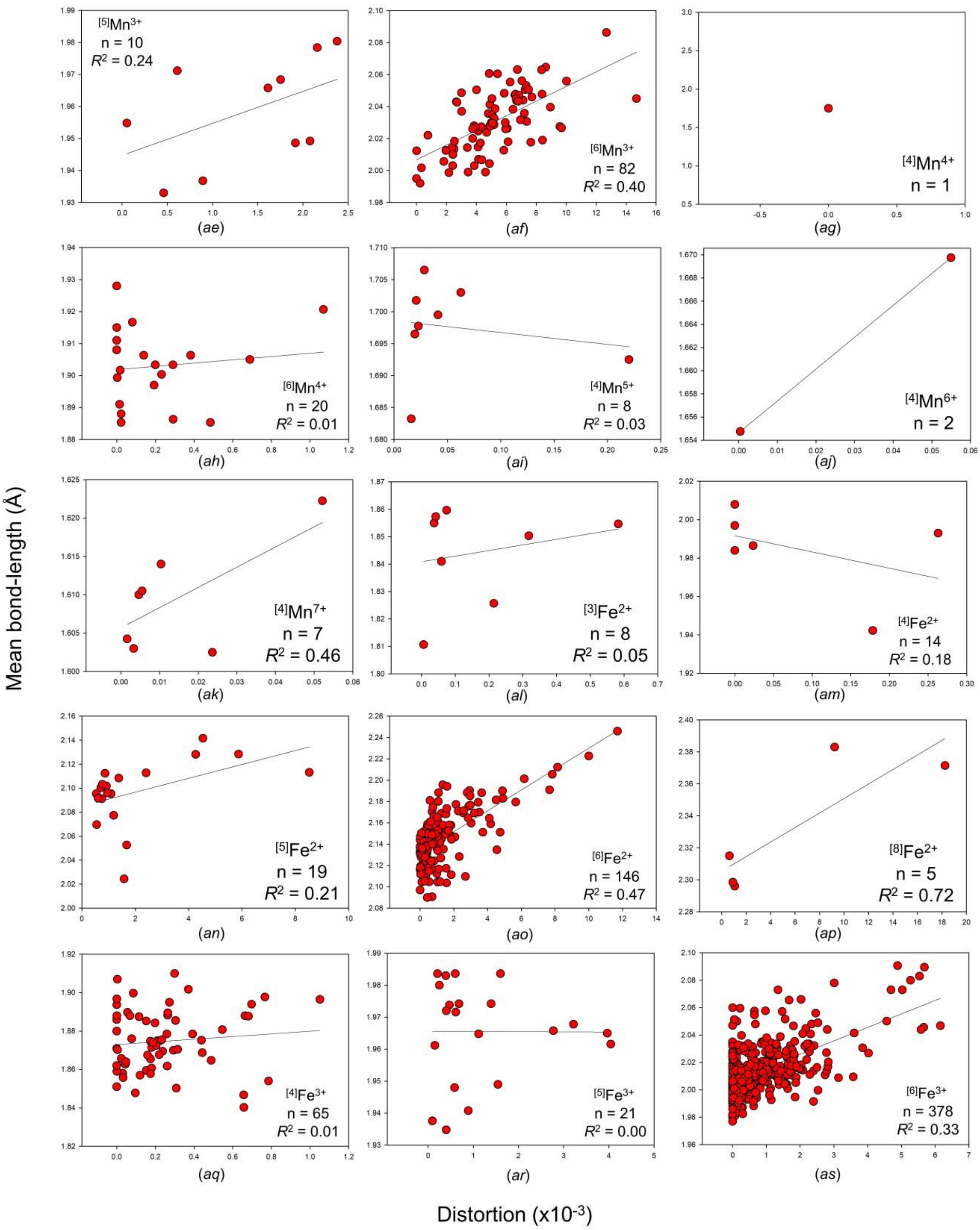

Mean bond-length (Å)
Distortion (x10^-3)
[5]Mn3+ n = 10 R2 = 0.24
[6]Mn3+ n = 82 R2 = 0.40
[4]Mn4+ n = 1
[6]Mn4+ n = 20 R2 = 0.01
[4]Mn5+ n = 8 R2 = 0.03
[4]Mn6+ n = 2
[4]Mn7+ n = 7 R2 = 0.46
[3]Fe2+ n = 8 R2 = 0.05
[4]Fe2+ n = 14 R2 = 0.18
[5]Fe2+ n = 19 R2 = 0.21
[6]Fe2+ n = 146 R2 = 0.47
[8]Fe2+ n = 5 R2 = 0.72
[4]Fe3+ n = 65 R2 = 0.01
[5]Fe3+ n = 21 R2 = 0.00
[6]Fe3+ n = 378 R2 = 0.33
(ae) (af) (ag) (ah) (ai) (aj) (ak) (al) (am) (an) (ao) (ap) (aq) (ar) (as)

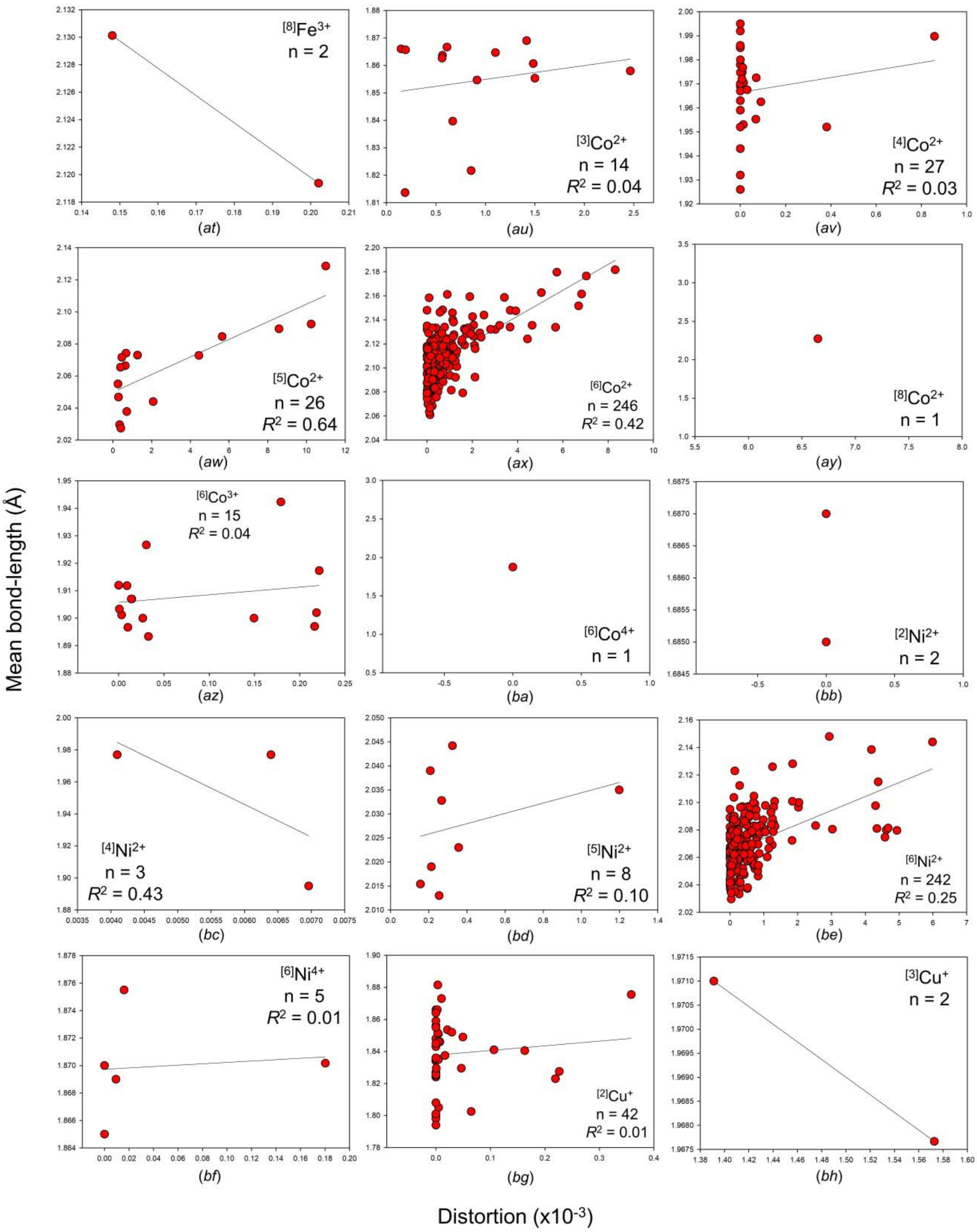
[8]Fe3+
n = 2
(at)
[3]Co2+
n = 14
R2 = 0.04
(au)
[4]Co2+
n = 27
R2 = 0.03
(av)
[5]Co2+
n = 26
R2 = 0.64
(aw)
[6]Co2+
n = 246
R2 = 0.42
(ax)
[8]Co2+
n = 1
(ay)
[6]Co3+
n = 15
R2 = 0.04
(az)
[6]Co4+
n = 1
(ba)
[2]Ni2+
n = 2
(bb)
[4]Ni2+
n = 3
R2 = 0.43
(bc)
[5]Ni2+
n = 8
R2 = 0.10
(bd)
[6]Ni2+
n = 242
R2 = 0.25
(be)
[6]Ni4+
n = 5
R2 = 0.01
(bf)
[2]Cu+
n = 42
R2 = 0.01
(bg)
[3]Cu+
n = 2
(bh)
Mean bond-length (Å)
Distortion (x10-3)

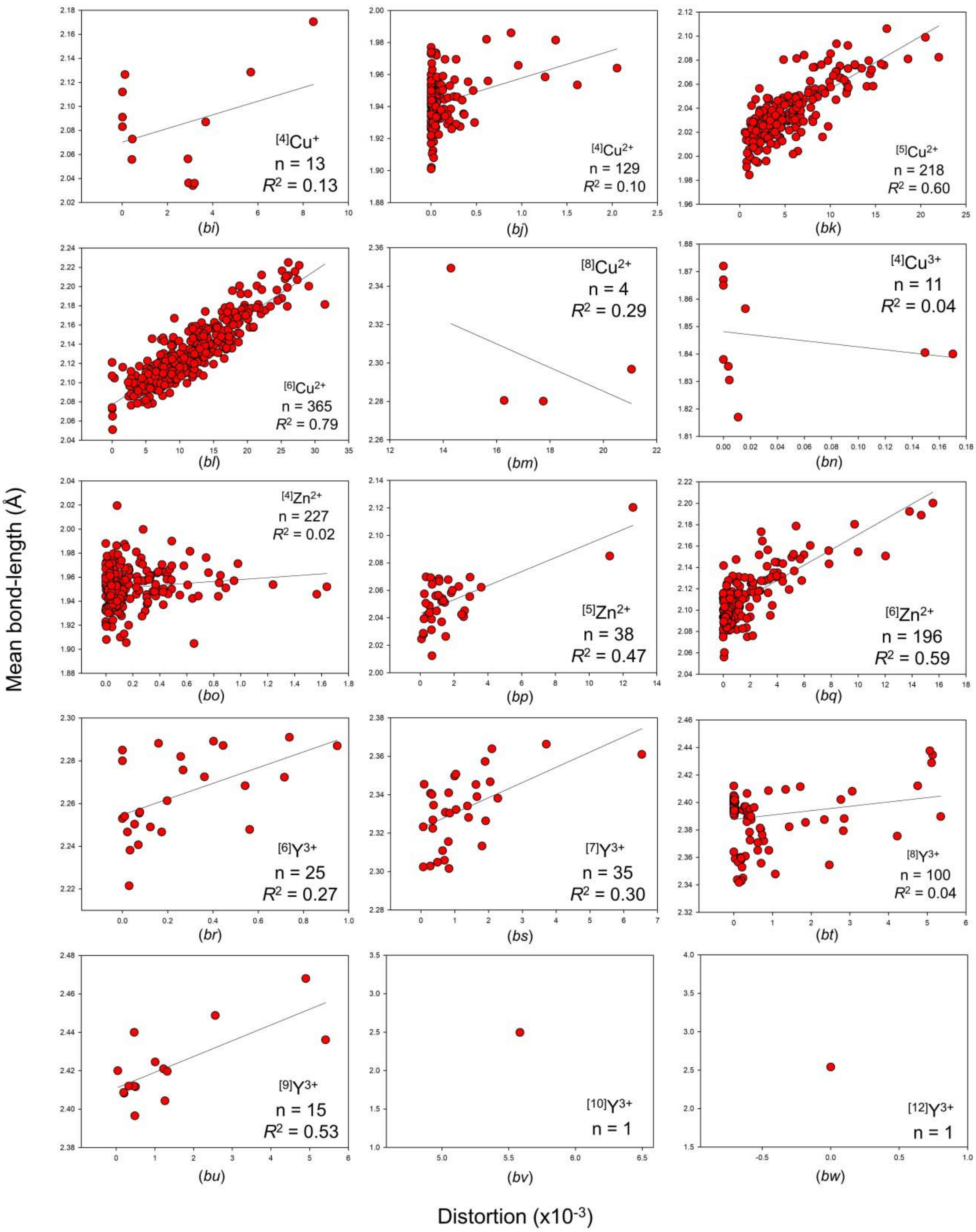
[4]Cu+
n = 13
R2 = 0.13
(bi)
[4]Cu2+
n = 129
R2 = 0.10
(bj)
[5]Cu2+
n = 218
R2 = 0.60
(bk)
[6]Cu2+
n = 365
R2 = 0.79
(bl)
[8]Cu2+
n = 4
R2 = 0.29
(bm)
[4]Cu3+
n = 11
R2 = 0.04
(bn)
[4]Zn2+
n = 227
R2 = 0.02
(bo)
[5]Zn2+
n = 38
R2 = 0.47
(bp)
[6]Zn2+
n = 196
R2 = 0.59
(bq)
[6]Y3+
n = 25
R2 = 0.27
(br)
[7]Y3+
n = 35
R2 = 0.30
(bs)
[8]Y3+
n = 100
R2 = 0.04
(bt)
[9]Y3+
n = 15
R2 = 0.53
(bu)
[10]Y3+
n = 1
(bv)
[12]Y3+
n = 1
(bw)
Mean bond-length (Å)
Distortion (x10-3)

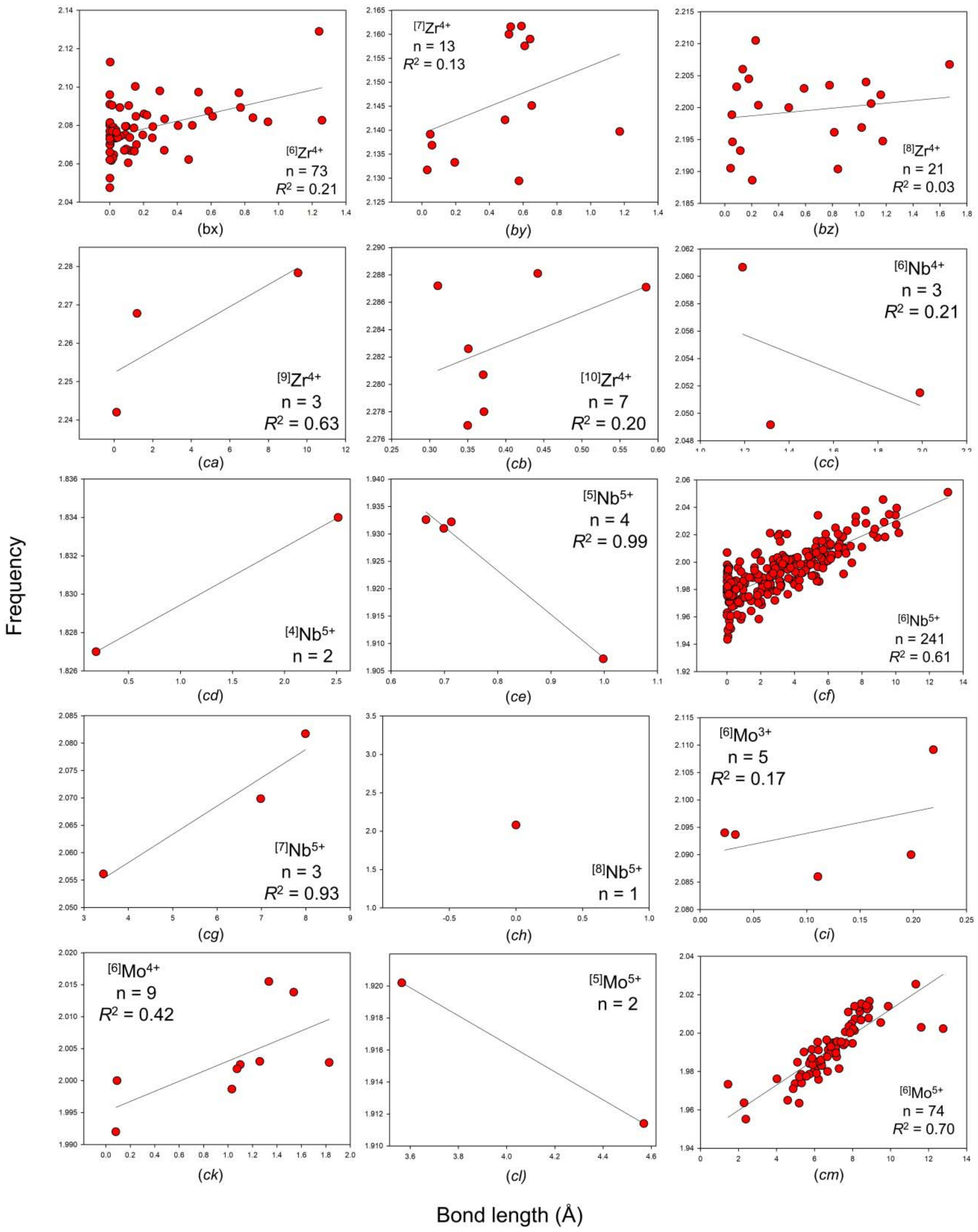
Frequency
Bond length (Å)
[6]Zr4+ n = 73 R2 = 0.21
(bx)
[7]Zr4+ n = 13 R2 = 0.13
(by)
[8]Zr4+ n = 21 R2 = 0.03
(bz)
[9]Zr4+ n = 3 R2 = 0.63
(ca)
[10]Zr4+ n = 7 R2 = 0.20
(cb)
[6]Nb4+ n = 3 R2 = 0.21
(cc)
[4]Nb5+ n = 2
(cd)
[5]Nb5+ n = 4 R2 = 0.99
(ce)
[6]Nb5+ n = 241 R2 = 0.61
(cf)
[7]Nb5+ n = 3 R2 = 0.93
(cg)
[8]Nb5+ n = 1
(ch)
[6]Mo3+ n = 5 R2 = 0.17
(ci)
[6]Mo4+ n = 9 R2 = 0.42
(ck)
[5]Mo5+ n = 2
(cl)
[6]Mo5+ n = 74 R2 = 0.70
(cm)

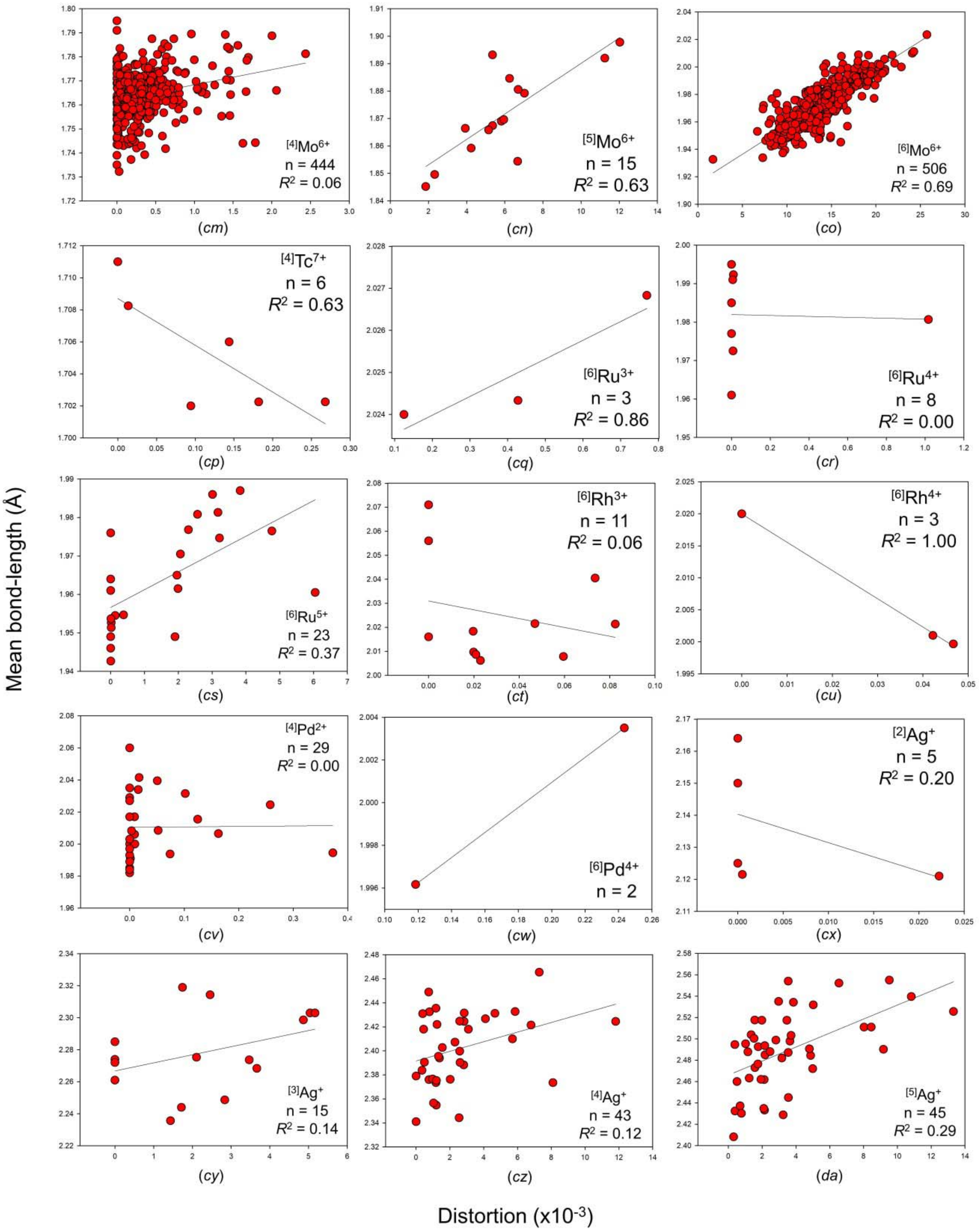
[4]Mo6+
n = 444
R2 = 0.06
(cm)
[5]Mo6+
n = 15
R2 = 0.63
(cn)
[6]Mo6+
n = 506
R2 = 0.69
(co)
[4]Tc7+
n = 6
R2 = 0.63
(cp)
[6]Ru3+
n = 3
R2 = 0.86
(cq)
[6]Ru4+
n = 8
R2 = 0.00
(cr)
[6]Ru5+
n = 23
R2 = 0.37
(cs)
[6]Rh3+
n = 11
R2 = 0.06
(ct)
[6]Rh4+
n = 3
R2 = 1.00
(cu)
[4]Pd2+
n = 29
R2 = 0.00
(cv)
[6]Pd4+
n = 2
(cw)
[2]Ag+
n = 5
R2 = 0.20
(cx)
[3]Ag+
n = 15
R2 = 0.14
(cy)
[4]Ag+
n = 43
R2 = 0.12
(cz)
[5]Ag+
n = 45
R2 = 0.29
(da)
Mean bond-length (Å)
Distortion (x10-3)

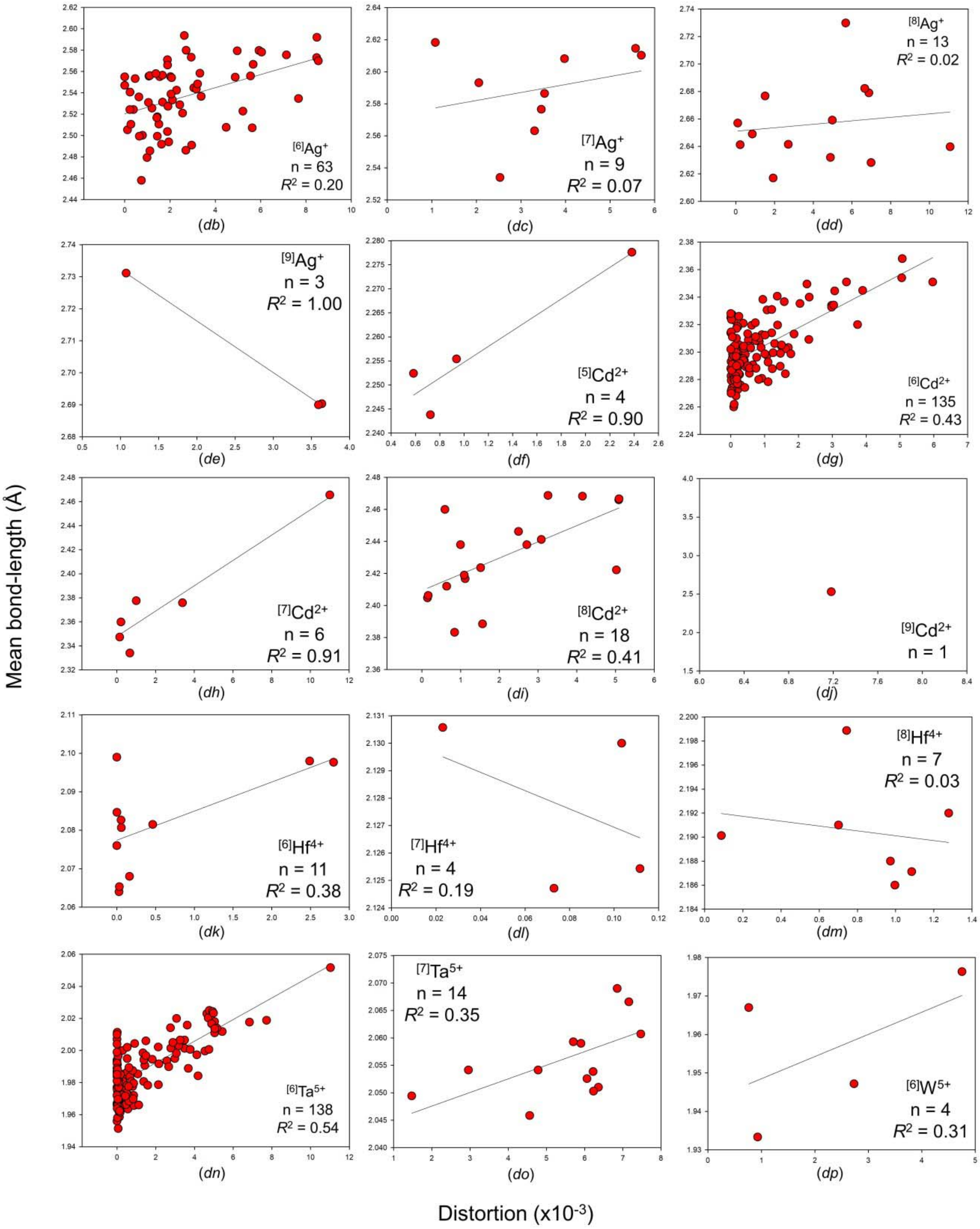
Mean bond-length (Å)
Distortion (x10-3)
[6]Ag+ n = 63 R2 = 0.20
(db)
[7]Ag+ n = 9 R2 = 0.07
(dc)
[8]Ag+ n = 13 R2 = 0.02
(dd)
[9]Ag+ n = 3 R2 = 1.00
(de)
[5]Cd2+ n = 4 R2 = 0.90
(df)
[6]Cd2+ n = 135 R2 = 0.43
(dg)
[7]Cd2+ n = 6 R2 = 0.91
(dh)
[8]Cd2+ n = 18 R2 = 0.41
(di)
[9]Cd2+ n = 1
(dj)
[6]Hf4+ n = 11 R2 = 0.38
(dk)
[7]Hf4+ n = 4 R2 = 0.19
(dl)
[8]Hf4+ n = 7 R2 = 0.03
(dm)
[6]Ta5+ n = 138 R2 = 0.54
(dn)
[7]Ta5+ n = 14 R2 = 0.35
(do)
[6]W5+ n = 4 R2 = 0.31
(dp)

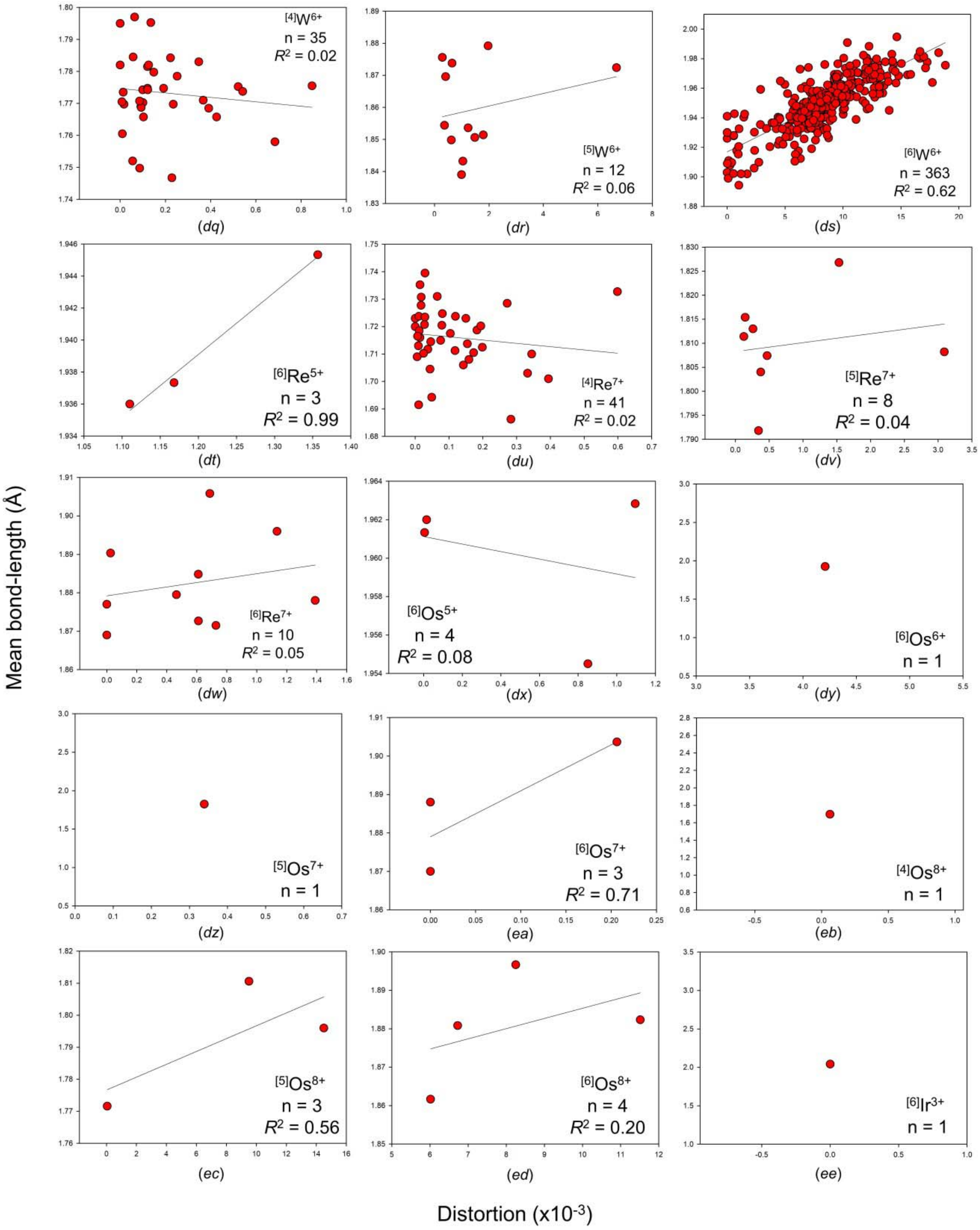
[4]W6+
n = 35
R2 = 0.02
(dq)
[5]W6+
n = 12
R2 = 0.06
(dr)
[6]W6+
n = 363
R2 = 0.62
(ds)
[6]Re5+
n = 3
R2 = 0.99
(dt)
[4]Re7+
n = 41
R2 = 0.02
(du)
[5]Re7+
n = 8
R2 = 0.04
(dv)
[6]Re7+
n = 10
R2 = 0.05
(dw)
[6]Os5+
n = 4
R2 = 0.08
(dx)
[6]Os6+
n = 1
(dy)
[5]Os7+
n = 1
(dz)
[6]Os7+
n = 3
R2 = 0.71
(ea)
[4]Os8+
n = 1
(eb)
[5]Os8+
n = 3
R2 = 0.56
(ec)
[6]Os8+
n = 4
R2 = 0.20
(ed)
[6]Ir3+
n = 1
(ee)
Mean bond-length (Å)
Distortion (x10-3)

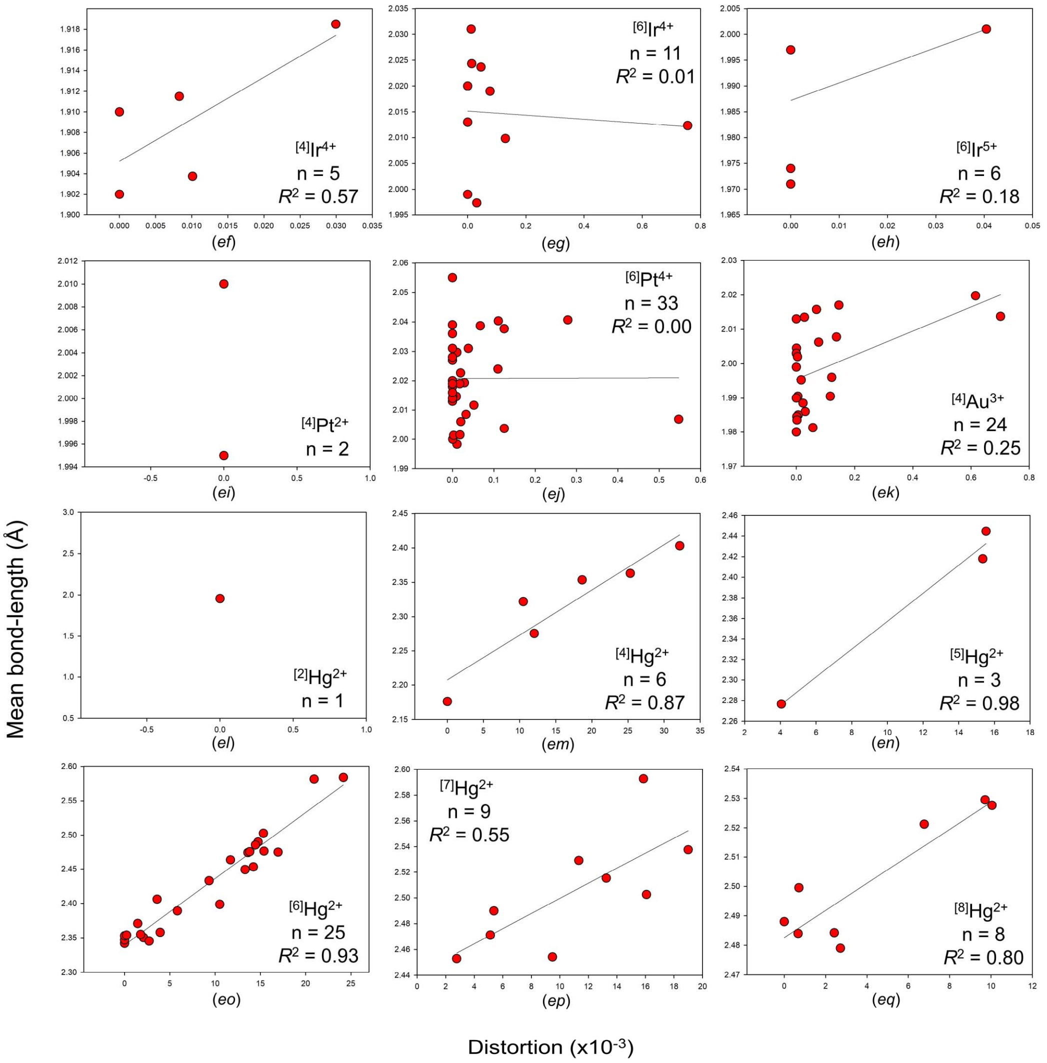

Figure S4 The effect of bond-length distortion on mean bond-length for all configurations of the transition metal ions bonded to O^{2-}: (*a*) $^{[6]}Sc^{3+}$, (*b*) $^{[7]}Sc^{3+}$, (*c*) $^{[8]}Sc^{3+}$, (*d*) $^{[6]}Ti^{3+}$, (*e*) $^{[7]}Ti^{3+}$, (*f*) $^{[8]}Ti^{3+}$, (*g*) $^{[4]}Ti^{4+}$, (*h*) $^{[5]}Ti^{4+}$, (*i*) $^{[6]}Ti^{4+}$, (*j*) $^{[7]}Ti^{4+}$, (*k*) $^{[6]}V^{3+}$, (*l*) $^{[5]}V^{4+}$, (*m*) $^{[6]}V^{4+}$, (*n*) $^{[4]}V^{5+}$, (*o*) $^{[5]}V^{5+}$, (*p*) $^{[6]}V^{5+}$, (*q*) $^{[4]}Cr^{2+}$, (*r*) $^{[5]}Cr^{2+}$, (*s*) $^{[6]}Cr^{2+}$, (*t*) $^{[6]}Cr^{3+}$, (*u*) $^{[4]}Cr^{4+}$, (*v*) $^{[6]}Cr^{4+}$, (*w*) $^{[4]}Cr^{5+}$, (*x*) $^{[4]}Cr^{6+}$, (*y*) $^{[4]}Mn^{2+}$, (*z*) $^{[5]}Mn^{2+}$, (*aa*) $^{[6]}Mn^{2+}$, (*ab*) $^{[7]}Mn^{2+}$, (*ac*) $^{[8]}Mn^{2+}$, (*ad*) $^{[4]}Mn^{3+}$, (*ae*) $^{[5]}Mn^{3+}$, (*af*) $^{[6]}Mn^{3+}$, (*ag*) $^{[4]}Mn^{4+}$, (*ah*) $^{[6]}Mn^{4+}$, (*ai*) $^{[4]}Mn^{5+}$, (*aj*) $^{[4]}Mn^{6+}$, (*ak*) $^{[4]}Mn^{7+}$, (*al*) $^{[3]}Fe^{2+}$, (*am*) $^{[4]}Fe^{2+}$, (*an*) $^{[5]}Fe^{2+}$, (*ao*) $^{[6]}Fe^{2+}$, (*ap*) $^{[8]}Fe^{2+}$, (*aq*) $^{[4]}Fe^{3+}$, (*ar*) $^{[5]}Fe^{3+}$, (*as*) $^{[6]}Fe^{3+}$, (*at*) $^{[8]}Fe^{3+}$, (*au*) $^{[3]}Co^{2+}$, (*av*) $^{[4]}Co^{2+}$, (*aw*) $^{[5]}Co^{2+}$, (*ax*) $^{[6]}Co^{2+}$, (*ay*) $^{[8]}Co^{2+}$, (*az*) $^{[6]}Co^{3+}$, (*ba*) $^{[6]}Co^{4+}$, (*bb*) $^{[2]}Ni^{2+}$, (*bc*) $^{[4]}Ni^{2+}$, (*bd*) $^{[5]}Ni^{2+}$, (*be*) $^{[6]}Ni^{2+}$, (*bf*) $^{[6]}Ni^{4+}$, (*bg*) $^{[2]}Cu^{+}$, (*bh*) $^{[3]}Cu^{+}$, (*bi*) $^{[4]}Cu^{+}$, (*bj*) $^{[4]}Cu^{2+}$, (*bk*) $^{[5]}Cu^{2+}$, (*bl*) $^{[6]}Cu^{2+}$, (*bm*) $^{[8]}Cu^{2+}$, (*bn*) $^{[4]}Cu^{3+}$, (*bo*) $^{[4]}Zn^{2+}$, (*bp*) $^{[5]}Zn^{2+}$, (*bq*) $^{[6]}Zn^{2+}$, (*br*) $^{[6]}Y^{3+}$, (*bs*) $^{[7]}Y^{3+}$, (*bt*) $^{[8]}Y^{3+}$, (*bu*) $^{[9]}Y^{3+}$, (*bv*) $^{[10]}Y^{3+}$, (*bw*) $^{[12]}Y^{3+}$, (*bx*) $^{[6]}Zr^{4+}$, (*by*) $^{[7]}Zr^{4+}$,

(*bz*) $^{[8]}Zr^{4+}$, (*ca*) $^{[9]}Zr^{4+}$, (*cb*) $^{[10]}Zr^{4+}$, (*cc*) $^{[6]}Nb^{4+}$, (*cd*) $^{[4]}Nb^{5+}$, (*ce*) $^{[5]}Nb^{5+}$, (*cf*) $^{[6]}Nb^{5+}$, (*cg*) $^{[7]}Nb^{5+}$, (*ch*) $^{[8]}Nb^{5+}$, (*ci*) $^{[6]}Mo^{3+}$, (*cj*) $^{[6]}Mo^{4+}$, (*ck*) $^{[5]}Mo^{5+}$, (*cl*) $^{[6]}Mo^{5+}$, (*cm*) $^{[4]}Mo^{6+}$, (*cn*) $^{[5]}Mo^{6+}$, (*co*) $^{[6]}Mo^{6+}$, (*cp*) $^{[4]}Tc^{7+}$, (*cq*) $^{[6]}Ru^{3+}$, (*cr*) $^{[6]}Ru^{4+}$, (*cs*) $^{[6]}Ru^{5+}$, (*ct*) $^{[6]}Rh^{3+}$, (*cu*) $^{[6]}Rh^{4+}$, (*cv*) $^{[4]}Pd^{2+}$, (*cw*) $^{[6]}Pd^{4+}$, (*cx*) $^{[2]}Ag^{+}$, (*cy*) $^{[3]}Ag^{+}$, (*cz*) $^{[4]}Ag^{+}$, (*da*) $^{[5]}Ag^{+}$, (*db*) $^{[6]}Ag^{+}$, (*dc*) $^{[7]}Ag^{+}$, (*dd*) $^{[8]}Ag^{+}$, (*de*) $^{[9]}Ag^{+}$, (*df*) $^{[5]}Cd^{2+}$, (*dg*) $^{[6]}Cd^{2+}$, (*dh*) $^{[7]}Cd^{2+}$, (*di*) $^{[8]}Cd^{2+}$, (*dj*) $^{[9]}Cd^{2+}$, (*dk*) $^{[6]}Hf^{4+}$, (*dl*) $^{[7]}Hf^{4+}$, (*dm*) $^{[8]}Hf^{4+}$, (*dn*) $^{[6]}Ta^{5+}$, (*do*) $^{[7]}Ta^{5+}$, (*dp*) $^{[6]}W^{5+}$, (*dq*) $^{[4]}W^{6+}$, (*dr*) $^{[5]}W^{6+}$, (*ds*) $^{[6]}W^{6+}$, (*dt*) $^{[6]}Re^{5+}$, (*du*) $^{[4]}Re^{7+}$, (*dv*) $^{[5]}Re^{7+}$, (*dw*) $^{[6]}Re^{7+}$, (*dx*) $^{[6]}Os^{5+}$, (*dy*) $^{[6]}Os^{6+}$, (*dz*) $^{[5]}Os^{7+}$, (*ea*) $^{[6]}Os^{7+}$, (*eb*) $^{[4]}Os^{8+}$, (*ec*) $^{[5]}Os^{8+}$, (*ed*) $^{[6]}Os^{8+}$, (*ee*) $^{[6]}Ir^{3+}$, (*ef*) $^{[4]}Ir^{4+}$, (*eg*) $^{[6]}Ir^{4+}$, (*eh*) $^{[6]}Ir^{5+}$, (*ei*) $^{[4]}Pt^{2+}$, (*ej*) $^{[6]}Pt^{4+}$, (*ek*) $^{[4]}Au^{3+}$, (*el*) $^{[2]}Hg^{2+}$, (*em*) $^{[4]}Hg^{2+}$, (*en*) $^{[5]}Hg^{2+}$, (*eo*) $^{[6]}Hg^{2+}$, (*ep*) $^{[7]}Hg^{2+}$, (*eq*) $^{[8]}Hg^{2+}$.

Table S1 *A priori* bond valences for crystal-structure refinements used in this work

1292: $Cu^{2+}{}_5O_2(PO_4)_2$					
	*Cu*1	*Cu*2	*Cu*3	*P*	∑
*O*1		0.367 ×2↓ ×2→		1.265	2
*O*2		0.551		1.449	2
*O*3	0.388 ×2↓		0.277 ×2↓ ×2→	1.059	2
*O*4		0.329	0.444	1.227	2
*O*5	0.612 ×2↓	0.385	0.501 ×2↓ ×2→		2
∑	2	2	2	5	

1640: $(Hg^{2+}(H_2O)_6)(Cl^{7+}O_4)_2$				
	Hg	*Cl*	*H*	∑
*O*1	0.333 ×6↓		0.833 ×2→	2
*O*2		1.667 ×3↓	0.167 ×2→	2
*O*3		2.000		2
∑	2	7	1	

2279: $Ba_3Cu^{2+}(Sb^{5+}{}_2O_9)$							
∑	*Ba*1	*Ba*2	*Ba*3	*Cu*	*Sb*1	*Sb*2	∑
*O*1	0.206 ×3↓	0.175 ×6↓ ×2→	0.152 ×3↓	0.396 ×3↓	0.896 ×3↓		2
*O*2	0.080 ×6↓ ×2→	0.149 ×3↓	0.026 ×3↓	0.270 ×3↓	0.770 ×3↓	0.724 ×3↓	2
*O*3	0.300 ×3↓	0.268 ×3↓	0.245 ×6↓ ×2→			0.943 ×3↓	2
∑	2	2	2	2	5	5	

8269: $Cr^{3+}(W^{5+}O_4)$			
	Cr	*W*	∑
*O*1	0.542 ×4↓ ×2→	0.917 ×2↓	2
*O*2	0.417	0.792 ×2↓ ×2→	2
*O*3	0.417	0.792 ×2↓ ×2→	2
∑	3	5	

14159: $Na2(Ni^{2+}O_2)$				
	*Na*1	*Na*2	*Ni*	∑
*O*1	0.219 ×2↓ ×2→	0.266 ×2↓ ×2→	0.516 ×2↓ ×2→	2
*O*2	0.188 ×3↓ ×3→	0.234 ×2↓ ×2→	0.484 ×2↓ ×2→	2
∑	1	1	2	

15505: $Y_3Re^{7+}O_8$					
	*Y*1	*Y*2	*Y*3	*Re*	∑
*O*1	0.495	0.486 ×2↓ ×2→	0.533		2
*O*2	0.495	0.486 ×2↓ ×2→	0.533		2
*O*3		0.254	0.302 ×2↓ ×2→	1.143	2
*O*4	0.282 ×2↓ ×2→	0.274		1.162	2
*O*5	0.361		0.399	1.240	2
*O*6	0.273	0.264	0.311	1.152	2
*O*7	0.273	0.264	0.311	1.152	2
*O*8	0.270 ×2↓ ×2→		0.309	1.150	2
∑	3	3	3	7	

15545: $Na_4Zr_2(SiO_4)_3$					
	*Na*1	*Na*2	*Zr*	*Si*	∑
*O*1	0.167 ×6↓	0.104 ×4↓ ×2→	0.646 ×3↓	0.979 ×2↓	2
*O*2		0.146 ×4↓ ×2→	0.688 ×3↓	1.021 ×2↓	2
∑	1	1	4	4	

17062: $Fe^{2+}{}_2(P_2O_7)$					
	*Fe*1	*Fe*2	*P*1	*P*2	∑
*O*1	0.333	0.333	1.333		2
*O*2	0.333	0.333	1.333		2
*O*3	0.333	0.333	1.333		2
*O*4			1.000	1.000	2
*O*5	0.333	0.333		1.333	2
*O*6	0.333	0.333		1.333	2
*O*7	0.333	0.333		1.333	2
∑	2	2	5	5	

20540: $Li_2[Os^{8+}O_4(OH)_2]$				
	*Li*1	*Li*2	*Os*	∑
*O*1	0.217 ×2↓	0.400 ×2↓	1.383 ×2↓	2
*O*2	0.417 ×2↓		1.583 ×2↓	2
*O*3	-0.133 ×2↓	0.050 ×4↓ ×2→	1.033 ×2↓	2
∑	1	1	8	

20611: Rb($Os^{8+}{}_2O_8$(OH))				
	Rb	*Os*1	*Os*2	∑
*O*1	0.000 ×2↓ ×2→		2.000	2
*O*2	0.000 ×2↓ ×2→		2.000	2
*O*3	0.176 ×2↓ ×2→	1.647		2
*O*4	0.265	1.735		2
*O*5	0.000		2.000	2
*O*6	0.176 ×2↓ ×2→	1.647		2
*O*7	0.000 ×2↓ ×2→		2.000	2
*O*8	0.265	1.735		2
OH	-0.235	1.235		1
∑	1	8	8	

20670: $YCo^{2+}(BO_2)_5$								
	Co	*Y*	*B*1	*B*2	*B*3	*B*4	*B*5	∑
*O*1	0.300	0.246	0.715	0.738				2
*O*2	0.323		0.738			0.939		2
*O*3		0.273	0.742				0.985	2
*O*4	0.390		0.805		0.806			2
*O*5	0.300	0.246		0.738	0.716			2
*O*6		0.259 ×2↓ ×2→		0.752	0.729			2
*O*7		0.280		0.772		0.949		2
*O*8		0.259			0.749		0.992	2
*O*9	0.344 ×2↓ ×2→	0.290					1.023	2
*O*10		0.444 ×2↓ ×2→				1.113		2
∑	2	3	3	3	3	3	3	

24819: $Na_5Nb^{5+}O_5$					
	*Na*1	*Na*2	*Na*3	*Nb*	Σ
*O*1	0.181 ×2↓	0.181 ×2↓ ×2→	0.238 ×2↓ ×2→	0.981 ×2↓	2
*O*2	0.229 ×2↓	0.229 ×2↓ ×2→	0.286	1.029 ×2↓	2
*O*3	0.181	0.181 ×2→	0.238 ×2→	0.981	2
Σ	1	1	1	5	

24973: $Ca_4Mn^{3+}{}_3B_3O_{12}CO_3$						
	*Ca*1	*Ca*2	*Mn*	*B*	*C*	Σ
*O*1	0.177 ×3↓	0.318	0.480	1.025		2
*O*2	0.177 ×3↓	0.318	0.480	1.025		2
*O*3		0.242	0.404 ×2↓ ×2→	0.949		2
*O*4	0.313 ×3↓	0.455	0.616 ×2↓ ×2→			2
*O*5		0.222 ×3↓ ×3→			1.333 ×3↓	2
Σ	2	2	3	3	4	

33194: $Pb^{2+}HfO_3$					
	*Pb*1	*Pb*2	*Hf*1	*Hf*2	Σ
*O*1	0.174 ×4↓ ×4→		0.645	0.661	2
*O*2		0.166 ×4↓ ×4→	0.661	0.676	2
*O*3	0.221	0.197 ×2↓ ×2→	0.692 ×2↓ ×2→		2
*O*4	0.179 ×2↓ ×2→	0.155 ×2↓ ×2→		0.666 ×2↓ ×2→	2
*O*5	0.184 ×2→	0.161 ×2→	0.655 ×2→		2
*O*6	0.179 ×2→	0.155 ×2→		0.666 ×2→	2
*O*7	0.184 ×2→	0.161 ×2→	0.655 ×2→		2
*O*8	0.179 ×2→	0.155 ×2→		0.666 ×2→	2
Σ	2	2	4	4	

33783: $La(Nb^{5+}{}_5O_{14})$					
	La	*Nb*1	*Nb*2	*Nb*3	Σ
*O*1	0.211 ×2↓		0.894 ×2↓ ×2→		2
*O*2	0.210 ×4↓ ×2→	0.688	0.893		2
*O*3	0.348 ×2↓	0.826 ×2↓ ×2→			2
*O*4		0.591	0.796	0.614 ×2↓	2
*O*5		0.591	0.796	0.614 ×2↓	2
*O*6	0.261 ×4↓ ×2→	0.739 ×2↓ ×2→			2
*O*7				1.000 ×2↓ ×2→	2
*O*8			0.727 ×2→	0.545	2
Σ	3	5	5	5	

33800: $La_3Ti^{4+}O_4Cl_5$					
	*La*1	*La*2	*La*3	*Ti*	Σ
*O*1			0.542 ×2↓ ×2→	0.915	2
*O*2		0.540 ×2↓ ×2→		0.920	2
*O*3	0.253	0.245	0.252	0.625 ×2↓ ×2→	2
*O*4	0.543 ×2↓ ×2→			0.915	2
*Cl*1	0.253	0.244	0.252 ×2↓ ×2→		1
*Cl*2	0.254	0.246 ×2↓ ×2→	0.253		1
*Cl*3		0.248 ×3↓ ×3→	0.255		1
*Cl*4	0.250 ×3↓ ×3→		0.249		1
*Cl*5	0.202 ×2↓ ×2→	0.194	0.201 ×2↓ ×2→		1
Σ	3	3	3	4	

33802: $Sr_2(Ru^{4+}O_4)$			
	Sr	*Ru*	Σ
*O*1	0.179 ×4↓ ×4→	0.641 ×4↓ ×2→	2
*O*2	0.256 ×5↓ ×5→	0.718 ×2↓	2
Σ	2	4	

33863: $Ba_4Ir^{4+}{}_3O_{10}$					
	*Ba*1	*Ba*2	*Ir*1	*Ir*2	Σ
*O*1	0.182 ×3↓ ×3→	0.153	0.663 ×2↓	0.638	2
*O*2	0.187 ×2↓ ×2→	0.158 ×2↓ ×2→	0.668 ×2↓	0.643	2
*O*3	0.187 ×2↓ ×2→	0.158 ×2↓ ×2→	0.668 ×2↓	0.643	2
*O*4	0.326	0.297 ×3↓ ×3→		0.782	2
*O*5	0.191 ×2↓ ×2→	0.162 ×2↓ ×2→		0.647 ×2↓ ×2→	2
Σ	2	2	4	4	

34392: $K_6(Mn^{3+}{}_2O_6)$					
	*K*1	*K*2	*K*3	*Mn*	Σ
*O*1	0.135	0.135	0.155 ×2↓ ×2→	0.710 ×2↓ ×2→	2
*O*2	0.234 ×2↓ ×2→	0.234 ×2↓ ×2→	0.254	0.809	2
*O*3	0.198 ×2↓ ×2→	0.198 ×2↓ ×2→	0.218 ×2↓ ×2→	0.772	2
Σ	1	1	1	3	

35084: $Cd_3Te^{6+}O_6$				
	*Cd*1	*Cd*2	*Te*	∑
*O*1	0.373 ×2↓	0.294 ×2↓ ×2→	1.039 ×2↓	2
*O*2	0.314 ×2↓	0.235 ×3↓ ×3→	0.980 ×2↓	2
*O*3	0.314 ×2↓	0.235 ×3↓ ×3→	0.980 ×2↓	2
∑	2	2	6	

35407: $CdPt^{4+}{}_3O_6$				
	Cd	*Pt*1	*Pt*2	∑
*O*1	0.286 ×4↓ ×2→		0.714 ×2↓ ×2→	2
*O*2	0.214 ×4↓	0.500 ×4↓	0.643 ×4↓ ×2→	2
∑	2	2	4	

36608: $Sm^{3+}(Ti^{4+}O_3Cl)$			
	Sm	*Ti*	∑
*O*1	0.320	0.560 ×3↓ ×3→	2
*O*2	0.587 ×2↓ ×2→	0.827	2
*O*3	0.507	0.747 ×2↓ ×2→	2
Cl	0.250 ×4↓ ×4→		1
∑	3	4	

36626: $Nb^{5+}(PO_4)O$			
	Nb	*P*	Σ
*O*1	0.750	1.250	2
*O*2	1.000 ×2↓ ×2→		2
*O*3	0.750	1.250	2
*O*4	0.750	1.250	2
*O*5	0.750	1.250	2
Σ	5	5	

40249: $KNa_3(W^{6+}O_5)$					
	*Na*1	*Na*2	*K*	*W*	Σ
*O*1	0.192	0.197 ×2→	0.111 ×2↓ ×2→	1.192	2
*O*2	0.197 ×2↓	0.202 ×3↓ ×3→		1.197 ×2↓	2
*O*3	0.192	0.197 ×2→	0.111 ×2↓ ×2→	1.192	2
*O*4	0.221		0.139 ×4↓ ×4→	1.221	2
Σ	1	1	1	6	

40312: $SrZn(V^{5+}{}_2O_7)$					
	Sr	*Zn*	*V*1	*V*2	Σ
*O*1	0.260	0.414	1.326		2
*O*2	0.260	0.414	1.326		2
*O*3	0.260	0.414	1.326		2
*O*4	-0.046		1.021	1.025	2
*O*5	0.310 ×2↓ ×2→			1.381	2
*O*6	0.259	0.412		1.329	2
*O*7	0.194 ×2↓ ×2→	0.347		1.265	2
Σ	2	2	5	5	

40850: $Mn^{2+}(V^{5+}{}_2O_6)$			
	Mn	*V*	Σ
*O*1	0.417 ×4↓ ×2→	1.167	2
*O*2	0.167 ×2↓	0.917 ×2↓ ×2→	2
*O*3		0.667 ×3↓ ×3→	2
Σ	2	5	

49746: $Na_5(Os^{7+}O_6)$					
	*Na*1	*Na*2	*Na*3	*Os*	Σ
*O*1	0.167 ×4↓ ×2→	0.167 ×4↓	0.167 ×4↓ ×2→	1.167 ×4↓	2
*O*2	0.167 ×2↓ ×2→	0.167 ×2↓	0.167 ×2↓ ×2→	1.167 ×2↓	2
Σ	1	1	1	7	

50010: $Cs(V^{5+}{}_3O_8)$				
	Cs	*V*1	*V*2	Σ
*O*1	0.500	1.500		2
*O*2	-0.375	0.625	0.875 ×2→	2
*O*3		0.500 ×2↓	0.750 ×2↓ ×2→	2
*O*4	0.188 ×6↓×3		1.438	2
*O*5	-0.062 ×4↓ ×2→	0.938 ×2↓	1.188	2
Σ	1	5	5	

50038: $K_2Fe^{2+}{}_2Nb^{5+}{}_4O_{13}$					
	K	*Fe*	*Nb*1	*Nb*2	Σ
*O*1	-0.083 ×2↓	0.259 ×2↓ ×2→	0.693	0.872	2
*O*2	0.176 ×2↓ ×2→	0.518		1.130	2
*O*3	0.090 ×2↓		0.866	1.044	2
*O*4	0.221 ×2↓ ×2→	0.562	0.996		2
*O*5		0.039		0.651 ×3↓ ×3→	2
*O*6	0.075 ×2↓ ×4→		0.851 ×2→		2
*O*7	0.021 ×2↓ ×2→	0.363	0.797 ×2↓ ×2→		2
Σ	1	2	5	5	

50459: $Cu^{2+}{}_4O(PO_4)_2$						
	*Cu*1	*Cu*2	*Cu*3	*P*1	*P*2	Σ
*O*1	0.551			1.449		2
*O*2		0.293	0.295 ×2→	1.118		2
*O*3		0.391 ×2↓	0.393	1.216 ×2↓		2
*O*4	0.335	0.408			1.257	2
*O*5			0.384 ×2→		1.231	2
*O*6	0.335 ×2↓		0.409		1.256 ×2↓	2
*O*7	0.445	0.517	0.519 ×2→			2
Σ	2	2	2	5	5	

55272: $Ca_2ZrB(Al_9O_{18})$						
	Ca	*Al*1	*Al*2	*Zr*	*B*	Σ
*O*1		0.444 ×2↓ ×2→	0.444 ×2↓	0.667 ×3↓		2
*O*2	0.500 ×4↓	0.667 ×2↓ ×2→	0.667 ×2↓			2
*O*3		0.444 ×2→	0.444	0.667 ×3↓		2
*O*4		0.333 ×2→	0.333		1.000 ×3↓	2
Σ	2	3	3	4	3	

59111: $HfSiO_4$			
	Hf	*Si*	Σ
O	0.500 ×8↓ ×2→	1.000 ×4↓	2
Σ	4	4	

59244: $LiV^{3+}(Si_2O_6)$				
	Li	*V*	*Si*	Σ
*O*1	0.125 ×2↓	0.450 ×4↓ ×2→	0.975	2
*O*2	0.275 ×2↓	0.600 ×2↓	1.125	2
*O*3	0.100 ×2↓		0.950 ×2↓ ×2→	2
Σ	1	3	4	

59819: $Ce^{4+}{}_2(Cr^{6+}O_4)_4(H_2O)_2$							
	$Ce1$	$Ce2$	$Cr1$	$Cr2$	$Cr3$	$Cr4$	Σ
$O1$			2.000				2
$O2$		0.632	1.368				2
$O3$		0.632	1.368				2
$O4$	0.737		1.263				2
$O5$		0.474		1.526			2
$O6$		0.474		1.526			2
$O7$		0.474		1.526			2
$O8$	0.579			1.421			2
$O9$	0.553				1.447		2
$O10$		0.447			1.553		2
$O11$	0.553				1.447		2
$O12$		0.447			1.553		2
$O13$		0.421				1.579	2
$O14$	0.526					1.474	2
$O15$	0.526					1.474	2
$O16$	0.526					1.474	2
Σ	4	4	6	6	6	6	

62577: $CaNb^{5+}_2(P_4O_{13})(P_2O_7)O$								
	*Nb*1	*Nb*2	*Ca*	*P*1	*P*2	*P*3	*P*4	Σ
*O*1			0.267 ×2↓	1.733				2
*O*2		0.782 ×2↓		1.218				2
*O*3				1 ×2→				2
*O*4				1.049	0.951			2
*O*5	0.805 ×2↓				1.195			2
*O*6			0.316 ×2↓		1.684			2
*O*7		0.831 ×2↓			1.169			2
*O*8	0.837 ×2↓					1.163 ×2↓		2
*O*9			0.348			1.652		2
*O*10						1.169	0.978	2
*O*11	0.859 ×2↓						1.141 ×2↓	2
*O*12		0.261					1.739	2
*O*13		1.515	0.458					2
Σ	5	5	2	5	5	5	5	

63103: $Y_2Ba_2Cu^{2+}Pt^{4+}O_8$							
	*Ba*1	*Ba*2	*Cu*	*Y*1	*Y*2	*Pt*	Σ
*O*1	0.232	0.206 ×3↓ ×3→	0.440			0.711	2
*O*2	0.177 ×2↓	0.151 ×2↓	0.385 ×2↓	0.430 ×4↓ ×2→	0.427 ×2↓		2
*O*3	0.174 ×4↓ ×2→	0.148 ×2↓		0.427 ×2↓	0.424 ×2↓	0.653 ×2↓	2
*O*4	0.174 ×2↓ ×2→	0.148		0.427	0.424	0.653	2
*O*5	0.186 ×2↓	0.160 ×4↓ ×2→	0.394 ×2↓		0.436 ×2↓	0.665 ×2↓	2
Σ	2	2	2	3	3	4	

64634: $V^{3+}_2(V^{4+}O(P_2O_7)_2$					
	$V1$	$V2$	$P1$	$P2$	∑
$O1$		0.583		1.417	2
$O2$	0.500 ×2↓	0.333	1.167		2
$O3$	0.500 ×2↓	0.333		1.167	2
$O4$		0.583	1.417		2
$O5$		0.583		1.417	2
$O6$		0.583	1.417		2
$O7$			1.000 ×2→		2
$O8$	2.000				2
$O9$				1.000 ×2→	2
∑	4	3	5	5	

65237: $Na_6(O_2(Cu^{3+}O_2)_2)$										
	$Na1$	$Na2$	$Na3$	$Na4$	$Na5$	$Na6$	$Cu1$	$Cu2$	$Cu3$	Σ
$O1$	0.167 ×2↓	0.250 ×2↓	0.167 ×2↓	0.194	0.194	0.250	0.778 ×2↓			2
$O2$				0.139 ×2↓ ×2→	0.139 ×2↓ ×2→		0.722 ×2↓	0.722 ×2↓		2
$O3$	0.167 ×2↓		0.167 ×2↓	0.194	0.194	0.250 ×2↓ ×2→		0.778 ×2↓		2
$O4$				0.139 ×2→	0.139 ×2→				0.722 ×2↓ ×2→	2
$O5$	0.167 ×2↓	0.250 ×2↓	0.167 ×2↓	0.194	0.194	0.250			0.778 ×2↓	2
Σ	1	1	1	1	1	1	3	3	3	

65407: $Na_3Sc_2(PO_4)_3$					
	*Na*1	*Na*2	*Sc*	*P*	Σ
*O*1		0.106 ×4↓ ×2→	0.519 ×3↓	1.269 ×2↓	2
*O*2	0.153 ×6↓	0.067 ×4↓ ×2→	0.481 ×3↓	1.231 ×2↓	2
Σ	0.92	0.693	3	5	

65476: $Ni^{2+}{}_5HfB_2O_{10}$								
	*Ni*1	*Ni*2	*Ni*3	*Ni*4	*Hf*	*B*1	*B*2	Σ
*O*1	0.392 ×2↓		0.425 ×2↓	0.427	0.756 ×2↓			2
*O*2	0.308 ×2↓	0.339 ×4↓ ×2→		0.343	0.672 ×2↓			2
*O*3		0.322		0.326 ×2→		1.025		2
*O*4		0.322		0.326 ×2→			1.025	2
*O*5	0.299			0.334 ×2→		1.033		2
*O*6				0.243 ×2→	0.572		0.942	2
*O*7			0.242 ×2↓ ×2→		0.573	0.943		2
*O*8	0.300		0.333 ×2↓ ×2→				1.034	2
Σ	2	2	2	2	4	3	3	

65512: $Zr(Mo^{6+}O_4)_2$				
	*Zr*1	*Zr*2	*Mo*	Σ
*O*1			2.000	2
*O*2		0.667 ×3↓	1.333	2
*O*3		0.667 ×3↓	1.333	2
*O*4	0.667 ×6↓		1.333	2
Σ	4	4	6	

65614: $Y_2Ba_2Cu^{2+}Pt^{4+}O_8$						
	*Ba*1	*Ba*2	*Cu*	*Y*	*Pt*	Σ
*O*1	0.228 ×4↓	0.163 ×4↓ ×2→	0.380 ×2↓	0.402 ×2↓	0.663 ×4↓	2
*O*2		0.174 ×3↓ ×3→	0.391	0.413	0.674 ×2↓	2
*O*3	0.272 ×4↓	0.207 ×4↓ ×2→	0.424 ×2↓	0.446 ×4↓ ×2→		2
Σ	2	2	2	3	4	

66994: $Cs(Np^{5+}O_2)(Mo^{6+}O_4)$				
	Cs	*Np*	*Mo*	Σ
*O*1	0.384 ×2↓ ×2→	1.232		2
*O*2	0.288 ×3↓ ×3→	1.137		2
*O*3	-0.446	0.402	1.022 ×2↓ ×2→	2
*O*4	-0.292	0.557 ×2↓ ×2→	1.177	2
*O*5	-0.292	0.557 ×2↓ ×2→	1.177	2
*O*6	0.133 ×3↓ ×3→		1.601	2
Σ	1	5	6	

67726: $Cu^{2+}{}_3Ba(V^{5+}O_4)_2(OH)_2$						
	Ba	*Cu*1	*Cu*2	*V*	*H*	Σ
*O*1		0.459 ×2↓	0.459 ×2↓ ×2→		0.623	2
*O*2	0.195 ×4↓	0.270 ×4↓	0.270 ×2↓	1.264 ×2↓		2
*O*3	0.195 ×2↓		0.270 ×2↓ ×2→	1.264		2
*O*4	0.138 ×6↓ ×3→			1.208	0.377	2
Σ	2	2	2	5	1	

68279: $Pr^{3+}_2(Mo^{6+}_4O_{15})$							
	*Pr*1	*Pr*2	*Mo*1	*Mo*2	*Mo*3	*Mo*4	Σ
*O*1		0.433	1.567				2
*O*2		0.433	1.567				2
*O*3	0.507		1.493				2
*O*4	0.388	0.239	1.373				2
*O*5	0.490			1.510			2
*O*6	0.490			1.510			2
*O*7		0.416		1.584			2
*O*8	0.376	0.228		1.396			2
*O*9					1.413	0.587	2
*O*10		0.333			1.667		2
*O*11	0.321	0.172			1.507		2
*O*12					1.413	0.587	2
*O*13						2.000	2
*O*14		0.746				1.254	2
*O*15	0.427					0.786 ×2↓ ×2→	2
Σ	3	3	6	6	6	6	

68614: $Cu^{+}La(W^{6+}{}_2O_8)$					
	Cu	*La*	*W*1	*W*2	∑
*O*1		0.148	0.959	0.892	2
*O*2	-0.136		0.734 ×2↓ ×2→	0.668	2
*O*3		0.148	0.959	0.892	2
*O*4		0.595	1.405		2
*O*5		0.396 ×2↓ ×2→	1.207		2
*O*6	0.379	0.439		1.183	2
*O*7	0.379	0.439		1.183	2
*O*8	0.379	0.439		1.183	2
∑	1	3	6	6	

69088: $Ba(Mo^{5+}{}_2P_4O_{16})$					
	Ba	*Mo*	*P*1	*P*2	∑
*O*1		2.000			2
*O*2	0.124 ×2↓	0.577	1.299		2
*O*3	0.150 ×2↓	0.604		1.246	2
*O*4		0.639	1.361		2
*O*5	0.150 ×2↓	0.604		1.246	2
*O*6	0.124 ×2↓	0.577	1.299		2
*O*7			1.040	0.960	2
*O*8	0.452 ×2↓			1.548	2
∑	2	5	5	5	

71450: $Sr_2(V^{4+}O_4)$			
	Sr	*V*	∑
*O*1	0.179 ×4↓ ×4→	0.641 ×4↓ ×2→	2
*O*2	0.256 ×5↓ ×5→	0.718 ×2↓	2
∑	2	4	

71562: $Ca_2Y(As^{5+}O_4)(W^{6+}O_4)_2$					
	Ca	*Y*	*W*	*As*	∑
*O*1	0.240 ×4↓ ×2→		1.519 ×2↓		2
*O*2	0.202 ×2↓	0.317 ×4↓	1.481 ×2↓		2
*O*3	0.317 ×2↓	0.433 ×4↓		1.250 ×4↓	2
∑	2	3	6	5	

72302: $Cr^{2+}{}_3Cr^{3+}{}_4(PO_4)_6$									
	*Cr*1	*Cr*2	*Cr*3	*Cr*4	*P*1	*P*2	*P*3	*P*4	Σ
*O*1				0.509			1.491		2
*O*2					1.020		0.980		2
*O*3						1.000		1.000	2
*O*4	0.459 ×2↓			0.249		1.293			2
*O*5		0.425 ×2↓	0.383		1.192				2
*O*6	0.583 ×2↓					1.417			2
*O*7	0.459 ×2↓			0.249				1.293	2
*O*8			0.467	0.255	1.277				2
*O*9		0.438 ×2↓	0.396				1.166		2
*O*10		0.636 ×2↓					1.364		2
*O*11			0.710			1.290			2
*O*12			0.584					1.416	2
*O*13			0.460	0.248				1.292	2
*O*14				0.489	1.511				2
Σ	3	3	3	2	5	5	5	5	

72312: $Ba_2(Hg_3Pd^{4+}{}_5Pd^{2+}{}_2O_{14})$								
	Ba	*Hg*1	*Hg*2	*Pd*1	*Pd*2	*Pd*3	*Pd*4	Σ
*O*1	0.250			0.750		0.500 ×2↓ ×2→		2
*O*2	-0.021 ×2↓ ×2→	0.563	1.000 ×2↓	0.479				2
*O*3	0.378 ×2↓ ×2→						0.622 ×2↓ ×2→	2
*O*4	0.250 ×2↓			0.750 ×2↓	0.500 ×4↓	0.500 ×2↓		2
*O*5	0.134 ×4↓ ×2→	0.718 ×2↓		0.635 ×2↓			0.378 ×2↓	2
Σ	2	2	2	4	2	2	2	

72682: $Cs_2(Ti^{4+}O)(P_2O_7)$						
	*Cs*1	*Cs*2	*Ti*	*P*1	*P*2	∑
*O*1	0.009	0.017	0.739	1.235		2
*O*2	0.191 ×2↓ ×2→	0.199		1.418		2
*O*3	0.009	0.017	0.739	1.235		2
*O*4	-0.115	-0.107		1.111	1.111	2
*O*5	0.191 ×2↓ ×2→	0.199			1.418	2
*O*6	0.314	0.321 ×2↓ ×2→	1.044			2
*O*7	0.009	0.017	0.739		1.235	2
*O*8	0.009	0.017	0.739		1.235	2
∑	1	1	4	5	5	

72714: $Ti^{3+}PO_4$							
	*Ti*1	*Ti*2	*P*1	*P*2	*P*3	*P*4	∑
*O*1	0.429 ×2→			1.143			2
*O*2	0.429 ×2→		1.143				2
*O*3	0.429 ×2→					1.143	2
*O*4	0.429 ×2→					1.143	2
*O*5	0.643					1.357 ×2↓	2
*O*6	0.643		1.357 ×2↓				2
*O*7		0.643		1.357 ×2↓			2
*O*8		0.643			1.357 ×2↓		2
*O*9		0.429 ×2→			1.143		2
*O*10		0.429 ×2→			1.143		2
*O*11		0.429 ×2→		1.143			2
*O*12		0.429 ×2→	1.143				2
∑	3	3	5	5	5	5	

72872: $Co^{3+}Re^{5+}O_4$			
	Co	*Re*	∑
*O*1	0.417 ×2↓	0.792 ×4↓ ×2→	2
*O*2	0.542 ×4↓ ×2→	0.917 ×2↓	2
∑	3	5	

72886: $Ca(V^{4+}O)_2(PO_4)_2$				
	Ca	*V*	*P*	∑
*O*1	0.250 ×2↓	0.500	1.250	2
*O*2	0.250 ×2↓	0.500	1.250	2
*O*3		1.000 ×2↓ ×2→		2
*O*4	0.250 ×2↓	0.500	1.250	2
*O*5	0.250 ×2↓	0.500	1.250	2
∑	2	4	5	

73183: $Ba_3Ca(Ru^{5+}{}_2O_9)$					
	*Ba*1	*Ba*2	*Ca*	*Ru*	∑
*O*1	0.119 ×6↓ ×2→	0.095 ×3↓ ×2→		0.786 ×3↓ ×2→	2
*O*2	0.214 ×6↓	0.190 ×9↓ ×3→	0.333 ×6↓	0.881 ×3↓	2
∑	2	2	2	5	

73261: $Cr^{2+}{}_3Cr^{3+}{}_4(PO_4)_6$								
	$Cr1$	$Cr2$	$Cr3$	$Cr4$	$P1$	$P2$	$P3$	$\sum$
$O1$			0.415 ×2↓ ×2→				1.170	2
$O2$			0.460	0.358		1.182		2
$O3$			0.443	0.341			1.216	2
$O4$	0.511			0.409	1.080			2
$O5$	0.460			0.358		1.182		2
$O6$	0.639					1.361		2
$O7$	0.409 ×2↓ ×2→						1.182	2
$O8$		0.568 ×2↓					1.432	2
$O9$		0.174 ×2↓	0.552			1.274		2
$O10$			0.715		1.285			2
$O11$	0.572				1.428			2
$O12$		0.259 ×2↓		0.535	1.207			2
$\sum$	3	2	3	2	5	5	5	

73686: Th($V^{5+}{}_2O_7$)							
	*Th*1	*Th*2	*V*1	*V*2	*V*3	*V*4	∑
*O*1	0.425 ×2↓ ×2→		1.151				2
*O*2	0.637		1.363				2
*O*3		0.665	1.335				2
*O*4	0.425 ×2↓ ×2→		1.151				2
*O*5		0.649		1.351			2
*O*6	0.621			1.379			2
*O*7		0.433 ×2↓ ×2→		1.135			2
*O*8		0.433 ×2↓ ×2→		1.135			2
*O*9	0.291 ×2↓ ×2→				1.417		2
*O*10		0.466			1.534		2
*O*11					1.024	0.976	2
*O*12					1.024	0.976	2
*O*13	0.462					1.538	2
*O*14		0.490				1.510	2
∑	4	4	5	5	5	5	

74212: $CaTi^{4+}O_3$			
	Ca	*Ti*	
*O*1	0.167 ×4↓ ×4→	0.667 ×2↓ ×2→	2
*O*2	0.167 ×8↓ ×4→	0.667 ×4↓ ×2→	2
	2	4	

75264: $Li_3Nb^{5+}O_4$			
	Li	*Nb*	∑
O1$_{br}$	-0.042 ×3→	0.708 ×3↓ ×3→	2
O2	0.208 ×5↓ ×5→	0.958 ×3↓	2
∑	1	5	

75583: $La_2(Ti^{4+}{}_2SiO_9)$						
	*La*1	*La*2	*Ti*1	*Ti*2	*Si*	∑
*O*1	0.280 ×2↓	0.186 ×2↓	0.619 ×2↓		0.914 ×2↓	2
*O*2		0.668			1.332	2
*O*3	0.268	0.174 ×2↓ ×2→		0.544	0.839	2
*O*4	0.454 ×4↓ ×2→	0.361 ×2		0.731 ×2↓		2
*O*5	0.354	0.260	0.693 ×2↓ ×2→			2
*O*6		0.315 ×2↓ ×2→		0.685 ×2↓ ×2→		2
*O*7			0.688 ×2↓ ×2→	0.625		2
∑	3	3	4	4	4	

78180: $La_2(W^{6+}O_4)_3$				
	La	*W*1	*W*2	∑
*O*1	0.095	0.952 ×2↓ ×2→		2
*O*2	0.381 ×2↓ ×2→	1.238		2
*O*3	0.571	1.429		2
*O*4	0.429		1.571 ×2↓	2
*O*5	0.571	1.429		2
*O*6	0.286 ×2↓ ×2→		1.429 ×2↓	2
∑	3	6	6	

78842: $Rb_2(Ti^{4+}O_3)$				
	*Rb*1	*Rb*2	*Ti*	∑
*O*1	0.185 ×2↓ ×2→	0.1885 ×3↓ ×3→	1.074	2
*O*2	0.185 ×3↓ ×3→	0.185 ×2↓ ×2→	1.074	2
*O*3	0.037 ×2↓ ×2→	0.037 ×2↓ ×2→	0.926 ×2↓ ×2→	2
∑	1	1	4	

79517: $Cs(Mo^{6+}{}_2O_3(PO_4)_2)$						
	Cs	*Mo*1	*Mo*2	*P*1	*P*2	∑
*O*1	0.382 ×2↓ ×2→	1.237				2
*O*2	-0.087	0.768		1.319		2
*O*3		0.724		1.276		2
*O*4	-0.083	0.772			1.311	2
*O*5	-0.087	0.768		1.319		2
*O*6		0.731			1.269	2
*O*7	0.255 ×2↓ ×2→		1.491			2
*O*8	0.255 ×2↓ ×2→		1.491			2
*O*9			0.915	1.085		2
*O*10	-0.210		1.026		1.184	2
*O*11	-0.157 ×2↓ ×2→		1.078		1.236	2
∑	1	5	6	5	5	

79702: $K_2Ni^{2+}(W^{6+}O_2(PO_4)_2)$					
	K	*Ni*	*W*	*P*	Σ
*O*1	-0.059		0.908 ×2↓	1.151	2
*O*2	-0.059		0.908 ×2↓	1.151	2
*O*3	0.125 ×3↓ ×3→	0.292 ×2↓		1.334	2
*O*4	0.156 ×2↓ ×2→	0.324 ×2↓		1.365	2
*O*5	0.216 ×2↓ ×2→	0.384 ×2↓	1.183 ×2↓		2
Σ	1	2	6	5	

79734: $K_3(Nb^{5+}{}_3O_6)(Si_2O_7)$				
	K	*Nb*	*Si*	Σ
*O*1	0.017 ×3→		0.975 ×2→	2
*O*2	0.105 ×3↓ ×3→	0.843 ×2↓ ×2→		2
*O*3	0.131 ×2↓ ×2→	0.869 ×2↓ ×2→		2
*O*4	0.051 ×8↓ ×4→	0.788 ×2↓	1.008 ×3↓	2
Σ	1	5	4	

80423: $CsTa^{5+}(B_2O_5)$				
	Cs	*Ta*	*B*	Σ
*O*1	0.114 ×4↓ ×2→	0.750 ×2↓	1.023	2
*O*2	0.114 ×4↓ ×2→	0.750 ×2↓	1.023	2
*O*3		1.000 ×2↓ ×2→		2
*O*4	0.045 ×2↓ ×2→		0.955 ×2→	2
Σ	1	5	3	

80430: $KMn^{3+}(Se^{6+}O_4)_2$				
	K	*Mn*	*Se*	∑
*O*1		0.512 ×2↓	1.488	2
*O*2	0.142 ×6↓×3		1.574	2
*O*3	0.037 ×2↓	0.494 ×2↓	1.469	2
*O*4	0.037 ×2↓	0.494 ×2↓	1.469	2
∑	1	3	6	

81473: $BaCo^{2+}_2(Si_2O_7)$							
	Ba	*Co*1	*Co*2	*Co*3	*Si*1	*Si*2	∑
*O*1			0.526 ×2↓	0.449	1.025		2
*O*2	0.246 ×2↓ ×2→		0.474 ×2↓			1.035	2
*O*3	0.227			0.379 ×2↓ ×2→		1.016	2
*O*4	0.210	0.429 ×2↓		0.362		0.999	2
*O*5	0.161				0.889	0.950	2
*O*6	0.351	0.571 ×2↓			1.079		2
*O*7	0.280 ×2↓ ×2→			0.432	1.008		2
∑	2	2	2	2	4	4	

82403: $Co^{2+}{}_2Si(P_2O_7)_2$					
	Co	*Si*	*P*1	*P*2	∑
*O*1		1.000 ×2↓	1.000		2
*O*2	0.429		1.571		2
*O*3	0.286 ×2↓ ×2→		1.429		2
*O*4			1.000	1.000	2
*O*5	0.429			1.571	2
*O*6	0.286 ×2↓ ×2→			1.429	2
*O*7		1.000 ×2↓		1.000	2
∑	2	4	5	5	

82488: $Na_4Zr_2Ti^{4+}O_4(CO_3)_4$							
	*Na*1	*Na*2	*Zr*	*Ti*	*C*1	*C*2	∑
*O*1	0.064	0.064		0.595 ×2↓	1.277		2
*O*2	0.091	0.091	0.484			1.333	2
*O*3	0.171		0.563 ×2↓ ×2→	0.702 ×2↓			2
*O*4		0.171	0.563 ×2↓ ×2→	0.702 ×2↓			2
*O*5	0.197 ×2↓ ×2→	0.197			1.410		2
*O*6	-0.007		0.386 ×2↓ ×2→			1.235	2
*O*7	0.098	0.098	0.491		1.312		2
*O*8	0.189	0.190 ×2↓ ×2→				1.431	2
∑	1	1	4	4	4	4	

82685: $SrV^{3+}{}_2O(PO_4)_2$						
	Sr	*V*1	*V*2	*P*1	*P*2	Σ
*O*1	0.273 ×2↓	0.472 ×2↓			1.255 ×2↓	2
*O*2	0.269	0.468		1.263		2
*O*3		0.609			1.391	2
*O*4	0.269	0.468		1.263		2
*O*5	0.312	0.511	0.588 ×2↓ ×2→			2
*O*6	0.117		0.392 ×2↓ ×2→		1.099	2
*O*7	0.243 ×2↓		0.519 ×2↓	1.237 ×2↓		2
Σ	2	3	3	5	5	

83285: $K(Fe^{3+}{}_{11}O_{17})$						
	K	*Fe*1	*Fe*2	*Fe*3	*Fe*4	Σ
*O*1		0.379 ×2↓ ×2→	0.742 ×3↓		0.500 ×6↓	2
*O*2	0.167 ×6↓	0.583 ×2↓ ×2→		0.667 ×3↓		2
*O*3		0.409 ×3→	0.773			2
*O*4		0.667 ×3→				2
*O*5	0.000 ×3↓ ×3→			1.000 ×2→		2
Σ	1	3	3	3	3	

85042: $Mn^{2+}Zn_2Ta^{5+}{}_2O_8$				
	Zn	*Mn*	*Ta*	Σ
*O*1	0.410 ×3↓ ×3→		0.769	2
*O*2	0.256 ×3↓ ×3→		0.615 ×2↓ ×2→	2
*O*3		0.417 ×4↓ ×2→	1.167	2
*O*4		0.167 ×2↓	0.917×2↓ ×2→	2
Σ	2	2	5	

85497: $NaY(GeO_4)$				
	Na	*Y*	*Ge*	Σ
*O*1	0.197 ×2↓ ×2→	0.561	1.045	2
*O*2	0.197 ×2↓ ×2→	0.561	1.045	2
*O*3	0.106 ×2↓	0.470 ×4↓ ×2→	0.955 ×2↓	2
Σ	1	3	4	

85735: $ZnLiNb^{5+}O_4$				
	Li	*Zn*	*Nb*	Σ
*O*1	0.212 ×4↓ ×2→	0.818 ×2↓	0.758 ×2↓	2
*O*2	0.076 ×2↓	0.682 ×2↓	0.621 ×4↓ ×2→	2
Σ	1	2	5	

86144: $W^{6+}O_3$		
	W	Σ
*O*1	1.000 ×2↓ ×2→	2
*O*2	1.000 ×4↓ ×2→	2
Σ	6	

88879: $Nb^{4+}O2$			
	*Nb*1	*Nb*2	Σ
*O*1	0.667	0.667 ×2↓ ×2→	2
*O*2	0.667 ×2↓ ×2→	0.667	2
*O*3	0.667	0.667 ×2↓ ×2→	2
*O*4	0.667 ×2↓ ×2→	0.667	2
Σ	4	4	

89466: $V^{5+}{}_2Se^{4+}{}_2O_9$					
	*Se*1	*Se*2	*V*1	*V*2	Σ
*O*1			2.000		2
*O*2	1.200		0.400	0.400	2
*O*3		1.400	0.600		2
*O*4	1.400		0.600		2
*O*5			1.000	1.000	2
*O*6		1.200	0.400	0.400	2
*O*7	1.400			0.600	2
*O*8		1.400		0.600	2
*O*9				2.000	2
Σ	4	4	5	5	

89506: $K(Mn^{7+}O_4)$			
	K	*Mn*	Σ
*O*1	0.083 ×3↓ ×3→	1.750	2
*O*2	0.083 ×3↓ ×3→	1.750	2
*O*3	0.083 ×6↓×3	1.750 ×2↓	2
Σ	1	7	

90110: $Tl^{+}_2(Te^{4+}Mo^{6+}_2O_6(PO_4)_2)$					
	Tl	*Mo*	*P*	*Te*	Σ
*O*1	0.005	0.924		1.071 ×2↓	2
*O*2	-0.066 ×2↓ ×2→	0.853	1.279		2
*O*3	0.360 ×2↓ ×2→	1.280			2
*O*4	0.360 ×2↓ ×2→	1.280			2
*O*5	-0.088	0.831	1.257		2
*O*6	-0.088	0.831	1.257		2
*O*7	-0.137		1.208	0.929 ×2↓	2
Σ	1	6	5	4	

91748: $Mg_4Nb^{5+}_2O_9$				
	*Mg*1	*Mg*2	*Nb*	Σ
*O*1		0.250 ×3↓ ×2→	0.750 ×3↓ ×2→	2
*O*2	0.333 ×6↓ ×2→	0.417 ×3↓	0.917 ×3↓	2
Σ	2	2	5	

92317: $(V^{4+}O)(Re^{7+}O_4)_2$				
	V	*Re*1	*Re*2	Σ
*O*1	0.333 ×2↓	1.667 ×2↓		2
*O*2	0.333	1.667		2
*O*3		2.000		2
*O*4	0.500 ×2↓		1.500 ×2↓	2
*O*5			2.000	2
*O*6			2.000	2
*O*7	2.000			2
Σ	4	7	7	

92489: $Eu^{3+}_4(Au^{3+}_2O_9)$				
	Au	*Eu*1	*Eu*2	Σ
*O*1		0.500 ×2→	0.500 ×2→	2
*O*2	0.750	0.417 ×2↓ ×2→	0.417	2
*O*3	0.750	0.417	0.417 ×2↓ ×2→	2
*O*4	0.750	0.417 ×2↓ ×2→	0.417	2
*O*5	0.750	0.417	0.417 ×2↓ ×2→	2
Σ	3	3	3	

92508: $Pr_3(Re^{7+}O_8)$					
	*Pr*1	*Pr*2	*Pr*3	*Re*	Σ
*O*1	0.484 ×2↓ ×2→	0.518	0.513		2
*O*2	0.484 ×2↓ ×2→	0.518	0.513		2
*O*3	0.276 ×2→		0.305 ×2↓	1.144	2
*O*4	0.266	0.300 ×2↓ ×2→		1.134	2
*O*5		0.390	0.385	1.225	2
*O*6	0.267	0.301	0.296	1.136	2
*O*7	0.255	0.289	0.294	1.133	2
*O*8		0.384	0.388	1.228	2
Σ	3	3	3	7	

94743: $NaKLaNb^{5+}O_5$					
	La	*Nb*	*Na*	*K*	Σ
*O*1	0.375 ×8↓ ×2→	0.978 ×4↓	0.178 ×4↓	0.047 ×8↓ ×2→	2
*O*2		1.088	0.288	0.156 ×4↓ ×4→	2
Σ	3	5	1	1	

95493: Gd($Mn^{3+}O_3$)			
	Gd	*Mn*	∑
*O*1	0.441 ×2↓ ×2→	0.559 ×2↓ ×2→	2
*O*2	0.353 ×6↓×3	0.471 ×4↓ ×2→	2
∑	3	3	

95929: $KMg(V^{5+}{}_5O_{14})(H_2O)_8$																								
	*V*1	*V*2	*V*3	*V*4	*V*5	*K*	*Mg*	*H*1	*H*2	*H*3	*H*4	*H*5	*H*6	*H*7	*H*8	*H*9	*H*10	*H*11	*H*12	*H*13	*H*14	*H*15	*H*16	Σ
*O*1		1.510				0.049													0.441					2
*O*2		0.870			0.756					0.137		0.236												2
*O*3	0.845	0.573	0.609															-0.027						2
*O*4	1.111				0.725			0.164																2
*O*5	1.102			0.682									0.216											2
*O*6	0.553 ×2↓ ×2→	0.280	0.316	0.132	0.166																			2
*O*7			1.568			0.071											0.361							2
*O*8			0.988	0.804					0.137							0.071								2
*O*9			0.920		0.770															0.155	0.155			2
*O*10		1.20		1.05		-																		2

		3		5		0.258																		
*O*11				0.876	0.910						0.214													2
*O*12				1.451		0.139								0.410										2
*O*13	0.836	0.564	0.600																					2
*O*14					1.673	0.327																		2
*O*15 (*O*I1)							0.301	0.836	0.863															2
*O*16 (*O*I2)						0.135	0.217			0.863	0.786													2
*O*17 (*O*I3)						-0.063						0.764	0.784		0.309								0.207	2
*O*18 (*O*I4)						0.320								0.590	0.691							0.398		2
*O*19 (*O*I5)							0.432									0.929	0.639							2
*O*20 (*O*I6)						0.166	0.249											1.027	0.559					2
*O*21						0.114	0.19													0.84	0.84			2

(*O*I7)							6													5	5			
*O*22<br>(*O*I8)							0.60<br>5															0.60<br>2	0.79<br>3	2
Σ	5	5	5	5	5	1	2	1	1	1	1	1	1	1	1	1	1	1	1	1	1	1	1	

96359: $SrFe^{3+}{}_3(PO_4)_3O$								
	*Sr*1	*Fe*1	*Fe*2	*Fe*3	*P*1	*P*2	*P*3	Σ
*O*1		0.671 ×2↓ ×2→	0.658					2
*O*2	0.239 ×2↓	0.467 ×2↓			1.295 ×2↓			2
*O*3	0.077 ×2↓ ×2→		0.292	0.434	1.120			2
*O*4	0.247		0.462		1.290			2
*O*5	0.259 ×2↓		0.474 ×2↓			1.267 ×2↓		2
*O*6	0.134	0.362 ×2↓ ×2→				1.141		2
*O*7				0.675		1.325		2
*O*8	0.235 ×2↓			0.591 ×2↓			1.174 ×2↓	2
*O*9			0.638				1.362	2
*O*10				0.709			1.291	2
Σ	2	3	3	3	5	5	5	

96454: $Pb^{2+}_2(Mo^{4+}_2O(PO_4)_2(P_2O_7))$					
	*Pb*2	*Mo*	*P*1	*P*2	Σ
*O*1		1.000 ×2→			2
*O*2	0.206	0.612		1.182	2
*O*3	0.206	0.612		1.182	2
*O*4		0.629	1.371		2
*O*5		0.629	1.371		2
*O*6	0.112 ×2↓ ×2→	0.518	1.259		2
*O*7			1.000 ×2→		2
*O*8	0.341 ×2↓ ×2→			1.318	2
*O*9	0.341 ×2↓ ×2→			1.318	2
Σ	2	4	5	5	

97525: $Ba_6Ru^{5+}_2Na_2Mn^{5+}_2O_{17}$								
	Na	*Ba*1	*Ba*2	*Ba*3	*Ba*4	*Mn*	*Ru*	Σ
*O*1	0.167 ×3↓		0.167 ×6↓ ×2→	0.333 ×6↓		1.167 ×3↓		2
*O*2	0.167 ×3↓	0.267 ×6↓ ×2→	0.167 ×3↓		0.233 ×6↓		0.900 ×3↓	2
*O*3		0.133 ×3↓ ×2→			0.100 ×6↓ ×2→		0.767 ×3↓ ×2→	2
*O*4			0.500			1.500		2
Σ	1	2	2	2	2	5	5	

99594: $Mn^{2+}V^{5+}Sb^{5+}O_6$				
	Mn	*V*	*Sb*	∑
*O*1	0.133 ×2↓	0.833 ×2↓	1.033 ×2↓	2
*O*2	0.433 ×4↓ ×2→	1.133 ×2↓		2
*O*3		0.533 ×2↓	0.733 ×4↓ ×2→	2
∑	2	5	5	

100082: $CaMn^{2+}_2(BeSiO_4)_3$										
	Ca	*Mn*1	*Mn*2	*Be*1	*Be*2	*Be*3	*Si*1	*Si*2	*Si*3	∑
*O*1	0.194 ×2↓ ×2→					0.556	1.056			2
*O*2	0.194 ×2↓ ×2→			0.556				1.056		2
*O*3	0.194 ×2↓ ×2→				0.556				1.056	2
*O*4		0.306	0.306	0.444			0.944			2
*O*5		0.306	0.306		0.444			0.944		2
*O*6		0.306	0.306			0.444			0.944	2
*O*7	0.139		0.361		0.500		1.000			2
*O*8	0.139	0.361				0.500		1.000		2
*O*9	0.139		0.361	0.500					1.000	2
*O*10	0.139	0.361		0.500			1.000			2
*O*11	0.139		0.361		0.500			1.000		2
*O*12	0.139	0.361				0.500			1.000	2
∑	2	2	2	2	2	2	4	4	4	

100158: $Na_2Ca_4ZrNb^{5+}(Si_2O_7)_2FO_3$													
	*Na*1	*Na*2	*Ca*1	*Ca*2	*Ca*3	*Ca*4	*Zr*	*Nb*	*Si*1	*Si*2	*Si*3	*Si*4	Σ
*O*1				0.355		0.349		0.762	0.534				2
*O*2	-0.019			0.435		0.428				1.156			2
*O*3	-0.252			0.202				0.758	1.292				2
*O*4	-0.233					0.364		0.777		1.092			2
*O*5			0.124				0.750		1.126				2
*O*6	0.170		0.140				0.767			0.923			2
*O*7		0.445	0.819		0.578						0.158		2
*O*8		0.738			0.871		0.004					0.386	2
*O*9		-0.206	0.168				0.795				1.242		2
*O*10			0.151		-0.090		0.778					1.160	2
*O*11		-0.003		0.282		0.276					1.445		2
*O*12				0.265	0.113	0.259						1.363	2
*O*13	0.309				0.038		0.906	0.747					2
*O*14	0.499	0.095	0.469					0.937					2
*O*1		0.176		0.46	0.309			1.01					2

5				1				8					
*O*16	0.076		0.046						1.048	0.829			2
*O*17		-0.292	0.082								1.155	1.091	2
F	0.450	0.046			0.179	0.325							1
∑	1	1	2	2	2	2	4	5	4	4	4	4	

100278: $ScAlO_3$			
	Sc	*Al*	∑
*O*1	0.441 ×2↓ ×2→	0.559 ×2↓ ×2→	2
*O*2	0.353 ×6↓ ×3→	0.471 ×4↓ ×2→	2
∑	3	3	

100571: $Ba_{10}(Re^{7+}O_5)_6Br_2$				
	*Ba*1	*Ba*2	*Re*	∑
*O*1		0.195 ×3↓ ×3→	1.414	2
*O*2	0.218 ×6↓ ×2→	0.172 ×2↓	1.391 ×2↓	2
*O*3	0.230 ×3↓	0.184 ×4↓ ×2→	1.402 ×2↓	2
Br		0.167 ×2↓ ×6→		1
∑	2	2	7	

156736: $Ca_3Mn^{2+}(Sb^{5+}{}_4O_{14})$							
	Mn	*Ca*1	*Ca*2	*Sb*1	*Sb*2	*Sb*3	∑
*O*1	0.280 ×2↓	0.154 ×2↓			0.771 ×2↓	0.795	2
*O*2			0.244			0.878 ×2↓ ×2→	2
*O*3	0.278 ×2↓		0.159		0.769 ×2↓	0.793	2
*O*4		0.343 ×2↓	0.349 ×2↓ ×2→		0.960 ×2↓		2
*O*5	0.442 ×2↓	0.316 ×2↓	0.322	0.920 ×2↓			2
*O*6		0.187 ×2↓	0.194	0.791 ×2↓		0.828	2
*O*7			0.192 ×2↓ ×2→	0.789 ×2↓		0.827	2
∑	2	2	2	5	5	5	

157733: $NaFe^{3+}Si_2O_6$				
	Na	*Fe*	*Si*	∑
*O*1	0.113 ×2↓	0.45 ×4↓ ×2→	0.988	2
*O*2	0.263 ×2↓	0.6 ×2↓	1.138	2
*O*3	0.062 ×4↓ ×2→		0.938 ×2↓ ×2→	2
∑	1	3	4	

170119: $K_2Mo^{6+}O_2(I^{5+}O_3)_4$					
	K	*I*1	*I*2	*Mo*	∑
*O*1	-0.143	0.502	0.593	1.048 ×2↓	2
*O*2	0.178 ×2↓ ×2→	0.822 ×2↓ ×2→			2
*O*3	0.155 ×2↓ ×2→	0.800	0.891		2
*O*4		0.454	0.545	1.001 ×2↓	2
*O*5	0.155 ×2↓ ×2→	0.800	0.891		2
*O*6	0.155 ×2↓ ×2→	0.800	0.891		2
*O*7	-0.141		0.595 ×2↓ ×2→	0.951 ×2↓	2
∑	1	5	5	6	

171028: $Cu^{2+}_2V^{5+}_2O_7$					
	*Cu*1	*Cu*2	*V*1	*V*2	∑
*O*1	0.356 ×2↓ ×2→			1.288	2
*O*2	0.355 ×2↓ ×2→		1.291		2
*O*3		0.363 ×2↓ ×2→	1.274		2
*O*4		0.365 ×2↓ ×2→		1.271	2
*O*5	0.044		0.980	0.976	2
*O*6	0.534			1.466	2
*O*7		0.545	1.455		2
∑	2	2	5	5	

171758: $Na_2Mo^{6+}_3Te^{4+}_3O_{16}$							
	*Na*1	*Na*2	*Mo*1	*Mo*2	*Te*1	*Te*2	∑
*O*1				0.975	1.025 ×2↓		2
*O*2	-0.087 ×2↓				0.975 ×2↓	1.112	2
*O*3			0.772 ×2↓	0.521		0.707	2
*O*4	-0.058 ×2↓	0.323 ×2↓		0.956		1.142	2
*O*5			1.125 ×2↓	0.875			2
*O*6		-0.142 ×2↓	1.103 ×2↓			1.039	2
*O*7	0.323 ×2↓	0.341 ×2↓		1.336			2
*O*8	0.323 ×2↓	0.341 ×2↓		1.336			2
∑	1	1	6	6	4	4	

200128: $LiSc(SiO_3)_2$				
	Li	*Sc*	*Si*	∑
*O*1	0.125 ×2↓	0.450 ×4↓ ×2→	0.975	2
*O*2	0.275 ×2↓	0.600 ×2↓	1.125	2
*O*3	0.100 ×2↓		0.950 ×2↓ ×2→	2
∑	1	3	4	

200743: $Cd_2P_6O_{17}$					
	*Cd*1	*P*1	*P*2	*P*3	∑
*O*1		0.898	1.102		2
*O*2	0.398	1.602			2
*O*3	0.398	1.602			2
*O*4		0.898	1.102		2
*O*5	0.296		1.704		2
*O*6			1.093	0.907	2
*O*7				1.000 ×2→	2
*O*8	0.389			1.611	2
*O*9	0.259 ×2↓ ×2→			1.481	2
∑	2	5	5	5	

200854: $RbNb^{5+}O_3$						
	*Rb*1	*Rb*2	*Rb*3	*Nb*1	*Nb*2	∑
*O*1		0.094 ×2↓	0.033 ×2↓ ×2→	0.917	0.923	2
*O*2	-0.240 ×2↓			0.557 ×2↓ ×2→	0.563 ×2↓ ×2→	2
*O*3		0.102 ×2↓	0.041	0.925	0.931	2
*O*4	0.121 ×2↓		0.035	0.919	0.925	2
*O*5	0.291 ×2↓		0.205 ×3↓ ×3→		1.095	2
*O*6	0.328 ×2↓	0.303 ×2↓	0.242	1.126		2
∑	1	1	1	5	5	

201658: $V^{4+}O(HPO_4)(H_2O)_{0.5}$				
	V	*P*	*H*	Σ
OH		0.739	0.261	1
*O*2	0.522 ×2↓	1.478 ×2		2
*O*3	0.348 ×2↓ ×2→	1.304		2
*O*4	2.000			2
*O*5	0.261 ×2→		0.739 ×2→	2
Σ	4	5	1	

201733: $Cu^{2+}_3(As^{5+}O_4)_2$				
	*Cu*1	*Cu*2	*As*	Σ
*O*1	0.500 ×2↓	0.327	1.173	2
*O*2		0.577	1.423	2
*O*3		0.385 ×2↓ ×2→	1.231	2
*O*4	0.500 ×2↓	0.327	1.173	2
Σ	2	2	5	

201793: $Tl^{3+}_2(Cr^{6+}O_4)_3$				
	Tl	*Cr*1	*Cr*2	Σ
*O*1	0.400 ×2↓ ×2→	1.200		2
*O*2		2.000		2
*O*3	0.600	1.400		2
*O*4	0.500		1.500 ×2↓	2
*O*5	0.600	1.400		2
*O*6	0.500		1.500 ×2↓	2
Σ	3	6	6	

202414: $W^{5+}{}_8P_8O_{40}$			
	W	*P*	∑
*O*1	1.000 ×2↓ ×2→		2
*O*2	0.500	1.500	2
*O*3	0.750	1.250	2
*O*4	1.000 ×2→		2
*O*5	0.750	1.250	2
*O*6		1.000 ×2→	2
∑	5	5	

203048: $PW^{5+}O_5$			
	W	*P*	∑
*O*1	0.75	1.25	2
*O*2	0.75	1.25	2
*O*3	0.75	1.25	2
*O*4	0.75	1.25	2
*O*5	1 ×2↓ ×2→		2
∑	5	5	

203232: $DyTa^{5+}{}_7O_{19}$				
	Dy	*Ta*1	*Ta*2	∑
*O*1			1.000 ×2→	2
*O*2	0.417 ×6↓	0.833 ×6↓	0.750	2
*O*3			0.667 ×3↓ ×3→	2
*O*4			0.667 ×3→	2
*O*5	0.250 ×2↓		0.583 ×3→	2
∑	3	5	5	

247056: $LiCr^{3+}(Mo^{6+}{}_4)_2$					
	Li	*Cr*	*Mo*1	*Mo*2	∑
*O*1		0.385 ×2↓ ×2→		1.230	2
*O*2	0.317			1.683	2
*O*3		0.562	1.438		2
*O*4		0.562	1.438		2
*O*5	0.038	0.559		1.404	2
*O*6	0.027	0.548	1.425		2
*O*7	0.301		1.699		2
*O*8	0.317			1.683	2
∑	1	3	6	6	

248227: $K_3V^{5+}{}_5O_{14}$				
	K	*V*1	*V*2	
*O*1	0.190 ×2↓ ×3→	1.430		2
*O*2	0.260 ×3↓ ×3→		1.220	2
*O*3	0.02 ×2↓ ×2→		0.980 ×2↓ ×2→	2
*O*4	-0.050 ×4↓ ×2→	1.190 ×3↓	0.910 ×2↓	2
	1	5	5	

249142: $Pb^{2+}{}_2(V^{4+}O(PO_4)_2)$						
	*Pb*1	*Pb*2	*V*	*P*1	*P*2	∑
*O*1	0.123		0.648		1.229	2
*O*2		0.531		1.469		2
*O*3	0.329 ×2↓ ×2→	0.202		1.140		2
*O*4	0.329 ×2↓ ×2→	0.202		1.140		2
*O*5	0.123		0.648		1.229	2
*O*6		0.039	0.690		1.271	2
*O*7		0.039	0.690		1.271	2
*O*8	0.438	0.312		1.250		2
*O*9		0.675	1.325			2
∑	2	2	4	5	5	

250466: $Na_2(Co^{2+}(NO_3)_4)$								
	*Na*1	*Na*2	*Co*	*N*1	*N*2	*N*3	*N*4	∑
*O*1	0.101		0.271	1.628				2
*O*2	0.236			1.764				2
*O*3		0.140	0.252	1.609				2
*O*4	0.101		0.271		1.628			2
*O*5		0.140	0.252		1.609			2
*O*6	0.236				1.764			2
*O*7	0.119	0.177				1.704		2
*O*8		0.120	0.232			1.648		2
*O*9		0.120	0.232			1.648		2
*O*10	0.085		0.255				1.661	2
*O*11		0.123	0.235				1.641	2
*O*12	0.122	0.180					1.698	2
∑	1	1	2	5	5	5	5	

280066: $Cs_2(Mo^{6+}{}_3O_{10})$				
	Cs	*Mo*1	*Mo*2	∑
*O*1	0.192 ×3↓ ×3→	1.424 ×2↓		2
*O*2	-0.172 ×2↓ ×2→	1.060 ×2↓	1.285	2
*O*3		0.516 ×2↓	0.742 ×2↓ ×2→	2
*O*4	0.181 ×2↓ ×2→		1.638	2
*O*5	0.136 ×3↓ ×3→		1.593	2
∑	1	6	6	

280154: $Na_2Ca_3Ta^{5+}{}_2O_9$					
	Na	*Ca*1	*Ca*2	*Ta*	∑
*O*1	0.167 ×6↓ ×2→	0.500 ×3↓	0.333 ×6↓	0.833 ×3↓	2
*O*2		0.167 ×3↓ ×2→		0.833 ×3↓ ×2→	2
∑	1	2	2	5	

280292: $Hg^{2+}(PO_3)_2$				
	Hg	*P*1	*P*2	∑
*O*1	0.429	1.571		2
*O*2	0.286 ×2↓ ×2→	1.429		2
*O*3	0.286 ×2↓ ×2→		1.429	2
*O*4	0.429		1.571	2
*O*5		1.000	1.000	2
*O*6		1.000	1.000	2
∑	2	5	5	

280309: $SrCr^{2+}(P_2O_7)$					
	Sr	*Cr*	*P*1	*P*2	Σ
*O*1	0.252	0.412	1.336		2
*O*2	0.253	0.413		1.334	2
*O*3	0.189 ×2↓ ×2→	0.349	1.273		2
*O*4			1.002	0.998	2
*O*5	0.305 ×2↓ ×2→		1.389		2
*O*6	0.253	0.413		1.334	2
*O*7	0.253	0.413		1.334	2
Σ	2	2	5	5	

280501: $Rb(Ti^{4+}O)(As^{5+}O_4)$							
	*Rb*1	*Rb*2	*Ti*1	*Ti*2	*As*1	*As*2	Σ
*O*1	0.226	0.187	0.363		1.224		2
*O*2	0.226	0.187	0.363		1.224		2
*O*3	0.209	0.171		0.379	1.241		2
*O*4		0.240		0.449	1.310		2
*O*5	0.433	0.394	0.570	0.603			2
*O*6	0.039	0.000	0.964	0.997			2
*O*7	-0.042	-0.081	0.883			1.241	2
*O*8	-0.070		0.856			1.213	2
*O*9	-0.010	-0.049		0.786		1.273	2
*O*10	-0.010	-0.049		0.786		1.273	2
Σ	1	1	4	4	5	1.241	

280589: $YMn^{3+}O_3$				
	*Y*1	*Y*2	*Mn*	∑
*O*1	0.444 ×3↓	0.444 ×3↓ ×2→	0.667	2
*O*2	0.444 ×3↓	0.444 ×3↓ ×2→	0.667	2
*O*3	0.333		0.556 ×3→	2
*O*4		0.333	0.556 ×2↓ ×3→	2
∑	3	3	3	

280775: $V^{5+}AlMo^{6+}O_7$				
	Al	*V*	*Mo*	∑
*O*1	0.500 ×2↓		1.500 ×2↓	2
*O*2	0.500		1.500	2
*O*3	0.500		1.500	2
*O*4	0.500 ×2↓	0.750 ×4↓ ×2→		2
*O*5		2.000		2
∑	3	5	6	

280902: $NaZnFe^{3+}_2(PO_4)_3$								
	Na	*Zn*	*Fe*1	*Fe*2	*P*1	*P*2	*P*3	Σ
*O*1	0.252	0.416			1.332			2
*O*2				0.775	1.225			2
*O*3	0.186		0.549		1.265			2
*O*4	0.098	0.263	0.462		1.178			2
*O*5			0.621			1.379		2
*O*6	0.084			0.712		1.204		2
*O*7	0.088	0.252	0.451			1.209		2
*O*8	0.088	0.252	0.451			1.209		2
*O*9		0.550					1.450	2
*O*10	0.103			0.731			1.166	2
*O*11				0.782			1.218	2
*O*12	0.102	0.266	0.465				1.166	2
Σ	1	2	3	3	5	5	5	

281197: $Pr_3Mo^{5+}O_7$					
	*Pr*1	*Pr*2	*Pr*3	*Mo*	Σ
*O*1	0.354	0.355	0.414	0.877	2
*O*2		0.355 ×2↓ ×2→	0.414	0.877	2
*O*3	0.470 ×2↓ ×2→		0.530 ×2↓ ×2→		2
*O*4	0.354	0.355	0.414	0.877	2
*O*5		0.224	0.283	0.746 ×2↓ ×2→	2
*O*6	0.354	0.355	0.414	0.877	2
*O*7	0.499 ×2↓ ×2→	0.501 ×2↓ ×2→			2
Σ	3	3	3	5	

281210: $NaAl(Mo^{6+}O_4)_2$				
	Na	*Al*	*Mo*	∑
*O*1	0.062 ×2↓	0.500 ×2↓	1.438	2
*O*2	0.062 ×2↓	0.500 ×2↓	1.438	2
*O*3	0.063 ×2↓	0.500 ×2↓	1.438	2
*O*4	0.313 ×2↓		1.688	2
∑	1	3	6	

281503: $BaTe^{4+}Mo^{6+}{}_2O_9$					
	Ba	*Te*	*Mo*1	*Mo*2	∑
*O*1		0.829	0.580	0.592	2
*O*2	0.095	1.071		0.834	2
*O*3		0.829	0.580	0.592	2
*O*4	-0.147 ×2↓ ×2→	1.272	1.023		2
*O*5	0.277 ×2↓ ×2→		1.447		2
*O*6	0.208 ×3↓ ×3→		1.377		2
*O*7			0.994	1.006	2
*O*8	0.409			1.591	2
*O*9	0.205 ×3↓ ×3→			1.386	2
∑	2	4	6	6	

400438: $Cu^{2+}{}_2Co^{2+}O(B_2O_5)$						
	*Cu*1	*Cu*2	*Co*	*B*1	*B*2	∑
*O*1		0.373 ×2↓ ×2→	0.231	1.023		2
*O*2	0.470	0.604	0.463 ×2↓ ×2→			2
*O*3	0.304 ×3↓ ×3→			1.089		2
*O*4		0.412	0.270 ×2↓ ×2→		1.047	2
*O*5	0.309 ×2↓ ×2→		0.302		1.079	2
*O*6		0.238		0.888	0.874	2
∑	2	2	2	3	3	

400764: $SrCo^{2+}{}_2(As^{5+}O_4)_2$						
	Sr	*Co*1	*Co*2	*As*1	*As*2	∑
*O*1		0.384	0.381		1.235	2
*O*2		0.273	0.269 ×2↓ ×2→	1.189		2
*O*3	0.169	0.305 ×2↓ ×2→		1.221		2
*O*4	0.170	0.306	0.302	1.222		2
*O*5	0.293	0.428			1.279	2
*O*6	0.294		0.426		1.280	2
*O*7	0.316 ×2↓ ×2→			1.368		2
*O*8	0.221 ×2↓ ×2→		0.352		1.206	2
∑	2	2	2	5	5	

400802: $KCu^{2+}{}_5V^{5+}{}_3O_{13}$										
	K	$Cu1$	$Cu2$	$Cu3$	$Cu4$	$Cu5$	$V1$	$V2$	$V3$	Σ
$O1$			0.303		0.303	0.242			1.151	2
$O2$		0.450	0.494	0.562	0.494					2
$O3$	0.094	0.475					1.431			2
$O4$	-0.013	0.367			0.412			1.234		2
$O5$		0.341		0.452				1.207		2
$O6$			0.251	0.318	0.251		1.179			2
$O7$	0.078 ×3↓ ×3→					0.441		1.325		2
$O8$	0.334		0.273		0.273				1.121	2
$O9$			0.267	0.334		0.205	1.195			2
$O10$	0.091 ×2↓ ×2→					0.454			1.364	2
$O11$				0.334	0.267	0.205	1.195			2
$O12$	0.091 ×2↓ ×2→					0.454			1.364	2
$O13$	-0.013	0.367	0.412					1.234		2
Σ	1	2	2	2	2	2	5	5	5	

401042: $K_4(Cu^{2+}V^{5+}{}_5O_{15}Cl)$					
	K	Cu	$V1$	$V2$	Σ
$O1$	0.206 ×3↓ ×3→		1.381		2
$O2$	0.158 ×2↓ ×2→	0.352 ×4↓	1.332		2
$O3$	0.025 ×2↓ ×2→		1.200	0.750 ×4↓	2
$O4$	-0.087 ×2→		1.087 ×2→		2
$O5$				2.000	2
Cl	0.102 ×4→	0.296 ×2↓ ×2→			1
Σ	1	2	5	5	

401951: $Zn_5Mn^{4+}(BO_3)_2O_4$						
	*Zn*1	*Zn*2	*Zn*3	*Zn*4/*Mn*	*B*	Σ
*O*1		0.288 ×4↓ ×2→		0.427	0.988	2
*O*2	0.427	0.425 ×2↓		0.574 ×2↓ ×2→		2
*O*3	0.326 ×2↓ ×2→		0.322 ×2↓		1.025	2
*O*4	0.343		0.339 ×4↓ ×2→	0.490 ×2↓ ×2→		2
*O*5	0.289 ×2↓ ×2→			0.436	0.987	2
Σ	2	2	2	3	3	

405153: $Ba_2(Nb^{5+}{}_2Te^{6+}O_{10})$				
	Ba	*Nb*	*Te*	Σ
*O*1	0.190 ×2↓ ×2→	0.810 ×2↓ ×2→		2
*O*2	0.175	0.796	1.029 ×2↓	2
*O*3	0.131 ×2↓ ×2→	0.752	0.985 ×2↓	2
*O*4	0.131 ×2↓ ×2→	0.752	0.985 ×2↓	2
*O*5	0.460 ×2↓ ×2→	1.080		2
Σ	2	5	6	

405329: $PbCu^{2+}(Cu^{2+}Te^{6+}O_7)$					
	*Pb*1	*Cu*1	*Cu*2	*Te*	Σ
*O*1		0.321 ×2→	0.372	0.986	2
*O*2	0.405	0.515 ×2→	0.565		2
*O*3		0.214 ×2↓ ×4→	0.265	0.879	2
*O*4	0.239 ×2↓	0.348	0.399 ×2↓	1.014 ×2↓	2
*O*5	0.279 ×4↓ ×2→	0.388		1.054 ×2↓	2
Σ	2	2	2	6	

409521: $Hg^{2+}(V^{5+}{}_2O_6)$			
	Hg	*V*	Σ
*O*1	0.167 ×2↓	0.917 ×2↓ ×2→	2
*O*2	0.417 ×4↓ ×2→	1.167	2
*O*3		0.667 ×3↓ ×3→	2
Σ	2	5	

409745: $Rb_2(Cr^{6+}{}_2O_7)$					
	*Ag*1	*Ag*2	*Cr*1	*Cr*2	Σ
*O*1	-0.225		1.131	1.094	2
*O*2		0.162 ×2↓ ×2→	1.676		2
*O*3	0.267	0.110	1.623		2
*O*4	0.215 ×2↓ ×2→		1.571		2
*O*5	0.249	0.091 ×2↓ ×2→		1.568	2
*O*6	0.279	0.122		1.599	2
*O*7		0.261		1.739	2
Σ	1	1	6	6	

411285: $KY(W^{6+}O_4)_2$				
	K	*Y*	*W*	Σ
*O*1	0.191 ×4↓ ×2→	0.470 ×2↓	1.148	2
*O*2	-0.048 ×2↓	0.230 ×2↓	0.909 ×2↓ ×2→	2
*O*3	0.122 ×2↓	0.400 ×4↓ ×2→	1.078	2
*O*4	0.022 ×4↓ ×2→		0.978 ×2↓ ×2→	2
Σ	1	3	6	

413000: $Rb_2Se^{4+}Mo^{6+}O_6$					
	*Rb*1	*Rb*2	*Mo*	*Se*	Σ
*O*1	0.038 ×2↓ ×2→		0.962 ×2↓ ×2→		2
*O*2	-0.039 ×4↓ ×2→	-0.074 ×2↓	0.885 ×2↓	1.267 ×2↓	2
*O*3	0.230 ×4↓ ×2→	0.194 ×4↓ ×2→	1.153 ×2↓		2
*O*4	0.160	0.125 ×3↓ ×3→		1.466	2
Σ	1	1	6	4	

415239: $Pd^{2+}(P_2O_7)$				
	*Pd*1	*Pd*2	*P*	Σ
*O*1	0.400 ×2↓	0.400 ×2↓	1.200	2
*O*2	0.600 ×2↓		1.400	2
*O*3			1.000 ×2→	2
*O*4		0.600 ×2↓	1.400	2
Σ	2	2	5	

415427: $Ce^{3+}Ta^{5+}O_4$			
	Ce	*Ta*	Σ
*O*1	0.353	0.824 ×2↓ ×2→	2
*O*2	0.353	0.824 ×2↓ ×2→	2
*O*3	0.382 ×3↓ ×3→	0.853	2
*O*4	0.382 ×3↓ ×3→	0.853	2
Σ	3	5	

415460: $Yb(Ta^{5+}O_4)$			
	Yb	*Ta*	Σ
*O*1	0.500 ×4↓ ×2→	1.000 ×2↓	2
*O*2	0.250 ×4↓ ×2→	0.750 ×4↓ ×2→	2
Σ	3	5	

416590: $Li(Nb^{5+}U^{6+}O_6)$				
	Li	*Nb*	*U*	Σ
*O*1	0.240 ×2↓ ×2→		1.520	2
*O*2		0.971	1.029	2
*O*3		0.647 ×2↓ ×2→	0.706	2
*O*4		0.627	0.686 ×2↓ ×2→	2
*O*5		0.627	0.686 ×2↓ ×2→	2
*O*6	0.260 ×2↓ ×2→	1.480		2
Σ	1	5	6	

417072: $RbW^{5+}O(P_2O_7)$					
	Rb	*W*	*P*1	*P*2	Σ
*O*1	0.394 ×2↓ ×2→	1.212			2
*O*2	-0.030	0.788		1.242	2
*O*3	-0.030	0.788		1.242	2
*O*4		0.727	1.273		2
*O*5		0.727	1.273		2
*O*6	-0.061	0.758	1.303		2
*O*7	-0.212		1.152	1.061	2
*O*8	0.182 ×3↓ ×3→			1.455	2
Σ	1	5	5	5	

Table S2 Values of Δ_{topol} and Δ_{cryst} for 266 transition metal coordination polyhedra taken from 140 crystal structures

	Oxidation state	Coordination number	Δ_{topol}	Δ_{cryst}	ICSD code
Sc	3	6	0.019	0.020	65407
		6	0.067	0.038	200128
		8	0.033	0.102	100278
Ti	3	6	0.095	0.030	72714
		6	0.095	0.042	""
	4	4	0.074	0.107	78842
		5	0.140	0.109	33800
		5	0.098	0.217	72682
		6	0.107	0.102	36608
		6	0.032	0.080	75583
		6	0.055	0.162	""
		6	0.048	0.090	82488
		6	0.235	0.250	280501
		6	0.190	0.295	""
V	3	6	0.067	0.018	59244
		6	0.111	0.030	64634
		6	0.040	0.071	82685
		6	0.072	0.052	""
	4	5	0.480	0.065	64634
		5	0.210	0.186	249142
		6	0.034	0.074	71450
		6	0.222	0.219	72886
		6	0.444	0.123	92317
		6	0.444	0.117	201658
	5	4	0.115	0.033	40312
		4	0.112	0.070	""
		4	0.021	0.156	67726

		4	0.099	0.129	73686
		4	0.115	0.093	""
		4	0.226	0.071	""
		4	0.274	0.154	""
		4	0.135	0.039	171028
		4	0.137	0.069	""
		4	0.091	0.111	400802
		4	0.038	0.040	""
		4	0.114	0.217	""
		4	0.107	0.104	401042
		5	0.250	0.112	50010
		5	0.400	0.179	280775
		5	0.400	0.073	401042
		6	0.167	0.336	40850
		6	0.292	0.314	50010
		6	0.444	0.171	89466
		6	0.444	0.160	""
		6	0.187	0.143	95929
		6	0.361	0.097	""
		6	0.325	0.027	""
		6	0.294	0.129	""
		6	0.305	0.060	""
		6	0.200	0.158	99594
		6	0.167	0.306	409521
Cr	2	5	0.057	0.076	73261
		5	0.020	0.077	280309
		6	0.170	0.118	72302
		6	0.156	0.041	73261
	3	6	0.056	0.065	8269
		6	0.055	0.067	72302
		6	0.091	0.052	""

		6	0.098	0.037	""
		6	0.074	0.038	73261
		6	0.089	0.061	""
		6	0.077	0.068	247056
	6	4	0.250	0.144	59819
		4	0.039	0.045	""
		4	0.053	0.080	""
		4	0.039	0.014	""
		4	0.250	0.164	201793
		4	0.000	0.081	""
		4	0.185	0.074	409745
		4	0.203	0.081	""
Mn	2	6	0.111	0.160	40850
		6	0.111	0.099	85042
		6	0.133	0.183	99594
		6	0.028	0.061	100082
		6	0.028	0.046	""
		6	0.072	0.110	156736
	3	4	0.040	0.092	34392
		5	0.053	0.176	280589
		6	0.077	0.211	24973
		6	0.008	0.211	80430
		6	0.039	0.186	95493
	5	4	0.250	0.097	97525
	7	4	0.000	0.028	89506
Fe	2	6	0.000	0.067	17062
		6	0.000	0.071	""
		6	0.148	0.148	50038
	3	4	0.011	0.075	83285
		4	0.125	0.039	""
		4	0.028	0.029	280902

		5	0.073	0.050	96359
		6	0.111	0.026	83285
		6	0.000	0.003	""
		6	0.114	0.070	96359
		6	0.099	0.066	""
		6	0.067	0.038	157733
		6	0.167	0.090	280902
Co	2	4	0.071	0.072	81473
		4	0.026	0.082	""
		5	0.032	0.070	81473
		6	0.026	0.044	20670
		6	0.063	0.028	82403
		6	0.086	0.063	400328
		6	0.049	0.053	400764
		6	0.053	0.089	""
		8	0.013	0.091	250466
	3	6	0.056	0.126	72872
Ni	2	4	0.016	0.052	14159
		6	0.039	0.024	65476
		6	0.007	0.017	""
		6	0.061	0.031	""
		6	0.035	0.035	""
		6	0.034	0.044	79702
Cu	2	4	0.112	0.055	1292
		4	0.000	0.028	201733
		5	0.060	0.100	1292
		5	0.099	0.033	""
		5	0.078	0.076	50459
		5	0.050	0.133	""
		5	0.051	0.063	""
		5	0.016	0.059	63103

		5	0.019	0.101	65614
		5	0.058	0.122	171028
		5	0.071	0.093	201733
		5	0.087	0.088	400438
		5	0.050	0.063	400802
		5	0.086	0.068	""
		5	0.066	0.091	405329
		6	0.063	0.070	2279
		6	0.084	0.039	67726
		6	0.084	0.041	""
		6	0.096	0.127	171028
		6	0.046	0.161	400438
		6	0.080	0.089	400802
		6	0.080	0.087	""
		6	0.116	0.115	""
		6	0.025	0.143	401042
		6	0.084	0.083	405329
	3	4	0.028	0.021	65237
		4	0.028	0.023	""
		4	0.028	0.018	""
Zn	2	4	0.250	0.277	85735
		5	0.021	0.075	40312
		6	0.077	0.129	85042
		6	0.100	0.055	280902
		6	0.034	0.038	401951
		6	0.061	0.022	""
		6	0.007	0.007	""
Y	3	6	0.040	0.046	85497
		7	0.001	0.044	63103
		7	0.004	0.056	""
		7	0.020	0.077	65614

		8	0.111	0.083	15505
		8	0.085	0.071	""
		8	0.058	0.088	71562
		8	0.072	0.131	411285
		9	0.078	0.110	15505
		10	0.086	0.079	20670
Zr	4	6	0.021	0.054	15545
		6	0.000	0.274	55272
		6	0.000	0.030	65512
		6	0.000	0.041	""
		6	0.221	0.186	100158
		8	0.063	0.040	82488
Nb	4	6	0.000	0.085	88879
		6	0.000	0.085	""
	5	5	0.023	0.094	24819
		5	0.035	0.112	94743
		6	0.061	0.210	33783
		6	0.111	0.095	36626
		6	0.071	0.189	50038
		6	0.182	0.218	""
		6	0.019	0.071	62577
		6	0.227	0.098	""
		6	0.125	0.174	75264
		6	0.030	0.056	79734
		6	0.167	0.204	85735
		6	0.083	0.161	91748
		6	0.096	0.198	100158
		6	0.184	0.178	200854
		6	0.180	0.179	""
		6	0.082	0.212	405153
		6	0.261	0.266	416590

		7	0.078	0.192	33783
		7	0.163	0.133	""
Mo	4	6	0.111	0.023	96454
	5	6	0.389	0.075	69088
		6	0.058	0.159	281197
	6	4	0.250	0.109	65512
		4	0.067	0.040	68279
		4	0.052	0.079	""
		4	0.087	0.134	""
		4	0.099	0.056	247056
		4	0.183	0.178	""
		4	0.000	0.055	280775
		4	0.094	0.047	281210
		5	0.161	0.176	66994
		5	0.232	0.256	79517
		5	0.367	0.159	280066
		6	0.418	0.235	68279
		6	0.134	0.204	79517
		6	0.186	0.329	90110
		6	0.033	0.531	170119
		6	0.152	0.340	171758
		6	0.224	0.261	""
		6	0.322	0.215	280066
		6	0.282	0.214	281503
		6	0.328	0.161	""
		6	0.102	0.496	413000
Ru	4	6	0.034	0.141	33802
	5	6	0.048	0.156	73183
		6	0.067	0.140	97525
Pd	2	4	0.100	0.058	415239
		4	0.100	0.065	""

		4	0.000	0.033	72312
		4	0.000	0.042	""
		4	0.122	0.103	""
		6	0.084	0.085	72312
Cd	2	6	0.026	0.061	35084
		6	0.062	0.020	200743
		8	0.022	0.108	35084
		8	0.036	0.027	35407
Hf	4	6	0.017	0.178	33194
		6	0.003	0.162	""
		6	0.063	0.026	65476
		8	0.000	0.092	59111
Ta	5	6	0.111	0.092	80423
		6	0.183	0.147	85042
		6	0.000	0.060	203232
		6	0.000	0.245	280154
		6	0.013	0.099	415427
		6	0.111	0.195	415460
		7	0.092	0.185	203232
W	5	6	0.056	0.026	8269
		6	0.111	0.046	203048
		6	0.167	0.098	202414
		6	0.126	0.067	417072
	6	4	0.019	0.054	71562
		4	0.071	0.141	78180
		5	0.008	0.086	40249
		5	0.149	0.169	78180
		6	0.204	0.170	68614
		6	0.183	0.239	""
		6	0.122	0.262	79702
		6	0.000	0.319	86144

		6	0.075	0.357	411285
Re	5	6	0.056	0.051	72872
	7	4	0.125	0.084	92317
		4	0.250	0.149	""
		5	0.007	0.235	100571
		6	0.025	0.128	15505
		6	0.040	0.077	92508
Os	7	6	0.000	0.105	49746
	8	4	0.000	0.077	20611
		5	0.146	0.248	20611
		6	0.200	0.320	20540
Ir	4	6	0.002	0.046	33863
		6	0.077	0.193	""
Pt	2	4	0.000	0.010	35407
	4	6	0.032	0.017	35407
		6	0.015	0.030	63103
		6	0.005	0.075	65614
Au	3	4	0.000	0.093	92489
Hg	2	2	0.000	0.203	72312
		3	0.069	0.204	72312
		6	0.000	0.010	1640
		6	0.063	0.066	280292
Average:			0.102	0.113	
